# Exploring interpretability in deep learning prediction of successful ablation therapy for atrial fibrillation

**DOI:** 10.3389/fphys.2023.1054401

**Published:** 2023-03-14

**Authors:** Shaheim Ogbomo-Harmitt, Marica Muffoletto, Aya Zeidan, Ahmed Qureshi, Andrew P. King, Oleg Aslanidi

**Affiliations:** School of Biomedical Engineering and Imaging Sciences, King’s College London, St Thomas’ Hospital, London, United Kingdom

**Keywords:** atrial fibrillation, catheter ablation, medical imaging, cardiac modelling, deep learning, interpretability

## Abstract

***Background:*** Radiofrequency catheter ablation (RFCA) therapy is the first-line treatment for atrial fibrillation (AF), the most common type of cardiac arrhythmia globally. However, the procedure currently has low success rates in dealing with persistent AF, with a reoccurrence rate of ∼50% post-ablation. Therefore, deep learning (DL) has increasingly been applied to improve RFCA treatment for AF. However, for a clinician to trust the prediction of a DL model, its decision process needs to be interpretable and have biomedical relevance.

***Aim:*** This study explores interpretability in DL prediction of successful RFCA therapy for AF and evaluates if pro-arrhythmogenic regions in the left atrium (LA) were used in its decision process.

***Methods:*** AF and its termination by RFCA have been simulated in MRI-derived 2D LA tissue models with segmented fibrotic regions (n = 187). Three ablation strategies were applied for each LA model: pulmonary vein isolation (PVI), fibrosis-based ablation (FIBRO) and a rotor-based ablation (ROTOR). The DL model was trained to predict the success of each RFCA strategy for each LA model. Three feature attribution (FA) map methods were then used to investigate interpretability of the DL model: GradCAM, Occlusions and LIME.

***Results:*** The developed DL model had an AUC (area under the receiver operating characteristic curve) of 0.78 ± 0.04 for predicting the success of the PVI strategy, 0.92 ± 0.02 for FIBRO and 0.77 ± 0.02 for ROTOR. GradCAM had the highest percentage of informative regions in the FA maps (62% for FIBRO and 71% for ROTOR) that coincided with the successful RFCA lesions known from the 2D LA simulations, but unseen by the DL model. Moreover, GradCAM had the smallest coincidence of informative regions of the FA maps with non-arrhythmogenic regions (25% for FIBRO and 27% for ROTOR).

***Conclusion:*** The most informative regions of the FA maps coincided with pro-arrhythmogenic regions, suggesting that the DL model leveraged structural features of MRI images to identify such regions and make its prediction. In the future, this technique could provide a clinician with a trustworthy decision support tool.

## 1 Introduction

Atrial fibrillation (AF), the rapid, uncoordinated contraction of the atria, is a heart condition that affects 33 million people worldwide—making it the most common type of cardiac arrhythmia globally (Hart and Halperin, 2001; Chugh et al., 2014). Currently, the precise mechanisms of AF are unclear. However, there is evidence that ectopic electrical beats originating from the pulmonary veins (PVs) can trigger AF (Chen et al., 1999). The triggers can then generate re-entrant drivers (rotors) that sustain AF, and spatial fibrosis distributions in the left atria (LA) have been demonstrated to facilitate such drivers (Morgan et al., 2016; Roy et al., 2020). A common treatment for AF is radiofrequency catheter ablation (RFCA) therapy. RFCA involves using induced heat from a rapidly alternating current in a catheter to ablate (isolate or destroy) the arrhythmogenic area of atrial tissue that harbours triggers or rotors, thus restoring sinus rhythm and the mechanical function of the heart (Townsend and Sabiston, 2001). Presently, the success rate of RFCA is ∼70% for paroxysmal AF—which is relatively high (Oketani et al., 2012). However, the procedure is much less successful when dealing with persistent AF, which has a reoccurrence rate of ∼75% post-intervention. Therefore, with the high reoccurrence rate of AF, there is a need for improvements within (Wang et al., 2017; Yubing et al., 2018).

Image-based computational modelling has been used to understand the structure-function relationship that determines re-entrant atrial drivers for AF with the aim of improving RFCA outcomes. As a result, computational methods have been introduced to improve RFCA outcomes, ultimately leading to the FIRM (Focal Impulse and Rotor Modulation) mapping, which locates rotational activity around a centre (rotor) from electroanatomical maps (Narayan et al., 2012a). The CONFIRM trial showed patients that underwent FIRM-guided ablation maintained a higher freedom of AF (AF termination in 86% of patients) when compared to patients with conventional ablation strategy (AF termination in 20% of patients) (Narayan et al., 2012b). However, the multicentre REAFFIRM trial did not show evidence that FIRM-guided ablation strategy is superior to pulmonary vein isolation (PVI) (Zhao et al., 2019).

With the recent rise of artificial intelligence (AI), machine and deep learning (DL) have been applied to patient medical imaging data and computational cardiac modelling with the aim to develop more effective RFCA treatments. The applications of AI include predicting AF reoccurrence post-RFCA and the origins of AF triggers and ablation (Kim et al., 2020; Liu et al., 2020; Firouznia et al., 2021; Roney et al., 2022). Furthermore, Luongo et al. have applied machine learning to predict AF ablation targets, but used 12-lead ECG data instead of medical imaging (Luongo et al., 2021). Other studies have also leveraged the power of AI in AF by using DL with ECG data to estimate atrial fibrosis and to classify AF from atrial flutter or tachycardia (Nagel et al., 2021; Rodrigo et al., 2022). Zololotarev et al. applied AI to identify AF drivers from multi-electrode mapping, with the AI model then validated using optical mapping; the results were comparable to the state-of-the-art with higher computational efficiency (Zolotarev et al., 2020). Popescu et al. applied DL for arrhythmic sudden death prediction from clinical and imaging data, which proved successful and achieved a concordance index of 0.83 and 0.74, and 10-year integrated Brier score of 0.12 and 0.14, respectively (Popescu et al., 2022).

However, DL is limited by its black-box nature. This is an issue when considering the European Union’s General Data Protection Regulation (GDPR), as any algorithmic decision used in patient care requires an explanation for transparency (Mourby et al., 2021). Moreover, clinicians have also argued that if AI can outperform human diagnosis, understanding the AI model’s decision process will be beneficial in discovering new biological processes and furthering medical knowledge (Watson et al., 2019). Moreover, it will increase confidence in the AI-generated results, which means the clinicians are more likely to trust and leverage them. Hence, this has led to the growing field of deep learning interpretability for medical imaging analysis, where methods such as concept learning models, latent space interpretation and attribution maps have been applied to many medical fields (Salahuddin et al., 2022). Organisations have also expressed an interest in AI interpretability, e.g., the Avicenna Alliance (AA) and the Virtual Physiological Human Institute (VPHI). The AA and VPHI aims are to promote the synergy of AI and *in silico* modelling into healthcare, while providing policymakers and regulators with directions towards applying these technologies safely in clinics, including AI interpretability (Geris et al., 2022).

Muffoletto et al. were the first to apply DL to directly informing a clinician to treat AF using RFCA therapy and developed a convolutional neural network (CNN) to predict suitable *in silico* ablation strategies for a given patient, using synthetic tissue-based atrial models with randomly distributed fibrotic patches. The approach proved effective (79% accuracy) and illustrated the proof-of-concept (Muffoletto et al., 2019). Ultimately, this led to the approach being applied to MRI-derived data to predict the patient-specific optimal RFCA strategy. As a result, the developed CNN had a 100% accuracy for classifying optimal fibrosis- (FIBRO) and rotor-based (ROTOR) strategies success and 33% accuracy for the PVI strategy (Muffoletto et al., 2021).

Currently, research into interpretability for DL-based AF management is very limited. For example, one study by Alhusseini et al. used gradient-weighted class activation mapping (GradCAM) to show that their feature attribution (FA) map closely replicated rules used by clinicians. However, only one method was validated within this study, and a comparison with other methods was not investigated. Furthermore, the study used spatial maps of the activation phase derived from electrocardiogram data from a basket catheter. Hence, there has been no investigation into DL interpretability for models which use medical imaging data to make explainable predictions for cardiac arrhythmias and anti-arrhythmic treatments (Alhusseini et al., 2020).

In this study, we present a novel qualitative and quantitative comparison of established DL interpretability methods for medical imaging and image-based cardiac modelling of RFCA, as well as new quantitative metrics to assess interpretability of FA maps for the image-based cardiac models.

## 2 Methods

### 2.1 Overview

We propose a DL approach to 1) accurately predict the outcomes of RFCA therapy based on image-based modelling and simulations and 2) interpret the decision process of the DL model. To achieve this, standardised 2D LA models with patient-specific distributions of fibrosis were derived from late gadolinium-enhanced (LGE) MR imaging data. Simulations of AF and its termination with three RFCA strategies were performed, the DL model was applied to predict the success of each strategy, and the RFCA simulation results were compared with DL interpretability maps to identify proarrhythmogenic locations. Three established interpretability approaches were also compared qualitatively and quantitatively to interpret the DL model’s predictions.

### 2.2 Data acquisition and pre-processing

The datasets used in this study were derived from 122 LGE MRI patient scans: 86 datasets with spatial resolution of 0.625 × 0.625 × 0.625 ${mm}^{3}$ were acquired from the Atrial Segmentation Challenge at the STACOM 2018 workshop (Xiong et al., 2021); additionally, 36 LGE MRI images were collected at St. Thomas’ Hospital London with resolution of 1.3 × 1.3 × 4 ${mm}^{3}$ (specifically, 18 AF patients were scanned both pre-and post-intervention) (Chubb et al., 2018).

**FIGURE 1:**
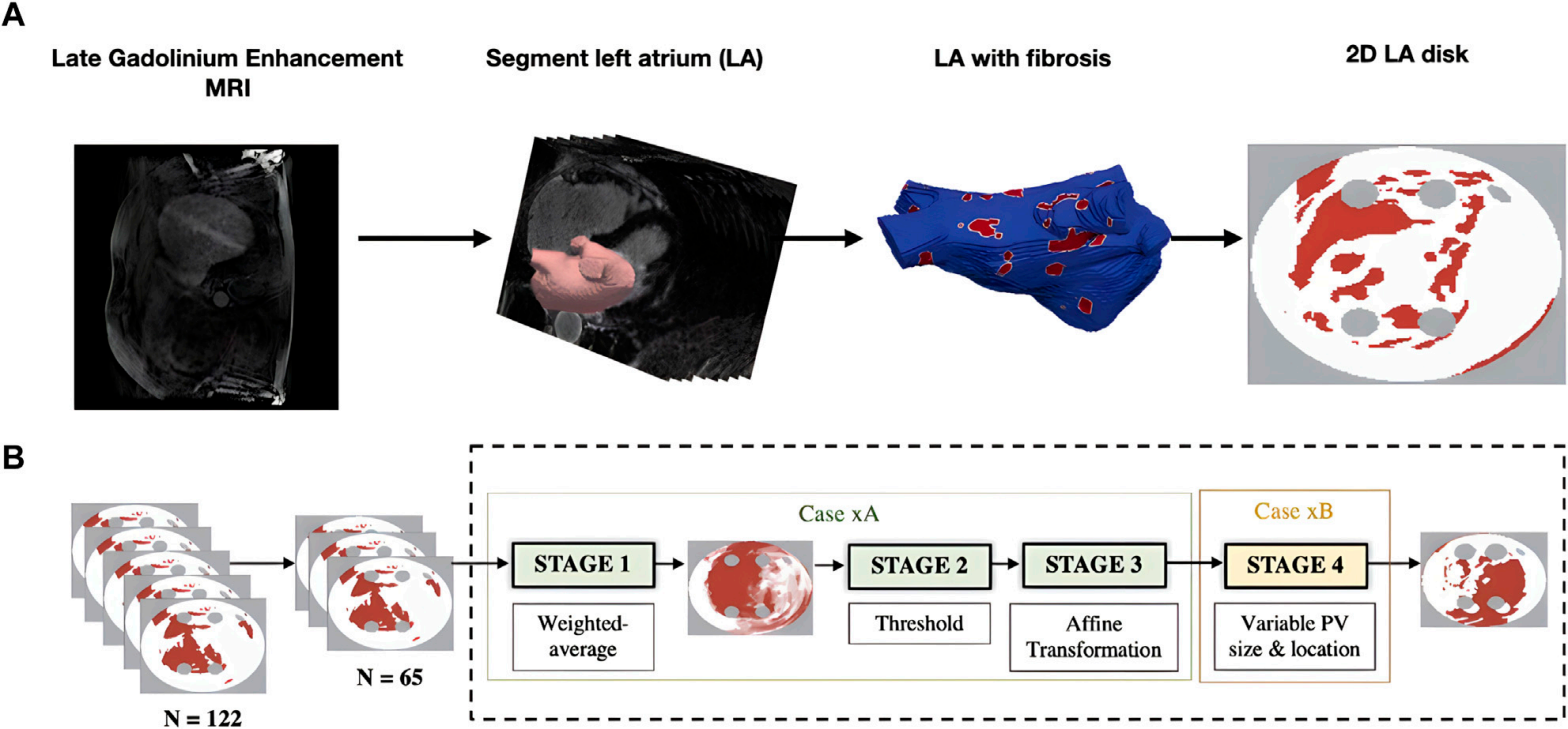
Diagram of MRI-derived 2D LA tissue disk. **(A)**. Workflow of 2D LA tissue generation pipeline. The figure illustrates the process of how the 2D LA tissue models are obtained from LGE MRI by LA segmentation, thresholding fibrosis from healthy tissue and mapping onto 2D LA tissues. **(B)**. Workflow for generating synthetic tissues. 65 tissues were randomly selected from the total dataset of 122 real tissues. These 65 tissues were used to generate the synthetic images by iterating overstages 1 to 4 (199 times) to create a virtual cohort of 199 tissues. ‘Case xA’ denotes the combination of data augmentation techniques used to create the synthetic fibrosis distributions. ‘Case xB’ determines how the PV sizes and locations were varied from those in the standardised discs.

Generating 2D LA models with fibrosis first required manual segmentation of patient LGE MRI data to produce 3D patient-specific endocardial LA surface meshes. The LGE MRI image intensities were then mapped to these models and the image intensity ratio thresholding technique was applied to quantify and visualise LA fibrosis (Roy et al., 2020). Finally, the 3D LA fibrosis maps were unwrapped using the LA standardised unfold mapping technique to produce models in the 2D LA disk format for use as input to the DL network, as shown in Figure 1A (Williams et al., 2017; Qureshi et al., 2020).

Furthermore, to increase the size of the dataset, synthetic 2D LA disks were generated by weighted-averaging of the patient datasets to vary the fibrosis distribution and PVs. The creation of synthetic disks consisted of three steps. First, 65 MRI images were extracted from the STACOM 2018 dataset and were each weighted by assigning a random weight (between 0 and 1) to all voxels of a given image; the weighted-average of all images was thresholded (Case xA in Figure 1B). This number was chosen as less than 65 would result in low variability in the synthetic tissues and more than 65 would result in most of the synthetic tissues being covered in fibrosis. Supplementary Figure S1 illustrates that selecting the 65 LA tissues in generating the synthetic LA tissues would result in a mean fibrotic tissue percentage of approximately 50%. Thus, 65 corresponds to a folding point of this sigmoidal dependence, and any number above 65 would lead to a majority of tissue being fibrotic. Then the extracted fibrosis distribution was further augmented by applying one or multiple affine transformations (translation, rotation and flipping). The fibrosis threshold value and the types of transformation were randomly selected. Lastly, the PVs were varied by assigning one of 6 different variants, which included changing PV size and position (Case xB in Figure 1B) (Muffoletto et al., 2021). This resulted in a total of 199 synthetic 2D LA tissue models in addition to the 122 patient-specific models, totalling 321 2D LA tissue models.

### 2.3 Atrial tissue modelling and AF simulation

Eq. 1 represents the Fenton-Karma semi-physiological model, which consists of three ionic currents representing the overall ion current in the electrical dynamics of atria cells; $I_{fi}$ represents the fast inward current ${Na}^{+}$ **,** $I_{so}$ is the slow outward current $K^{+}$ and $I_{si}$ is the slow inward current ${Ca}^{+}$ (Fenton and Karma, 1998):

$$I_{ion}=I_{fi}+I_{so}+I_{si}$$ (1)

Eq. 2 is the standard monodomain equation to describe electrical wave propagation.

$$\frac{\partial V_{m}}{\partial t}=\nabla .D\nabla V_{m}-\frac{I_{ion}}{C_{m}}$$ (2)

$V_{m}$ is the membrane potential, $C_{m}$ is the membrane capacitance, D is a tensor that represents the diffusion of the electrical coupling within tissue. Eq. 2 with ion current determined in Equation 1 was solved using the forward Euler method with a finite-difference approximation of the Laplacian. Therefore, Equation 1 and Equation 2 were solved using each 2D tissue disk as a spatial domain to simulate electrical waves sustaining AF. Such waves in the form of rotors were generated using the standard cross-field protocol at 28 *ms* into the simulation (Tobón et al., 2014). The numerical integration steps were 0.01 ms time step and 0.3 *mm* spatial step. Additionally, healthy tissue had a D value of 0.1 ${mm}^{2}s^{-1}$ to match the physiological value of healthy myocardium tissue. Fibrotic tissue had D value of 0.015 ${mm}^{2}s^{-1}$ .

The three ablation strategies were simulated to terminate persistent AF: PVI, FIBRO and ROTOR strategies; details of the simulations have been published previously (Muffoletto et al., 2021). The FIBRO strategy involved ablating the perimeter of the fibrotic tissue, while PVI consisted of ablating the circumference of the PVs and ROTOR ablated the phase singularities of the electrical wave. The ablation strategy was deemed successful for a tissue if AF was terminated within 2000 *ms* and less than 40% of the tissue was ablated (Muffoletto et al., 2021). Therefore, using the stated simulation pipeline, the success of the three RFCA strategies was determined for AF simulations in the 2D LA tissues (including patient MRI derived and synthetic data). Furthermore, since multiple strategies can be successful/unsuccessful for a given 2D LA tissue, the classification task was multi-label.

### 2.4 Deep learning

**FIGURE 2:**
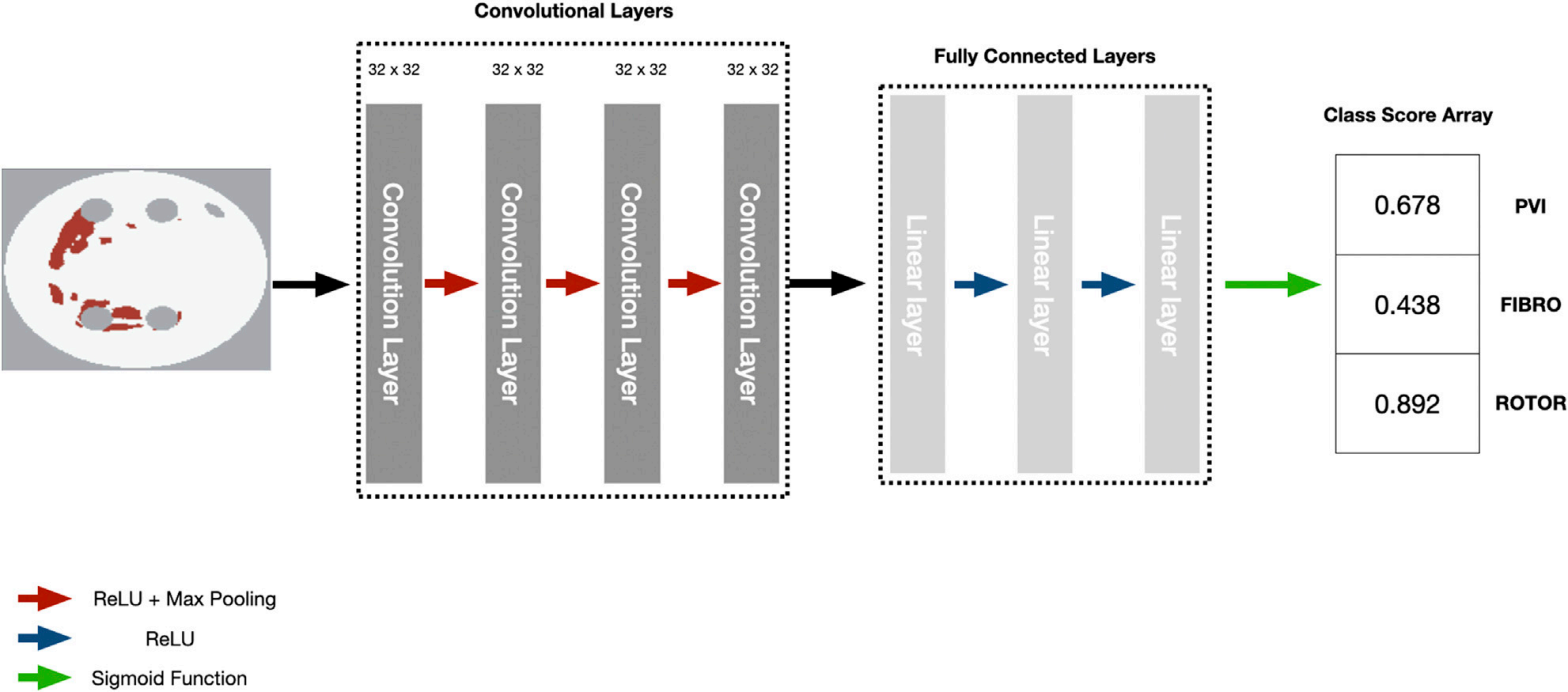
Diagram of CNN with parameters to predict RFCA simulation strategy success from 2D LA tissue.

We employed the CNN with hyperparameters (parameters and number of convolutional and fully connected layers) based on the study by Muffoletto et al. as the basis of our interpretability framework (Muffoletto et al., 2021). The hyperparameters were tuned by Muffoletto et al. by performing 24 experiments which involved changing number of layers, filter size of convolutional layers and dropout rate. The optimal hyperparameters were chosen by selecting the DL model with the highest average accuracy across a 5-fold cross-validation. The CNN consisted of four convolutional layers of 32 × 32 filters, each followed by Rectified Linear Unit (ReLU) activation and max pooling with a pool size of two. These are followed by three linear layers (2048, 128 and 3 units, respectively) and another ReLU activation. A Dropout layer followed this at a rate of 0.8 and a sigmoid function (Paszke et al., 2019). Since we address a multi-label classification problem (i.e., multiple ablation strategies), we modified the loss function to be a mean-squared error tailored to perform multi-label classification for the three ablation strategies (Figure 2).

$$MSE\left(y_{score},y\right)=\overset{N}{\underset{i=0}{\sum }}\frac{y_{score}^{i}-y^{i}}{N}$$ (3)

Eq. 3 is the mean-squared error function formulation, where $y_{score}$ is the predicted class score array and y is the RFCA strategy success ground truth (where 1 = success and 0 = unsuccessful). Here, N represents the number of classes/strategies (three in this study) and i is the index of a class in the class score array. To train and effectively test the CNN, a leave-one-out cross-validation was used where the total dataset was split into two sets: a hold-out test set and training set. The training set was then split into five folds, where four folds were used to train the CNN, and the last fold was used as a validation set to select the optimal CNN model state (i.e. the model with the lowest loss during training) (Raschka, 2018; Muffoletto et al., 2021). In total, there were 271 2D LA tissues in the leave-one-out cross-validation dataset (96 MRI derived and 175 synthetic). Within each fold the DL model was trained for 100 epochs using an ADAM optimiser with a learning rate of 1e-4 (Kingma and Ba, 2014). For each fold, the optimal model was tested on the hold-out test set of 50 2D LA tissues (26 MRI derived and 24 synthetic) from the total dataset to evaluate the DL model’s performance. Pre- and post-ablation images were not split during cross-validation, as there was little similarity between the respective fibrosis distributions (see Supplementary Materials Section 2 and Supplementary Figure S2).

### 2.5 Interpretability

Three popular local *post hoc* interpretability methods were used to interpret the CNN’s predictions - GradCAM, occlusions and local interpretable model-agnostic explanations (LIME) (Zeiler and Fergus, 2014; Ribeiro et al., 2016; Selvaraju et al., 2017; Kokhlikyan et al., 2020). GradCAM and LIME were chosen as they are widely used saliency maps in DL medical image analysis (Magesh et al., 2020; Graziani et al., 2021; Patel et al., 2021; Mahapatra et al., 2022), while occlusions is one of the first saliency map methods used for DL computer vision. Each method evaluates feature attribution using different approaches: GradCAM uses gradient information, LIME uses an interpretable model within a local space and the occlusions method uses perturbations.

The DL model state from the most accurate fold of the leave-one-out cross-validation was used to produce the FA maps for the three methods on the hold-out test set. The GradCAM method was applied to the last convolutional layer of the CNN. Each FA map was thresholded above the respective map’s average FA to highlight the most informative features. Three metrics were evaluated to quantitatively analyse the informative regions of each FA map: Jacquard index (IoU), lesion percentage and non-arrhythmogenic tissue (NAT) percentage. The IoU was evaluated by calculating IoU of the informative regions of a FA map and lesions of a given ablation strategy. Lesion percentage was evaluated by calculating the percentage of lesions of a given ablation strategy within the informative regions.

The motivation for analysing the lesion percentage was to determine if the DL model focused on clinically relevant features. The number of the lesions (unseen by the DL model but known from simulations–and known to clinicians when ablating a patient) found in a FA map’s informative region is a relevant metric, as such lesions are associated with arrhythmogenic regions in atrial tissue. Thus, PVI lesions isolate the area of the initial arrhythmogenic triggers, FIBRO lesions aim to isolate the fibrotic tissue border where AF reentrant drivers commonly reside, and ROTOR lesions directly target such reentrant drivers. Therefore, the ability of DL model to predict lesion locations (again, without seeing such lesions during training) should help the clinician to understand and trust these predictions.

Lastly, the NAT percentage was calculated by finding the percentage of healthy tissue (with no lesions or fibrosis) within the informative regions of a FA map. NAT percentage was evaluated to assess how much of the clinically irrelevant features were highlighted as informative by the DL model.

#### 2.5.1 GradCAM

GradCAM uses the gradient from a given convolutional layer to measure FA for a particular decision of interest. GradCAM is an improvement of the class activation map (CAM) method. CAM produces a localisation map for an image classification model, utilising a specific architecture where globally averaged pooled convolutional feature maps are fed directly into a softmax layer. GradCAM improves on CAM by being architecture-independent, and it can be applied to any CNN. Furthermore, a study by Adebayo et al. implemented a sanity check of GradCAM through a model parameter and data randomisation test. It demonstrated that GradCAM’s saliency maps could support tasks that require explanations that are faithful to the model and the data generation process (Adebayo et al., 2018).

$$\alpha^{c}=\frac{1}{z}\underset{i}{\sum }\underset{j}{\sum }\frac{\partial y^{c}}{\partial A_{ij}}$$ (4)

Feature attribution, $\alpha_{ij}^{c}$ (***i*** and ***j*** are the indices of the feature in a FA map), of a given class ***c*** is calculated in GradCAM by evaluating the partial derivative of the score of class ***c*** and a feature from activation map of a given convolutional layer $A_{ij}$ . The result of evaluating the partial differential for each feature is then pooled globally by dividing each element of the FA map by the total number of features to find the final FA map (Selvaraju et al., 2017).

#### 2.5.2 LIME

The core idea of LIME is to explain predictions of any classifier faithfully by learning an interpretable model locally around the prediction. LIME achieves this by generating simulated data points around an instance through random perturbation and weighting them as a function of proximity to the original data points, fitting a sparse linear model to the predicted responses from the perturbed points and using the sparse linear model as an explanation model (i.e., weights of features in linear model).

$$\xi \left(x\right)=\underset{g\in G}{\mathrm{argmin}}L\left(f,g,\pi_{x}\right)+\Omega \left(g\right)$$ (5)

$$L\left(f,g,\pi_{x}\right)=\underset{z,z^{\prime }\in Z}{\sum }\pi_{x}\left(z\right){\left(f\left(z\right)-g\left(z^{\prime }\right)\right)}^{2}$$ (6)

FA $\xi \left(x\right)$ of given features x is calculated in LIME by minimising the loss function L and complexity, $\Omega \left(g\right)$ , of the function g (a model from a class of possibly interpretable models). In essence L is a function that measures how unfaithful the function g is at approximating f (the model being explained) in the local space defined by $\pi_{x}$ . Eq. 6 shows how the loss function uses the L2 distance to measure how unfaithful function g is at approximating f , where z is sample from x , z is the set perturbed samples of x with associated labels and $z^{\prime }$ is perturbed sample from set z (Kokhlikyan et al., 2020).

#### 2.5.3 Occlusions

Occlusions is a perturbation-based approach to calculate FA, which involves perturbing the feature space with a rectangular region and evaluating the difference of class score from a given class prediction by the perturbation. FA is then assigned by looking at the feature in the multiple rectangular regions it is in and averaging the multiple class score differences (Ancona et al., 2017). The occlusion FA method was based on an occlusion sensitivity analysis used to validate a DL interpretability method by Zeiler et al. (Zeiler and Fergus, 2014).

## 3 Results

### 3.1 Dataset analysis

In the dataset comprising of 122 LA tissues derived from MRI data, the PVI strategy led to successful AF termination in only 11.6% of cases, while 88.4% resulted in failed terminations. Meanwhile, the FIBRO and ROTOR strategies resulted in 42.6% and 74.4% successful terminations, respectively. Notably, FIBRO demonstrated the most balanced AF termination outcomes, whereas ROTOR and PVI exhibited a similar level of misbalance in the outcomes. In the larger dataset consisting of 321 LA tissues, including both MRI-derived and synthetic data, the PVI strategy achieved successful AF termination in 27.1% of cases, demonstration a positive impact of augmentation. The FIBRO and ROTOR strategies also resulted in 58.3% and 75.7% successful terminations, respectively.

### 3.2 Convolutional neural network performance

The success of the FIBRO ablation strategy was predicted most accurately by the CNN, as shown in Table 1, where the FIBRO class has the highest AUC score and the most balanced recall and precision scores. Furthermore, the FIBRO strategy also had the highest AUC score when predicting ablation success exclusively on the real data (Table 2). PVI had the second-highest AUC score on mixed real and synthetic data, as well as exclusively on real data. Meanwhile, ROTOR had a comparable AUC score to PVI on the real and synthetic data but performed worse exclusively on the MRI-derived data (Table 2).

**TABLE 1:** Mean lesion percentage, NAT percentage, IoU of the informative region and ablation lesions with errors (standard deviation) for each FA map method and RFCA strategy.

Ablation strategy	Method	Lesion percentage	IoU	NAT percentage
PVI	LIME	0.44 ± 0.24	**0.077 ± 0.023**	**0.32 ± 0.24**
	Occlusions	**0.55 ± 0.15**	0.065 ± 0.17	0.57 ± 0.15
	GradCAM	0.47 ± 0.17	0.063 ± 0.029	0.60 ± 0.12
FIBRO	LIME	0.57 ± 0.19	0.18 ± 0.09	0.47 ± 0.27
	Occlusions	0.45 ± 0.14	0.19 ± 0.11	0.38 ± 0.20
	GradCAM	**0.62 ± 0.25**	**0.26 ± 0.11**	**0.27 ± 0.16**
ROTOR	LIME	0.62 ± 0.16	0.12 ± 0.07	0.63 ± 0.25
	Occlusions	0.53 ± 0.16	0.14 ± 0.06	0.36 ± 0.16
	GradCAM	**0.71 ± 0.13**	**0.20 ± 0.08**	**0.25 ± 0.06**

**TABLE 2:** Mean AUC score on independent hold-out test set (with standard deviation) for each RFCA strategy and type of data.

Ablation strategy	MRI derived data	MRI derived + synthetic data
**PVI**	0.67 ± 0.03	0.78 ± 0.04
**FIBRO**	0.85 ± 0.02	0.92 ± 0.02
**ROTOR**	0.62 ± 0.05	0.77 ± 0.02

However, the CNN struggled to predict successful AF termination cases by PVI, which is reflected in the low recall and F1 score in Table 3. Even though there was a similar class imbalance in ROTOR compared to PVI, the CNN was able to predict the successful and failed AF termination cases to a reasonable degree (see recall and F1 score in Table 3). Lastly, the CNN had a significantly higher AUC score (*p* < 0.05) when trained and predicted on a dataset comprised of synthetic and MRI derived data compared to training exclusively on MRI derived data (Table 4). This was confirmed using a one-sided *t*-test (PVI: *p* = 0.030; FIBRO: *p* = 3.5e-05; ROTOR: *p* = 6.15e-06). This was due to the increased dataset size when combining the real and synthetic data as the CNN has more training examples–effectively improving the task’s generalisation. Notably, incorporating synthetic data has improved accuracy in predicting the outcomes of PVI. When trained exclusively on MRI-derived data, the model showed a zero F1-score for PVI, attributed to significant class imbalance. This resulted in the model predicting unsuccessful AF termination for all PVI cases, explaining the precision score of 1.0. However, integrating synthetic data into the dataset improved the model’s ability to classify successful ablation for PVI (F1 score of 0.42 ± 0.06), due to the 15.5% increase in successful PVI cases in the dataset. This allowed the model to improve its classification of successful AF termination by PVI.

**TABLE 3:** Mean area under the receiver operating characteristic curve (AUC) score, recall, precision and F1-score on independent hold-out test set (with standard deviation) for each RFCA strategy.

Ablation strategy	AUC	Recall	Precision	F1-score
**PVI**	0.78 ± 0.03	0.35 ± 0.07	0.68 ± 0.28	0.42 ± 0.06
**FIBRO**	0.92 ± 0.02	0.89 ± 0.03	0.82 ± 0.02	0.85 ± 0.01
**ROTOR**	0.77 ± 0.02	0.93 ± 0.04	0.76 ± 0.02	0.84 ± 0.01

**TABLE 4:** Mean AUC, recall, precision and F1 score (with standard deviation) of DL model trained with real data only and with synthetic and real data from a leave-one-out cross-validation on a hold-out test (∼20% of the respective dataset).

Ablation strategy	MRI derived data				MRI derived + synthetic data			
	AUC	Recall	Precision	F1 score	AUC	Recall	Precision	F1 score
**PVI**	0.67 ± 0.03	0	1.0	0	0.78 ± 0.03	0.35 ± 0.07	0.68 ± 0.28	0.42 ± 0.06
**FIBRO**	0.85 ± 0.02	0.75 ± 0.08	0.70 ± 0.03	0.72 ± 0.04	0.92 ± 0.02	0.89 ± 0.03	0.82 ± 0.02	0.85 ± 0.01
**ROTOR**	0.62 ± 0.05	0.99 ± 0.02	0.64 ± 0.01	0.78 ± 0.02	0.77 ± 0.02	0.93 ± 0.04	0.76 ± 0.02	0.84 ± 0.01

Bold numbers signify the highest score from each FA method for each metric.

### 3.3 Qualitative interpretability analysis

**FIGURE 3:**
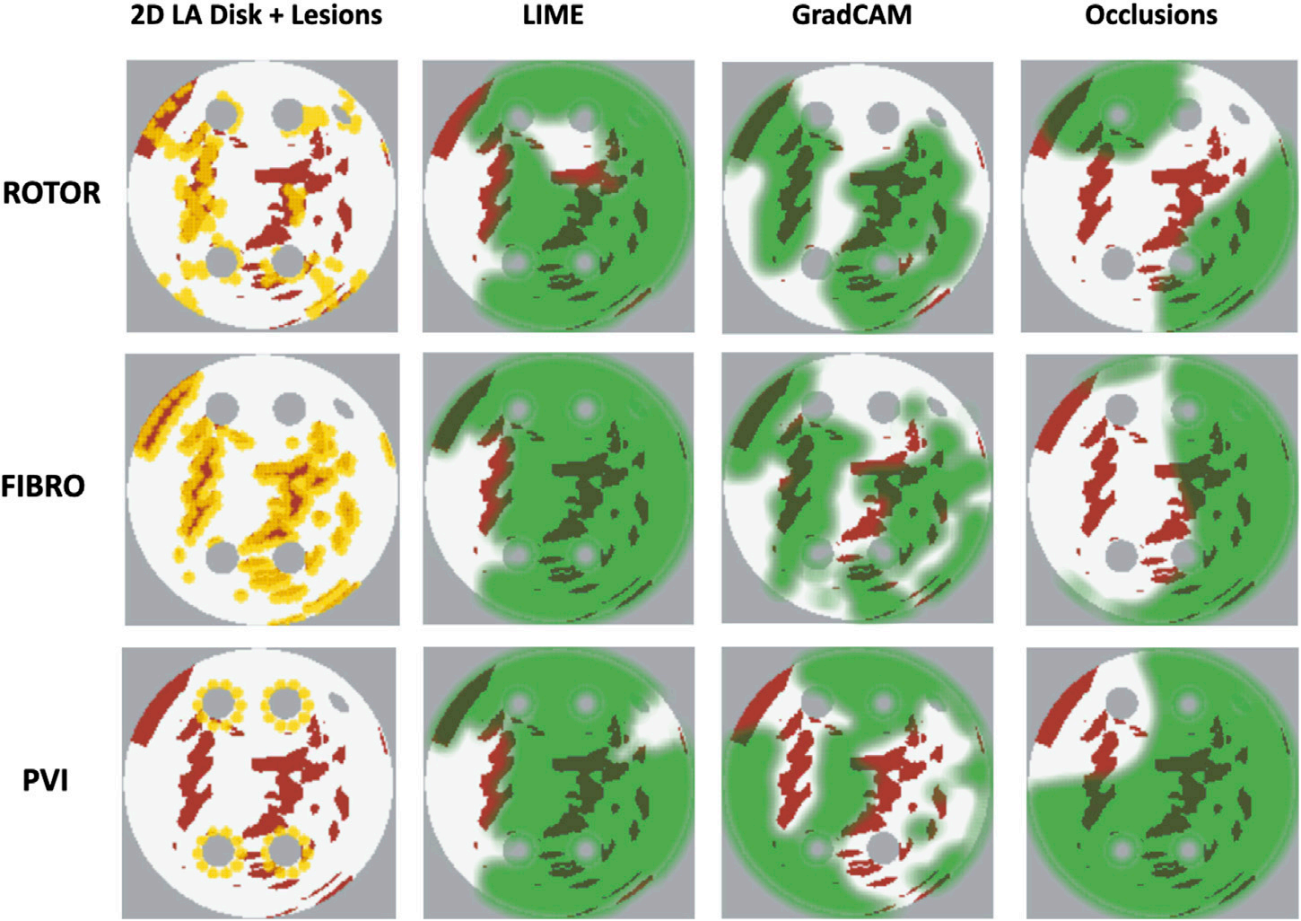
Diagram of 2D LA tissues with highlighted feature attribution maps. White areas in the 2D tissues are healthy tissue and red areas are fibrosis. Ablation lesion locations known from simulations are shown (yellow) for all three RFCA strategies, along with respective FA maps for LIME, GradCAM and occlusions and highlighted thresholded informative regions (translucent green). Same colour scheme in used in Figures 7, 8 below.

As shown in Table 1, GradCAM was characterised by the highest lesion percentage and IoU metrics for the FIBRO and ROTOR strategies. Additionally, Figure 3 shows that in FA maps obtained with GradCAM for ROTOR and FIBRO, the informative regions coincided with most ablation lesions. Figure 3 also illustrates that GradCAM had the lowest NAT percentage for the FIBRO and ROTOR strategies, as the FA maps did not highlight large, but clinically irrelevant regions of healthy tissue–whereas LIME and occlusions did. For the PVI strategy, the occlusions method provided FA maps with the greatest lesion percentage, and LIME provided FA maps with the highest IoU score.

### 3.4 Quantitative interpretability analysis

Using the Wilcoxon signed-rank test, the ROTOR strategy lesion percentage for GradCAM was significantly greater (*p* < 0.017 using Bonferroni correction) than that for occlusions, but not for LIME (*p* = 3.1e-8 and *p* = 0.0253, respectively). Moreover, for the FIBRO strategy, the lesion percentage for GradCAM was significantly higher than that for the occlusions method, but again not for LIME (*p* = 4.0 e-6, *p* = 0.06, respectively). However, the IoU scores for GradCAM were significantly greater (*p* < 0.017) than those for occlusions and LIME for ROTOR (*p* = 3.3e-6 and *p* = 2.1e-9, respectively) and FIBRO (*p* = 4.2e-6 and *p* = 1.6e-9, respectively). GradCAM also had a significantly less NAT percentage (*p* < 0.017) than occlusions and LIME for ROTOR (*p* = 5.5e-05 and *p* = 2.3e-09, respectively) and FIBRO (*p* = 1.2 e-5 and 2.3e-6, respectively).

**FIGURE 4:**
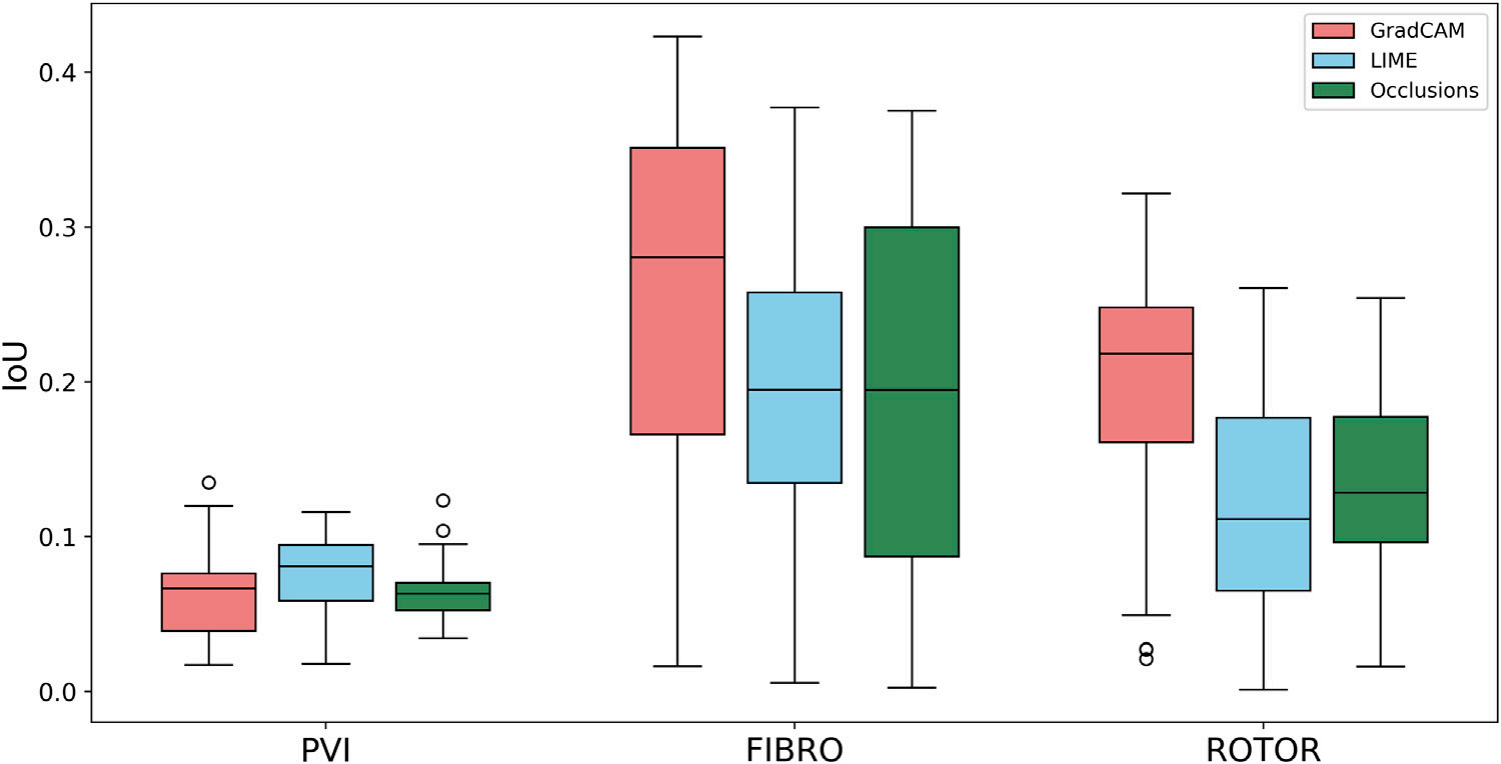
Boxplot of Jacquard index (IoU) for each FA method (GradCAM, LIME and Occlusions) and RFCA strategy (PVI, FIBRO and ROTOR) on the hold-out test set.

**FIGURE 5:**
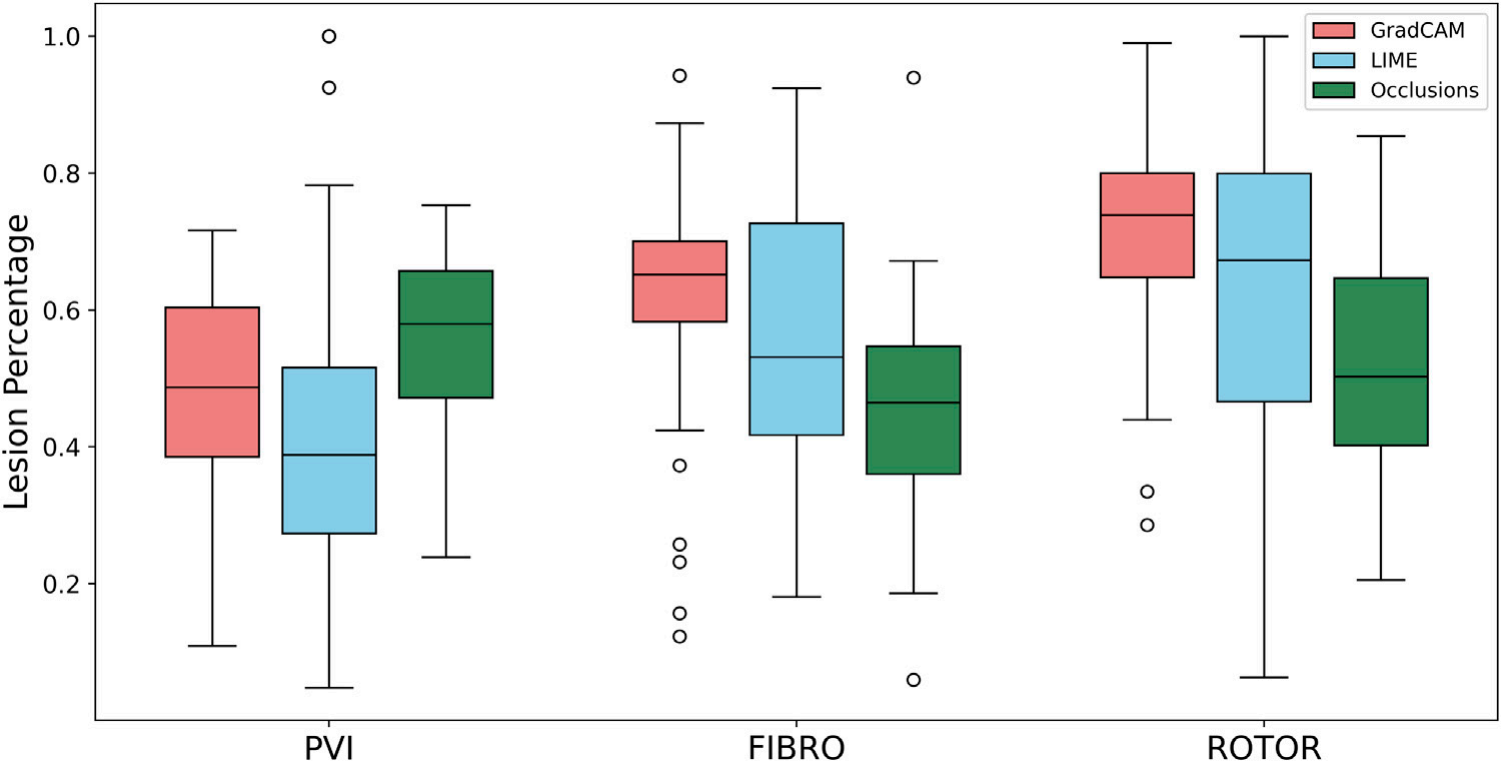
Boxplot of lesion percentage for each FA method (GradCAM, LIME and Occlusions) and RFCA strategy (PVI, FIBRO and ROTOR) on the hold-out test set.

**FIGURE 6:**
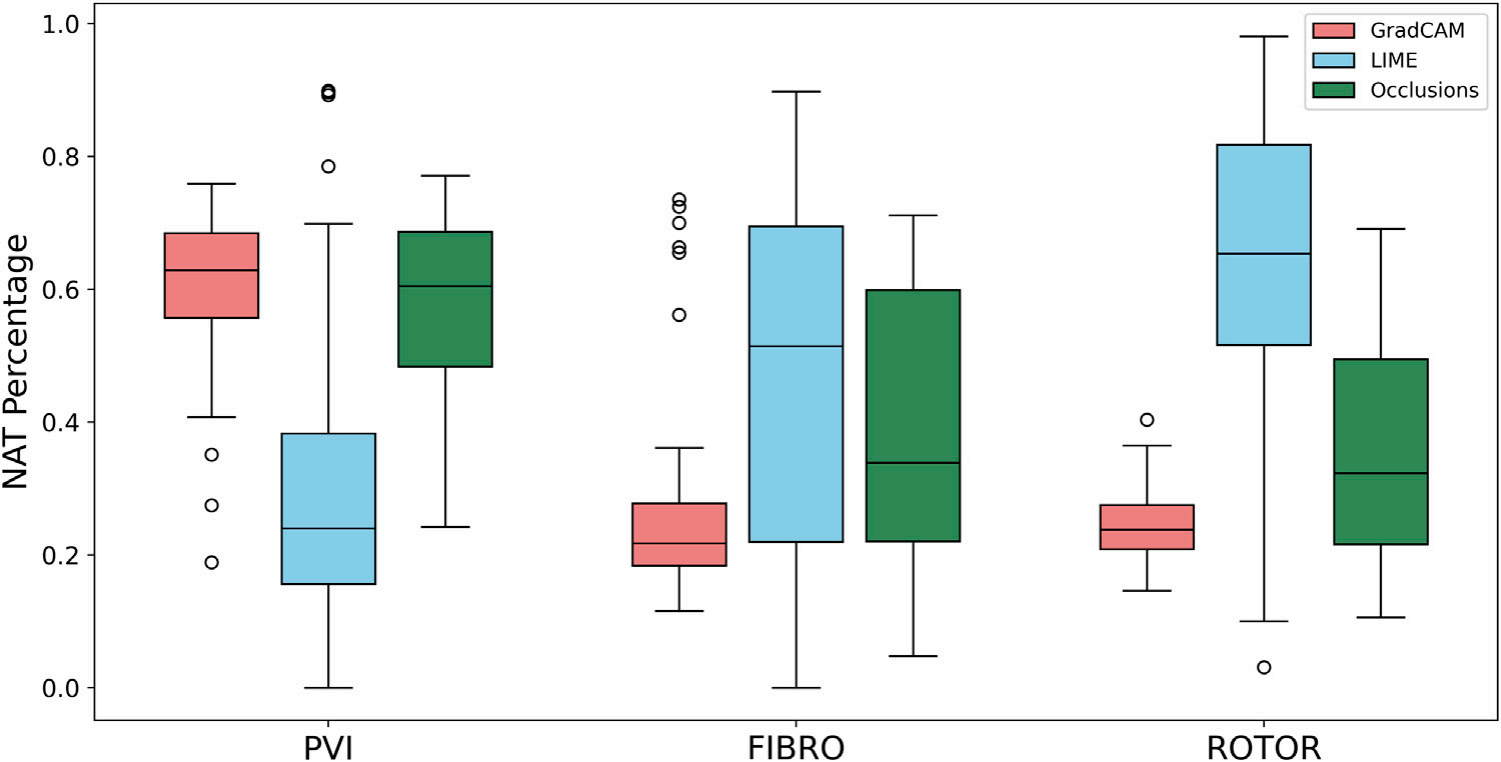
Boxplot of NAT percentage for each FA method (GradCAM, LIME and Occlusions) and RFCA strategy (PVI, FIBRO and ROTOR) on the hold-out test set.

Therefore, GradCAM produced more interpretable FA maps than LIME (for FIBRO and ROTOR) as the informative regions were more focused on areas with a high number of ablation lesions–reflected in GradCAM having a significantly greater IoU score than LIME (Figures 4, 5). Furthermore, GradCAM was also more interpretable in a sense that its FA maps highlighted less regions that were non-arrhythmogenic, and hence it had a significantly less NAT percentage than LIME and occlusions (Figure 6).

For the PVI strategy, the occlusions method provided FA maps with the greatest lesion percentage and LIME FA maps had the highest IoU score. The difference in best FA map methods in terms of lesion percentage and IoU score can be seen in Table 1, as informative regions in the occlusions’ FA maps cover a vast area highlighting the ablation lesions but are not local to the PVs. Meanwhile, the LIME FA map highlights areas around the PVs, but does not cover many ablation lesions.

Supplementary Figure S3, S4, S5 show the difference in the mean score of each interpretability metric for correct and incorrect classifications of AF termination for each ablation strategy and FA method on the hold-out test set. This analysis shows no clear or consistent relationship between interpretability and model accuracy. The mean interpretability scores reflect this, as they were similar across the correct and incorrect classification groups. Additionally, the mean interpretability score variability is inconsistent across each ablation strategy FA method and interpretability metric - further illustrating no relationship between interpretability and accuracy.

### 3.5 Feature attribution thresholding sensitivity analysis

**FIGURE 7:**
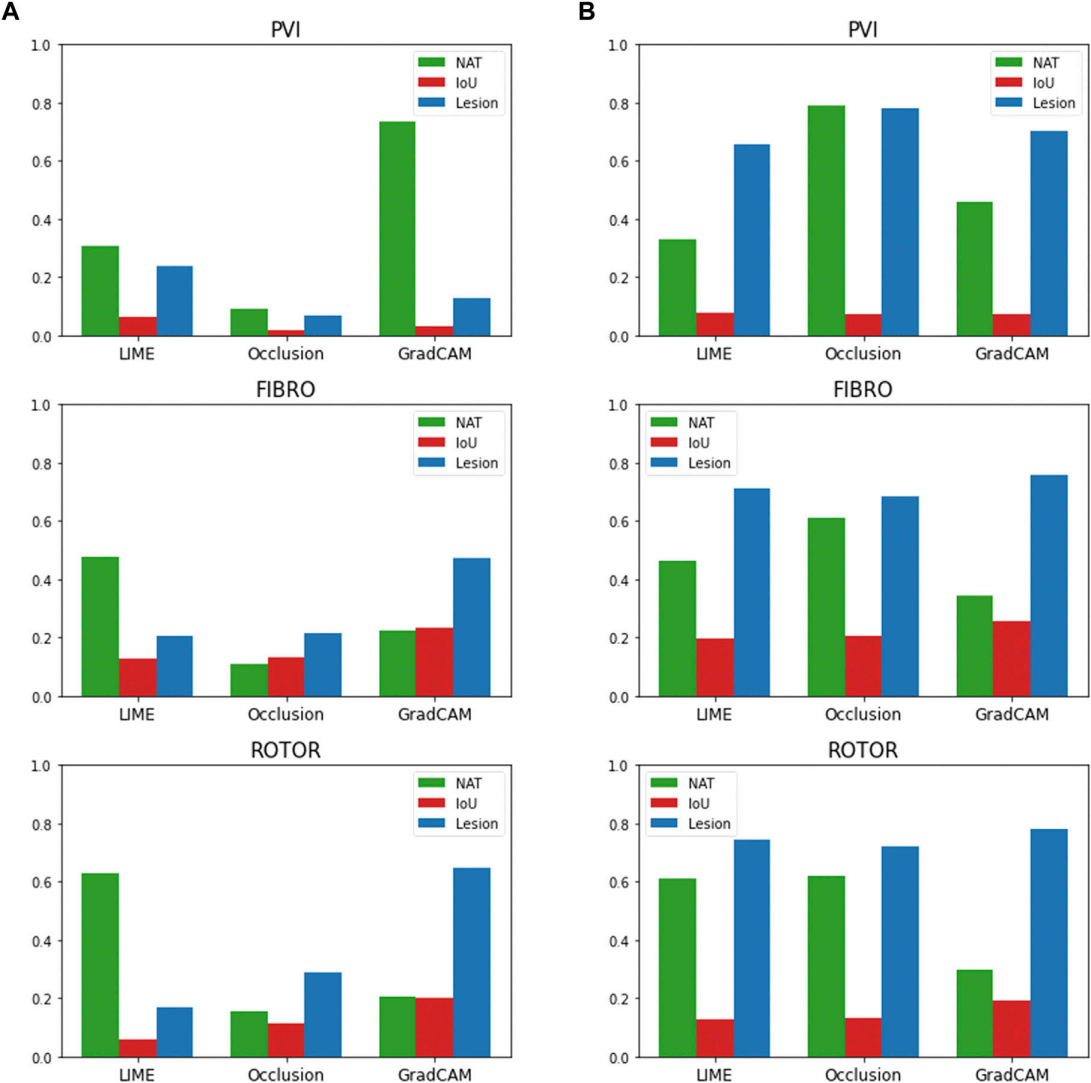
IoU, lesion and NAT percentage values for each interpretability method and ablation strategy with altered informative region threshold value. **(A)**. Informative region threshold value 25% above the average FA. **(B)**. Informative region threshold value 25% below the average FA.

The findings presented above show little dependence on the threshold between informative and uninformative regions. As shown in Figure 7, when the threshold value is set to 25% above and below the average feature attribution, Grad-CAM still has the highest lesion percentage and IoU compared to LIME and Occlusions for the ROTOR and FIBRO strategies. GradCAM still had a lower NAT percentage for FIBRO and ROTOR when the threshold value was 25% below the average FA. However, occlusions had a lower NAT percentage for FIBRO and ROTOR when the threshold value was above 25% of the average FA. Occlusions had a lower lesion percentage and IoU, which shows that GradCAM was more interpretable when the threshold was 25% above the average FA.

### 3.6 Population-level interpretability analysis

**FIGURE 8:**
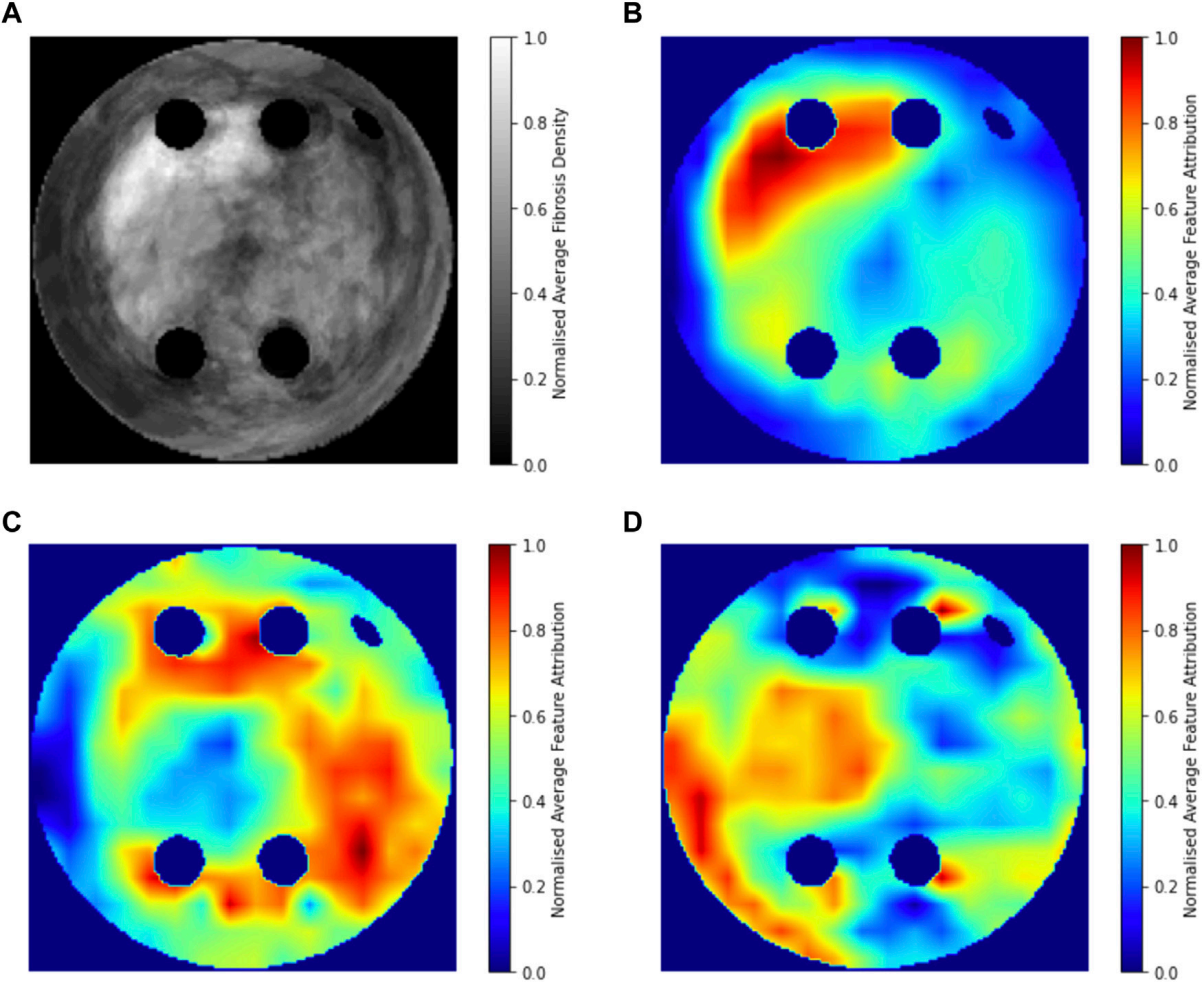
Averaged LGE MRI intensities and FA maps on the hold-out test set. **(A)**. Averaged and normalised LGE MRI intensity in the LA tissue disks. **(B)**. Averaged and normalised GradCAM FA map for the ROTOR ablation strategy. **(C)**. Averaged and normalised GradCAM FA map for the FIBRO ablation strategy. **(D)**. Averaged and normalised GradCAM FA map for the PVI ablation strategy.

**FIGURE 9:**
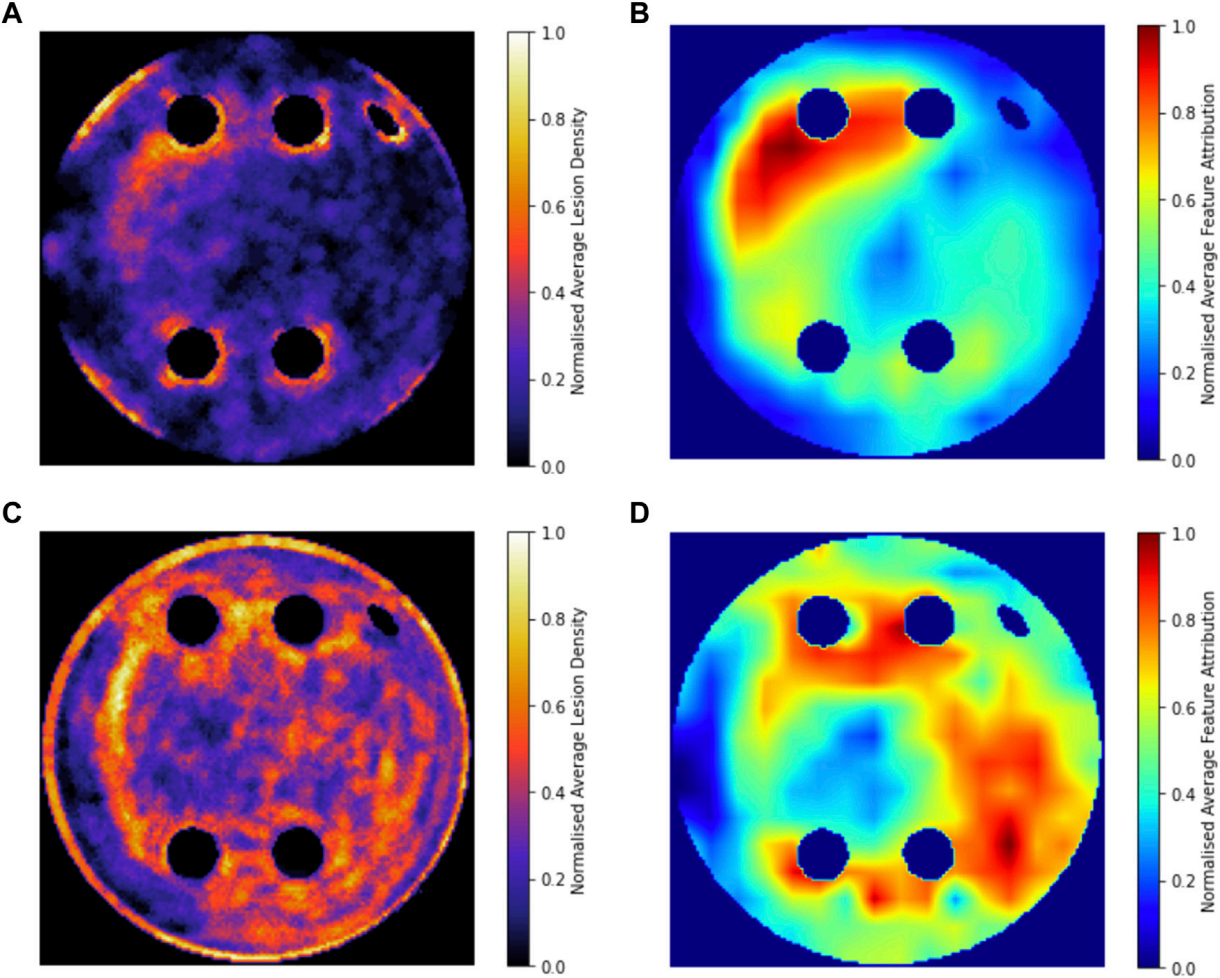
Averaged and normalised ablation lesions and GradCAM FA maps for FIBRO and ROTOR on the hold-out test set. **(A)**. Ablation lesions for ROTOR. **(B)**. FA map for ROTOR. **(C)**. Ablation lesions for FIBRO. **(D)**. FA map for FIBRO.

Figure 8 compares the average GradCAM FA maps for ROTOR, FIBRO and PVI with the average fibrosis density across the 2D LA tissue disks. It shows that the high FA regions in the average FA map for ROTOR (Figure 8B) and FIBRO (Figure 8C) correspond with dense fibrotic areas (Figure 8A). Furthermore, there was a similar good correspondence between the average GradCAM FA maps for ROTOR and FIBRO (Figure 9B,D) and the respective average lesions across the 2D LA tissue disks (Figure 9A,C). Unsurprisingly, the average GradCAM FA map for PVI (Figure 8D) showed relatively small correspondence to areas with high fibrosis density areas.

## 4 Discussion and conclusion

Predicting RFCA outcomes from imaging data is a challenging task, as shown by Kim et al., who predicted AF recurrence post-RFCA with a 0.61 accuracy from a CNN which used a combination of MRI data and patient demographics (Kim et al., 2020). Moreover, Roney et al. applied machine learning to predict *in silico* AF recurrence after multiple ablation strategies (Roney et al., 2018; Roney et al., 2020).

Therefore, developing a successful DL model to predict RFCA outcomes in AF simulations is the natural first step to predict real RFCA outcomes in AF patients. Hence, this study i) demonstrates a multi-label classification CNN for the success of ablation strategies in patient-specific simulations of AF, with AUC scores of 0.92 ± 0.02 for FIBRO, 0.78 ± 0.04 for PVI and 0.77 ± 0.02 for ROTOR, and iii) explores different methods of DL interpretability in the classification, with GradCAM shown to provide the most interpretable FA maps for the ROTOR and FIBRO strategy, suggesting that the DL model utilises pro-arrhythmogenic regions to make its prediction. This is further supported by the population-level interpretability analysis, as average FA maps for ROTOR and FIBRO are focused on areas with high fibrotic density. This can be explained by the fact that the respective ablation lesions are primarily located within these areas. Hence, the DL model can learn to predict AF termination outcomes by implicitly leveraging pro-arrhythmogenic regions related to a given strategy. Importantly, locations of the ablation lesions have not been explicitly used in the CNN’s learning process.

It is worth noting that classification of the PVI strategy was difficult to interpret. A possible reason for this difficulty is that the PVI strategy in the clinic is based on ablating PV triggers that typically initiate AF. However, these initial PV triggers were not present in the 2D LA tissue models. Therefore, the three FA methods could not produce interpretable maps in this case.

A possible explanation for why GradCAM performed better than the other methods is that LIME is susceptible to unstable generated interpretations due to random perturbations and feature selection. Moreover, LIME and occlusions are not class discriminative–meaning that they cannot localise the class (RFCA strategy) within the feature space. GradCAM is gradient-based (does not randomise parameters to obtain FA maps) and is class discriminative, allowing it to localise pro-arrhythmogenic regions more faithfully than LIME and occlusions (Selvaraju et al., 2017; Zafar and Khan, 2021).

The RFCA strategy that has the highest magnitude of lesion percentage and lowest magnitude of NAT percentage (ROTOR) also had the lowest AUC score in testing (Table 3), showing that the interpretability of a FA map does not increase with the accuracy of the strategy’s prediction. This observation demonstrates that the need for interpretability in RFCA strategy prediction likely goes beyond FA, and in future work, we will investigate the incorporation of confidence in prediction outputs to enable our method to be used as a decision support tool to help clinicians select the appropriate therapy. Since Varela et al. showed that LA anatomy is a significant factor in prediction of AF recurrence post ablation (Varela et al., 2017a), the DL approach of the study should be extended to 3D LA images and simulations. Future work should also focus on using exclusively real patient LA data and investigating intrinsically interpretable DL models such as ICAM (Bass et al., 2022).

Note that 2D LA disks were used in this study due to the efficiency in providing the needed proof of concept and had clear advantages over extremely computationally-intensive 3D atrial simulations. Moreover, the standardised 2D unfolded LA images allowed for generation of a large number of additional synthetic images, which is crucial for training CNNs. Hence, image-based 2D LA models provided a sensible balance between realistic details (such as fibrosis distributions) and computational efficiency (i.e., the ability to run a large number of simulations and train the CNN). Previous work has shown that atrial wall thickness is distributed more or less evenly in the LA outside of PVs and that slow conduction in fibrotic areas is the main determinant of the rotor dynamics (Varela et al., 2017b; Roy et al., 2018).

Another worthwhile direction is applying an approach based on counterfactual explanations, which alters the input’s feature space to change the classifier’s prediction. Mertes et al. has applied this approach to a generative adversarial network and showed its superiority to LIME in an X-ray imaging study of pneumonia (Mertes et al., 2022). This research utilised over 100 non-medical experts for the evaluation, which ultimately should become a standard for any interpretability study.

**FIGURE 10:**
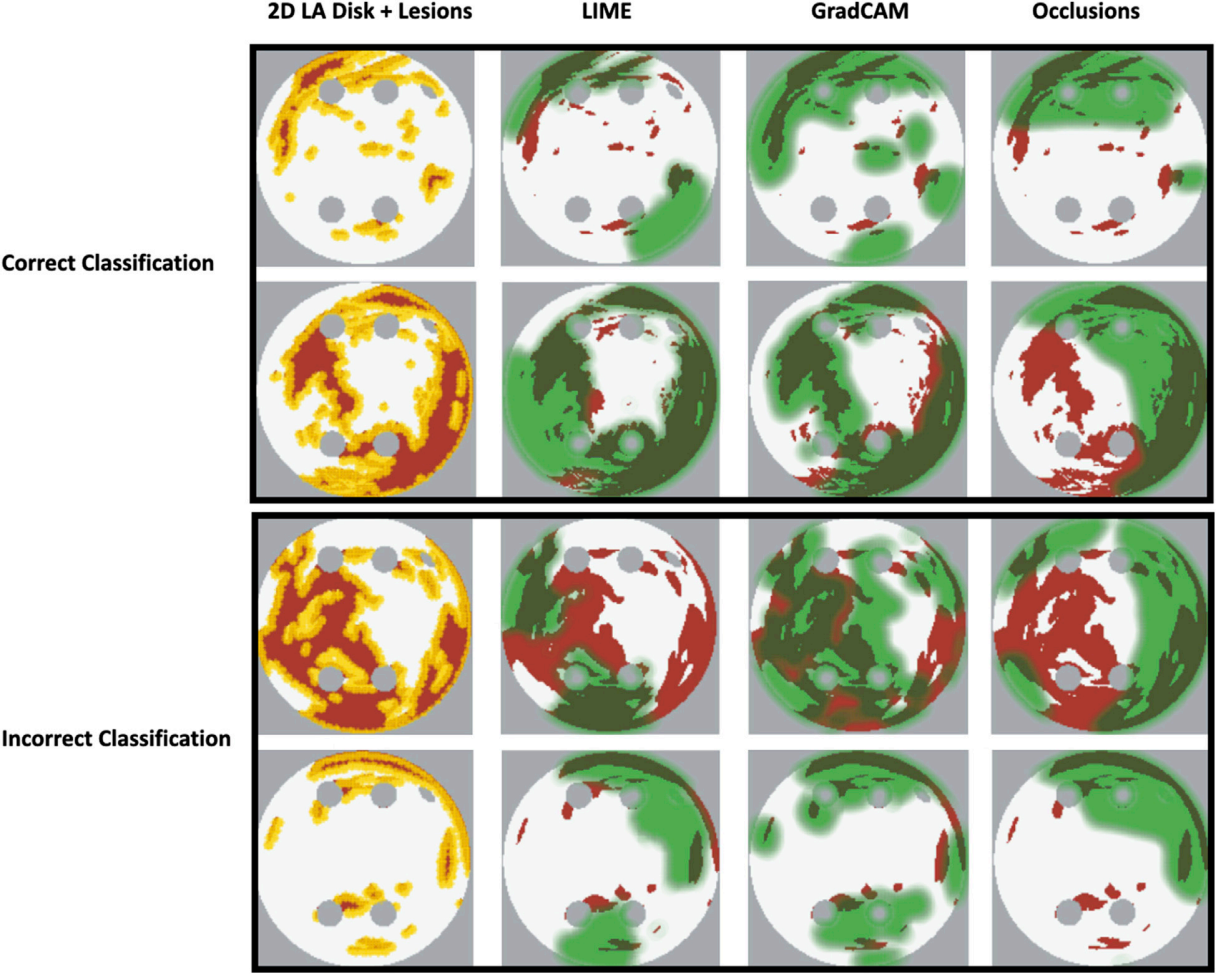
Correct and incorrect classification examples of FA maps (LIME, GradCAM and occlusions) for FIBRO.

**FIGURE 11:**
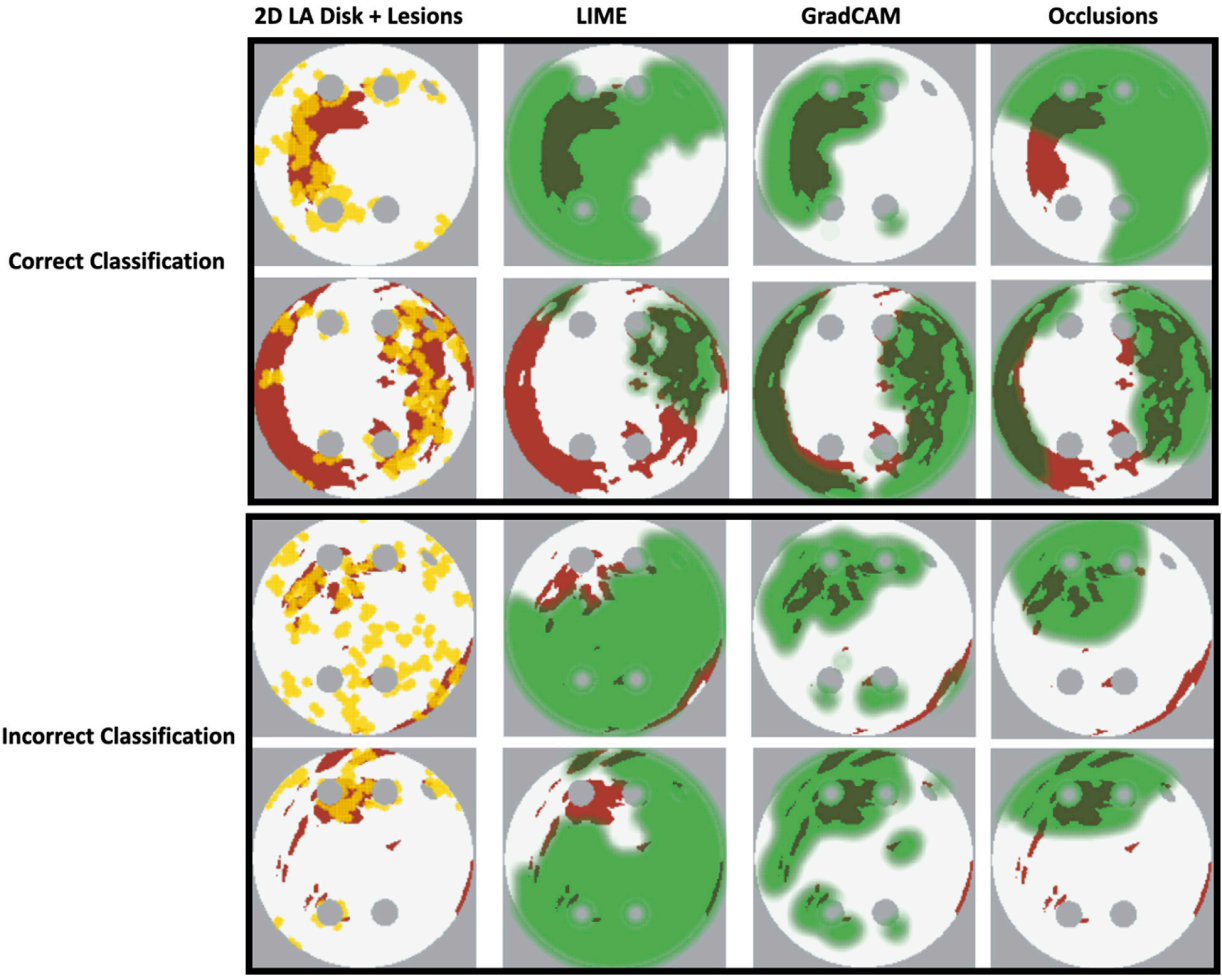
Correct and incorrect classification examples of FA maps (LIME, GradCAM and occlusions) for ROTOR.

Our original approach to the evaluation is based on using a large number of 2D LA tissue models with tractable features (rather than a large number of experts) to understand the predictions of the DL model. Simulations of the test set of 50 2D LA tissue models reveal the important features determining the success of each given RFCA strategy, such as the precise locations of ablation lesions and underlying structural features. This evaluation shows that GradCAM best characterises if a DL model leverages relevant features in its predictions. The fact that GradCAM highlights relevant features and does not highlight healthy tissue devoid of such features is illustrated in Figures 3, Figure 10, Figure 11 and supported by numerical metrics calculated using all 50 LA tissue models and summarised in Table 1.

The EU’s GDPR requires an explanation for any algorithmic decision used in patient care; we believe our work represents a significant step to meet this requirement. Most of the ablation lesions in our study coincided with informative regions of the GradCAM FA maps (specifically, for ROTOR and FIBRO, see Figures 10,11), whereas healthy, non-arrhythmogenic tissue (NAT) was outside of these informative regions. This suggests that the DL model can learn from structural features of patient MR images even without knowledge of the LA function. The explanation is that the structural features constitute pro-arrhythmogenic LA regions (e.g., fibrotic regions are well-known for their ability to harbour rotors sustaining AF) that need to be targeted by ablation. Such mechanistic explanations should increase clinician’s confidence in using the DL predictions in future.

This study’s analysis also suggests that there is no clear relationship between a model’s interpretability and accuracy, which opens future directions of research into the relationship and interaction between a model’s performance and explainability. Another interesting investigation would be into how FA maps can be used as model feedback to improve its performance. To our knowledge, no study has investigated the application of interpretability feedback for DL model design and development for biomedical applications. Bell et al. investigated the trade-off between accuracy and explainability for black box and interpretable models. They showed that the trade-off is inconsistent, and in some cases models with high explainability can also have high accuracy - but in others higher explainability comes at the expense of low accuracy (Bell et al., 2022).

Importantly, the purpose of FA maps is not to be directly applied in the clinic to predict ablation lesions in a patient–but to explain why the DL approach is making a certain prediction, and to increase clinical confidence in this approach (Lipton, 2017). The lesion percentage is a relevant metric as each RFCA lesion is associated with an arrhythmogenic location of the atrial tissue. The lesions are well defined from simulation of 2D LA models in the current study (and known by a clinician when treating a patient)—but the DL model does not learn the locations of the ablation lesions during training. Hence, the ability of the DL model to utilise these (unseen) lesion locations in its predictions of the RFCA strategy from patient MRI provides foundation for the development of interpretable AI. In the future, such AI approaches can provide a clinician with decision support tools that they understand and trust.

## Data Availability

The datasets presented in this study can be found in online repositories. The names of the repository/repositories and accession number(s) can be found in the article/Supplementary Material.
