# Supplementary material for: Exploring interpretability in deep learning prediction of successful ablation therapy for atrial fibrillation
Source: Front Physiol. 2023 Mar 14;14:1054401. doi: 10.3389/fphys.2023.1054401 (PMC10043207; doi:10.3389/fphys.2023.1054401)
Supplement: Supplementary file 1 [file DataSheet2.pdf]

Supplementary Material

Exploring Interpretability in Deep Learning Prediction of Successful Ablation Therapy for Atrial Fibrillation

Shaheim Ogbomo-Harmitt, Marica Muffoletto, Aya Zeidan, Ahmed Qureshi, Andrew P. King, Oleg Aslanidi*

*** Correspondence:** Oleg Aslanidi: oleg.aslanidi@kcl.ac.uk

1. Synthetic Tissue Subject Parameter Analysis:

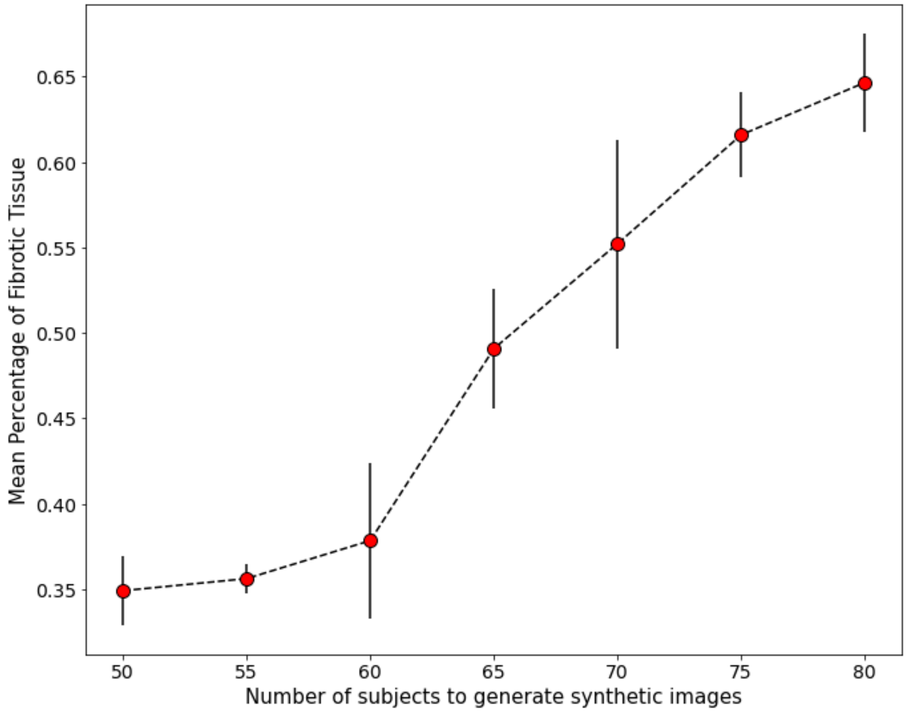

Supplementary Figure S1. The plot of the average percentage of fibrotic tissue with errors from five synthetic LA tissue against the number of subjects used in the synthetic tissue generation.

2. Pre-and-Post Ablation Fibrosis Distribution Comparison:

The pre-and post-ablation fibrosis distributions were vastly different, so they did not need to be accounted for during data splitting; this was validated by evaluating the normalised cross-correlation of the fibrosis distribution between the pre- and post-ablation 2D LA tissue models. The normalised cross-correlation was 0.28 ± 0.11, showing little similarity between the fibrosis distribution pre- and post-ablation. Supplementary Figure S2 also illustrates the minor similarity in fibrosis distribution between pre- and post-ablation LA tissues. In order to compare the fibrosis distributions of different pre/post subjects, we calculated the normalised cross-correlation between each subject's distribution. We then determined the maximum normalised cross-correlation for each pre- and post-ablation subject. Our results showed that the average maximum normalised cross-correlation for pre-ablation subjects was 0.39 ± 0.10, and for post-ablation subjects was 0.38 ± 0.10. Finally, we conducted a t-test to compare the normalised cross-correlation between the post- and pre-fibrosis distribution and the maximum normalised cross-correlation between pre- and post-ablation subjects. Our findings revealed that the normalised cross-correlation between the post- and pre-fibrosis distribution was significantly less than the maximum normalised cross-correlation between pre- and post-ablation subjects ($p = 0.0007$ and $p = 0.002$, respectively). Therefore, our results suggest that it is unnecessary to split pre- and post-ablation subjects during cross-validation based on fibrosis distribution correlation, as higher correlations can be observed between a pre/post-ablation subject and a randomly selected pre/post-ablation fibrosis distribution. Chubb et al. have also reported a weak correlation between thresholded fibrosis before and after ablation (Dice score of 0.032) (Chubb et al., 2018). This can be due to the myocardium developing new fibrosis post-ablation, as well as strong LGE intensity of the ablation scar itself changing the overall intensity distribution (Fukumoto et al., 2015; Kheirkhahan et al., 2020). These findings highlight independence of pre- and post-ablation scar location in LGE MR images.

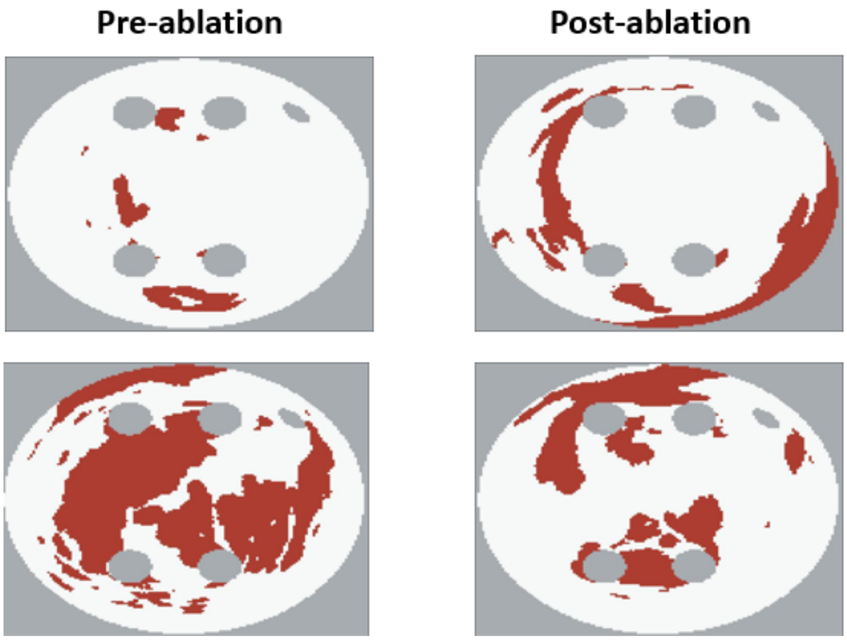

Supplementary Figure S2. Comparison of pre and post ablation fibrosis distribution on 2D LA disks from two representative subjects.

3. Interpretability and Prediction Accuracy Analysis:

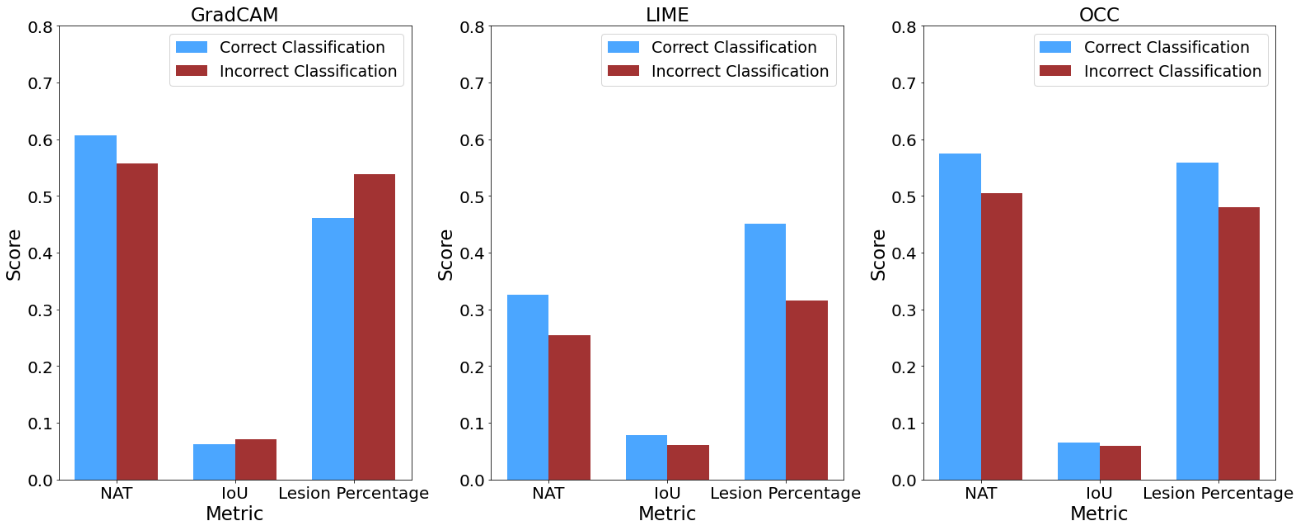

Supplementary Figure S3. Barcharts of the mean score for each interpretability score (NAT, IoU and lesion percentage) and FA method (GradCAM, LIME and occlusions) for incorrect and correct classification of PVI AF Termination on the hold-out test set.

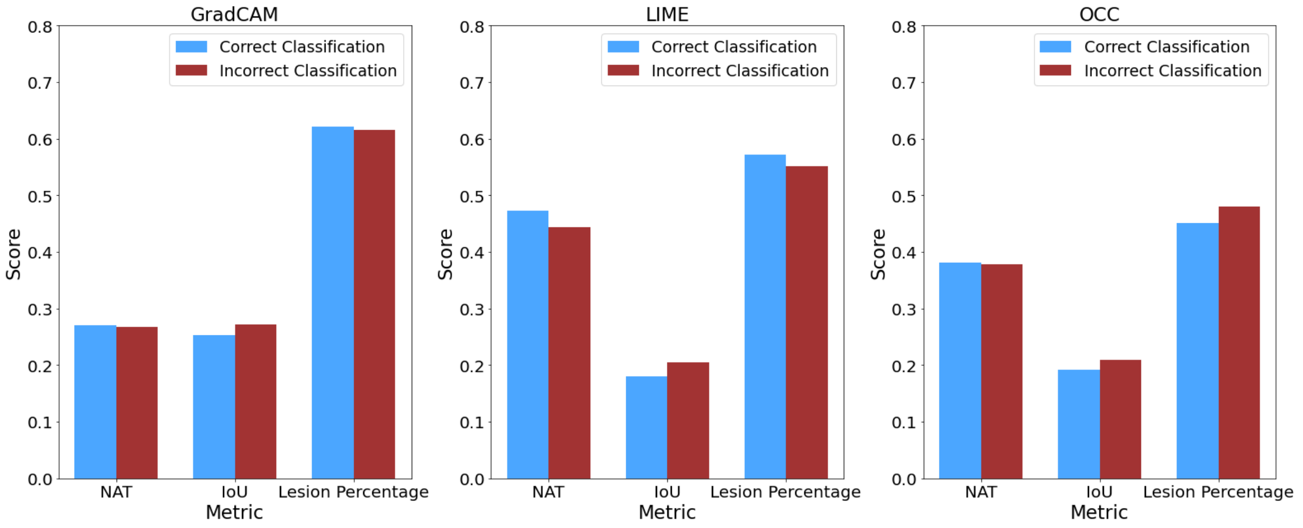

Supplementary Figure S4. Barcharts of the mean score for each interpretability score (NAT, IoU and lesion percentage) and FA method (GradCAM, LIME and occlusions) for incorrect and correct classification of FIBRO AF Termination on the hold-out test set.

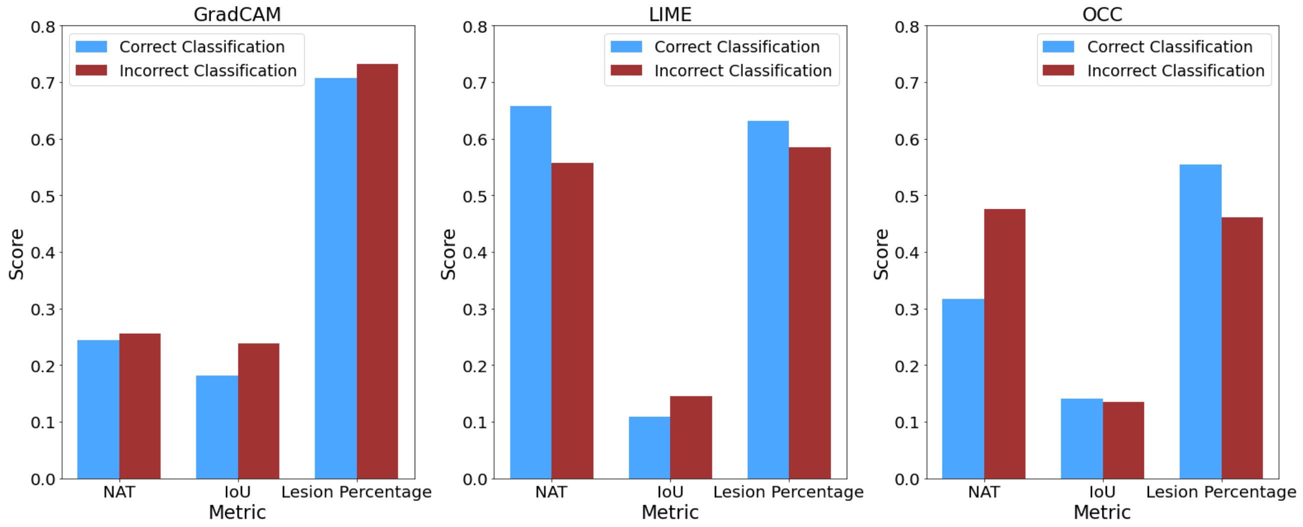

Supplementary Figure S5. Barcharts of the mean score for each interpretability score (NAT, IoU and lesion percentage) and FA method (GradCAM, LIME and occlusions) for incorrect and correct classification of ROTOR AF Termination on the hold-out test set.

4. Code and Data:

Code and data for 'Exploring Interpretability in Deep Learning Prediction of Successful Ablation Therapy for Atrial Fibrillation' study.

Data Sheet 1.zip file contains 321 LA tissue disks (real and synthetic) used for training, validation and testing of DL model.

Code for DL model and generation of FA maps, can be found on GitHub:

https://github.com/ShaheimOH/AF_Interpretability

Dependencies:

NumPy captum lime cv2 skimage Pytorch

Example code:

```
FA_type = 'GradCAM'
model_pth = 'C:/Users/test_user/Desktop/Code/Model.pt'
image_pth = 'C:/Users/test_user/Desktop/Code/real_1R71W.jpg'
strat_index = 0
FA_obj = FA(FA_type,model_pth,image_pth,strat_index)
test = FA_obj.run()
```

5. References

Chubb, H., Karim, R., Roujol, S., Nuñez-Garcia, M., Williams, S. E., Whitaker John and Harrison, J., et al. (2018). The reproducibility of late gadolinium enhancement cardiovascular magnetic resonance imaging of post-ablation atrial scar: a cross-over study. *Journal of Cardiovascular Magnetic Resonance* 20.

Fukumoto, K., Habibi, M., Gucuk Ipek, E., Khurram, I. M., Zimmerman, S. L., Zipunnikov, V., et al. (2015). Comparison of preexisting and ablation-induced late gadolinium enhancement on left atrial magnetic resonance imaging. *Heart Rhythm* 12, 668–672. doi: 10.1016/J.HRTHM.2014.12.021.

Kheirkhahan, M., Baher, A., Goldooz, M., Kholmovski, E. G., Morris, A. K., Csecs, I., et al. (2020). Left atrial fibrosis progression detected by LGE-MRI after ablation of atrial fibrillation. *Pacing Clin Electrophysiol* 43, 402–411. doi: 10.1111/PACE.13866.
