# Supplementary figures and images for: Exploring interpretability in deep learning prediction of successful ablation therapy for atrial fibrillation (part 2 of 2)
Source: Front Physiol. 2023 Mar 14;14:1054401. doi: 10.3389/fphys.2023.1054401 (PMC10043207; doi:10.3389/fphys.2023.1054401)

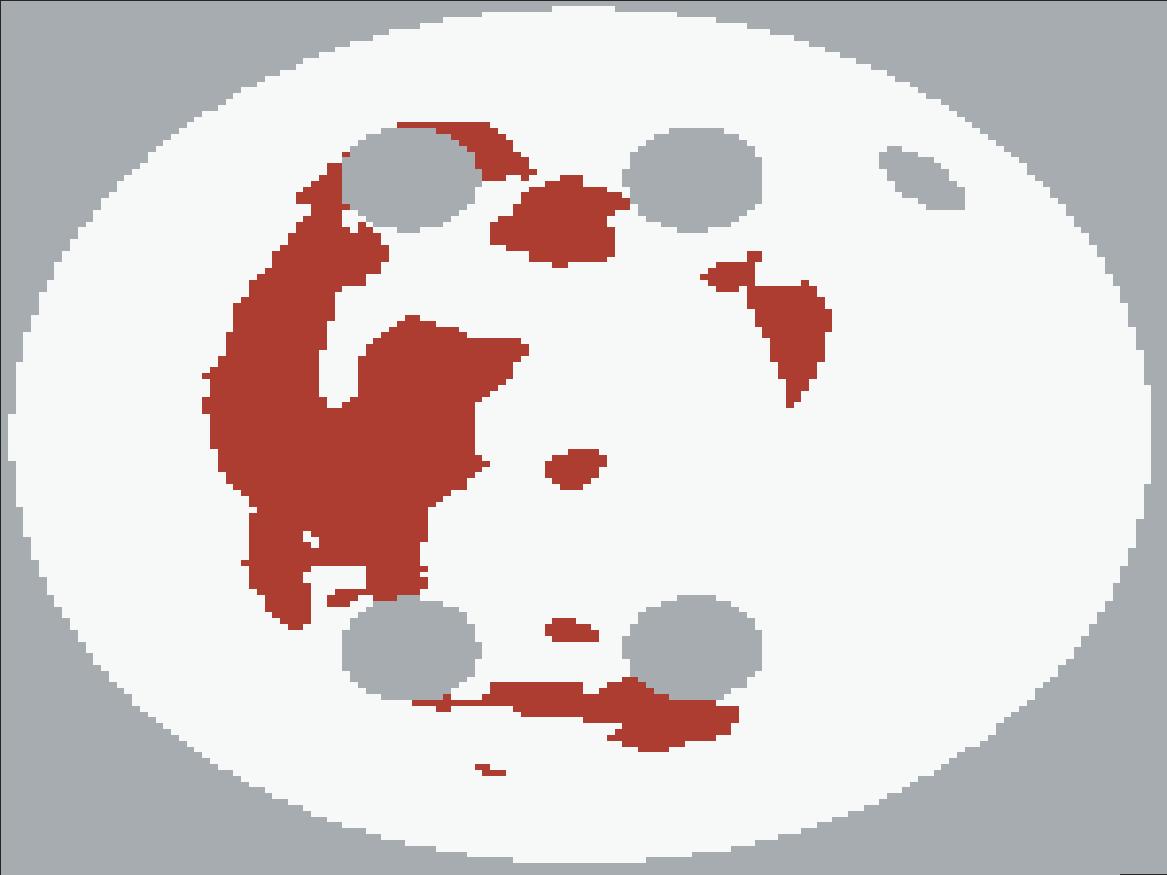

Supplement: Supplementary file 2 [file DataSheet1.ZIP › Dataset/real_SEOHJ.jpg]

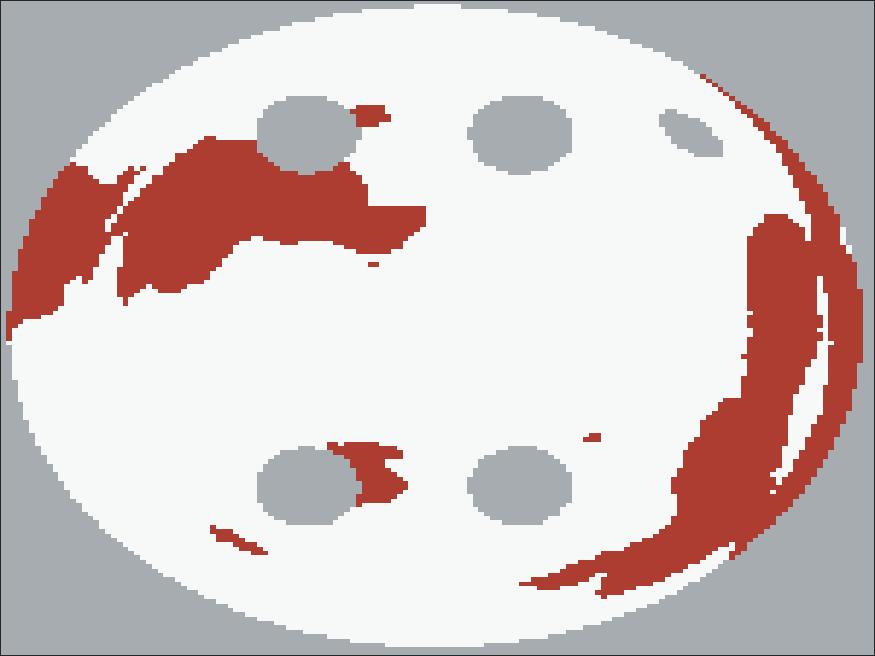

Supplement: Supplementary file 2 [file DataSheet1.ZIP › Dataset/real_SHQXZ.jpg]

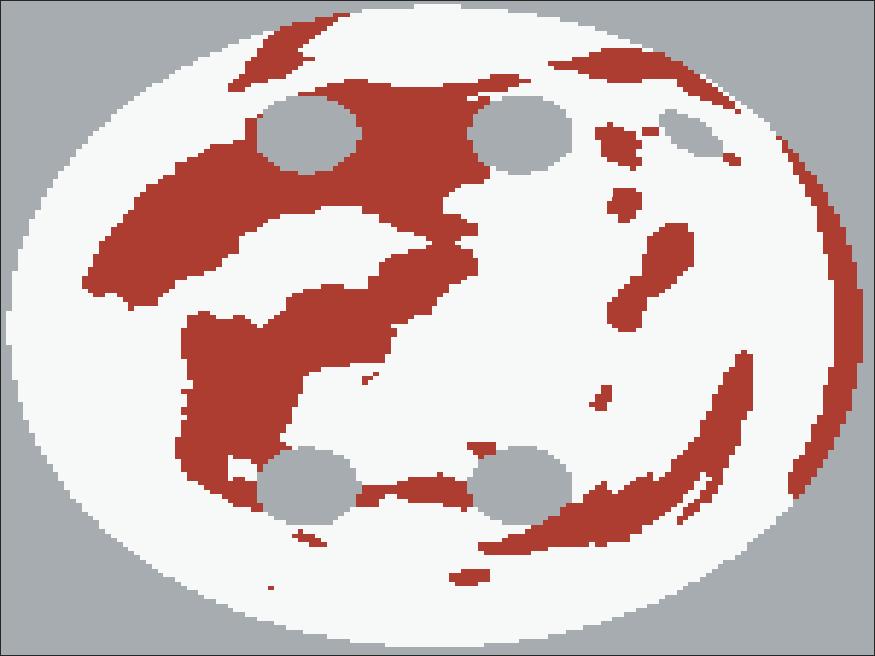

Supplement: Supplementary file 2 [file DataSheet1.ZIP › Dataset/real_SMNP3.jpg]

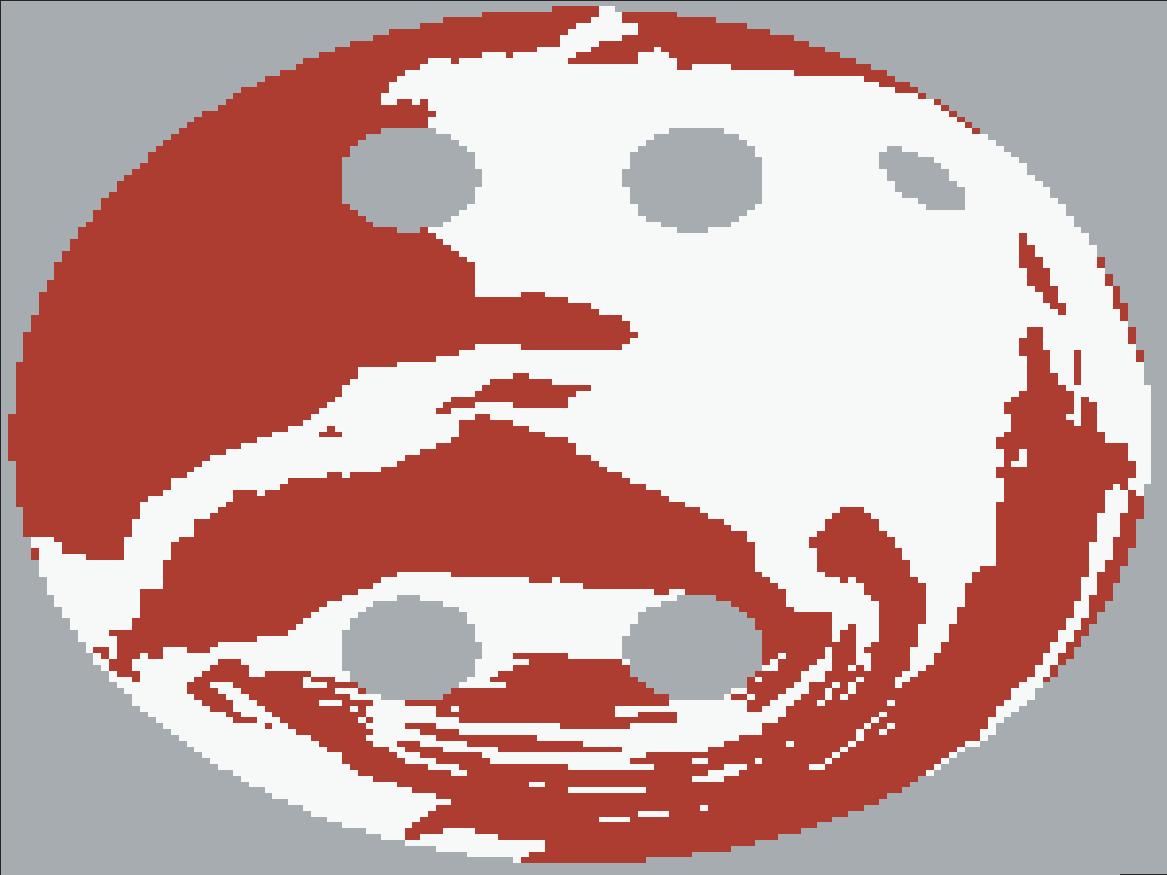

Supplement: Supplementary file 2 [file DataSheet1.ZIP › Dataset/real_SZ47I.jpg]

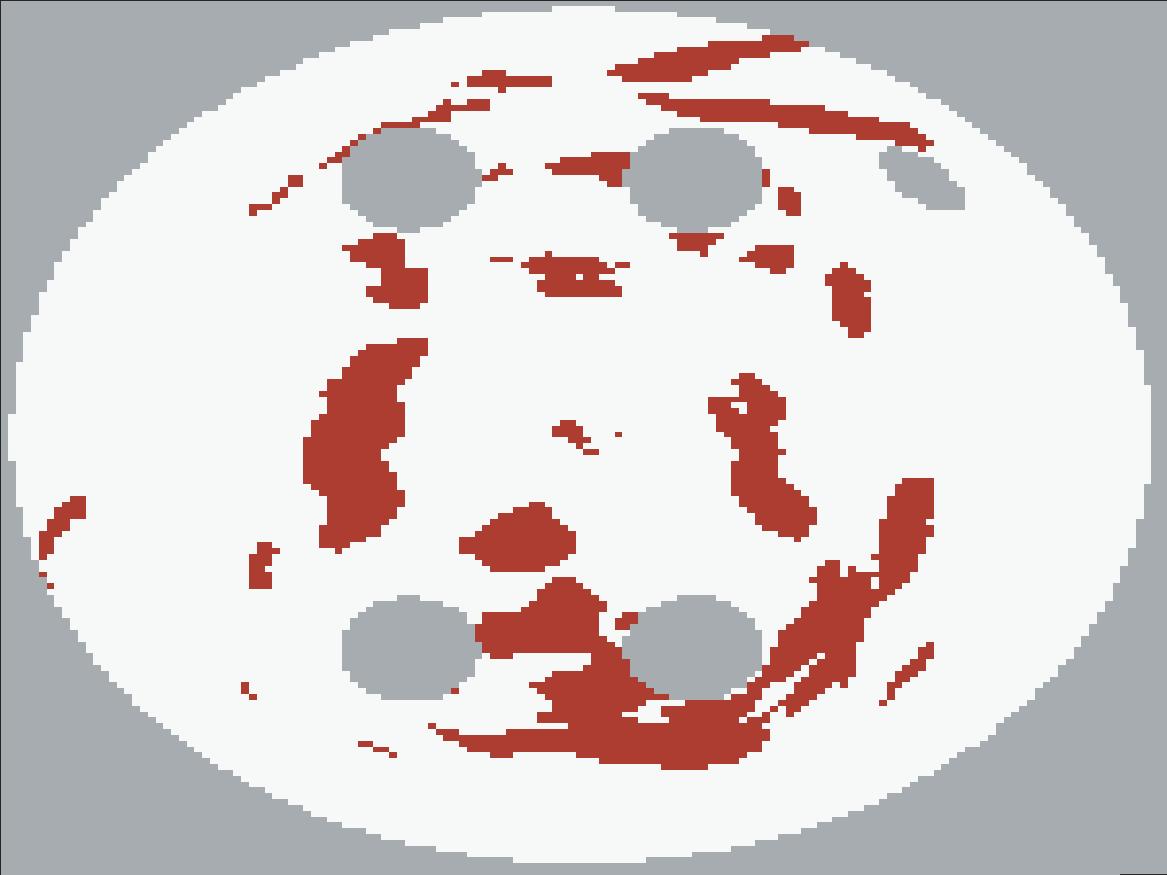

Supplement: Supplementary file 2 [file DataSheet1.ZIP › Dataset/real_UJBAB.jpg]

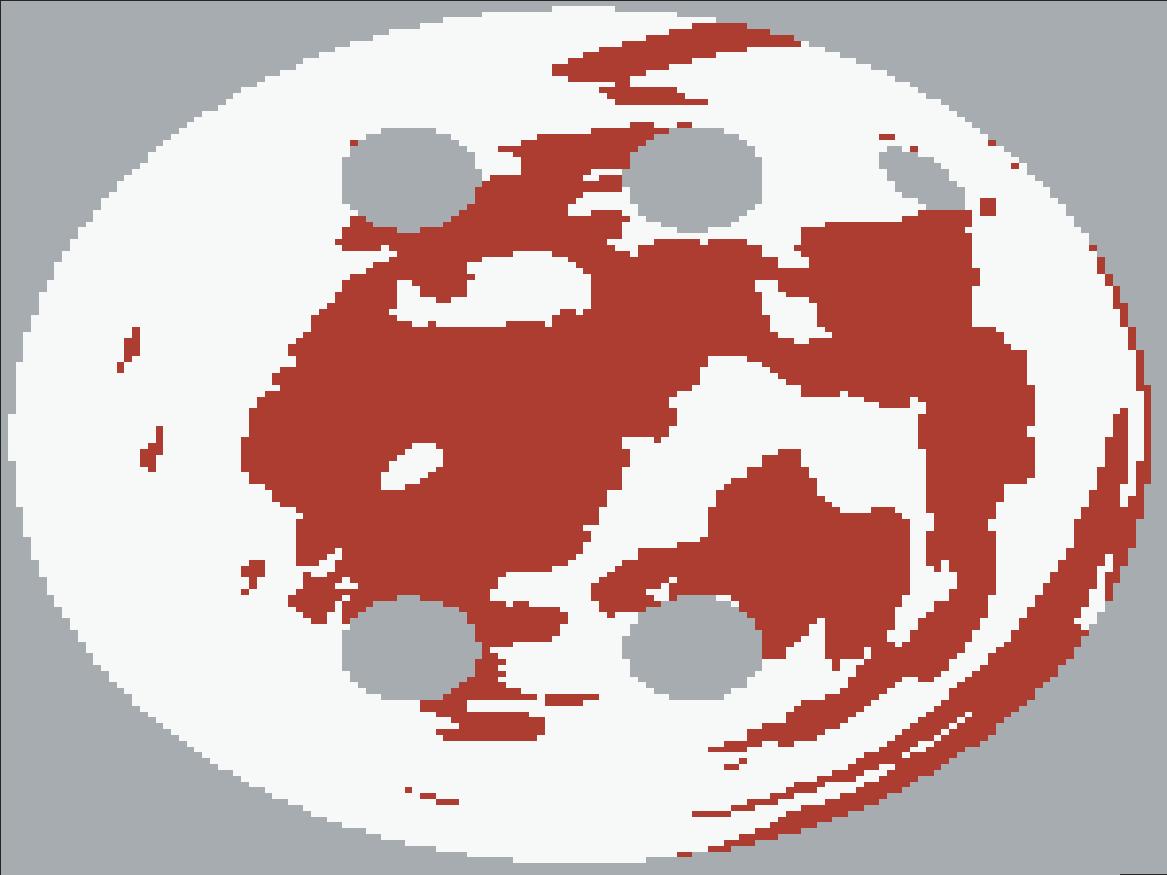

Supplement: Supplementary file 2 [file DataSheet1.ZIP › Dataset/real_UXJWB.jpg]

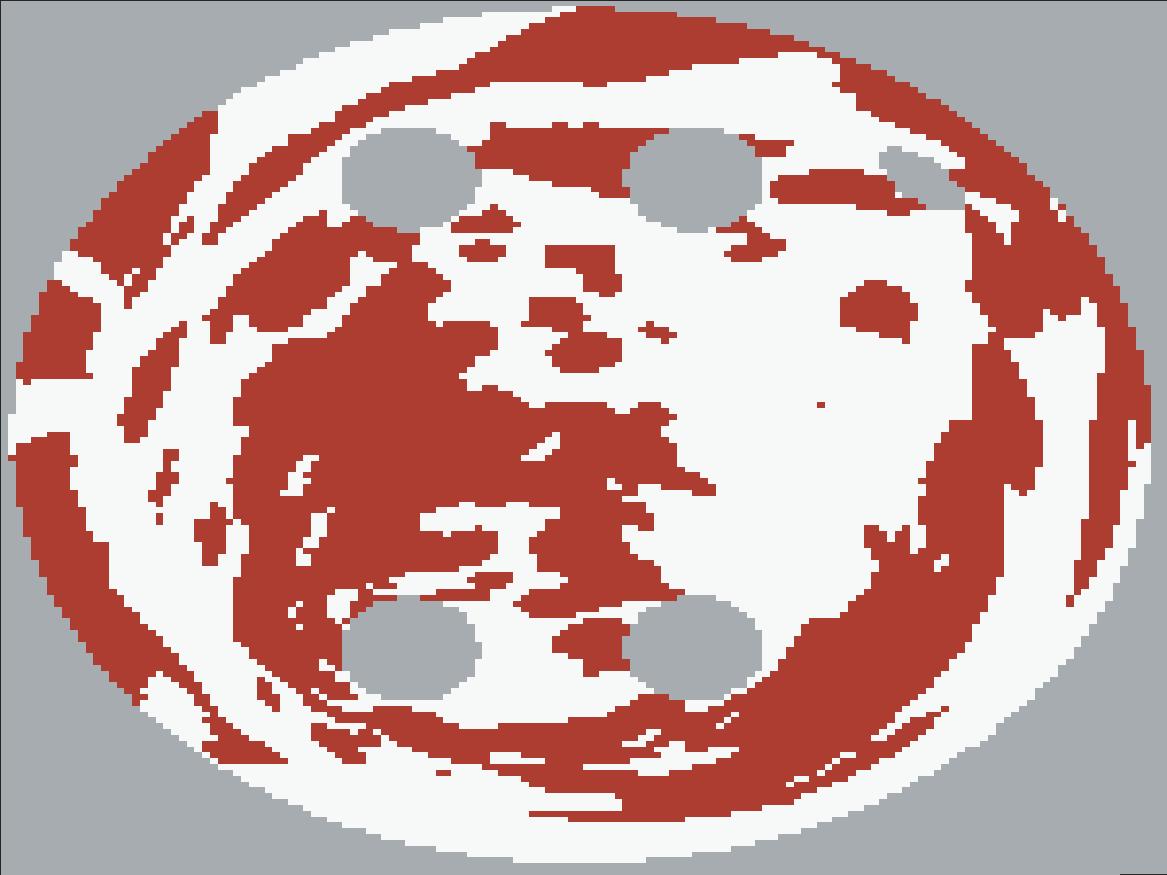

Supplement: Supplementary file 2 [file DataSheet1.ZIP › Dataset/real_UY2ZW.jpg]

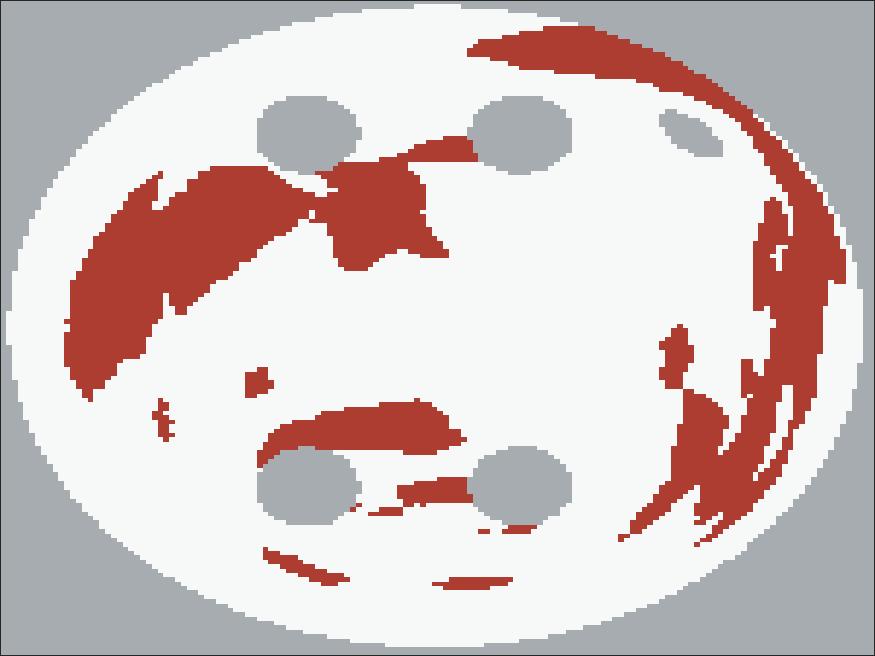

Supplement: Supplementary file 2 [file DataSheet1.ZIP › Dataset/real_V0U6Q.jpg]

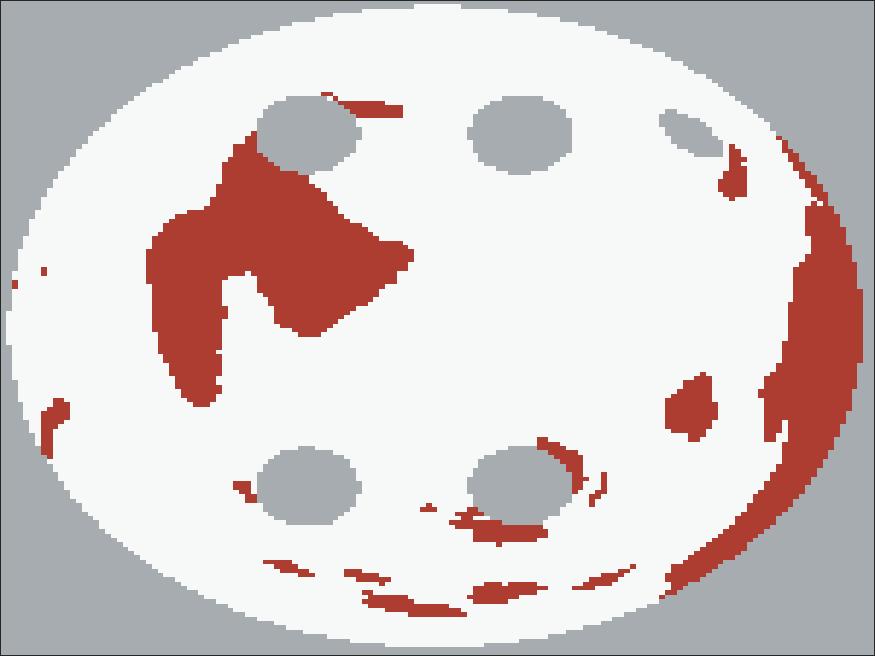

Supplement: Supplementary file 2 [file DataSheet1.ZIP › Dataset/real_V3H9F.jpg]

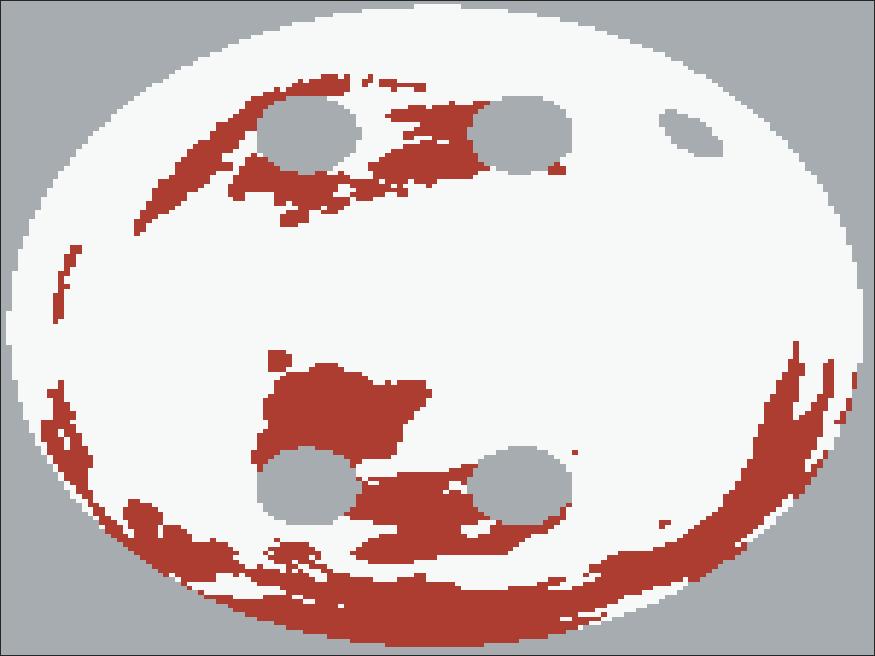

Supplement: Supplementary file 2 [file DataSheet1.ZIP › Dataset/real_VACZ0.jpg]

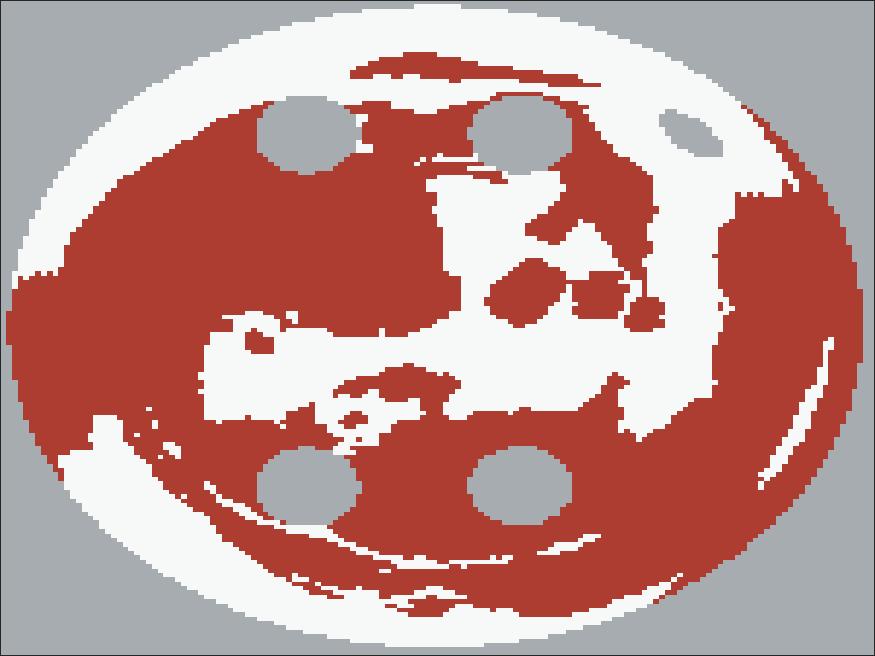

Supplement: Supplementary file 2 [file DataSheet1.ZIP › Dataset/real_VDQT7.jpg]

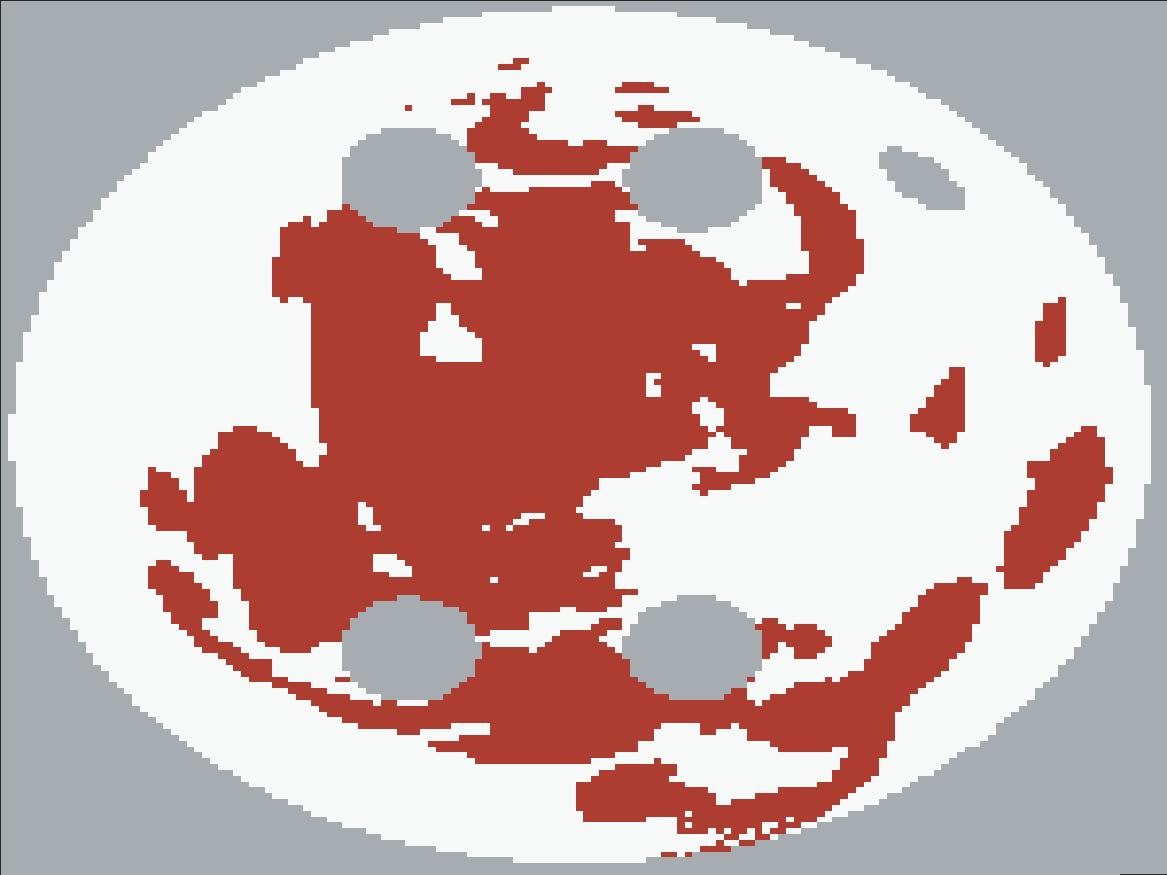

Supplement: Supplementary file 2 [file DataSheet1.ZIP › Dataset/real_VGL4N.jpg]

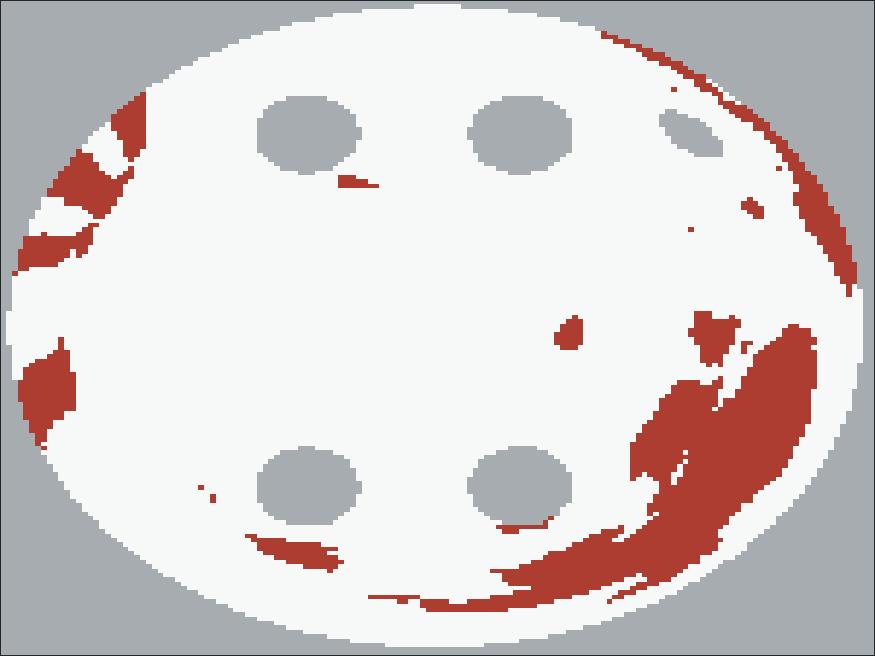

Supplement: Supplementary file 2 [file DataSheet1.ZIP › Dataset/real_VO0DX.jpg]

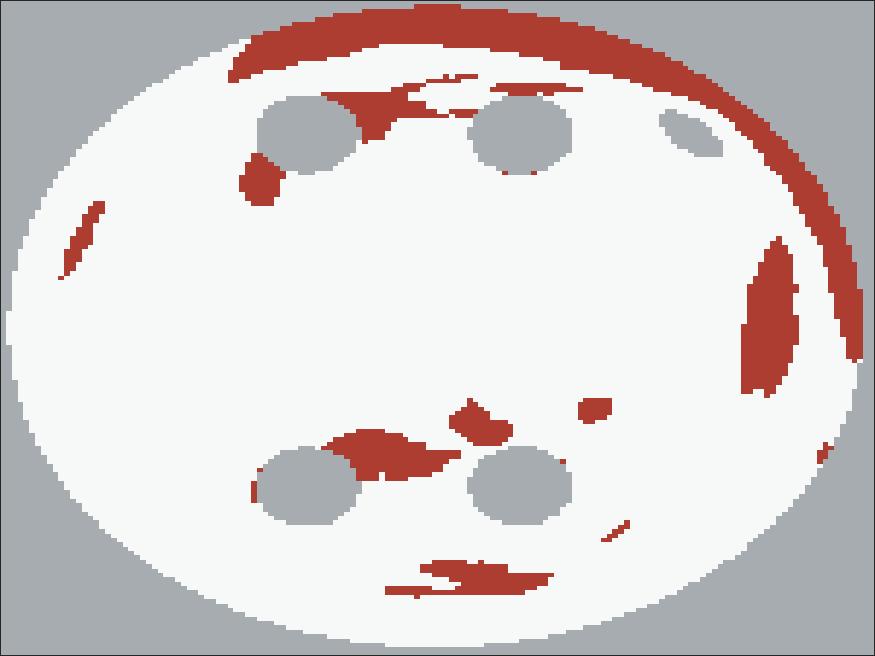

Supplement: Supplementary file 2 [file DataSheet1.ZIP › Dataset/real_VOQUV.jpg]

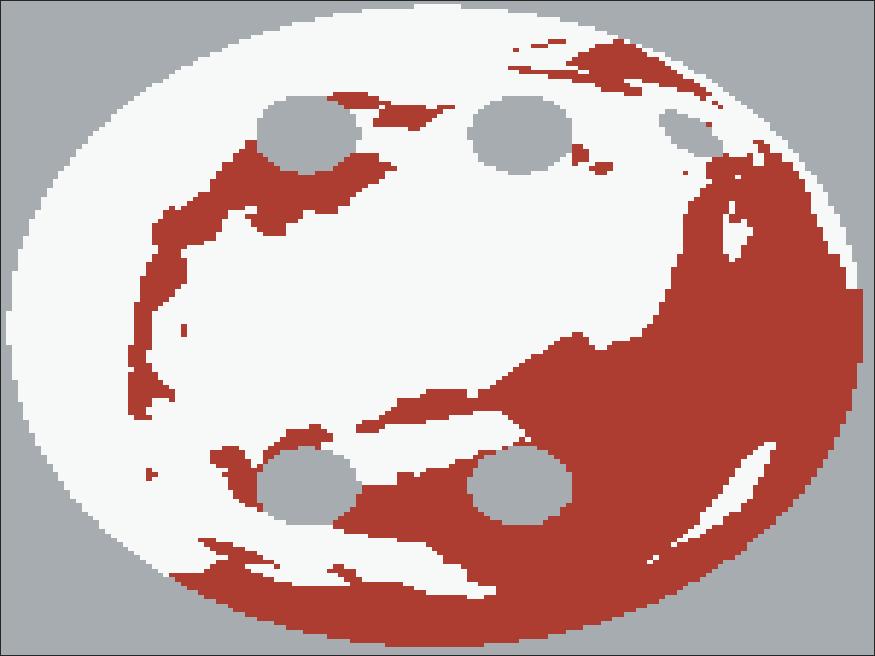

Supplement: Supplementary file 2 [file DataSheet1.ZIP › Dataset/real_VY1PQ.jpg]

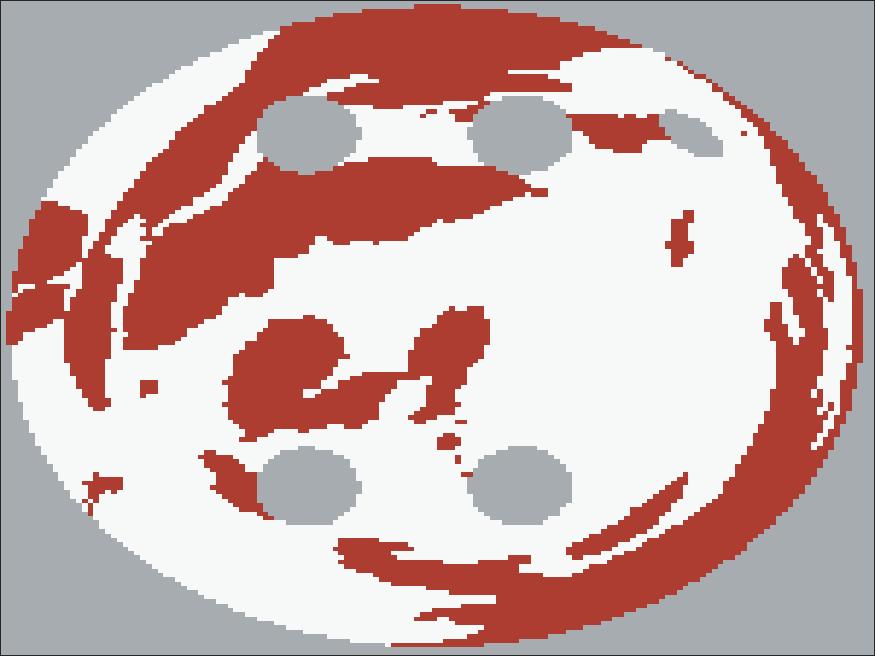

Supplement: Supplementary file 2 [file DataSheet1.ZIP › Dataset/real_WQRBU.jpg]

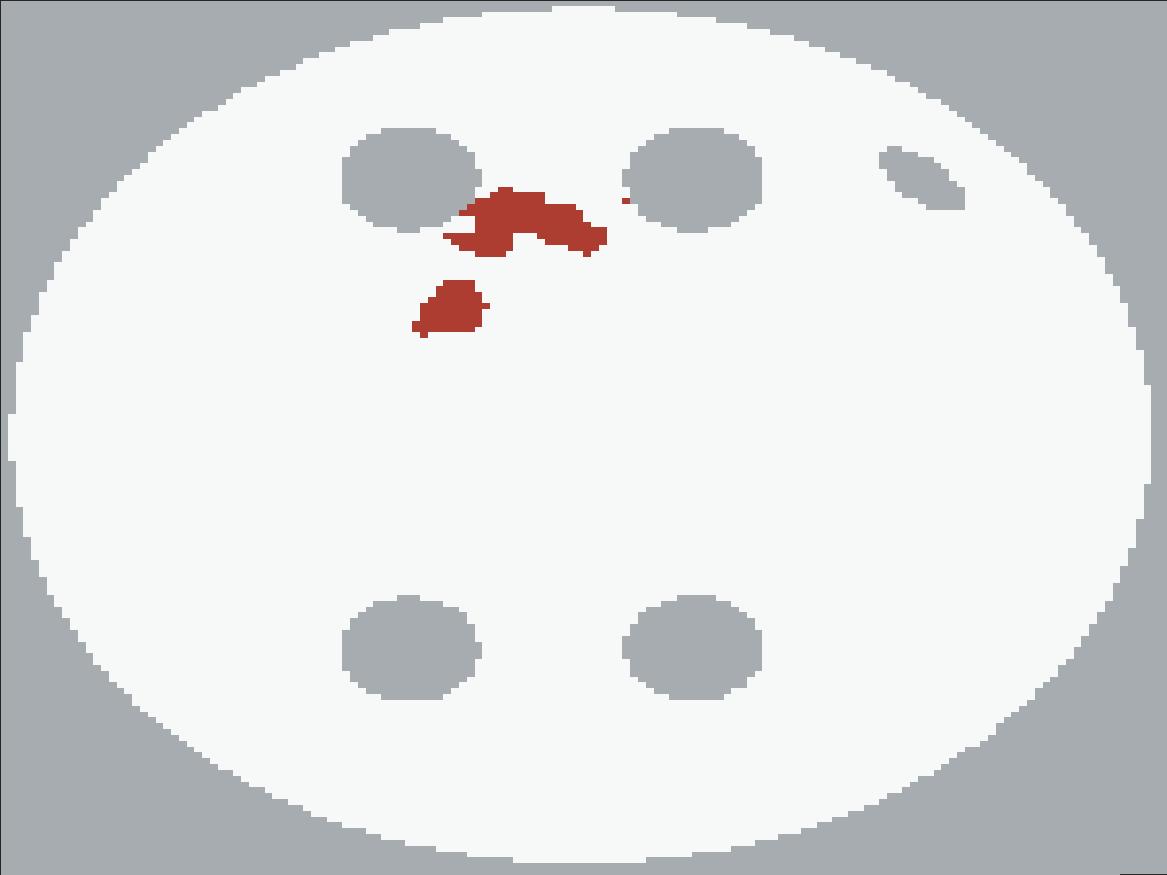

Supplement: Supplementary file 2 [file DataSheet1.ZIP › Dataset/real_WV2RO.jpg]

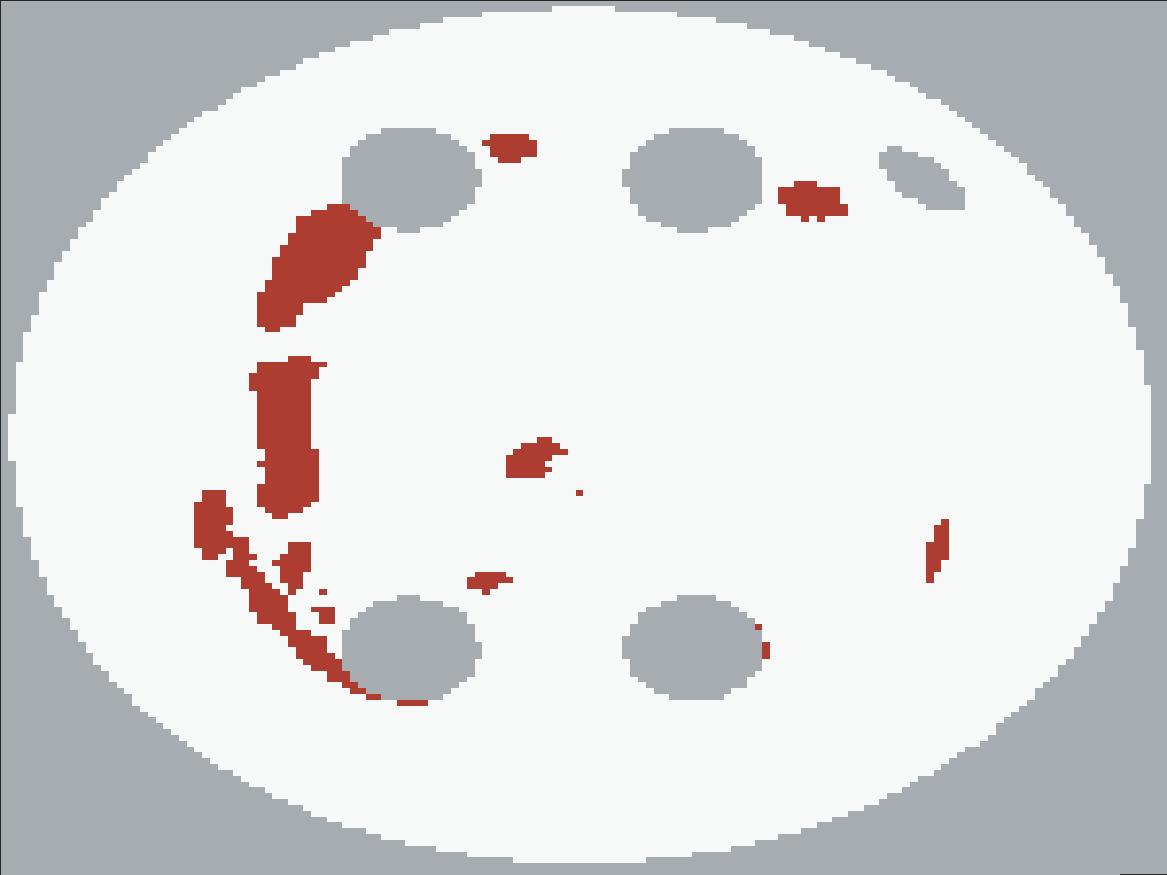

Supplement: Supplementary file 2 [file DataSheet1.ZIP › Dataset/real_XKPB2.jpg]

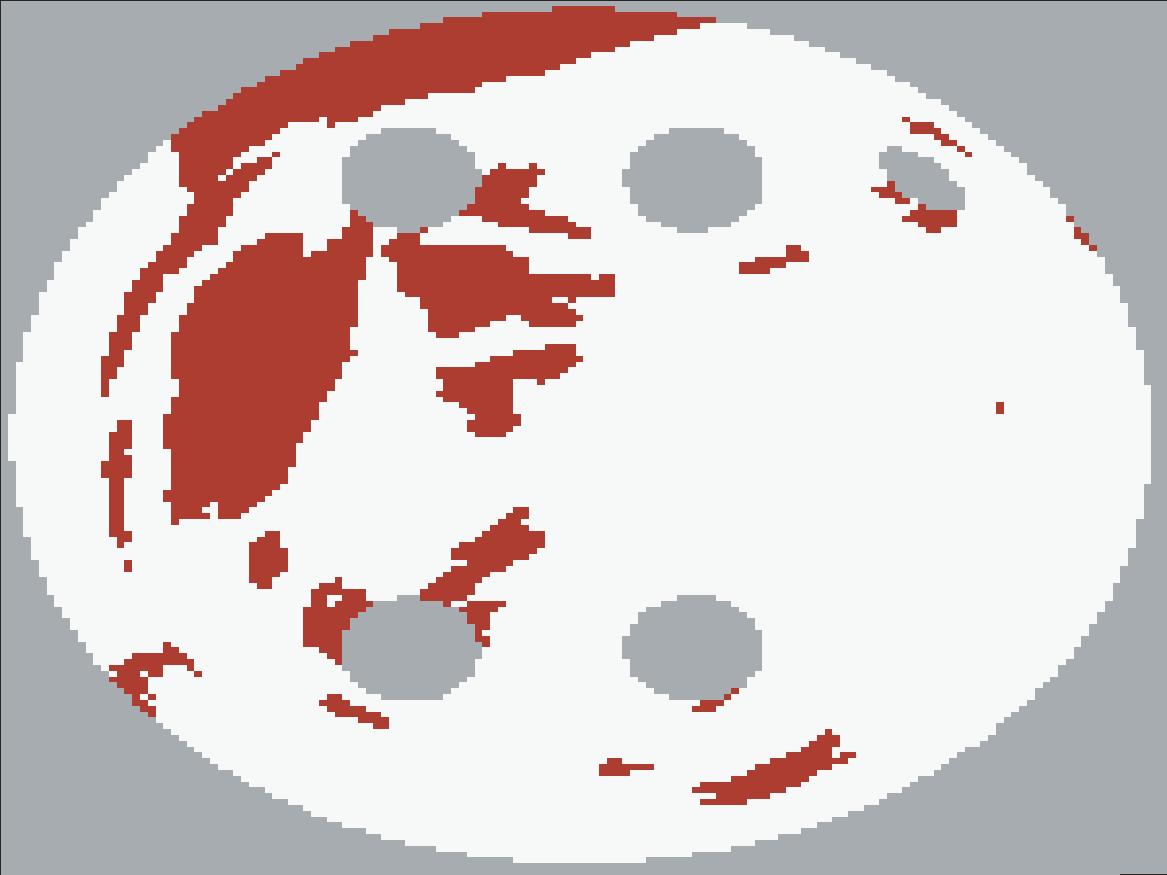

Supplement: Supplementary file 2 [file DataSheet1.ZIP › Dataset/real_Y0YVN.jpg]

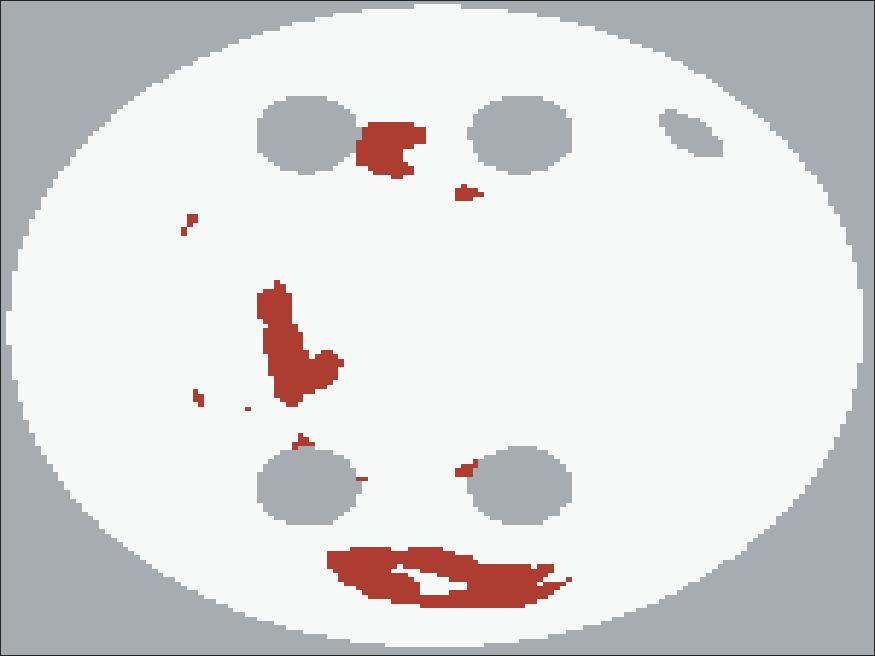

Supplement: Supplementary file 2 [file DataSheet1.ZIP › Dataset/real_Y4WGV.jpg]

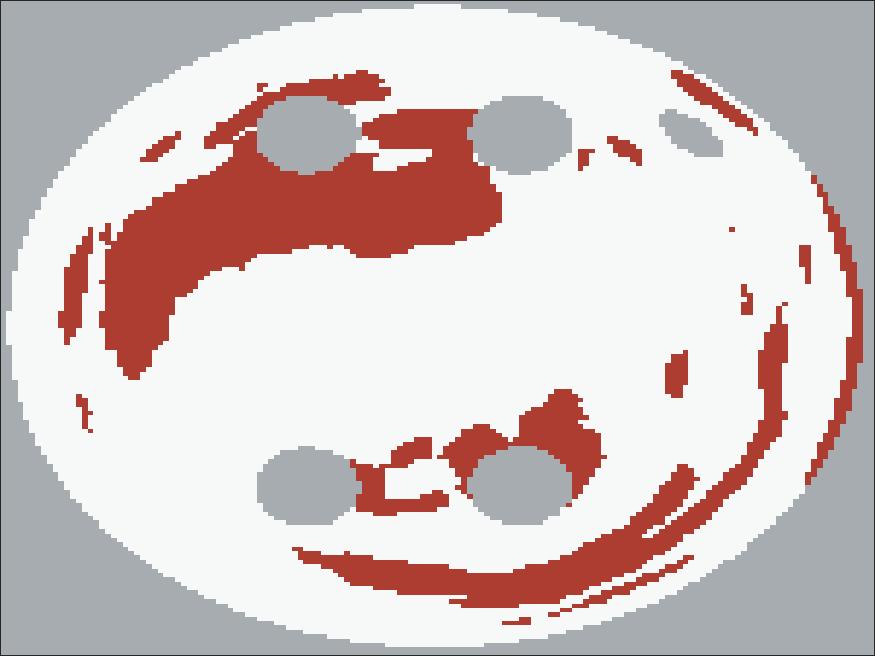

Supplement: Supplementary file 2 [file DataSheet1.ZIP › Dataset/real_Y61V6.jpg]

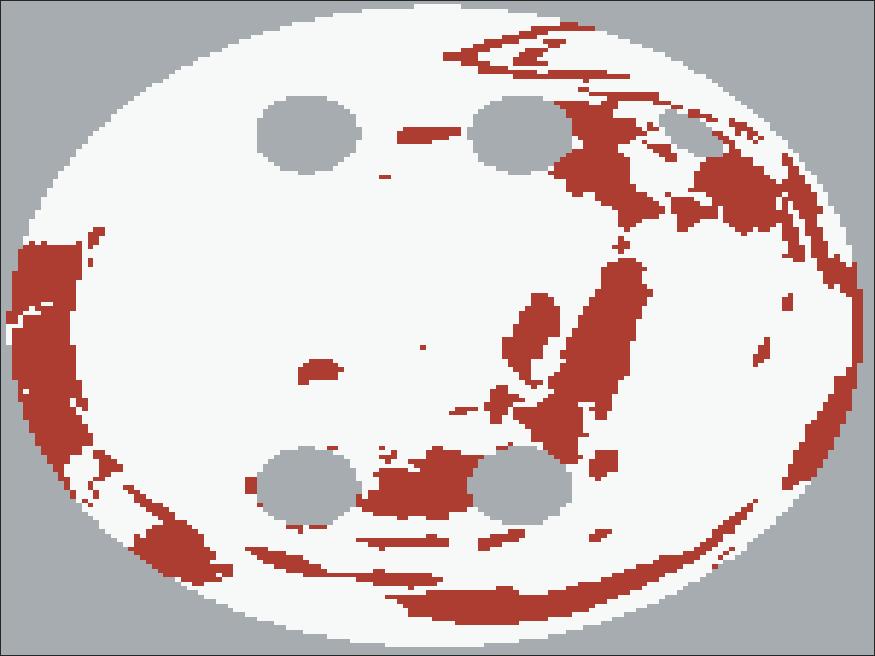

Supplement: Supplementary file 2 [file DataSheet1.ZIP › Dataset/real_Y6IRH.jpg]

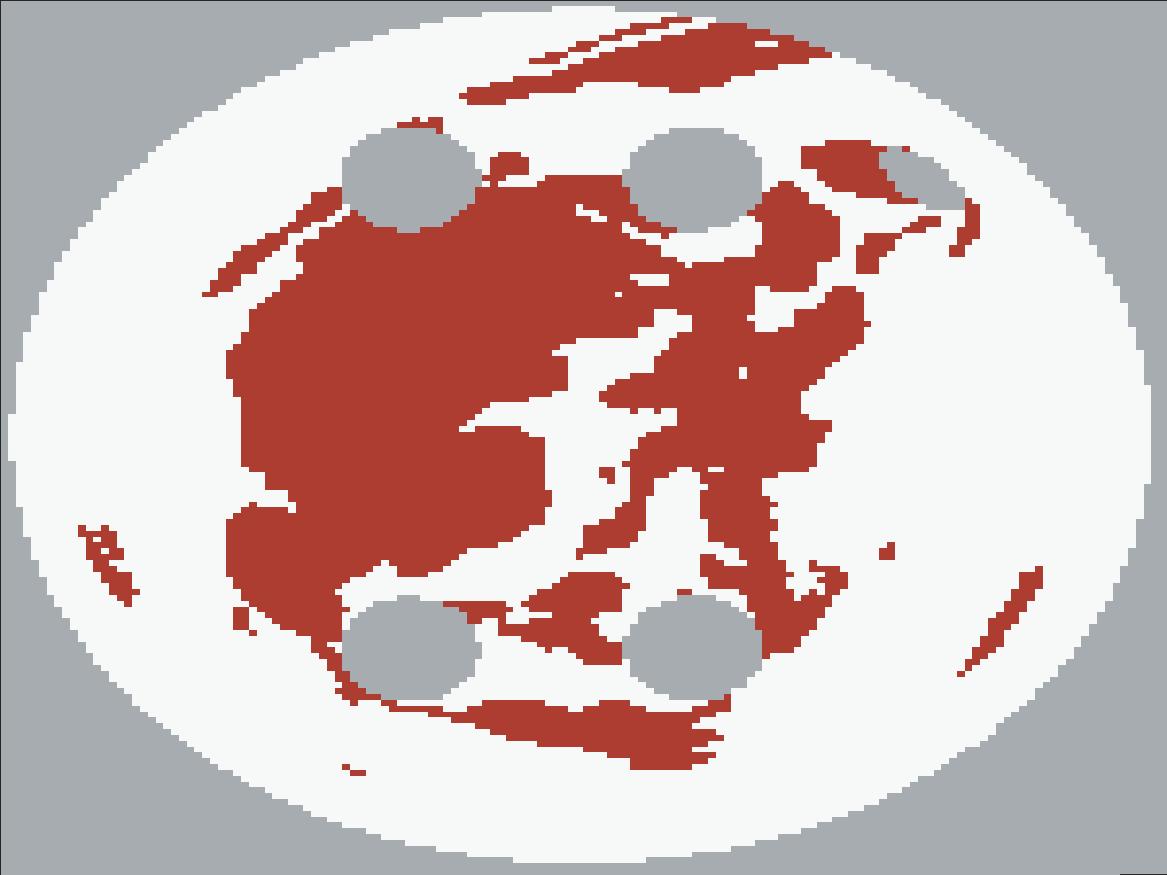

Supplement: Supplementary file 2 [file DataSheet1.ZIP › Dataset/real_YLZ7E.jpg]

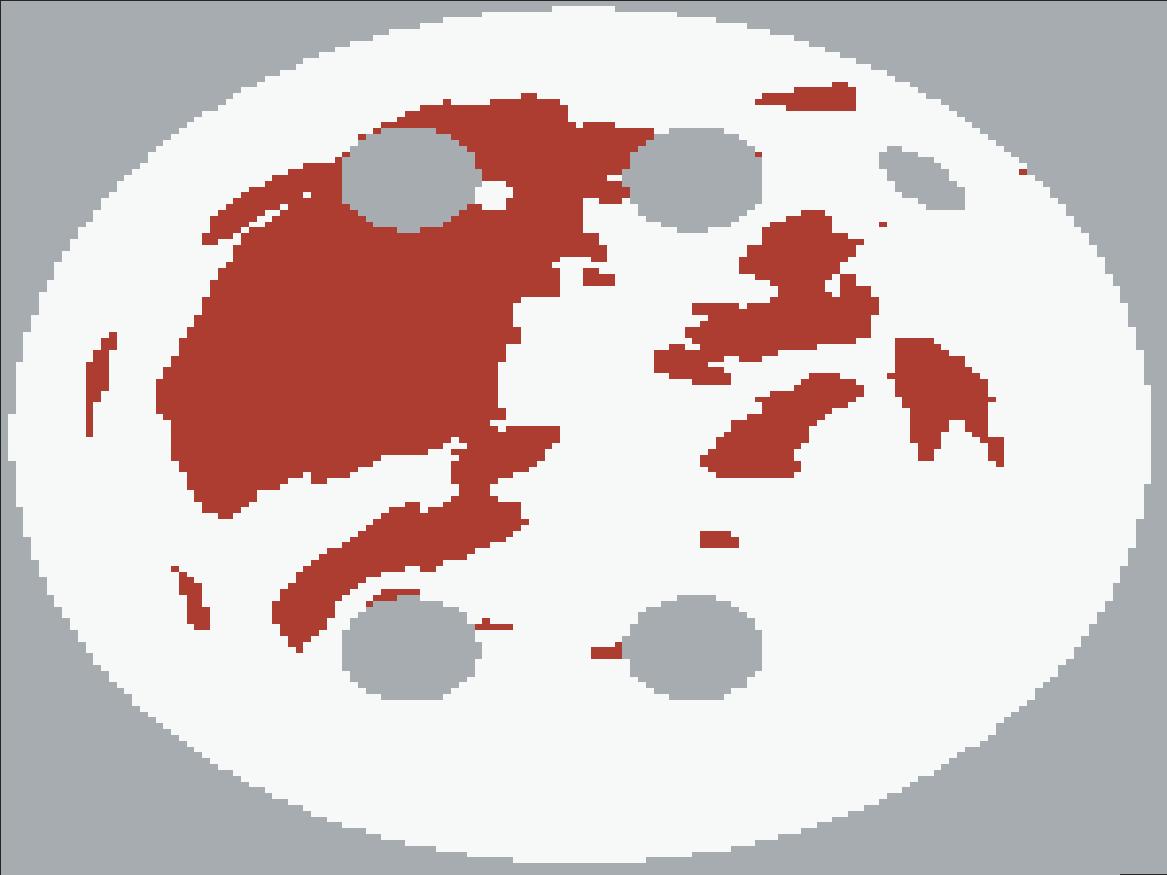

Supplement: Supplementary file 2 [file DataSheet1.ZIP › Dataset/real_YRIS4.jpg]

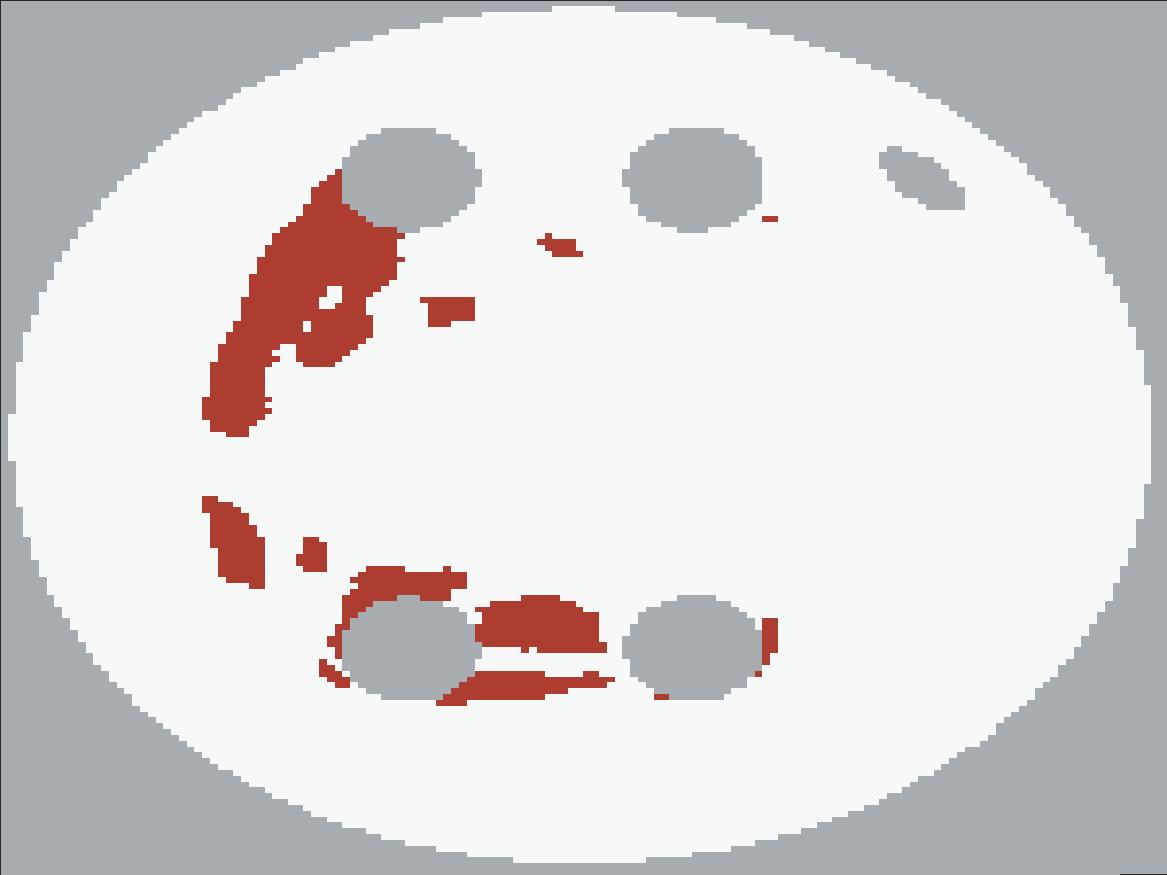

Supplement: Supplementary file 2 [file DataSheet1.ZIP › Dataset/real_ZCIXM.jpg]

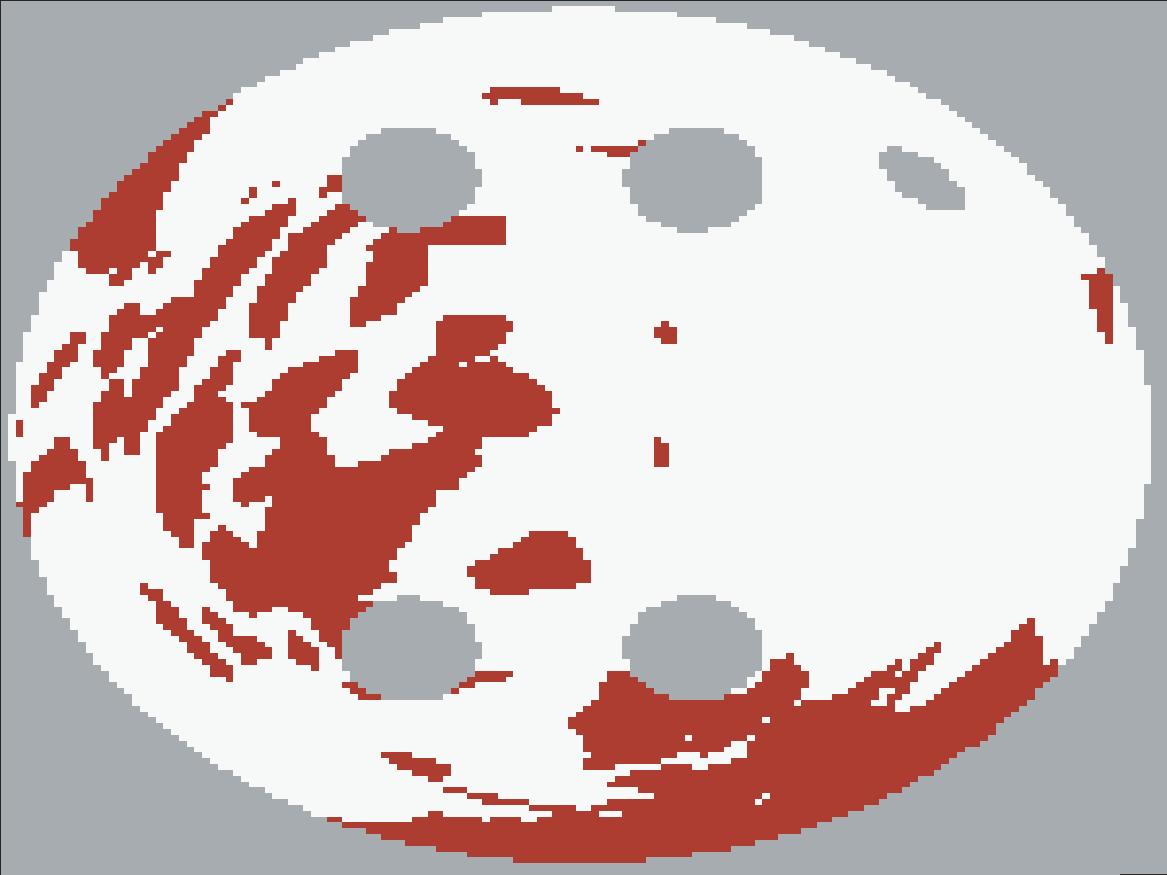

Supplement: Supplementary file 2 [file DataSheet1.ZIP › Dataset/real_ZLSTF.jpg]

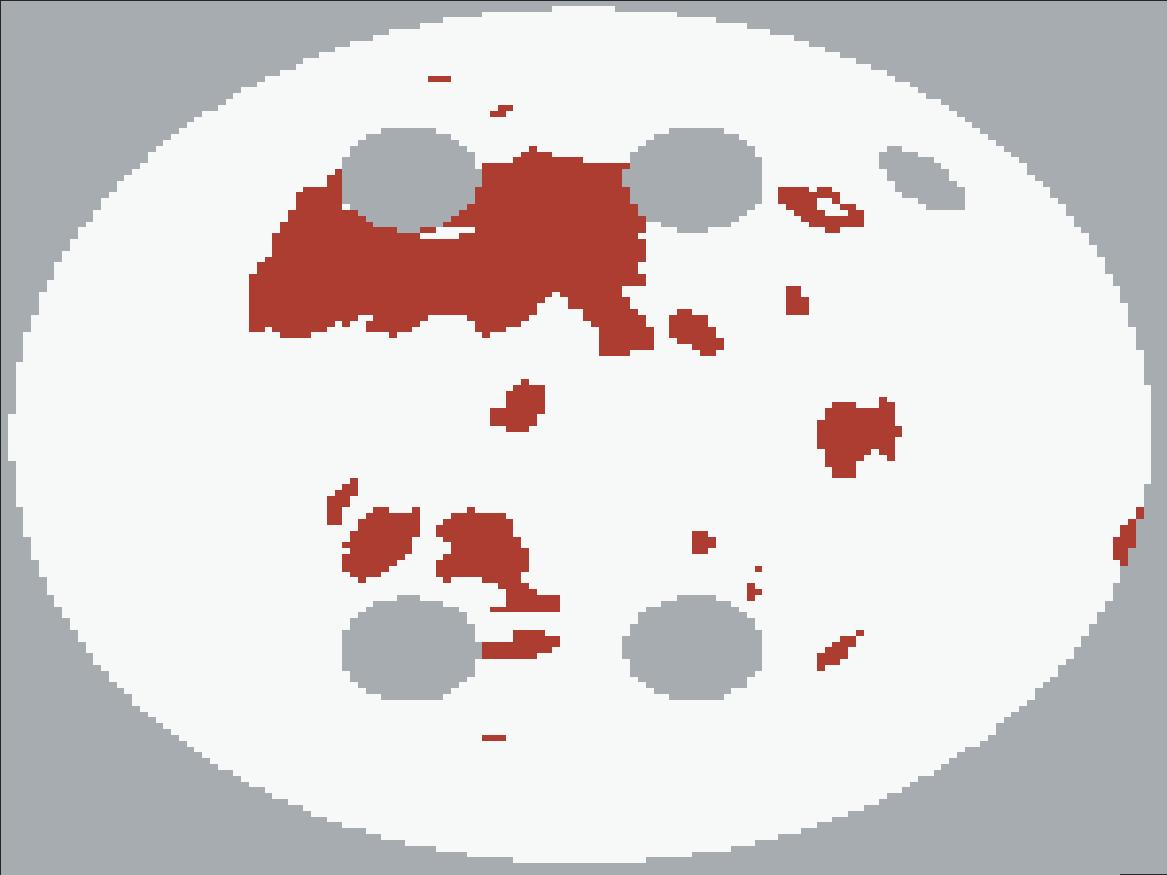

Supplement: Supplementary file 2 [file DataSheet1.ZIP › Dataset/real_ZR142.jpg]

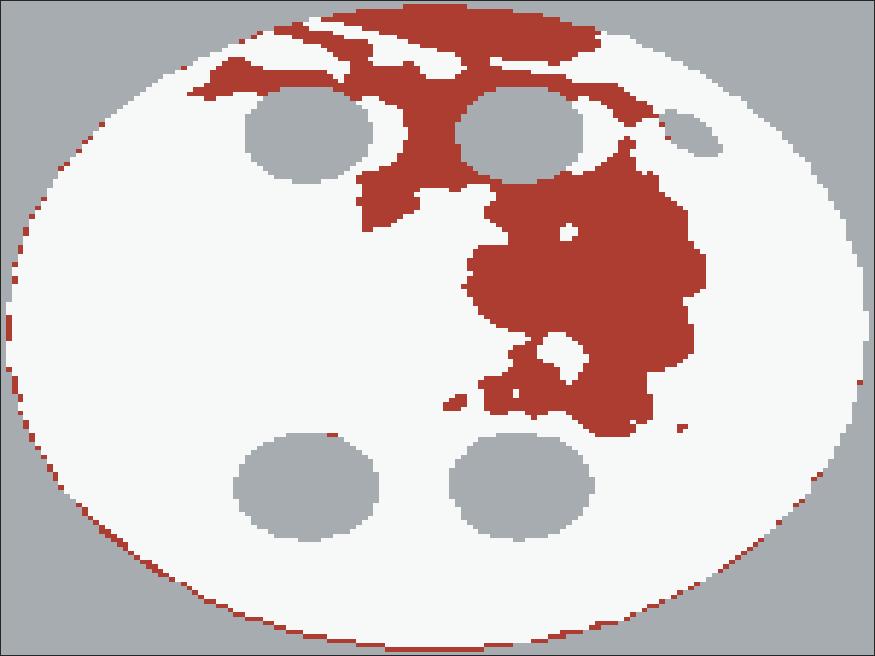

Supplement: Supplementary file 2 [file DataSheet1.ZIP › Dataset/synthetic_07J4G.jpg]

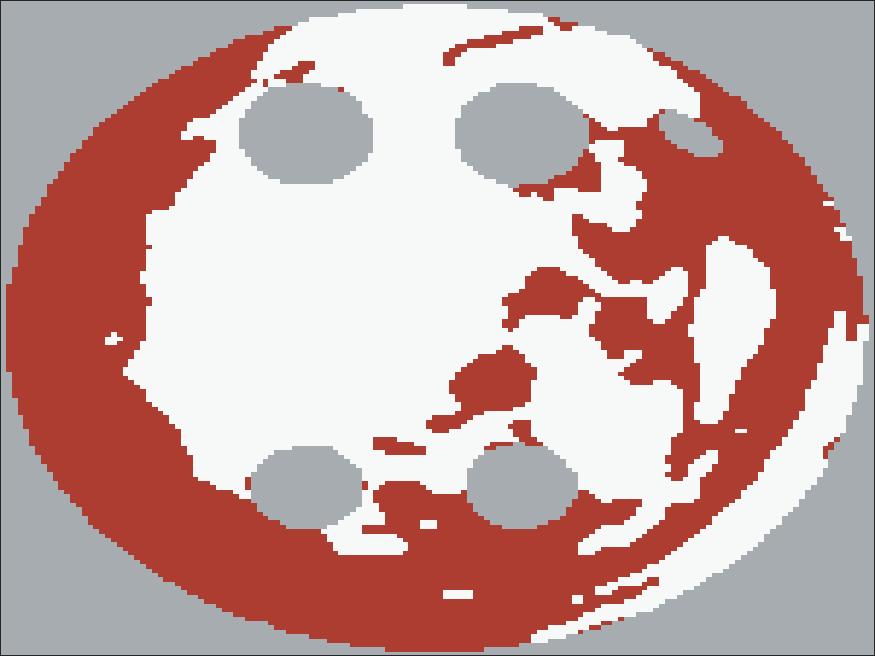

Supplement: Supplementary file 2 [file DataSheet1.ZIP › Dataset/synthetic_087R5.jpg]

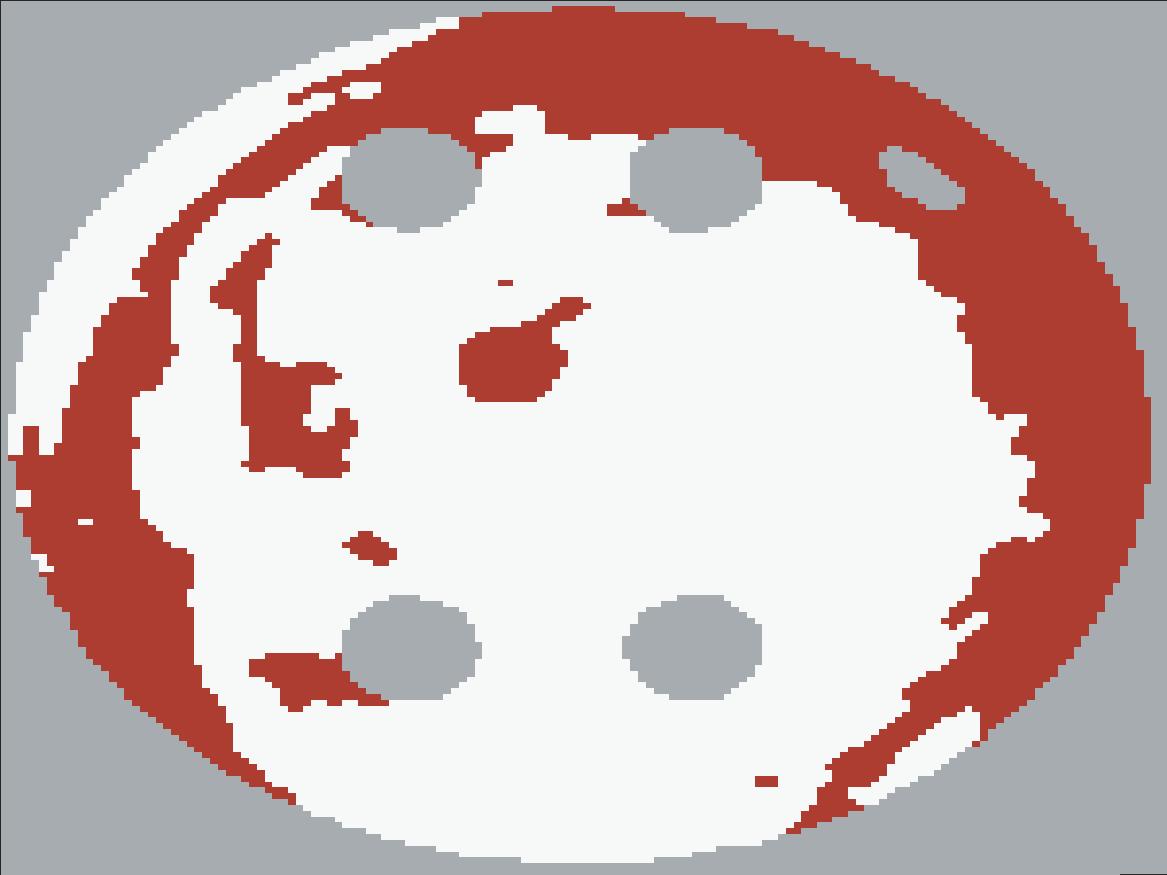

Supplement: Supplementary file 2 [file DataSheet1.ZIP › Dataset/synthetic_0M486.jpg]

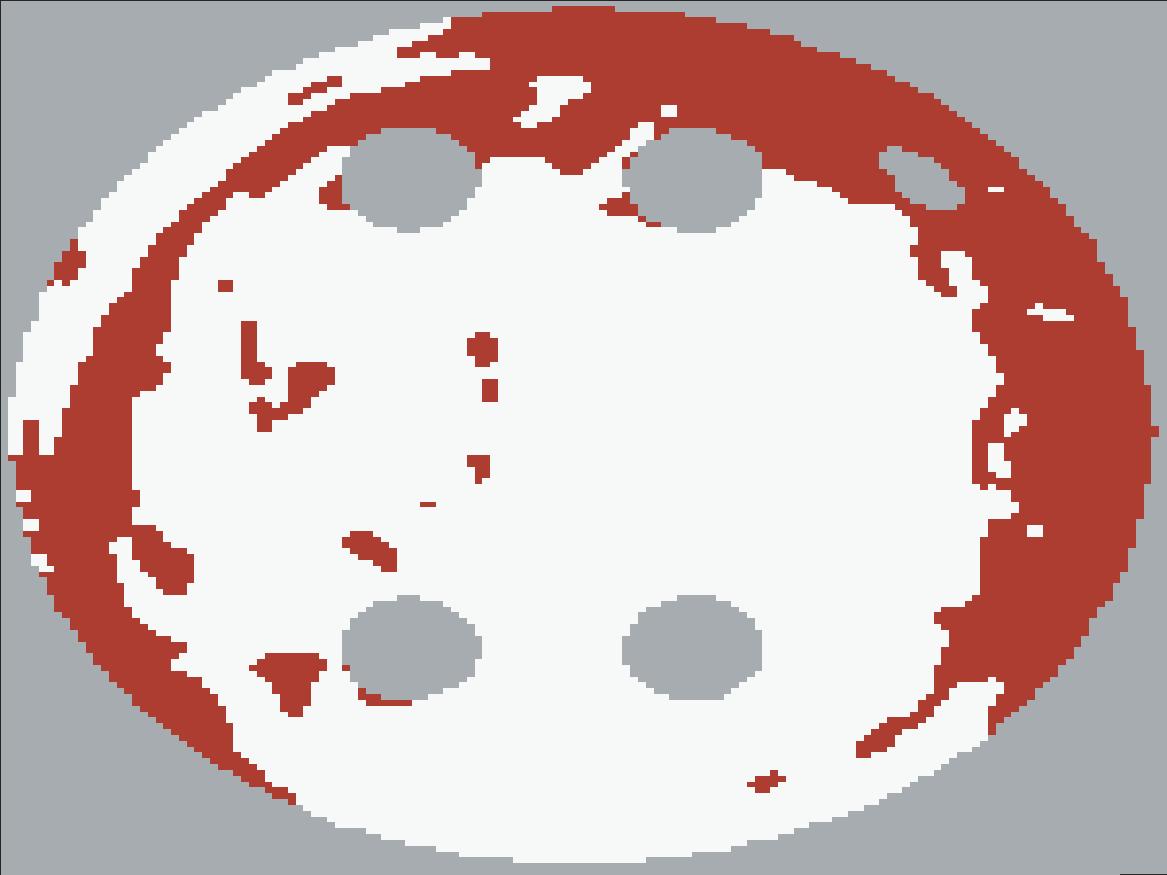

Supplement: Supplementary file 2 [file DataSheet1.ZIP › Dataset/synthetic_0N8I2.jpg]

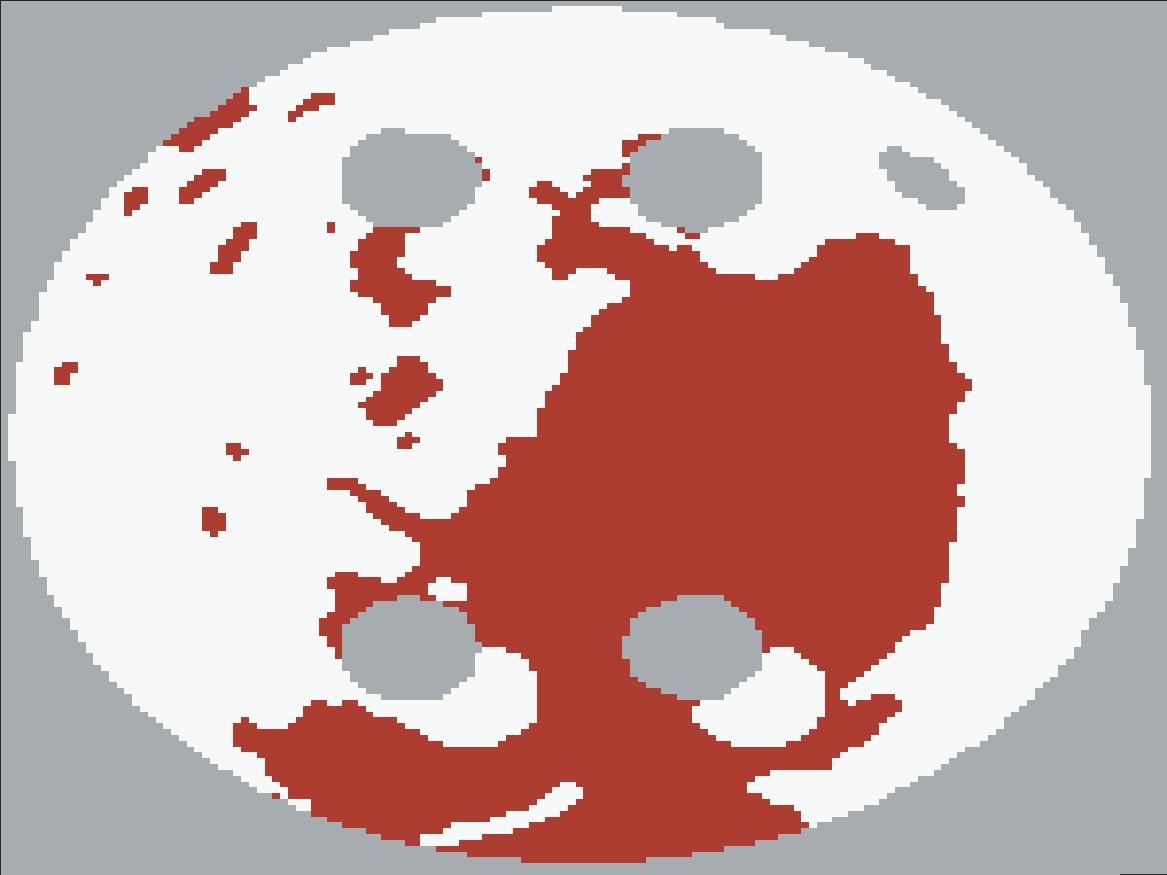

Supplement: Supplementary file 2 [file DataSheet1.ZIP › Dataset/synthetic_0SUAZ.jpg]

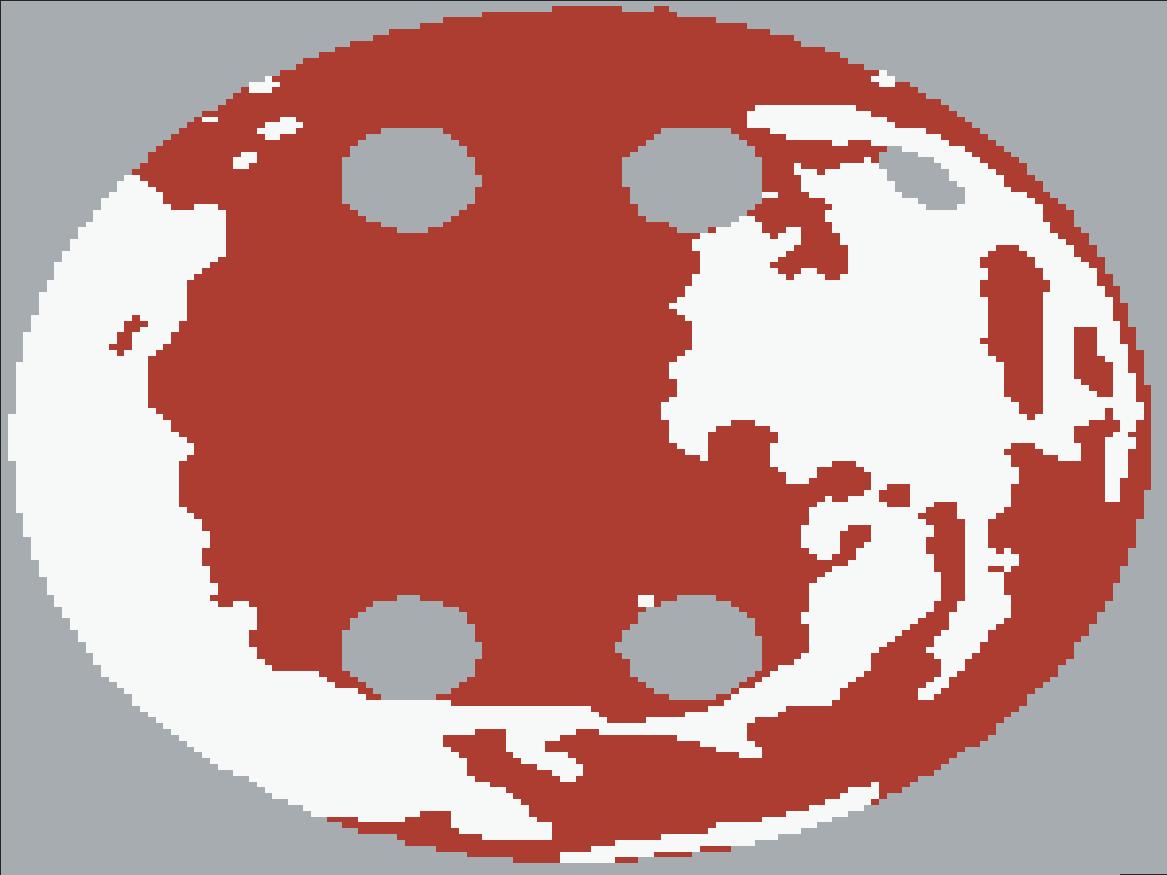

Supplement: Supplementary file 2 [file DataSheet1.ZIP › Dataset/synthetic_0T53Q.jpg]

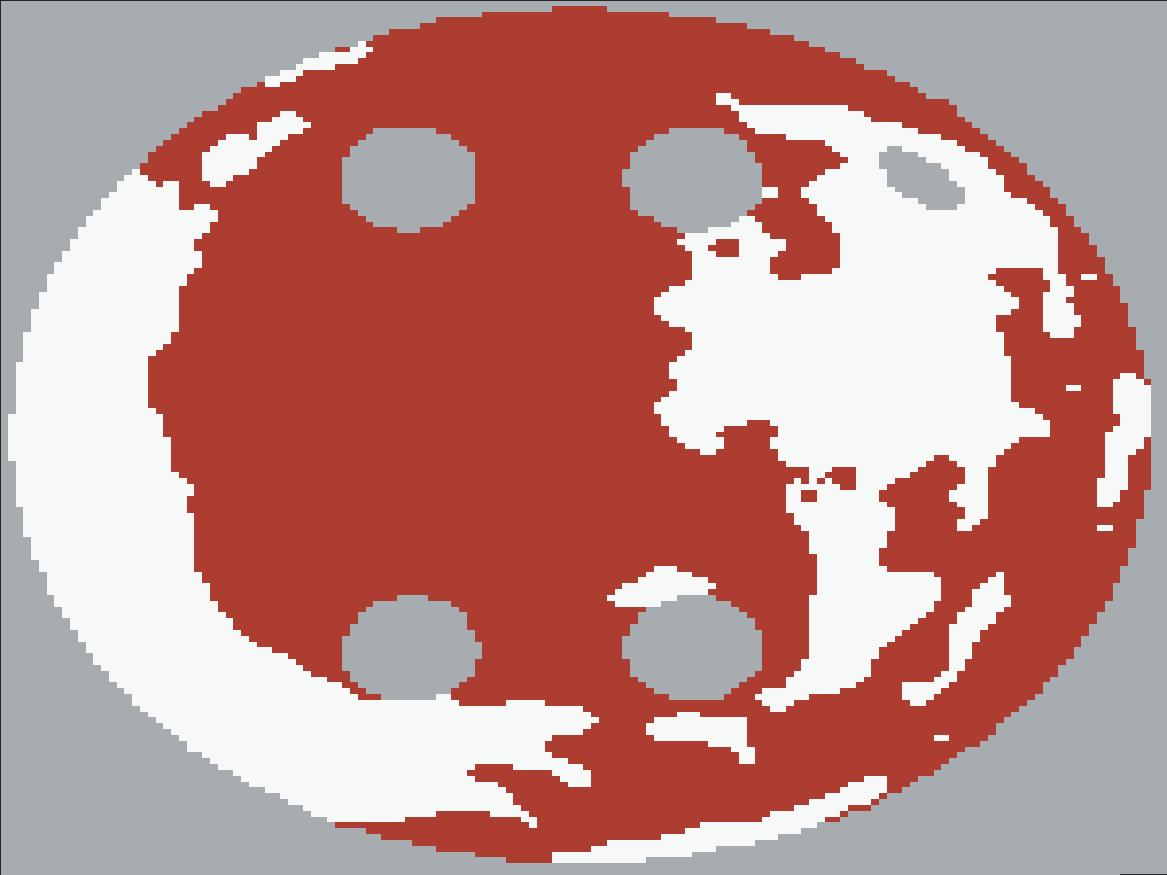

Supplement: Supplementary file 2 [file DataSheet1.ZIP › Dataset/synthetic_0XMZ0.jpg]

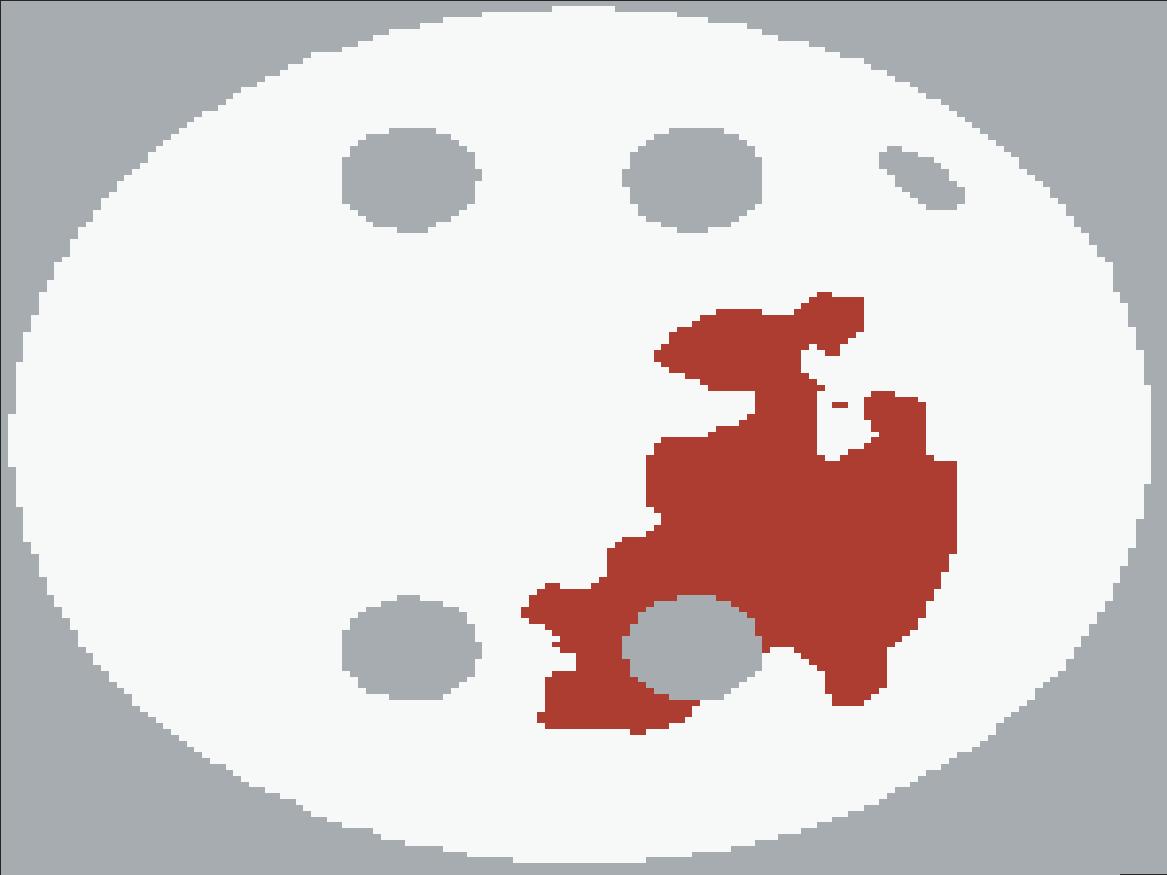

Supplement: Supplementary file 2 [file DataSheet1.ZIP › Dataset/synthetic_136MC.jpg]

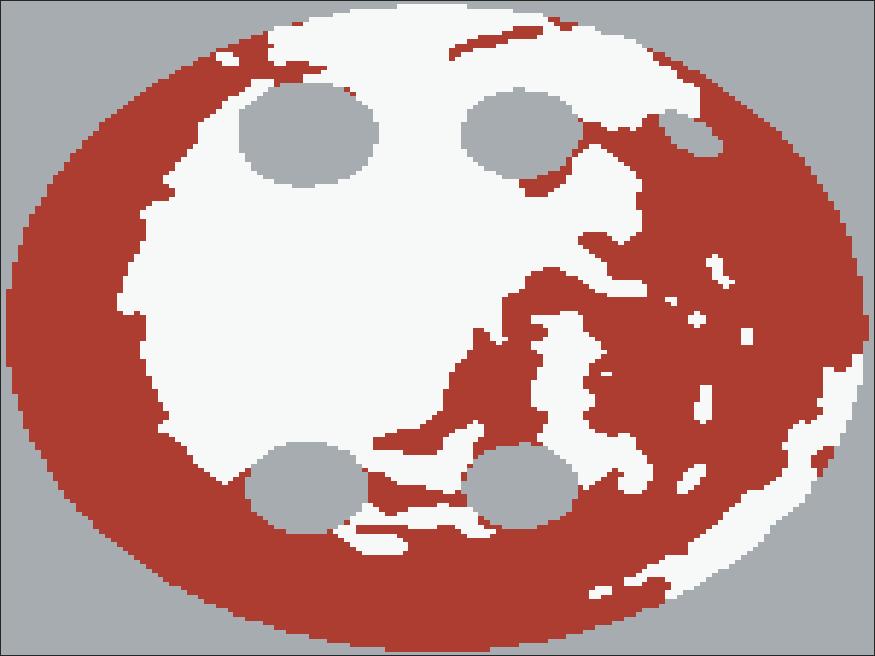

Supplement: Supplementary file 2 [file DataSheet1.ZIP › Dataset/synthetic_1E2K0.jpg]

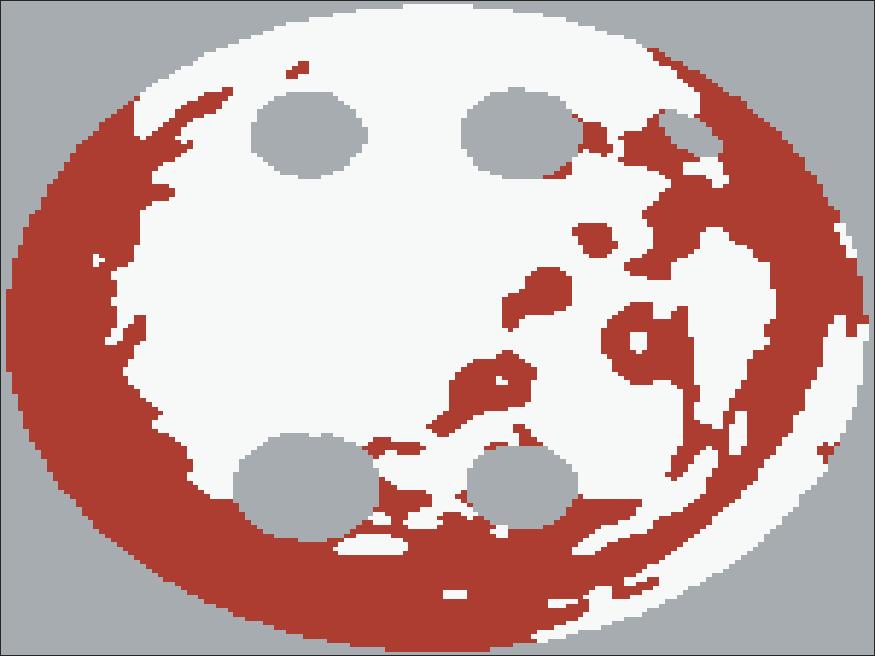

Supplement: Supplementary file 2 [file DataSheet1.ZIP › Dataset/synthetic_1KPF0.jpg]

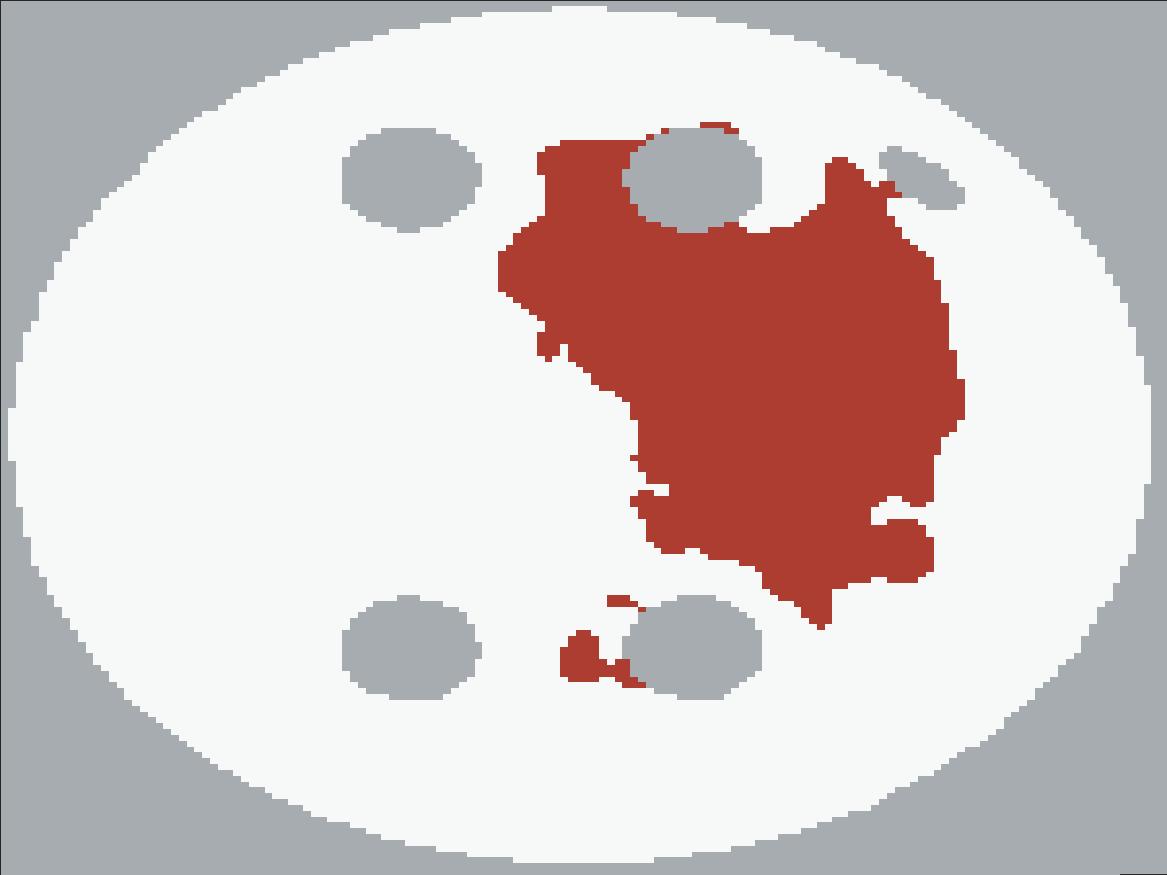

Supplement: Supplementary file 2 [file DataSheet1.ZIP › Dataset/synthetic_1RWXR.jpg]

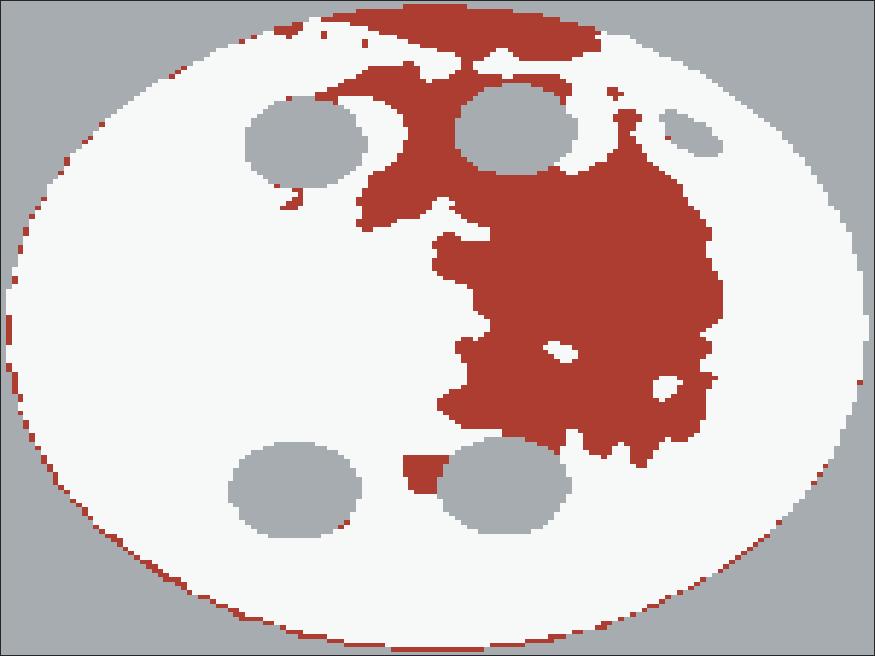

Supplement: Supplementary file 2 [file DataSheet1.ZIP › Dataset/synthetic_1Y4M1.jpg]

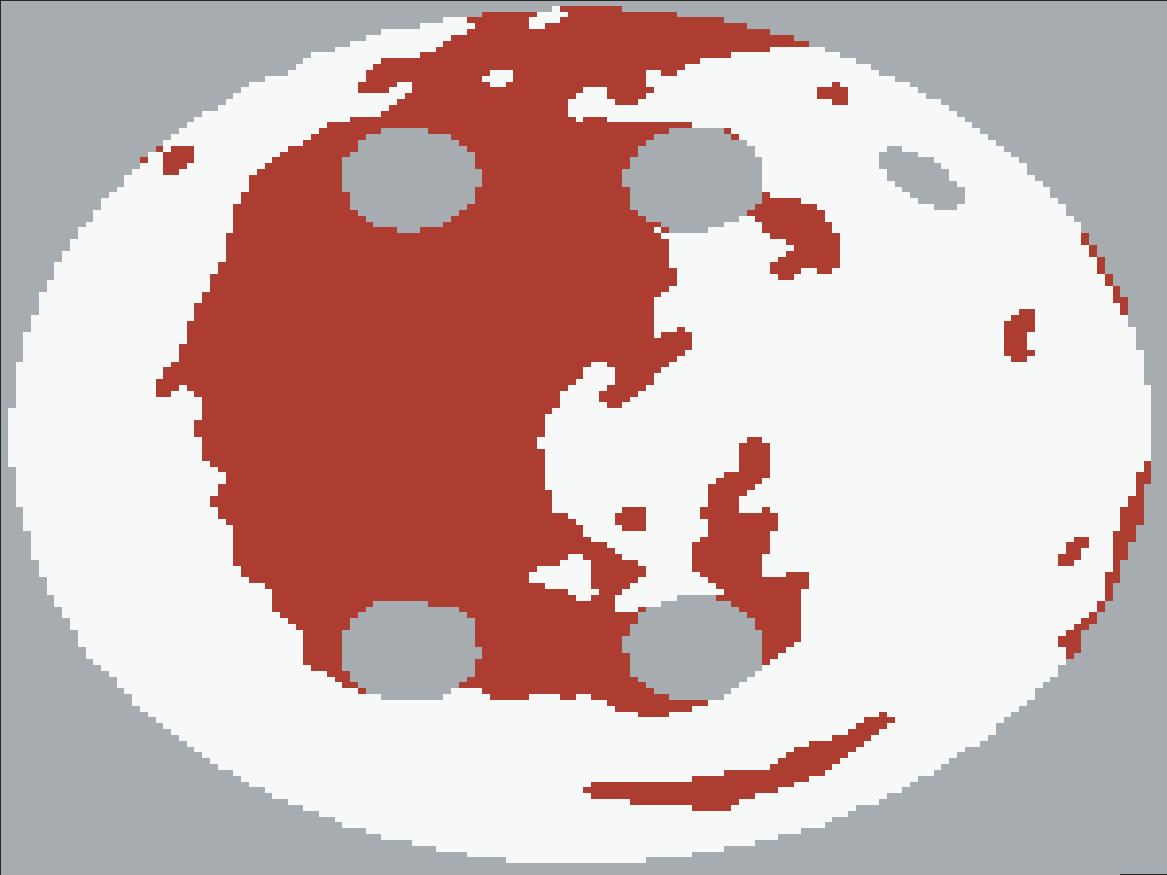

Supplement: Supplementary file 2 [file DataSheet1.ZIP › Dataset/synthetic_27ESI.jpg]

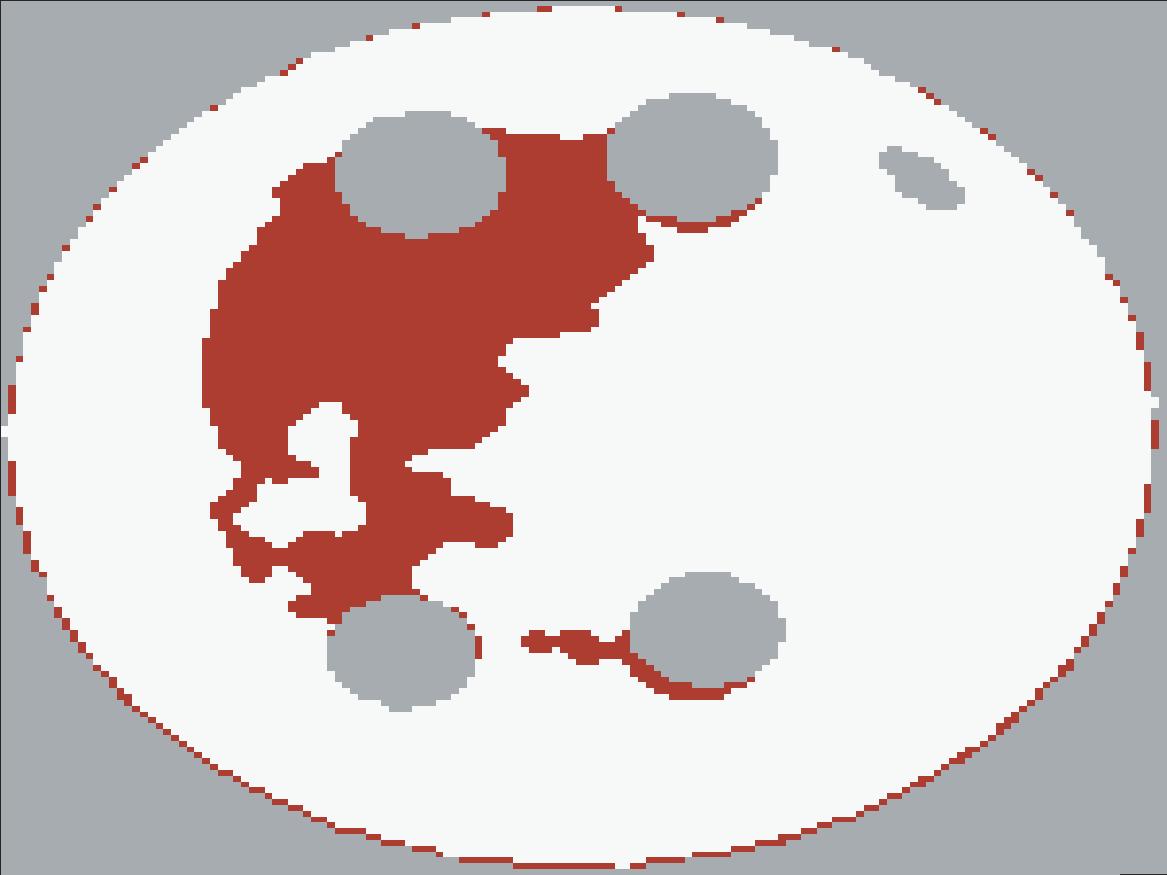

Supplement: Supplementary file 2 [file DataSheet1.ZIP › Dataset/synthetic_2A88K.jpg]

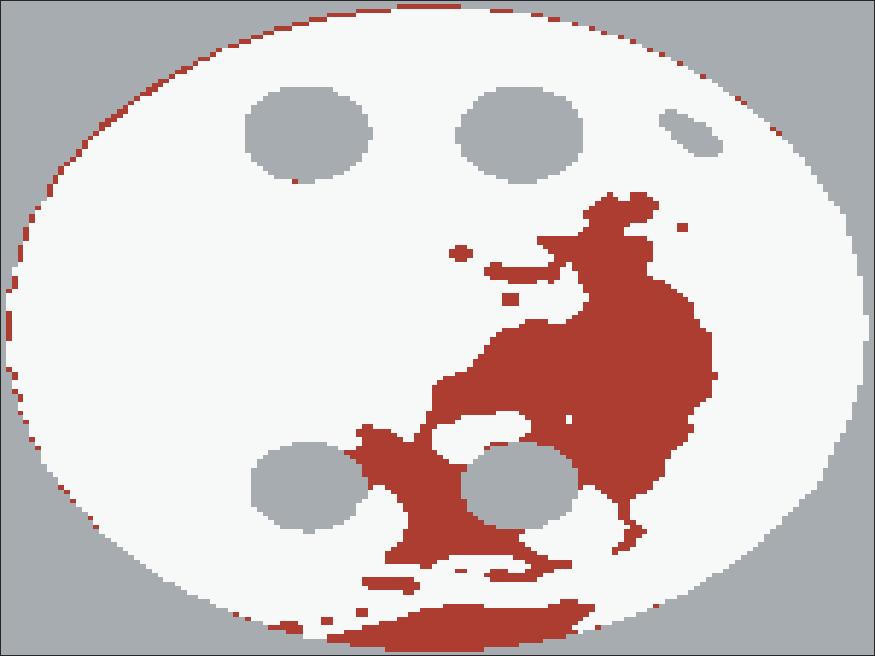

Supplement: Supplementary file 2 [file DataSheet1.ZIP › Dataset/synthetic_2FMSK.jpg]

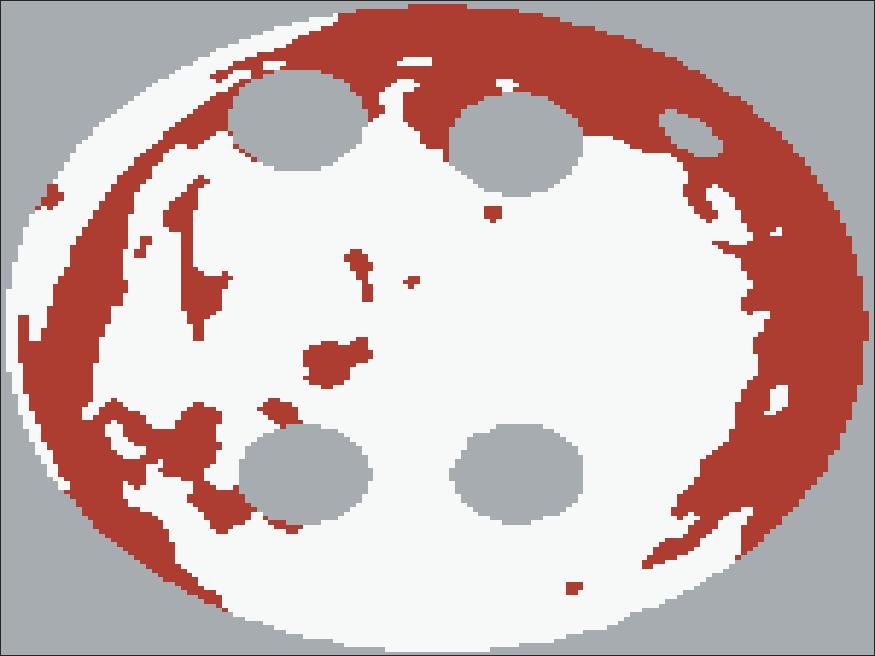

Supplement: Supplementary file 2 [file DataSheet1.ZIP › Dataset/synthetic_2L01K.jpg]

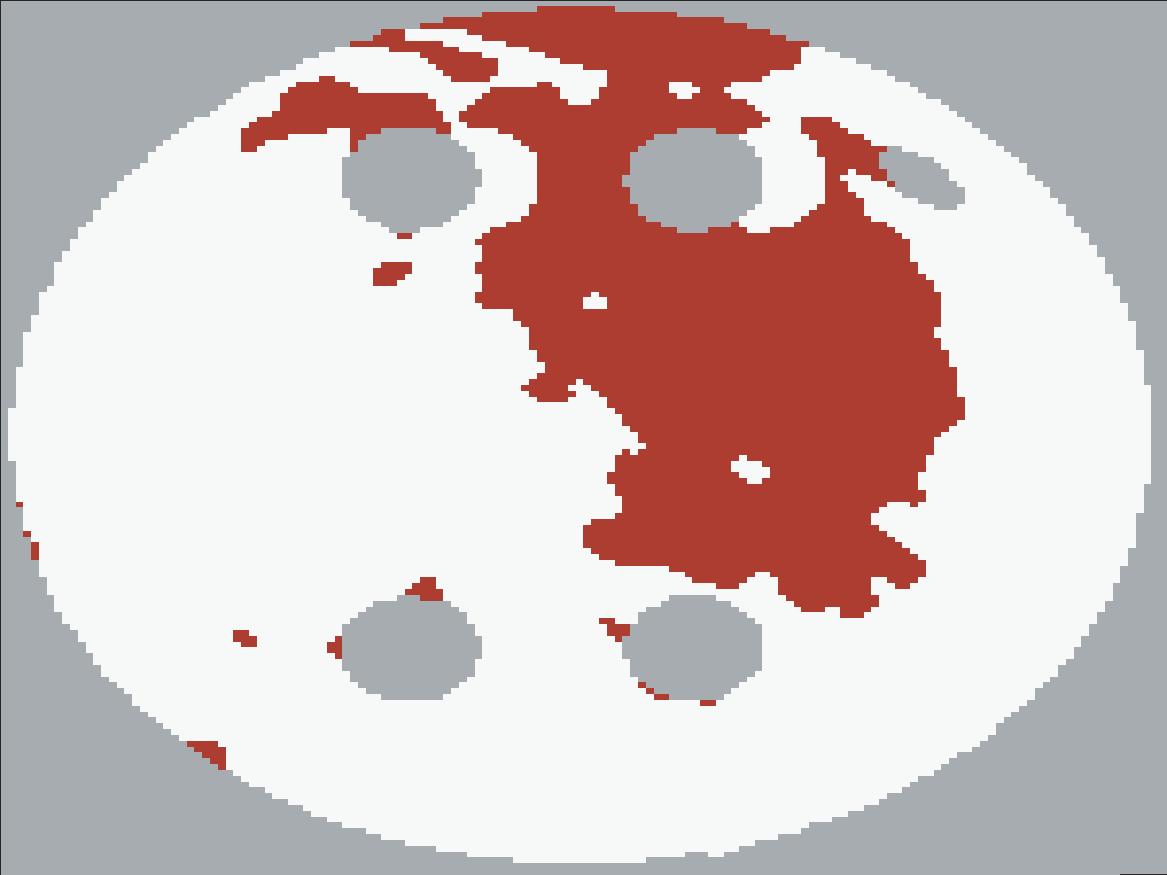

Supplement: Supplementary file 2 [file DataSheet1.ZIP › Dataset/synthetic_2X765.jpg]

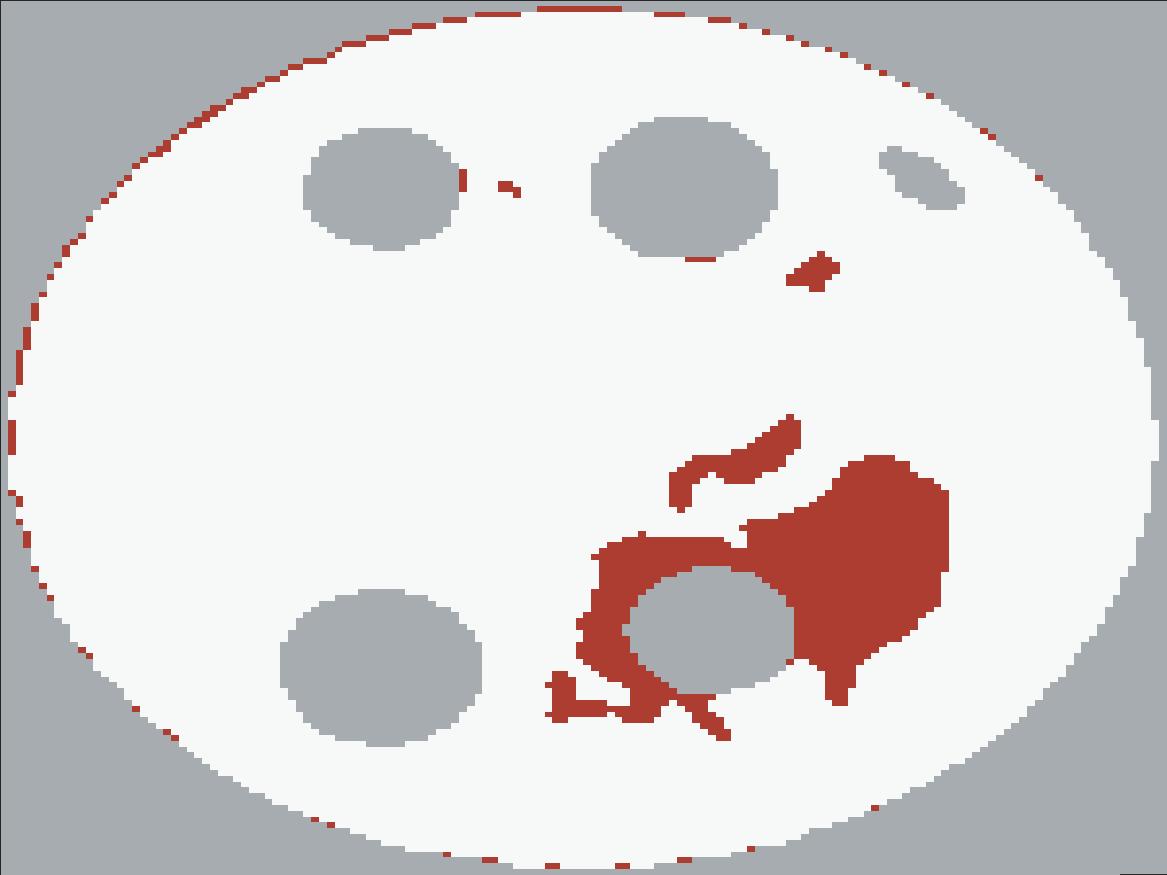

Supplement: Supplementary file 2 [file DataSheet1.ZIP › Dataset/synthetic_2Z0BO.jpg]

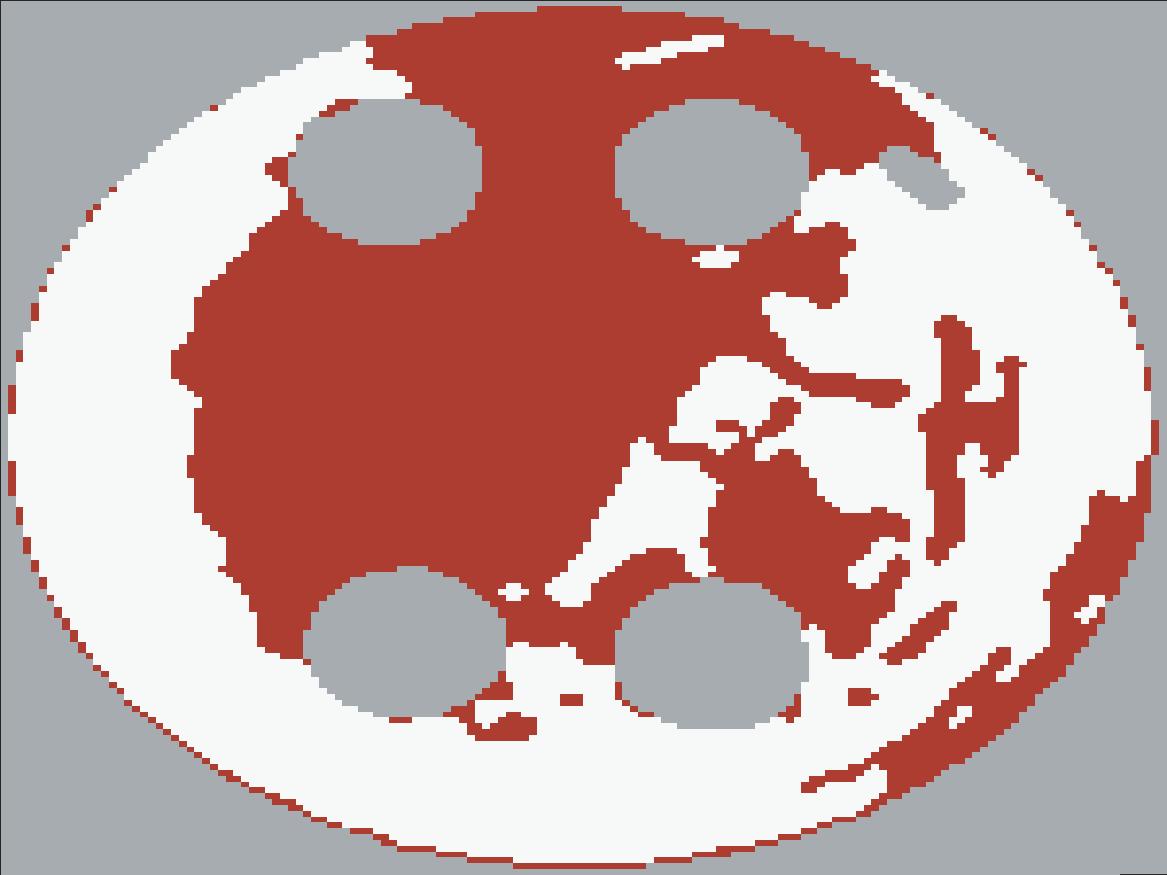

Supplement: Supplementary file 2 [file DataSheet1.ZIP › Dataset/synthetic_33EAT.jpg]

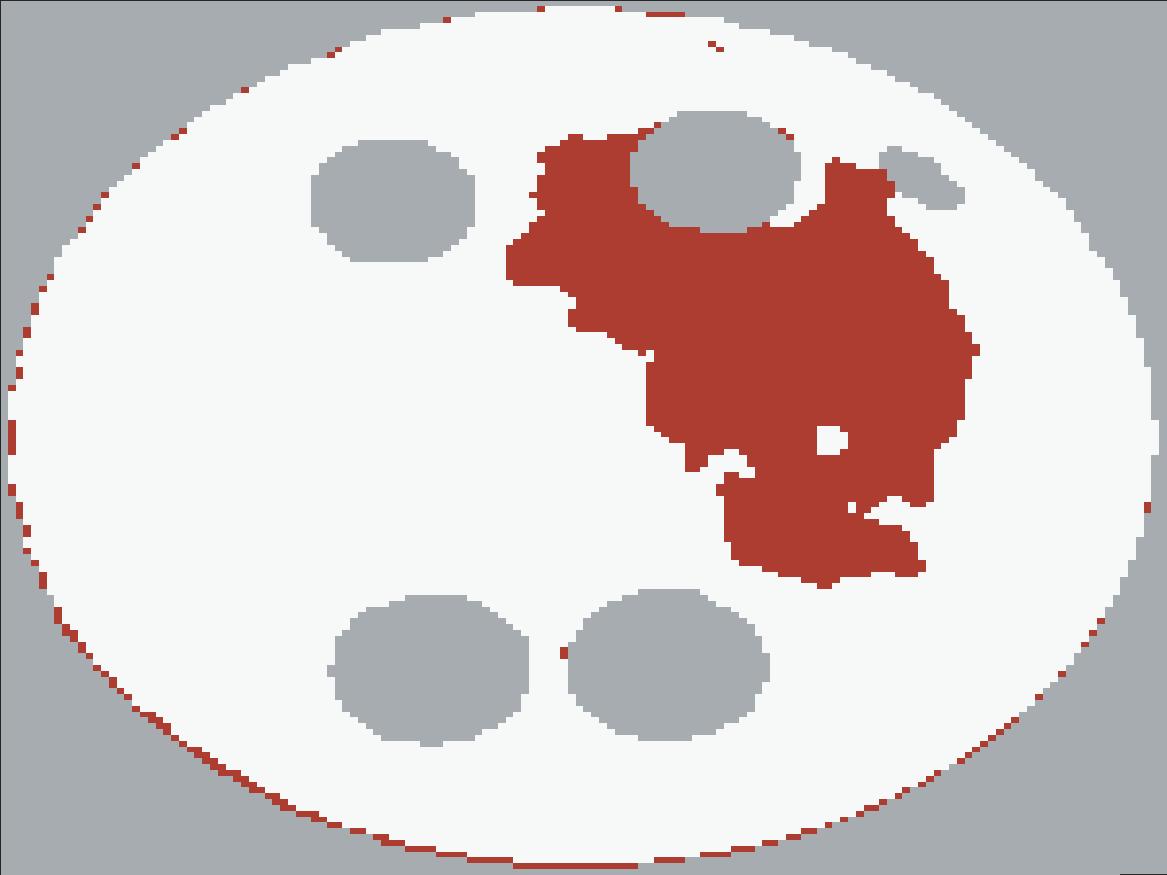

Supplement: Supplementary file 2 [file DataSheet1.ZIP › Dataset/synthetic_37F66.jpg]

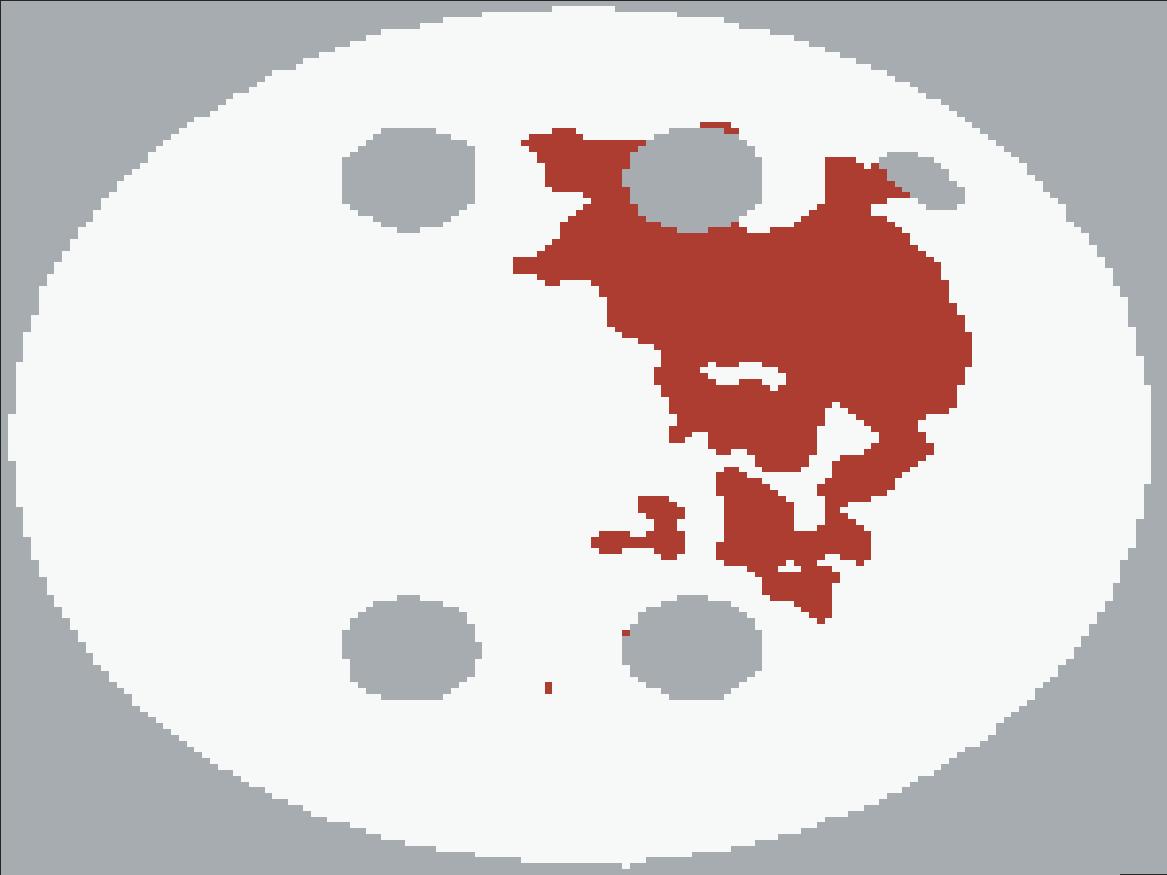

Supplement: Supplementary file 2 [file DataSheet1.ZIP › Dataset/synthetic_38WR2.jpg]

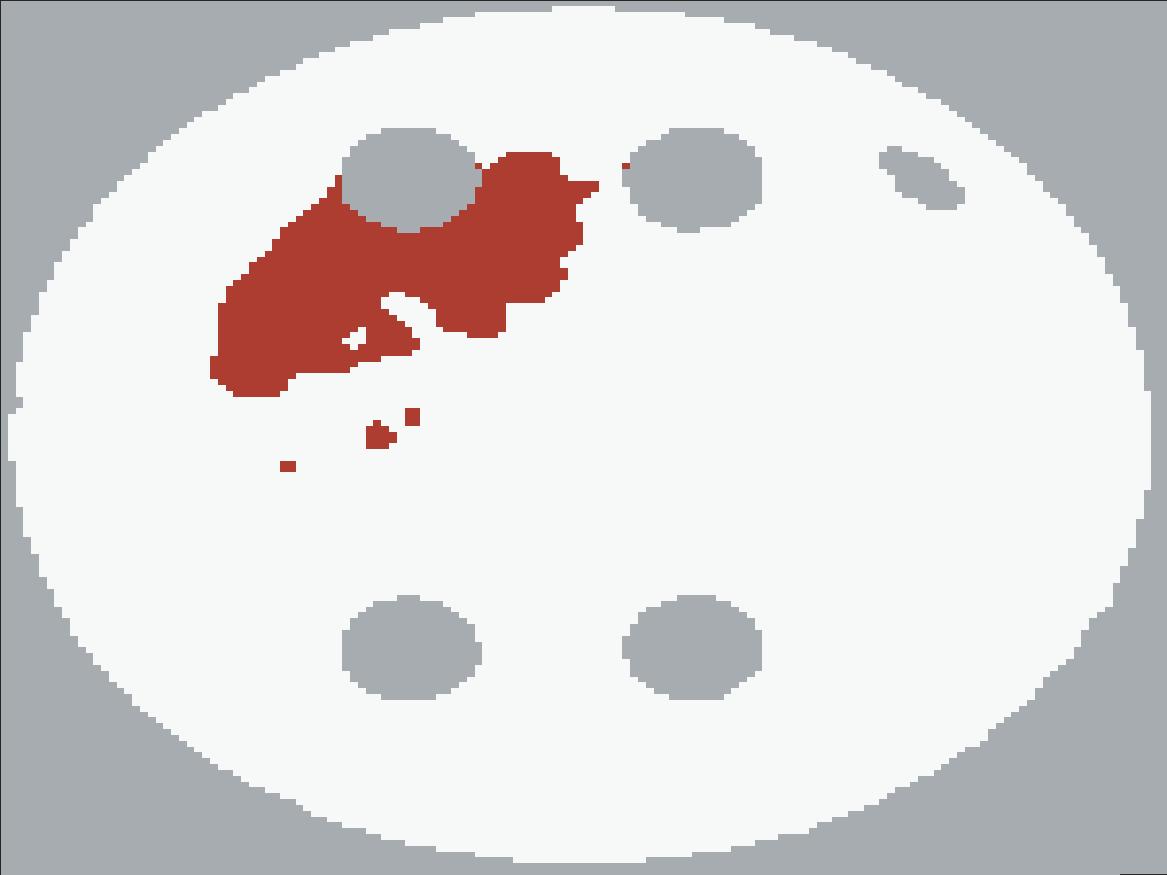

Supplement: Supplementary file 2 [file DataSheet1.ZIP › Dataset/synthetic_39XID.jpg]

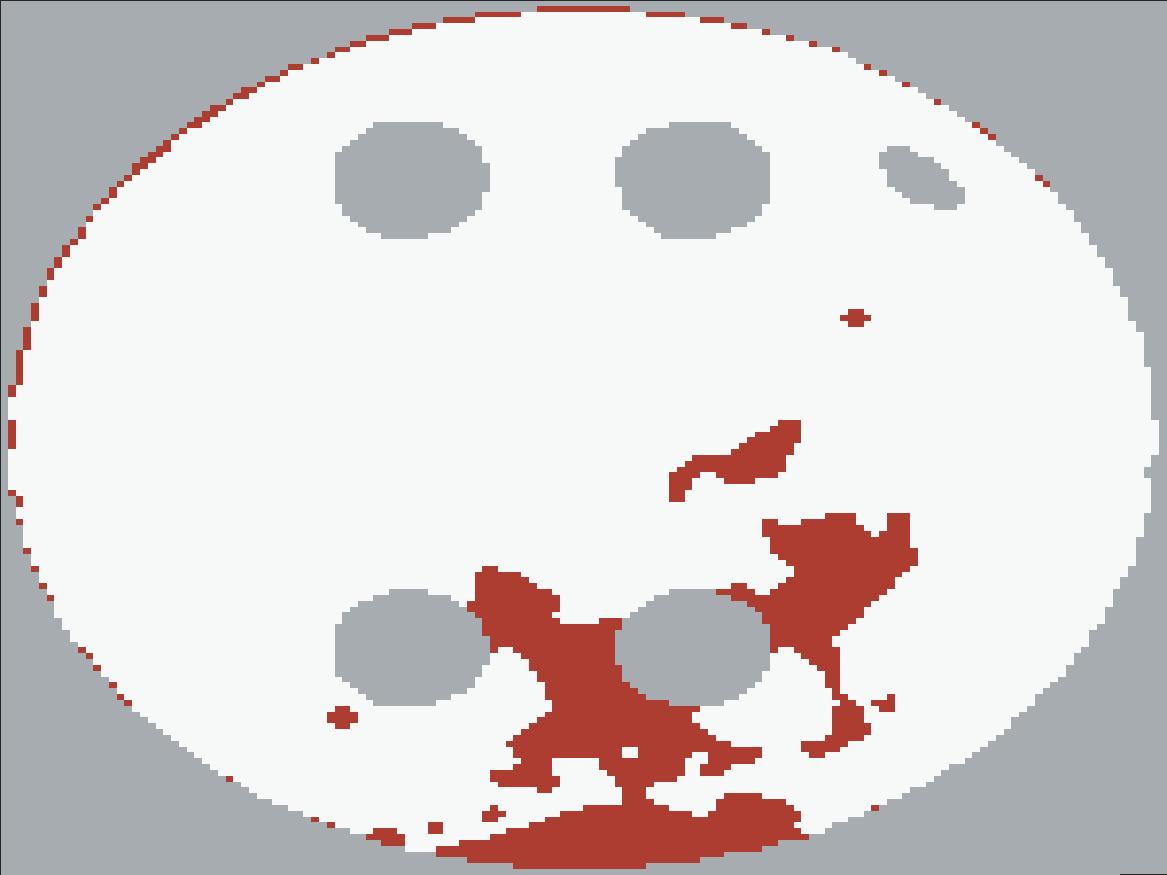

Supplement: Supplementary file 2 [file DataSheet1.ZIP › Dataset/synthetic_3CZOW.jpg]

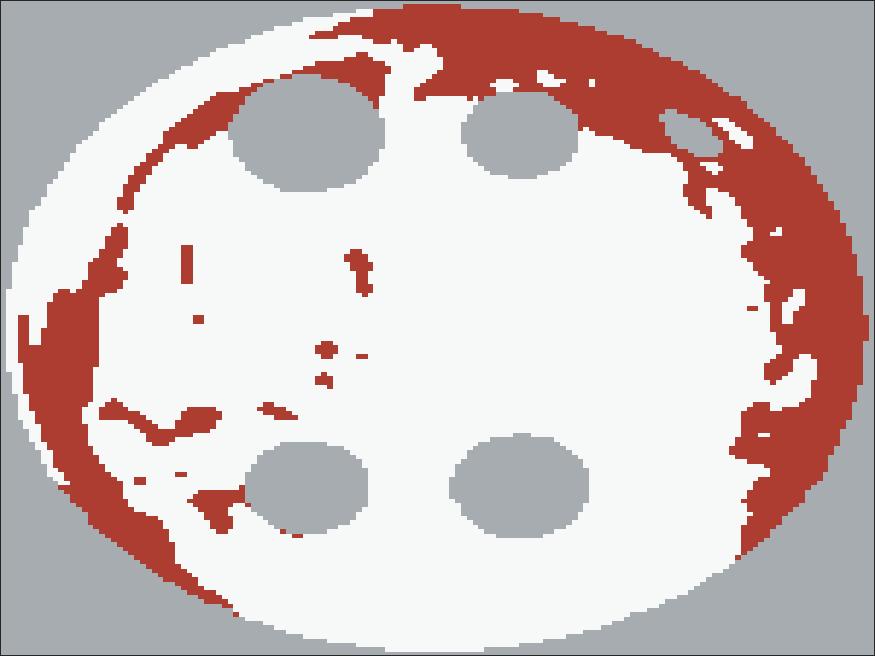

Supplement: Supplementary file 2 [file DataSheet1.ZIP › Dataset/synthetic_3JIND.jpg]

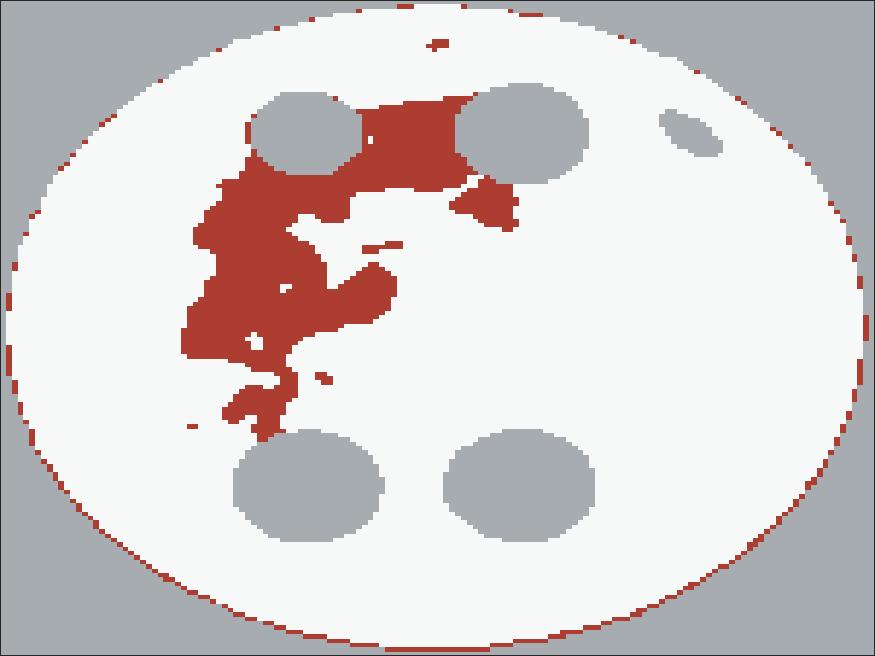

Supplement: Supplementary file 2 [file DataSheet1.ZIP › Dataset/synthetic_3MSVL.jpg]

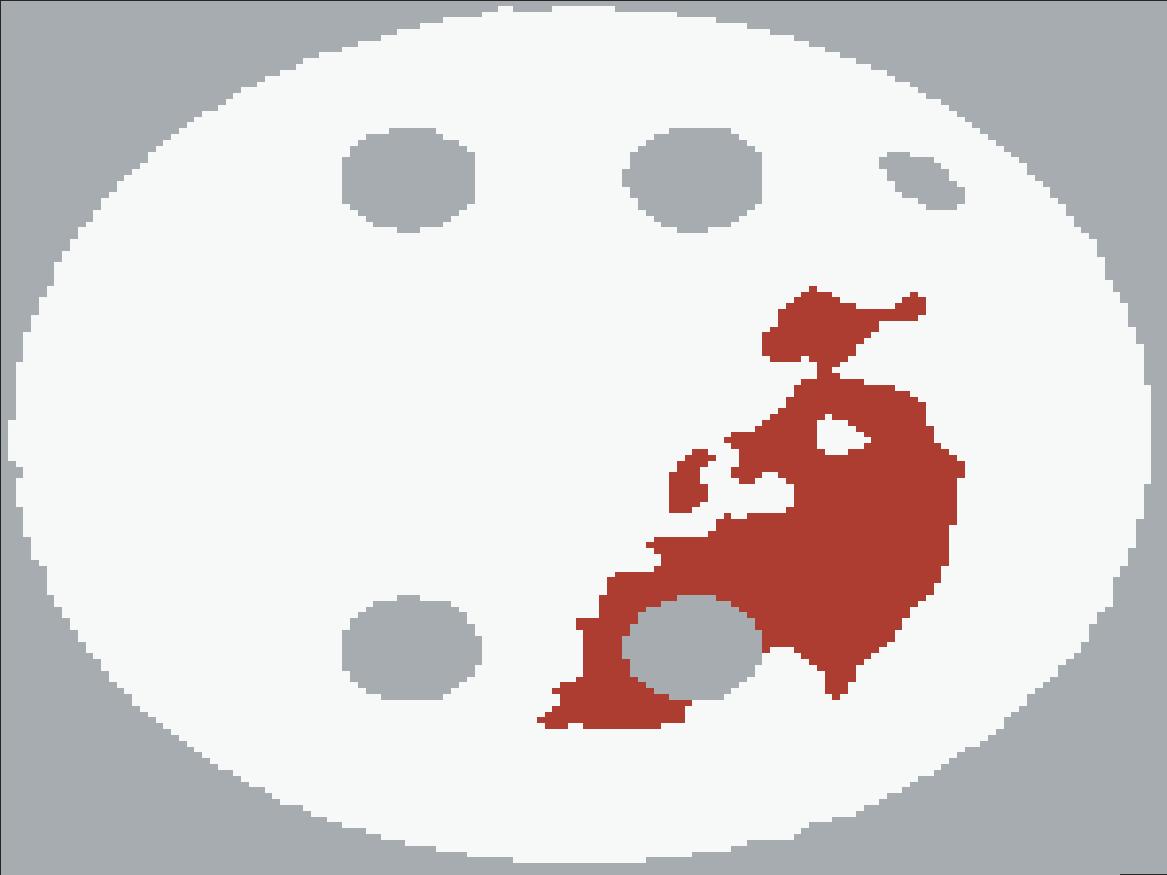

Supplement: Supplementary file 2 [file DataSheet1.ZIP › Dataset/synthetic_3WN1S.jpg]

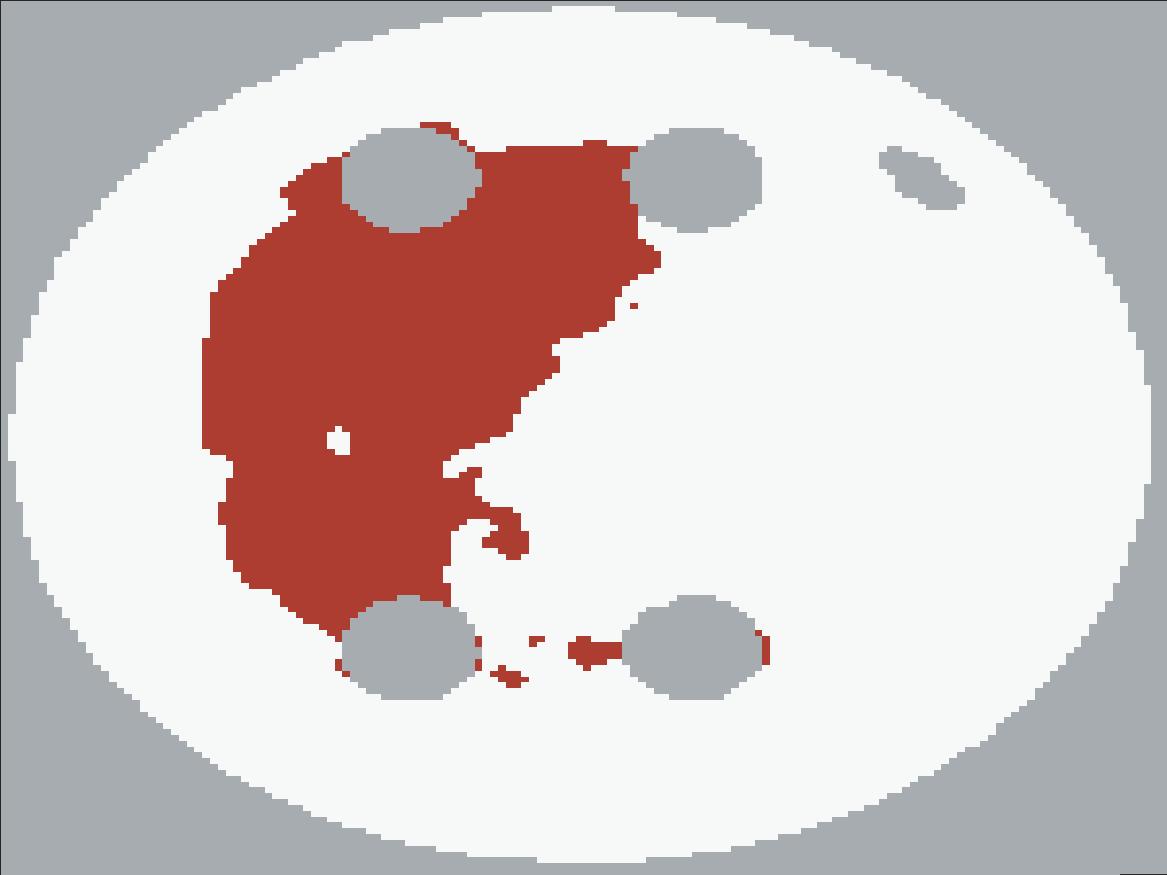

Supplement: Supplementary file 2 [file DataSheet1.ZIP › Dataset/synthetic_4UD13.jpg]

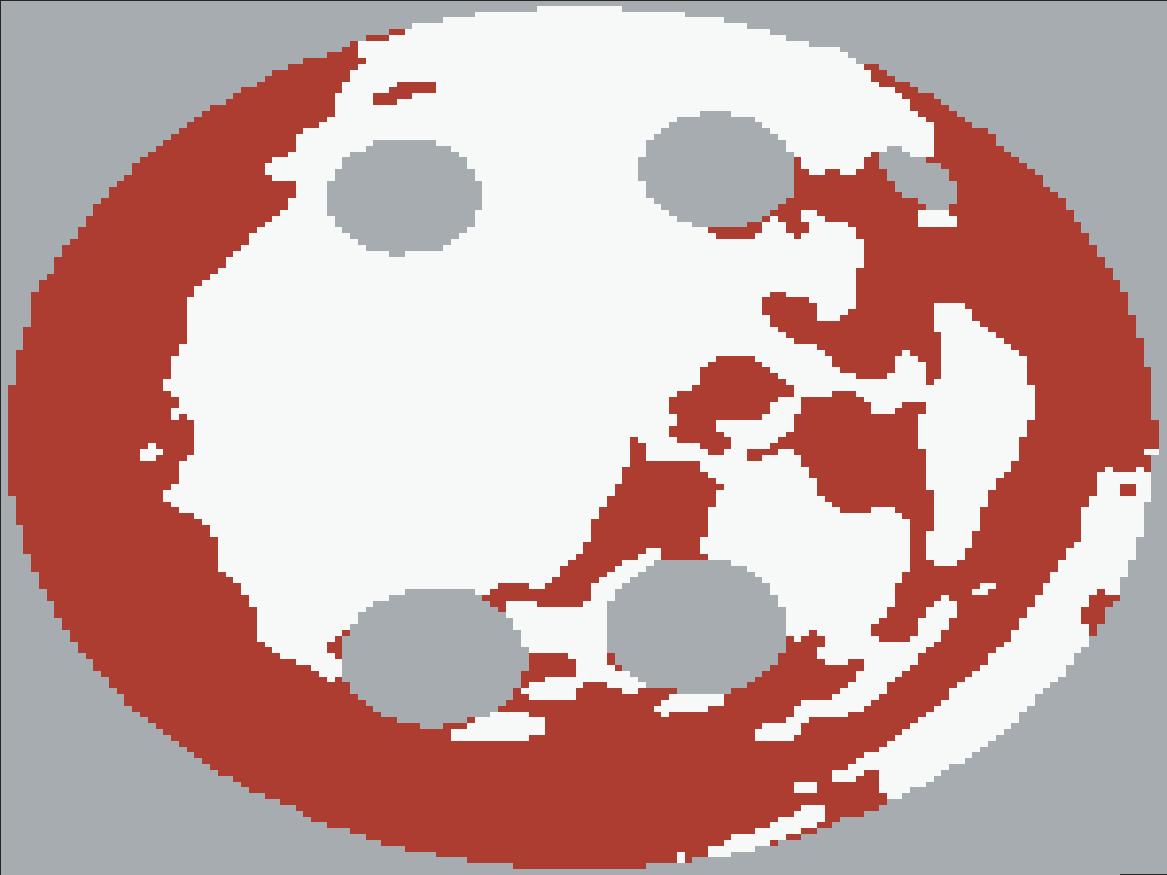

Supplement: Supplementary file 2 [file DataSheet1.ZIP › Dataset/synthetic_4YIGO.jpg]

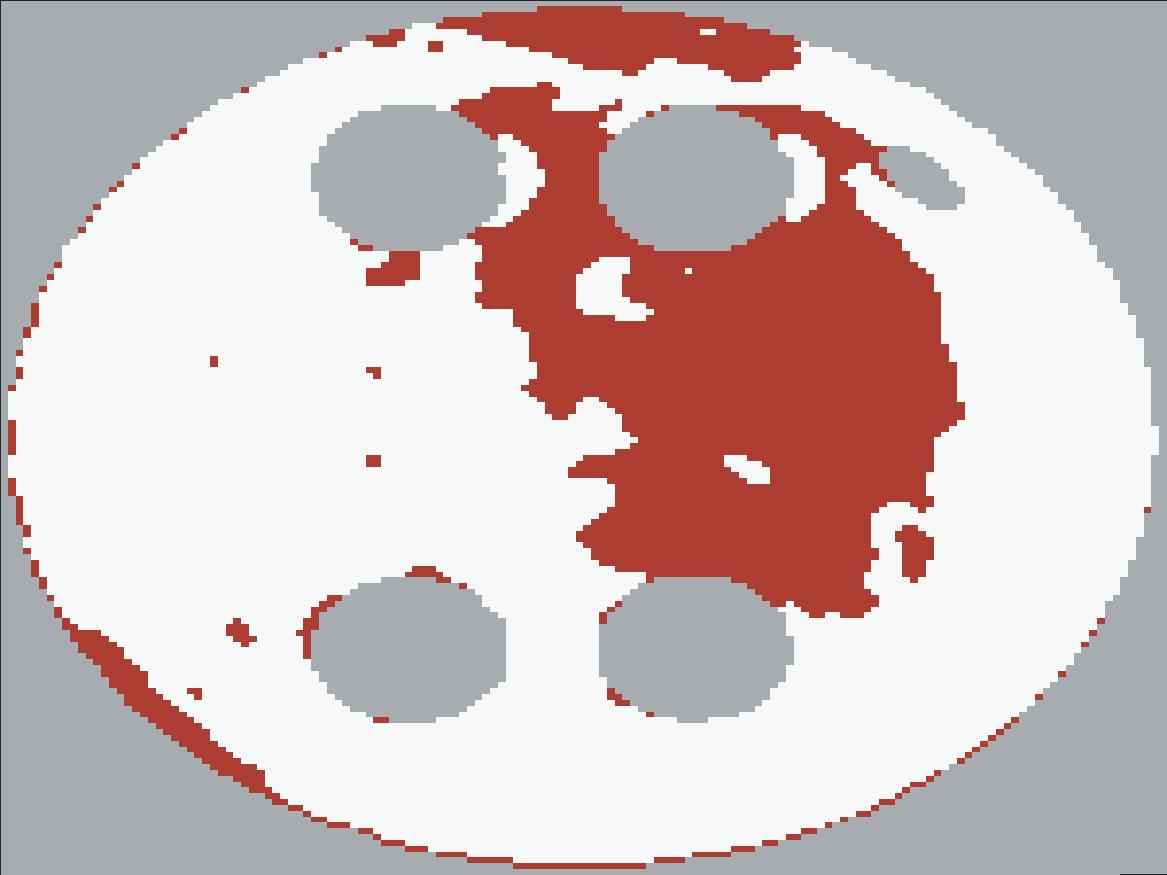

Supplement: Supplementary file 2 [file DataSheet1.ZIP › Dataset/synthetic_57POG.jpg]

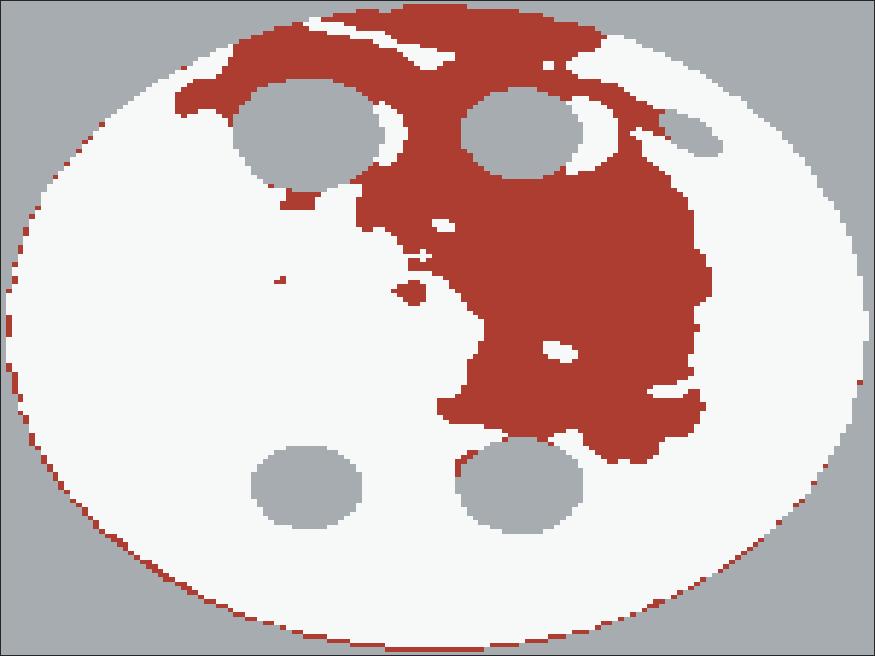

Supplement: Supplementary file 2 [file DataSheet1.ZIP › Dataset/synthetic_5EIJQ.jpg]

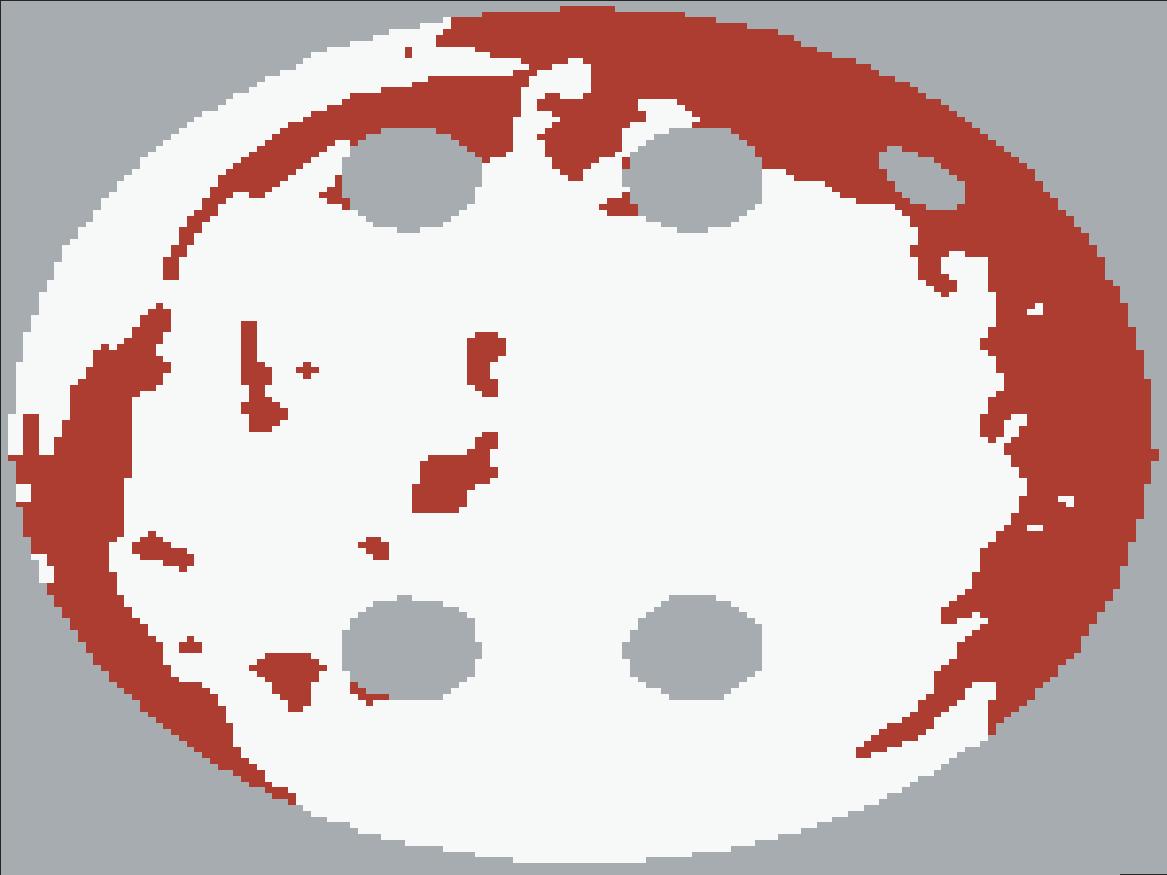

Supplement: Supplementary file 2 [file DataSheet1.ZIP › Dataset/synthetic_5GN5Y.jpg]

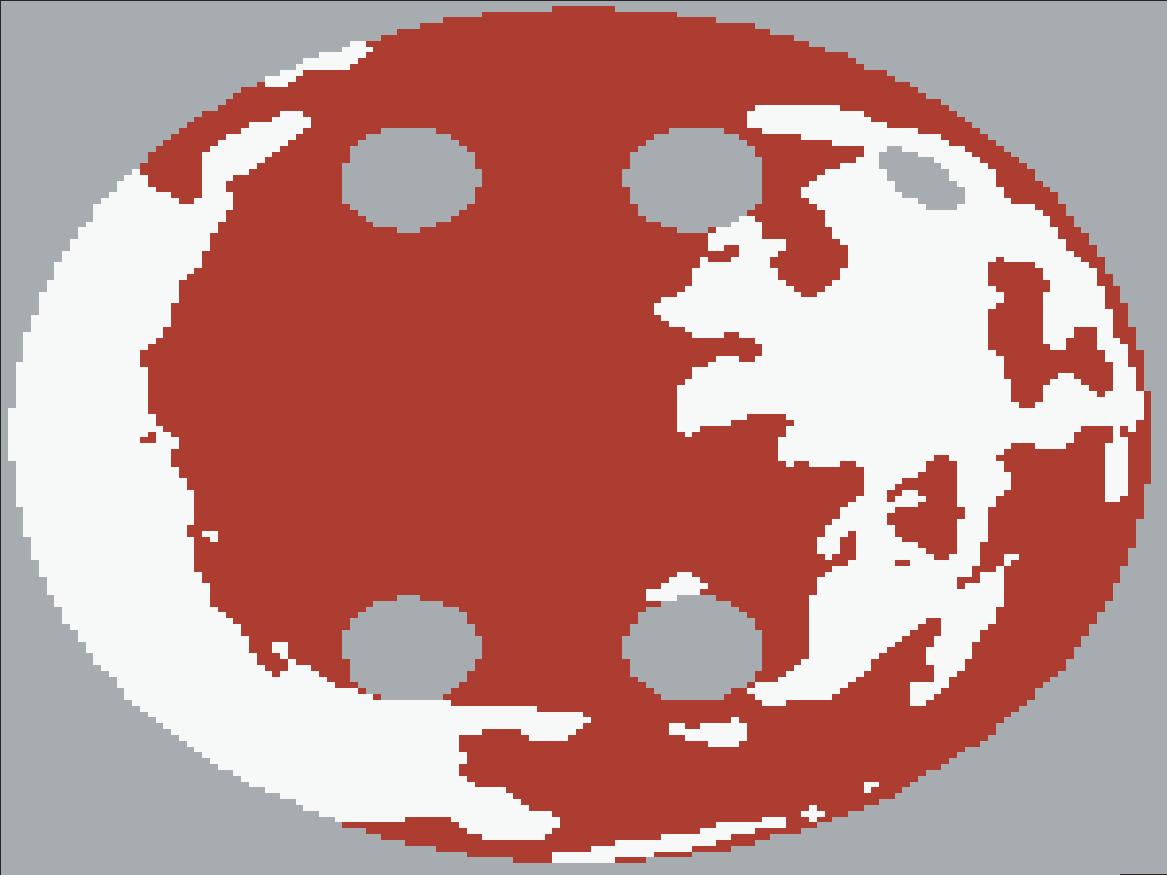

Supplement: Supplementary file 2 [file DataSheet1.ZIP › Dataset/synthetic_5HZ0F.jpg]

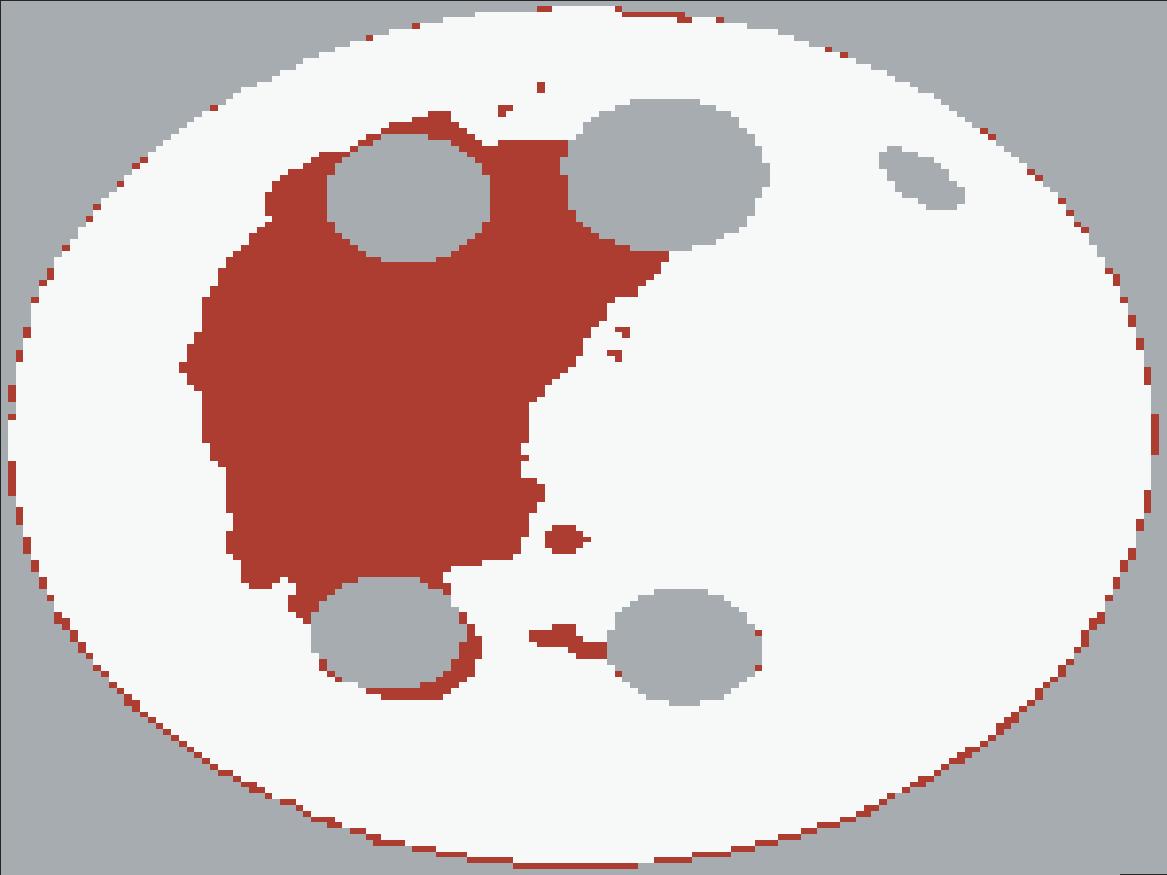

Supplement: Supplementary file 2 [file DataSheet1.ZIP › Dataset/synthetic_5MKAC.jpg]

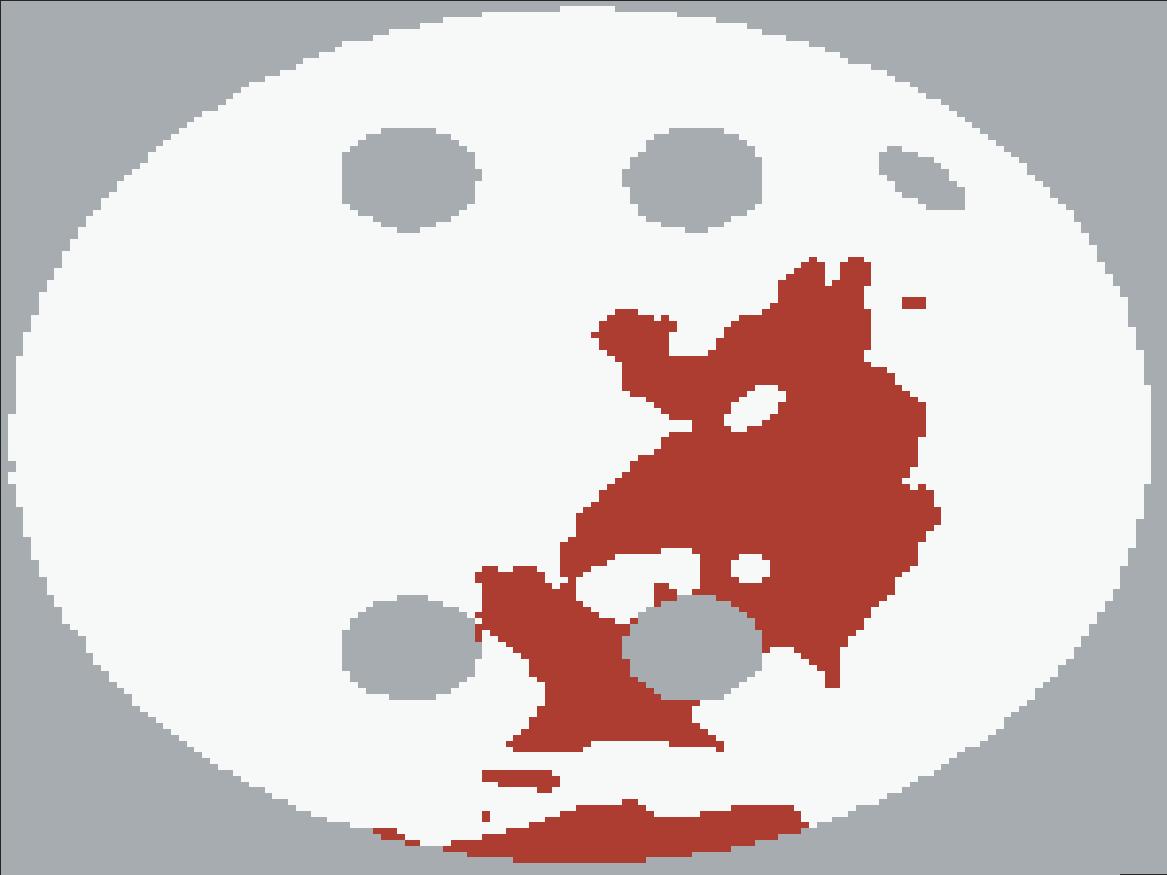

Supplement: Supplementary file 2 [file DataSheet1.ZIP › Dataset/synthetic_5P5KY.jpg]

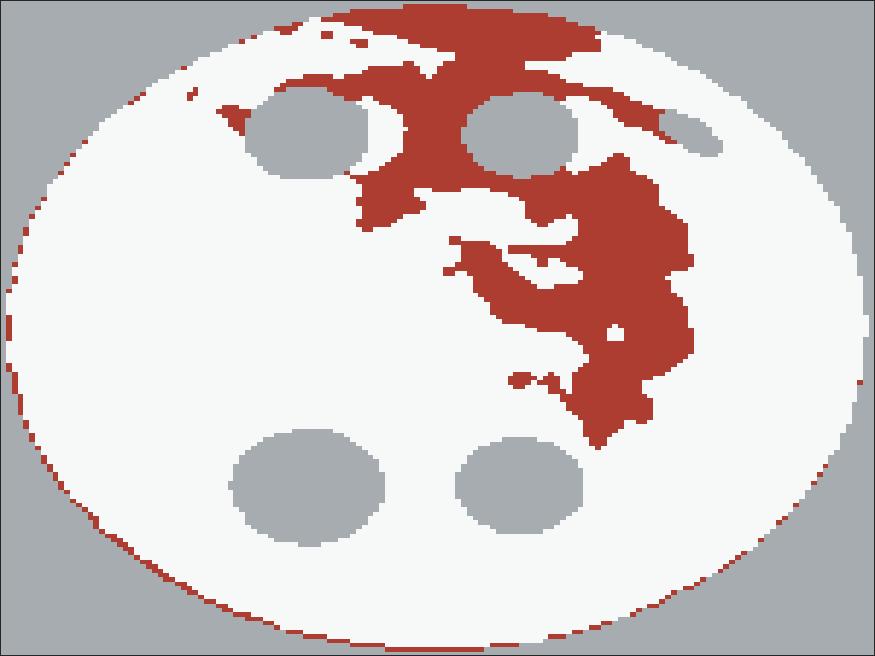

Supplement: Supplementary file 2 [file DataSheet1.ZIP › Dataset/synthetic_5PB0U.jpg]

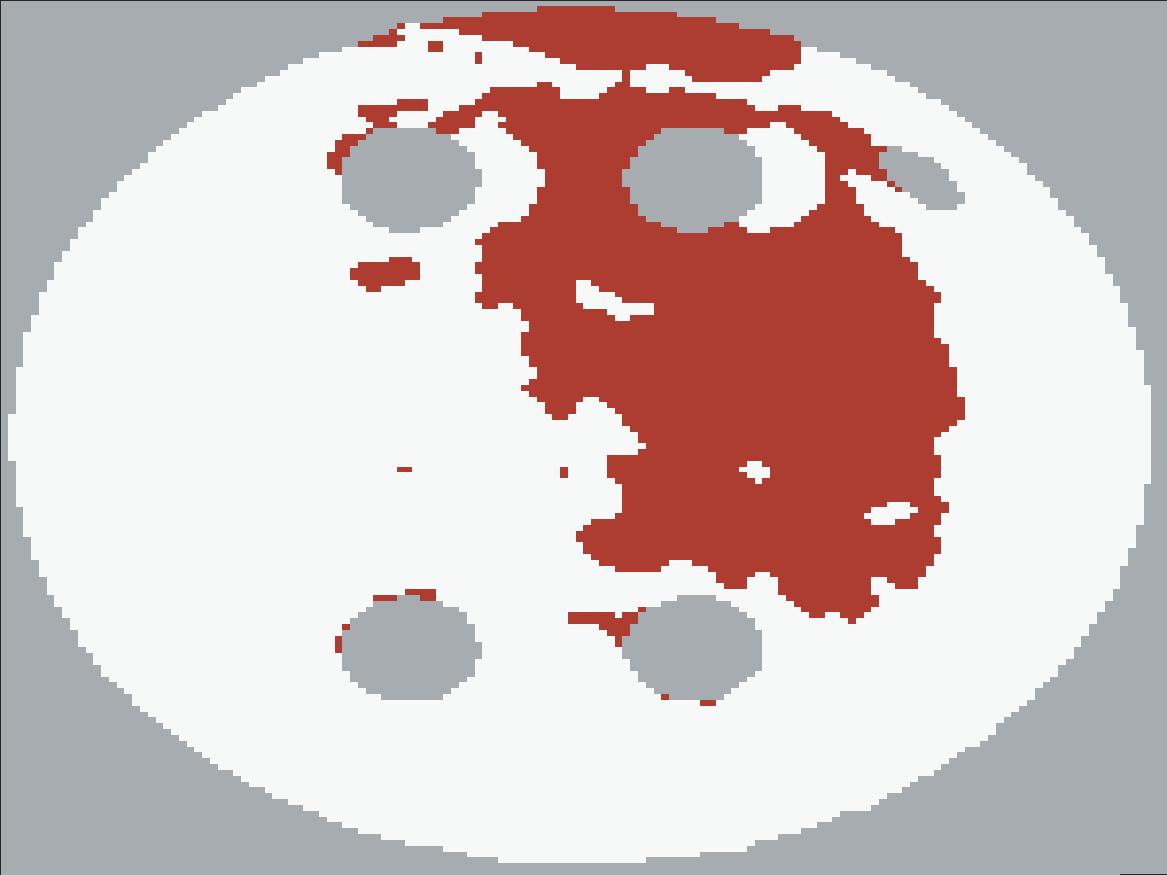

Supplement: Supplementary file 2 [file DataSheet1.ZIP › Dataset/synthetic_5TSCQ.jpg]

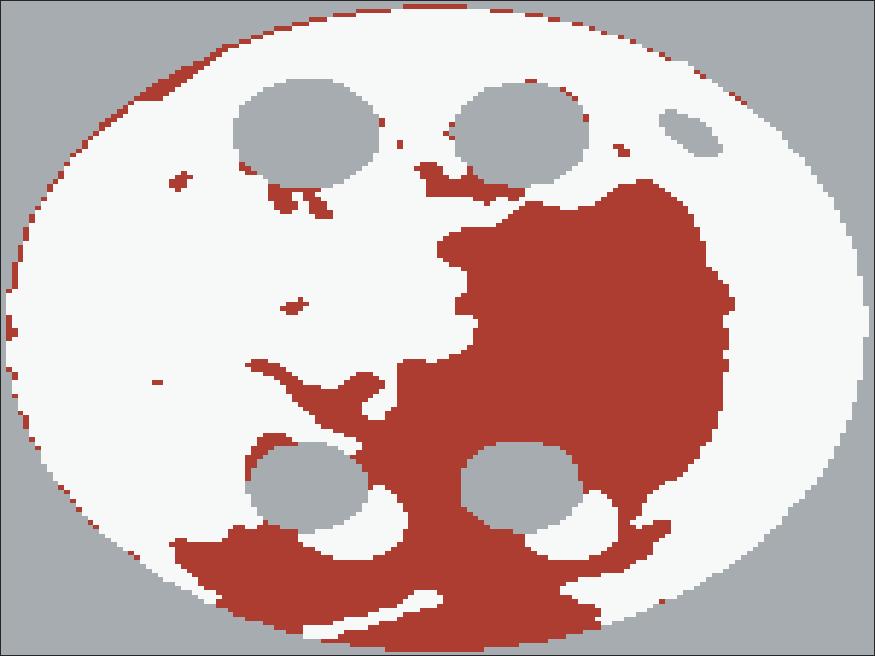

Supplement: Supplementary file 2 [file DataSheet1.ZIP › Dataset/synthetic_60ASA.jpg]

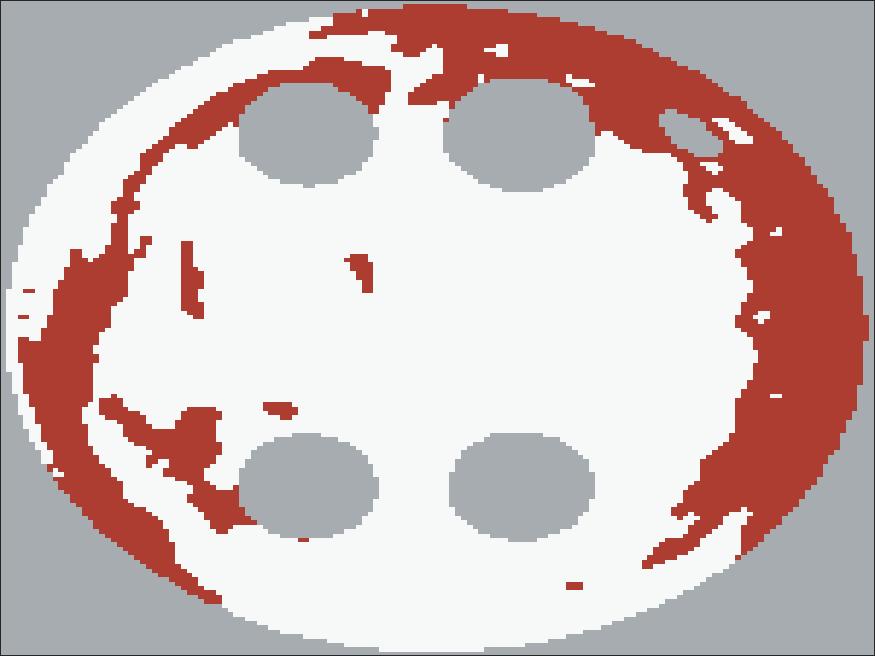

Supplement: Supplementary file 2 [file DataSheet1.ZIP › Dataset/synthetic_6BA7Q.jpg]

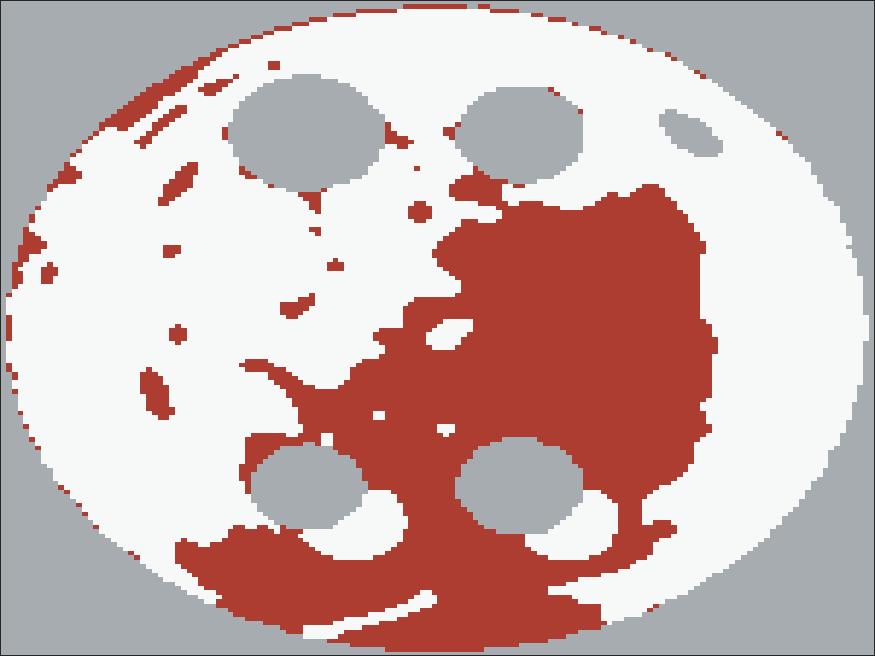

Supplement: Supplementary file 2 [file DataSheet1.ZIP › Dataset/synthetic_6BCBI.jpg]

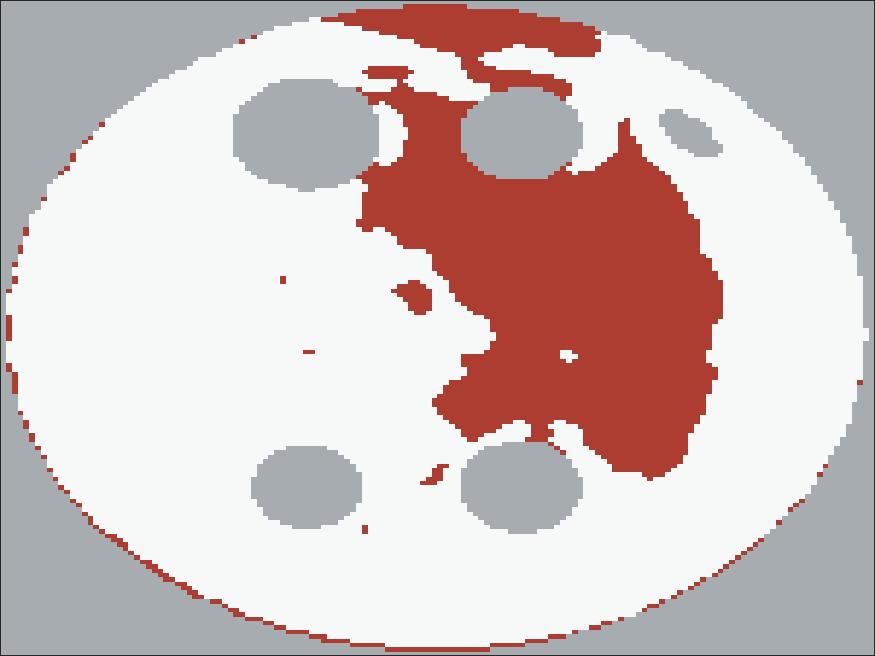

Supplement: Supplementary file 2 [file DataSheet1.ZIP › Dataset/synthetic_6R7JC.jpg]

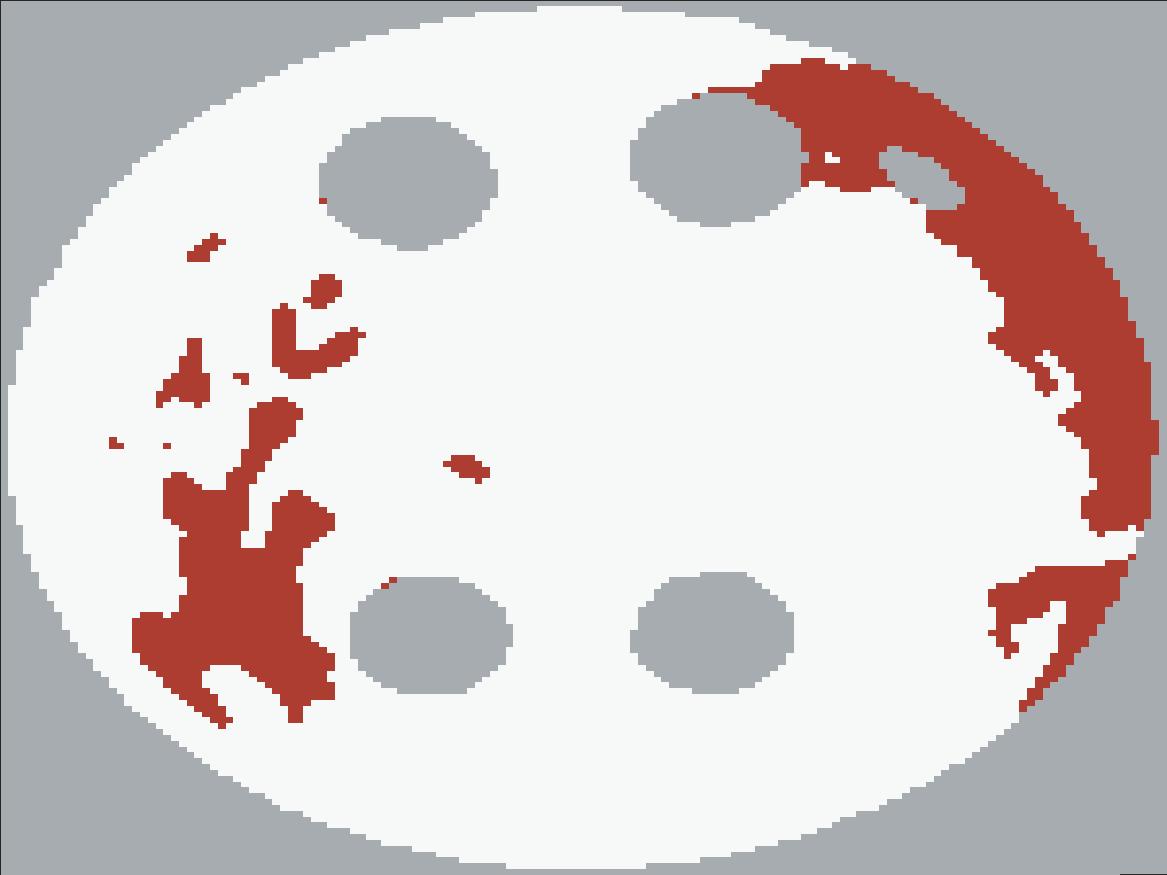

Supplement: Supplementary file 2 [file DataSheet1.ZIP › Dataset/synthetic_6WIDD.jpg]

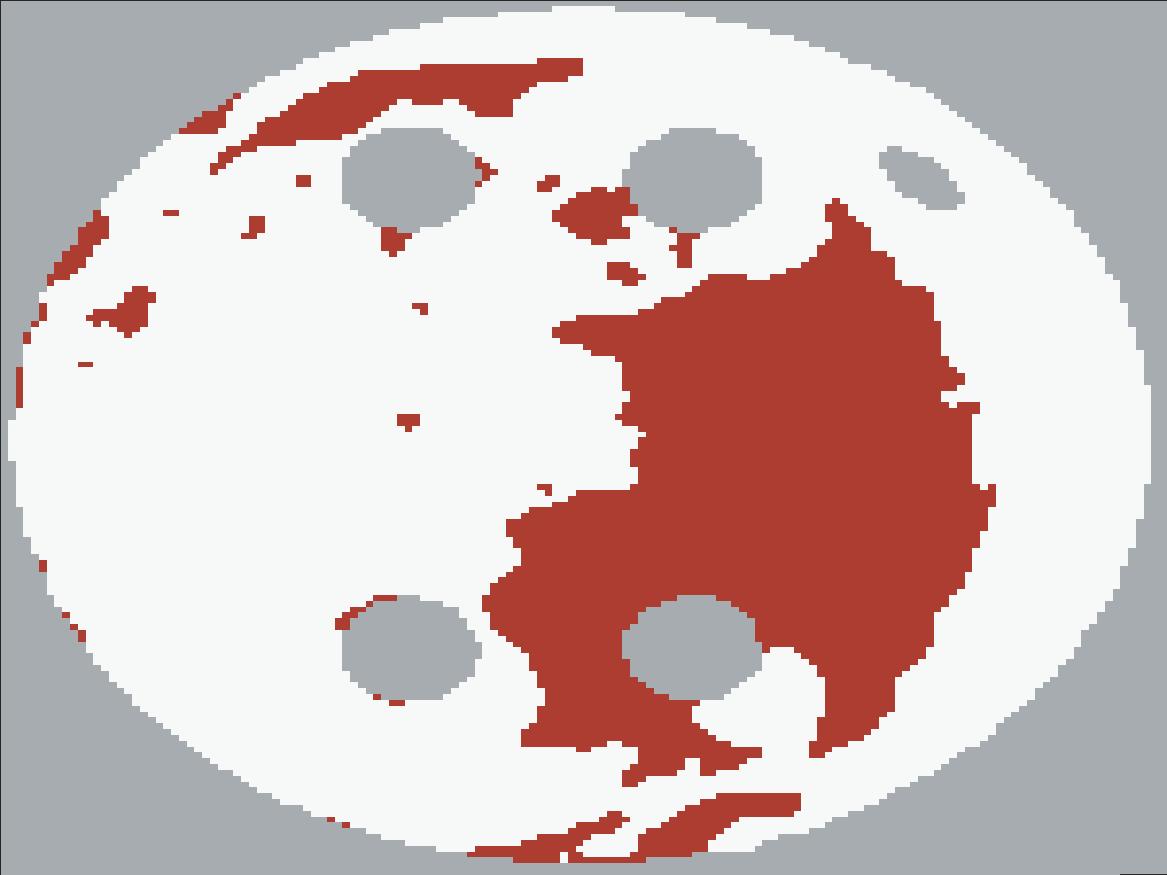

Supplement: Supplementary file 2 [file DataSheet1.ZIP › Dataset/synthetic_6X9LJ.jpg]

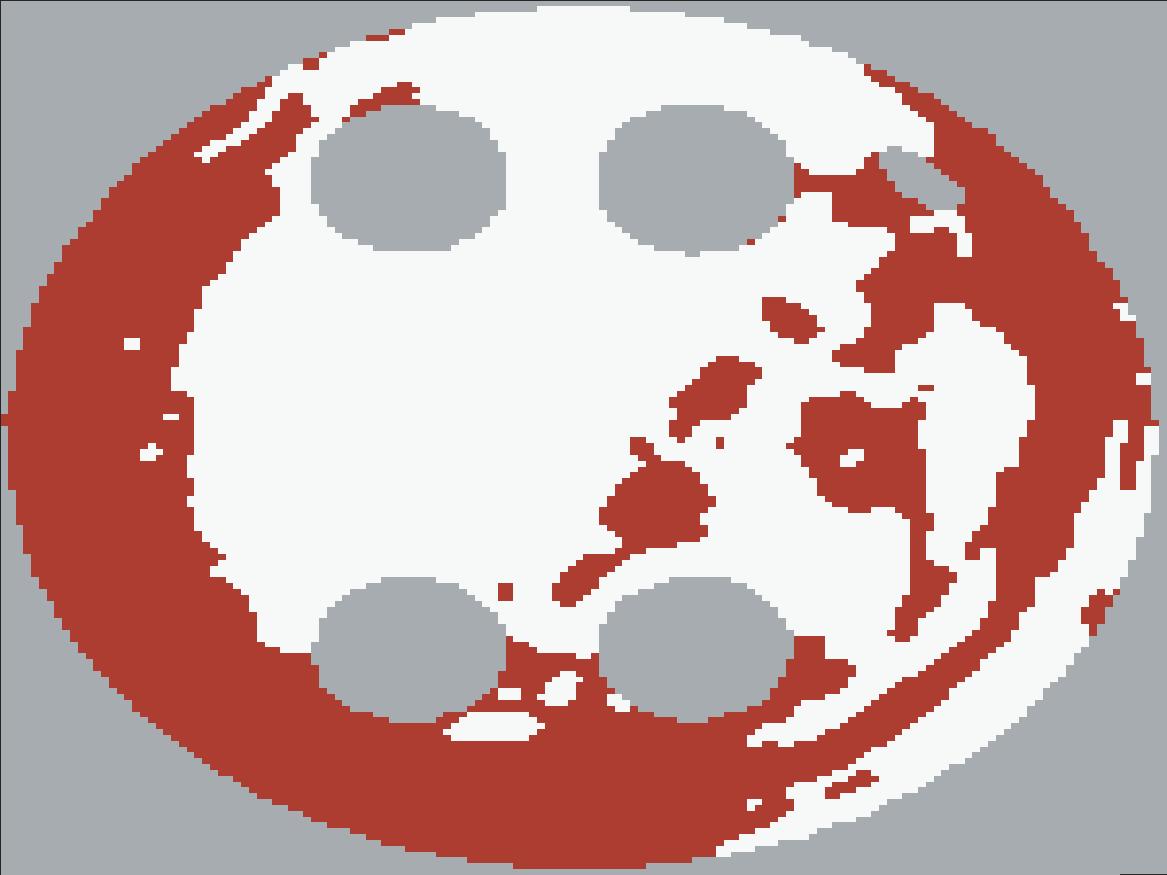

Supplement: Supplementary file 2 [file DataSheet1.ZIP › Dataset/synthetic_6YCZS.jpg]

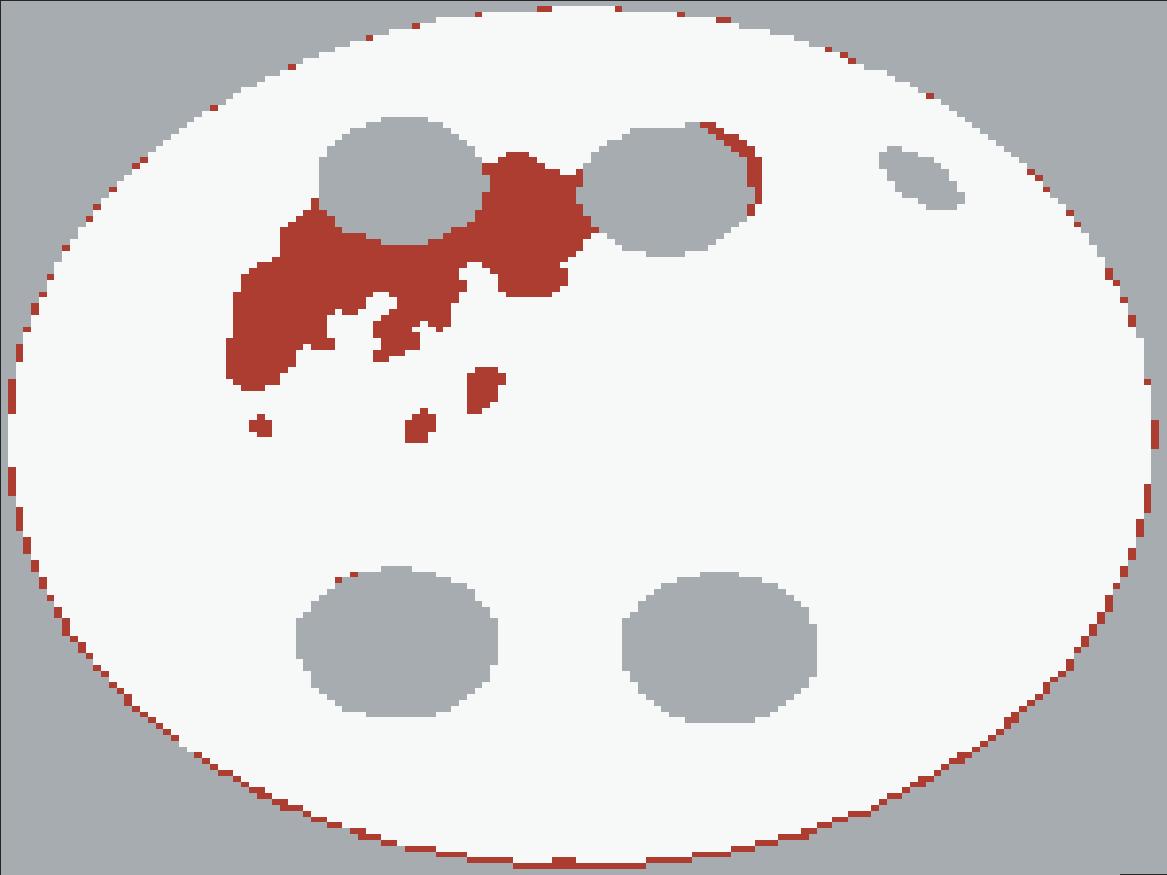

Supplement: Supplementary file 2 [file DataSheet1.ZIP › Dataset/synthetic_70QKD.jpg]

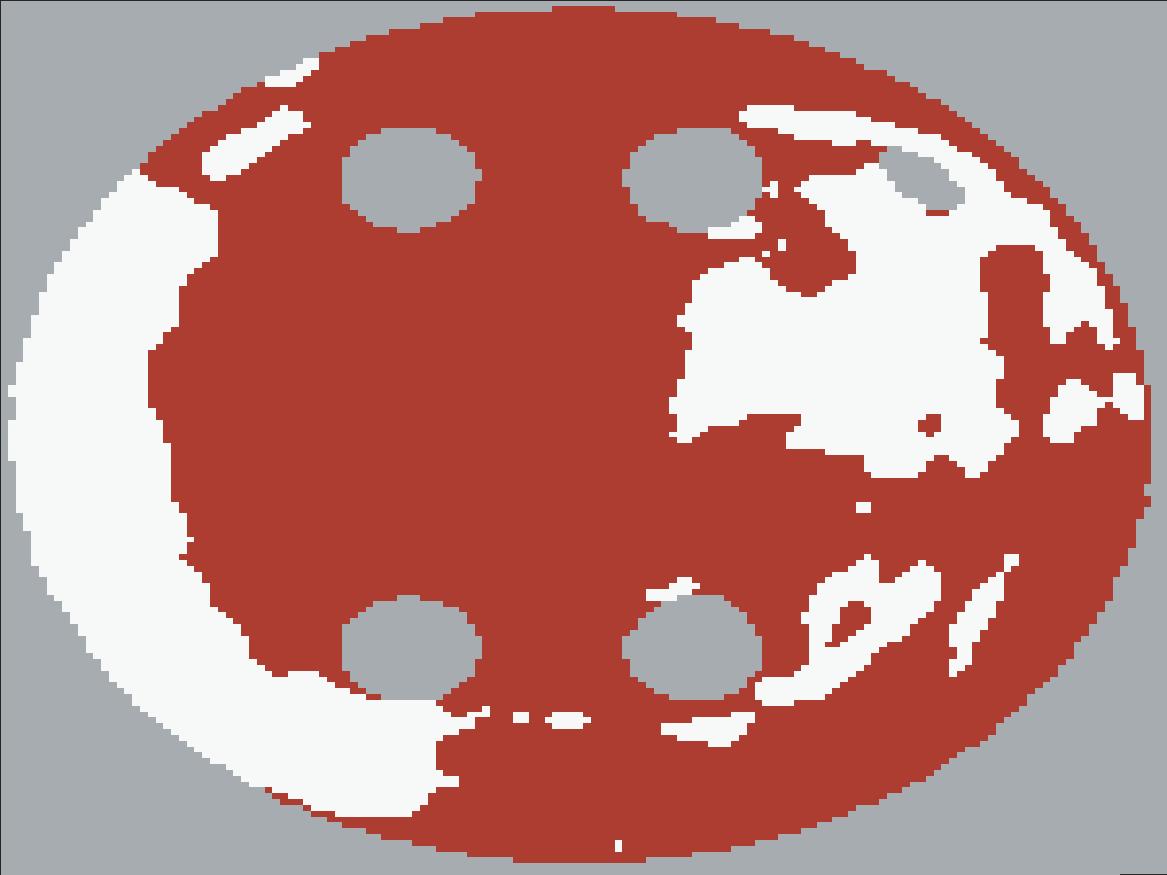

Supplement: Supplementary file 2 [file DataSheet1.ZIP › Dataset/synthetic_752GY.jpg]

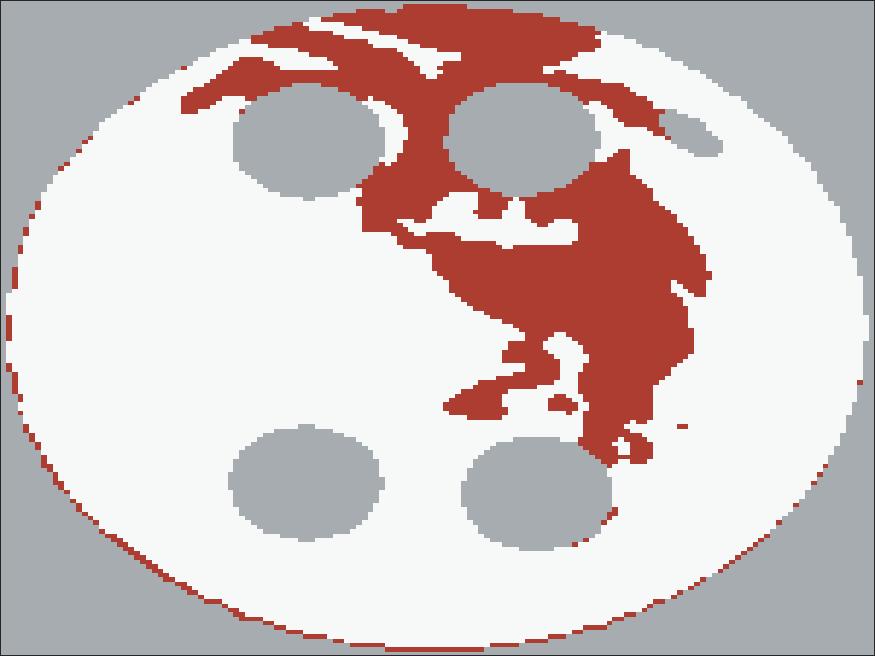

Supplement: Supplementary file 2 [file DataSheet1.ZIP › Dataset/synthetic_79CDD.jpg]

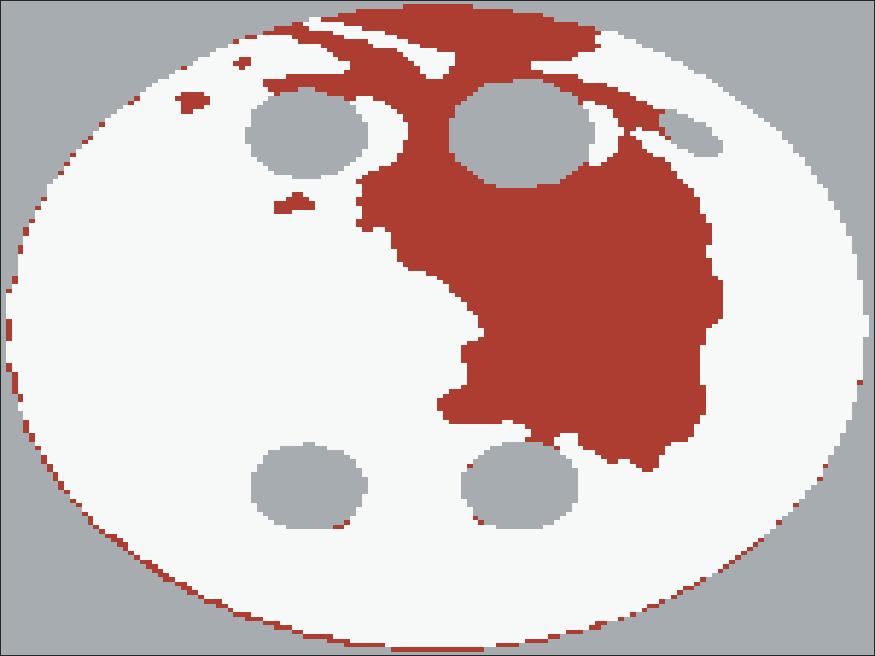

Supplement: Supplementary file 2 [file DataSheet1.ZIP › Dataset/synthetic_7OE45.jpg]

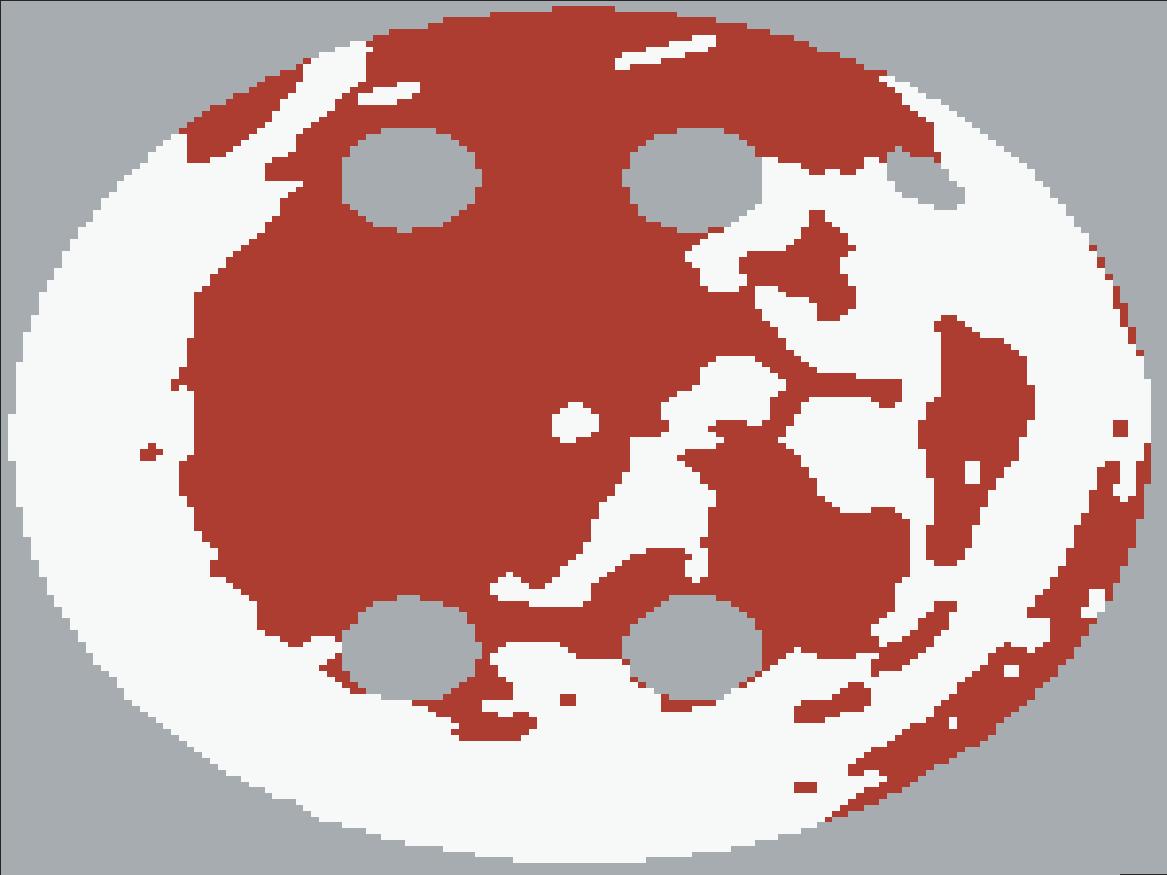

Supplement: Supplementary file 2 [file DataSheet1.ZIP › Dataset/synthetic_822D4.jpg]

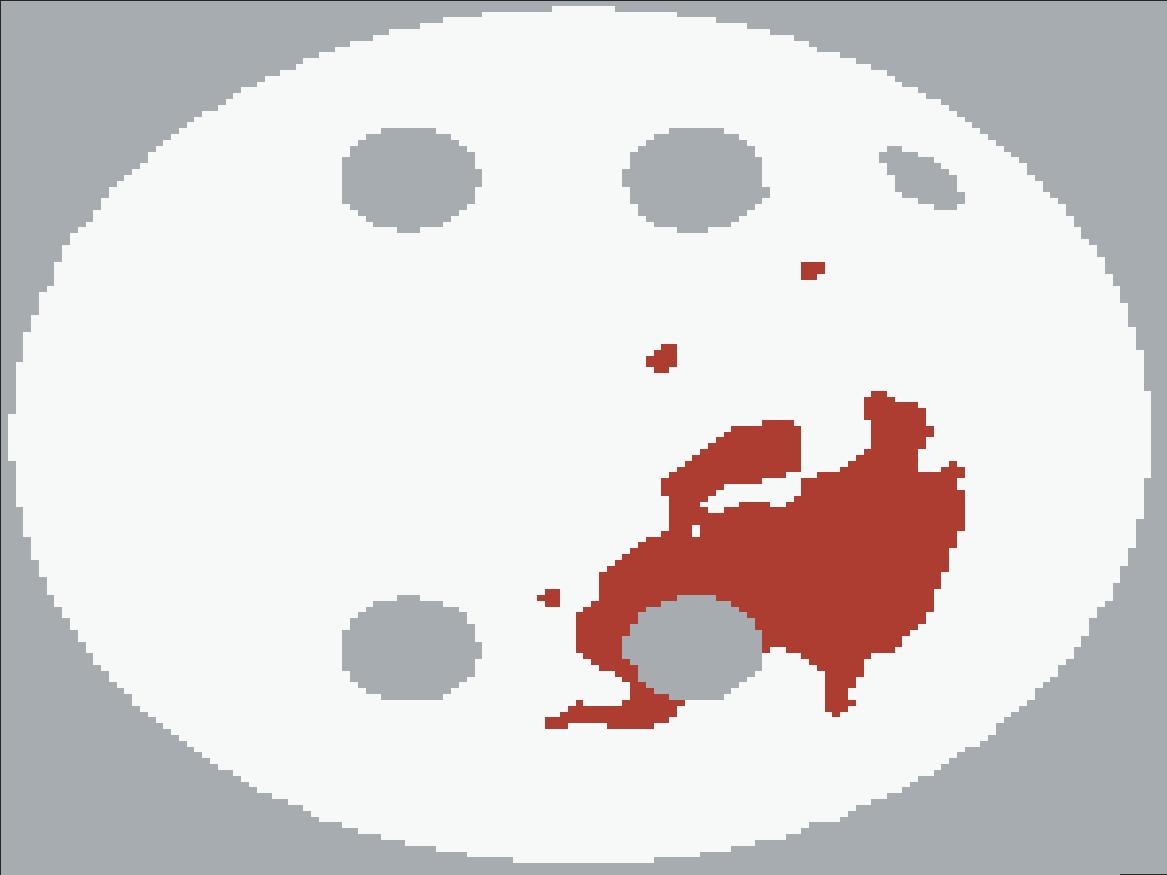

Supplement: Supplementary file 2 [file DataSheet1.ZIP › Dataset/synthetic_83EVF.jpg]

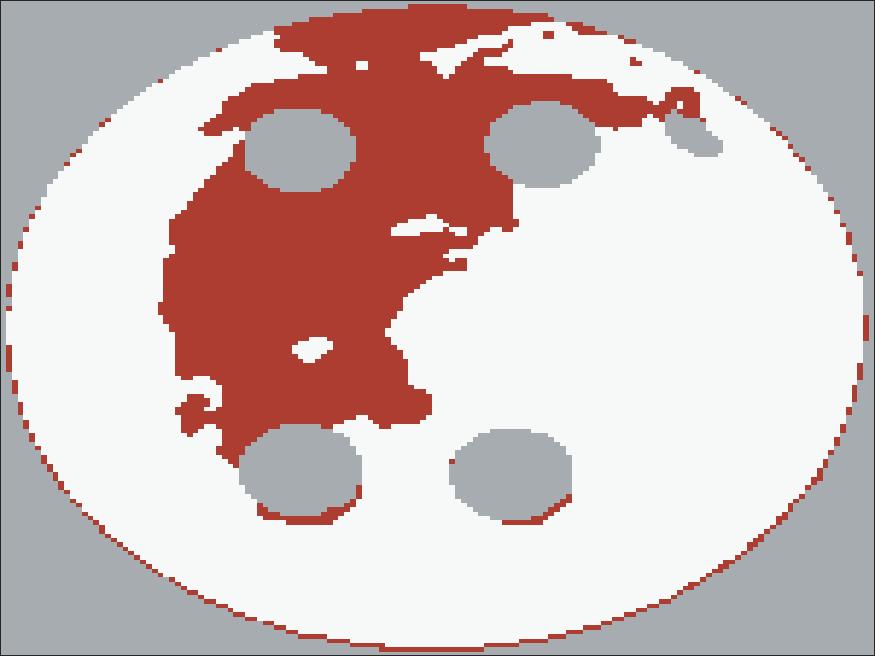

Supplement: Supplementary file 2 [file DataSheet1.ZIP › Dataset/synthetic_87FTV.jpg]

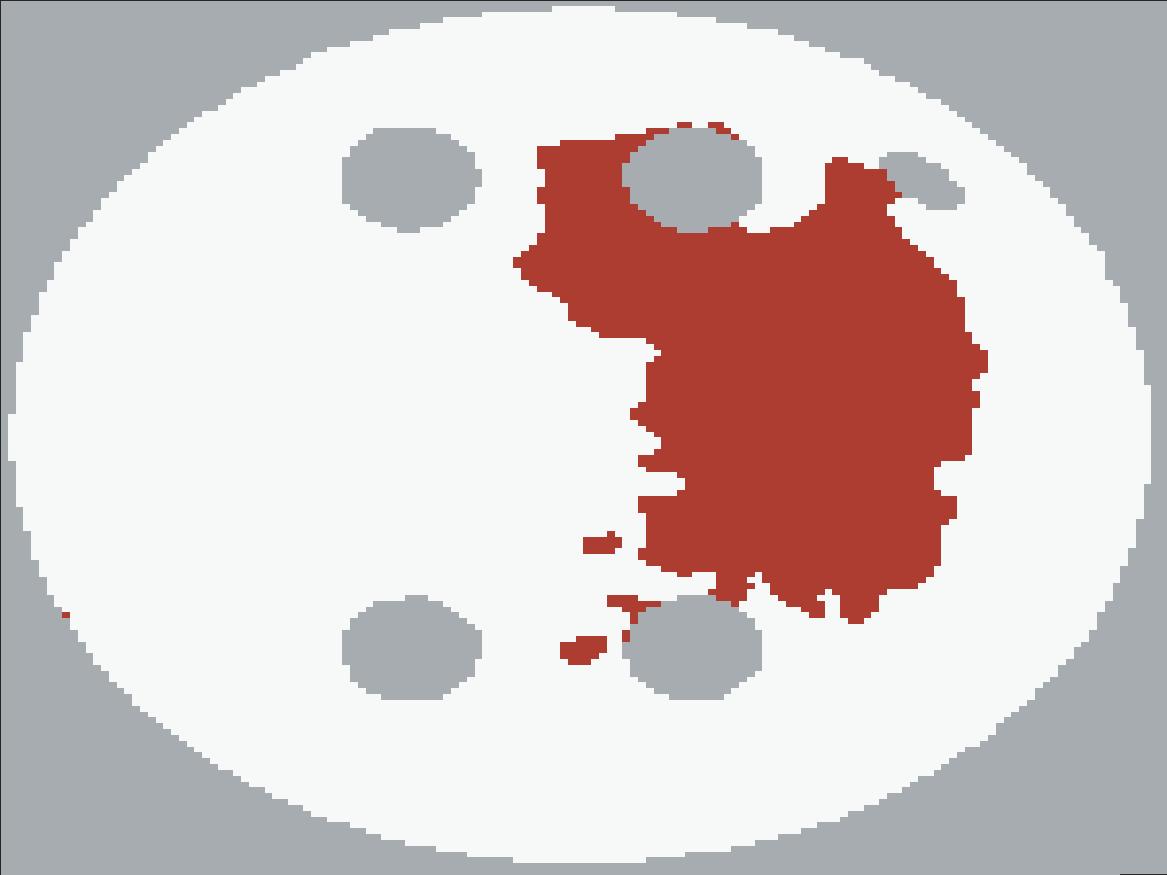

Supplement: Supplementary file 2 [file DataSheet1.ZIP › Dataset/synthetic_8EBF7.jpg]

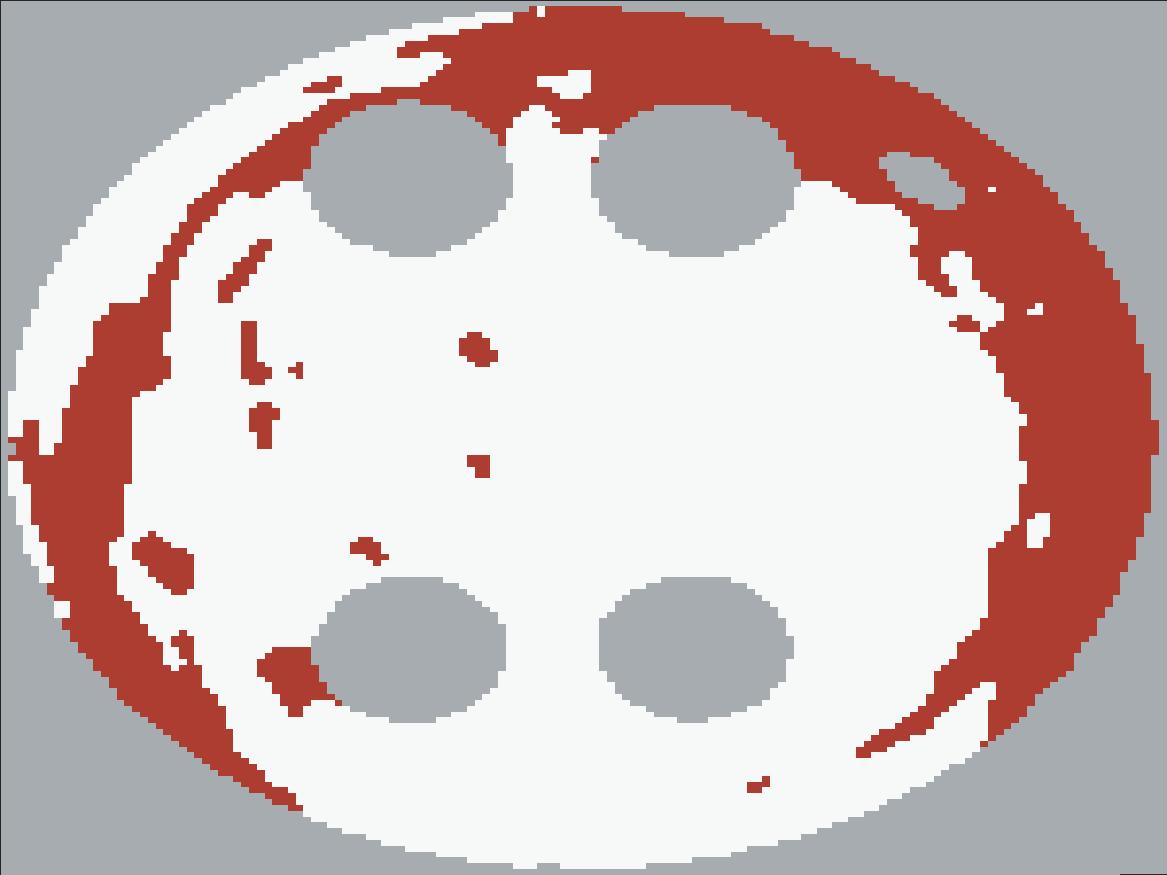

Supplement: Supplementary file 2 [file DataSheet1.ZIP › Dataset/synthetic_8JB0Z.jpg]

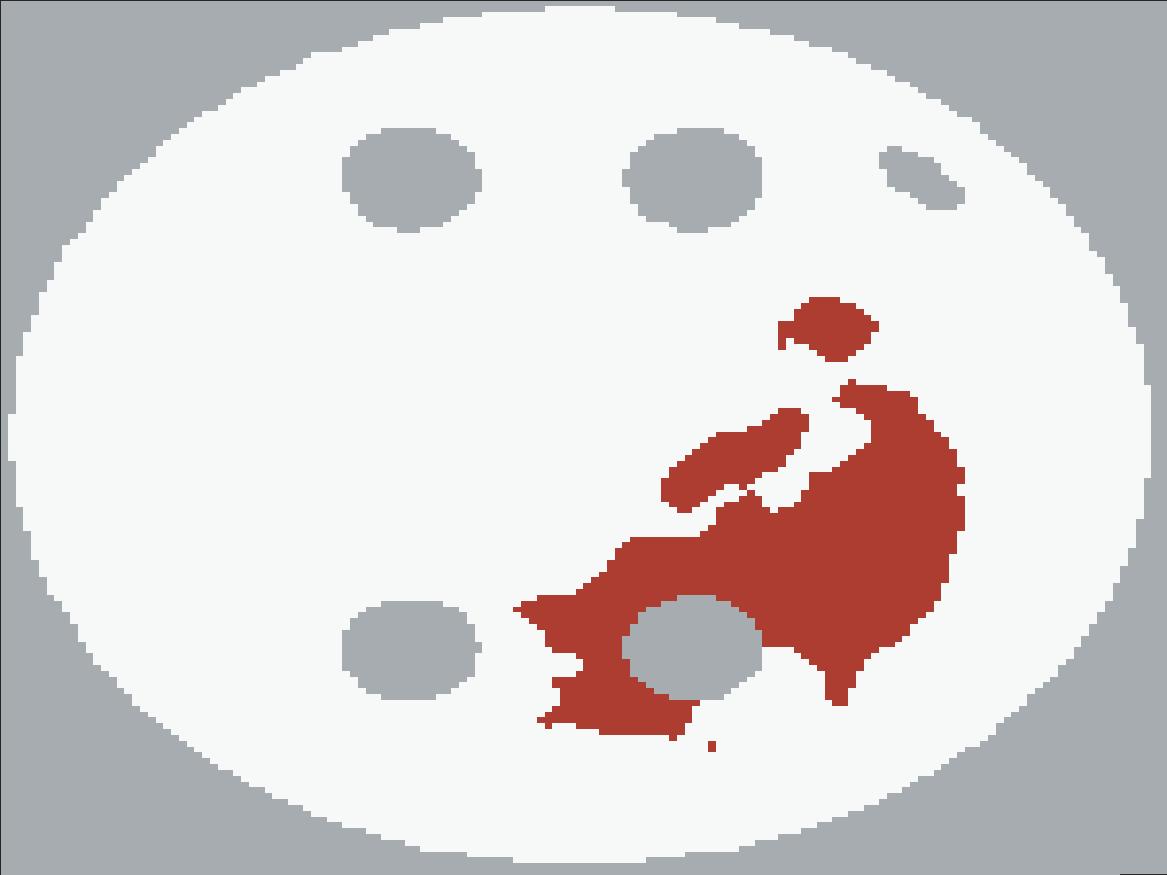

Supplement: Supplementary file 2 [file DataSheet1.ZIP › Dataset/synthetic_8JTMO.jpg]

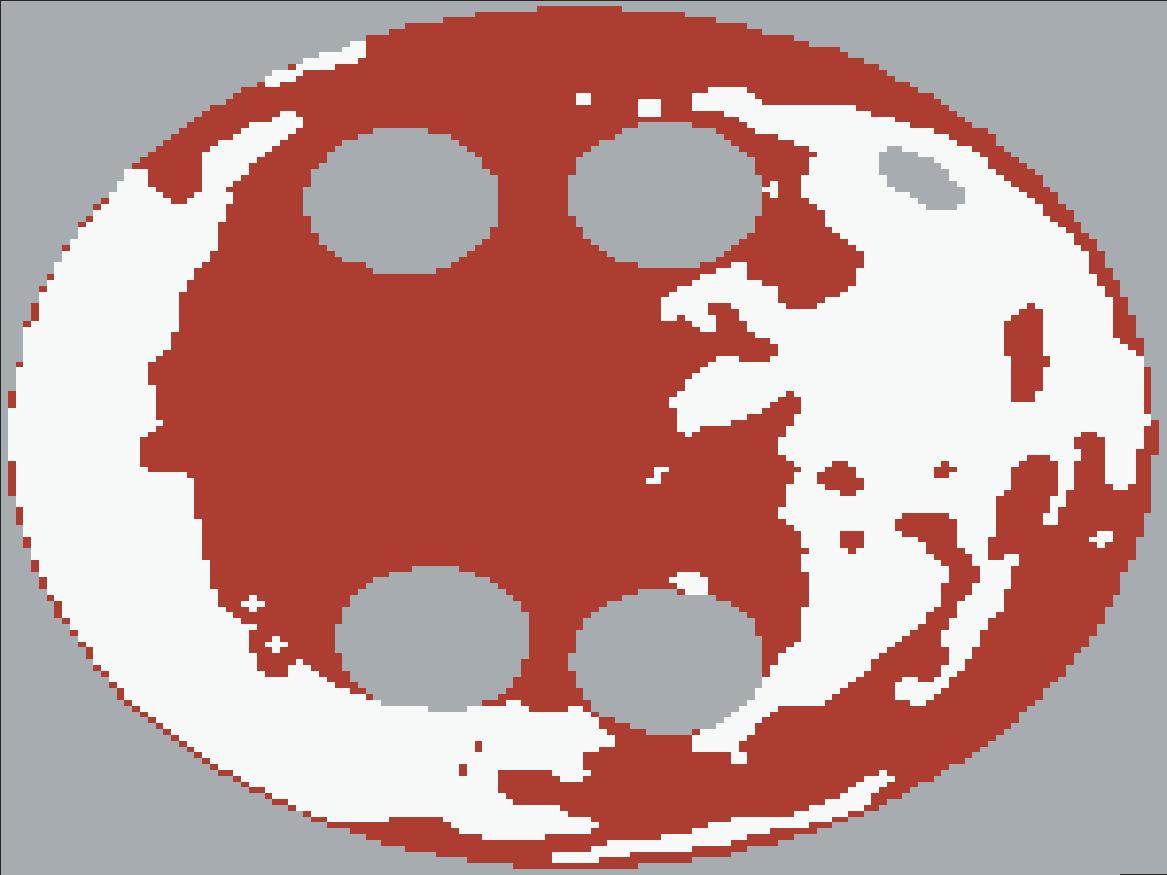

Supplement: Supplementary file 2 [file DataSheet1.ZIP › Dataset/synthetic_8LN7T.jpg]

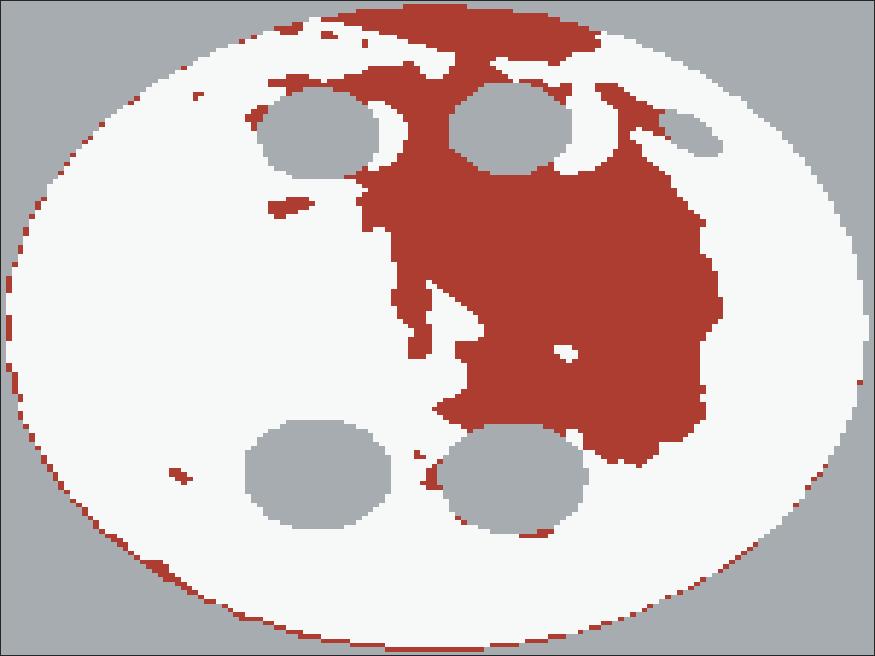

Supplement: Supplementary file 2 [file DataSheet1.ZIP › Dataset/synthetic_8S0QO.jpg]

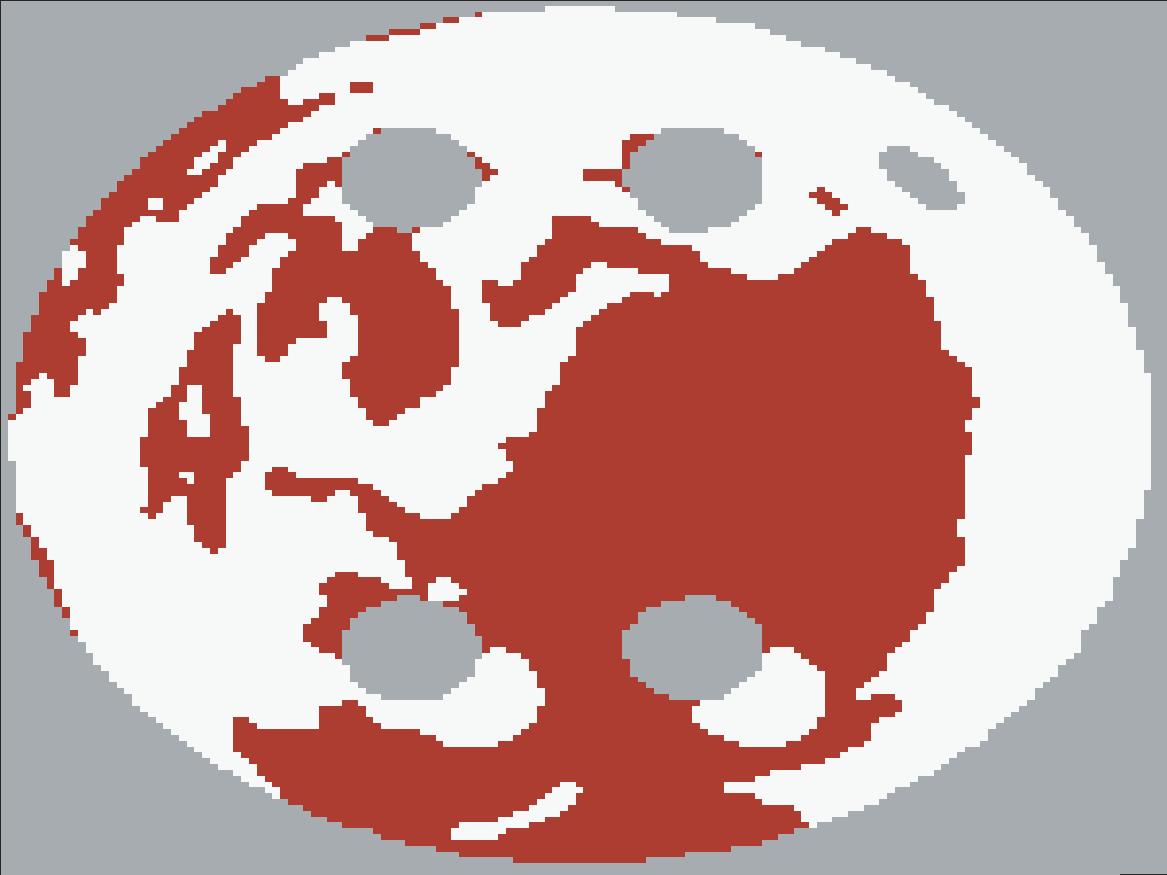

Supplement: Supplementary file 2 [file DataSheet1.ZIP › Dataset/synthetic_8SWI9.jpg]

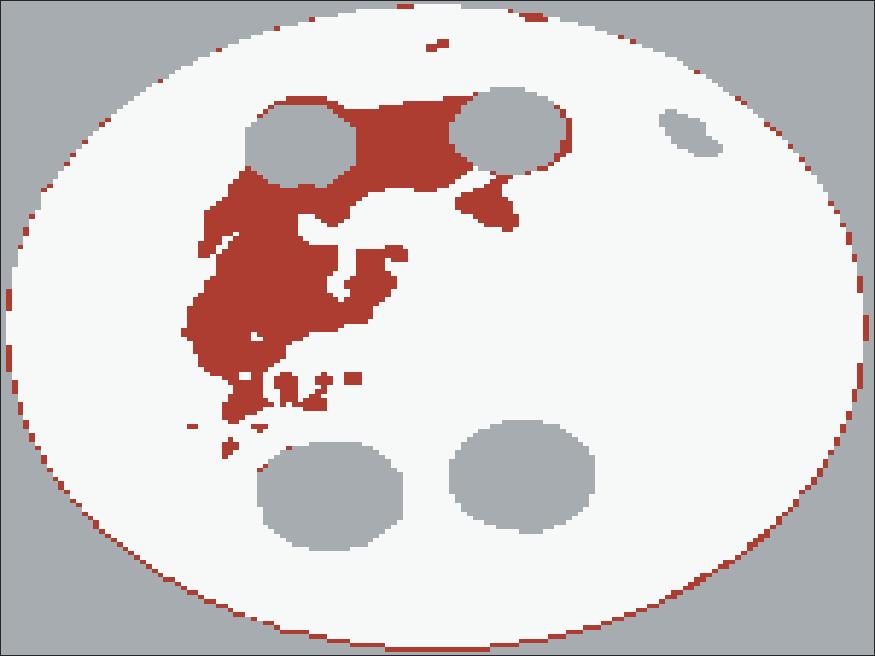

Supplement: Supplementary file 2 [file DataSheet1.ZIP › Dataset/synthetic_98HH1.jpg]

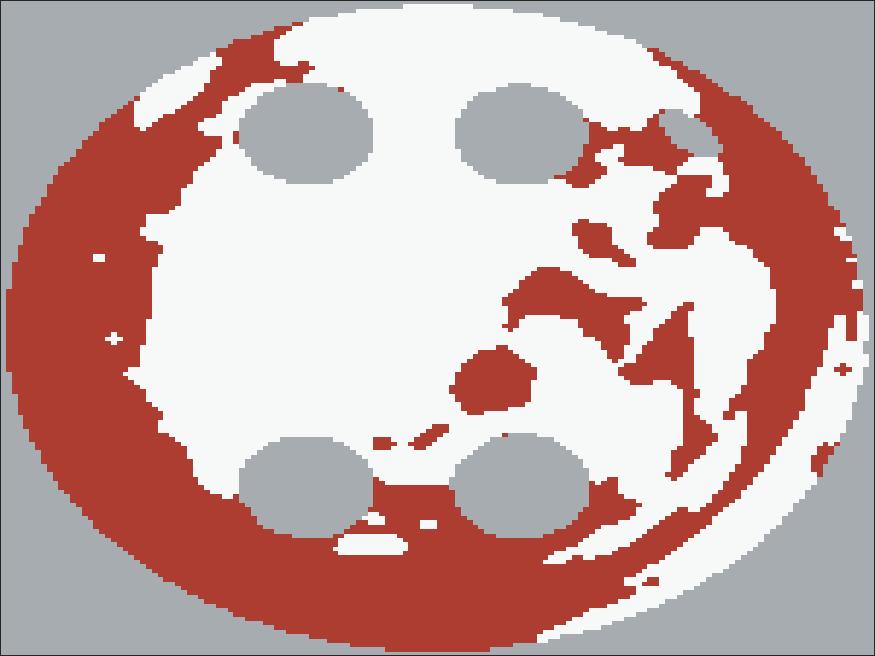

Supplement: Supplementary file 2 [file DataSheet1.ZIP › Dataset/synthetic_9QH2E.jpg]

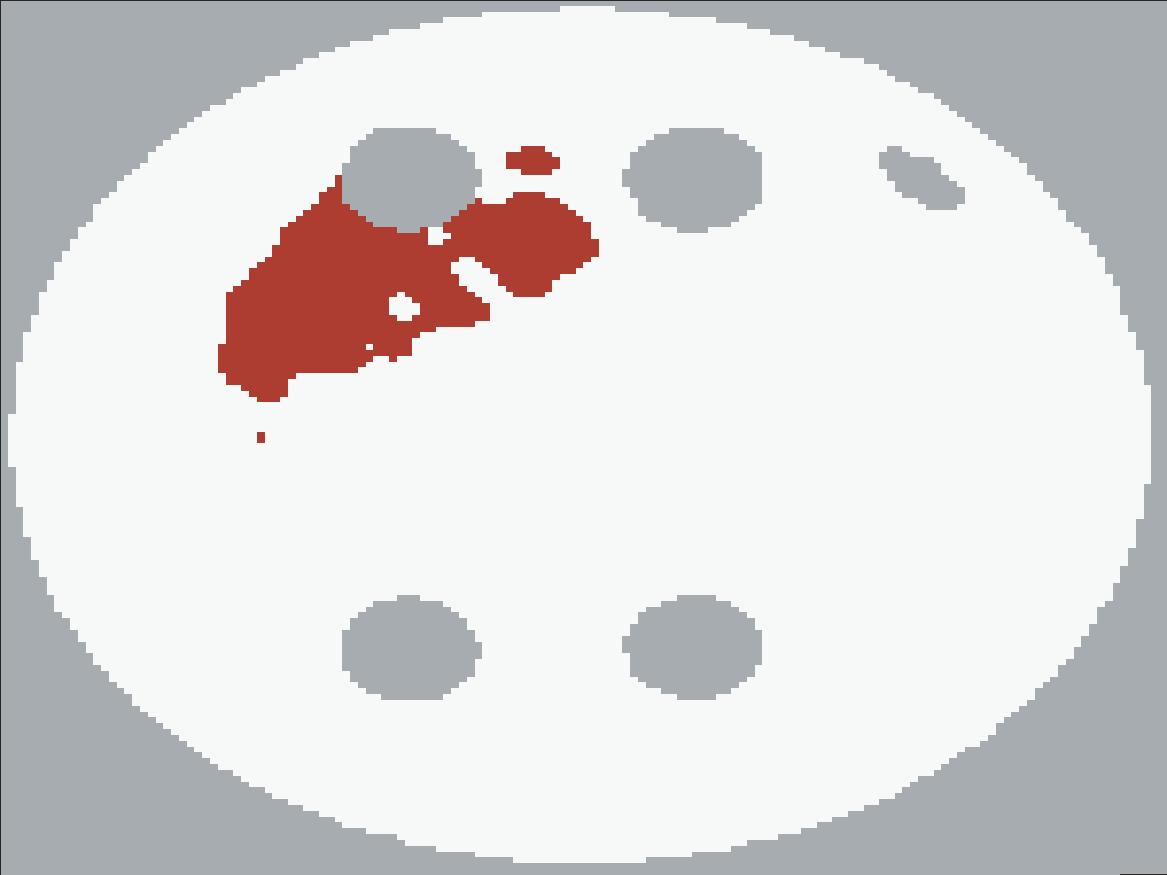

Supplement: Supplementary file 2 [file DataSheet1.ZIP › Dataset/synthetic_9UJ5E.jpg]

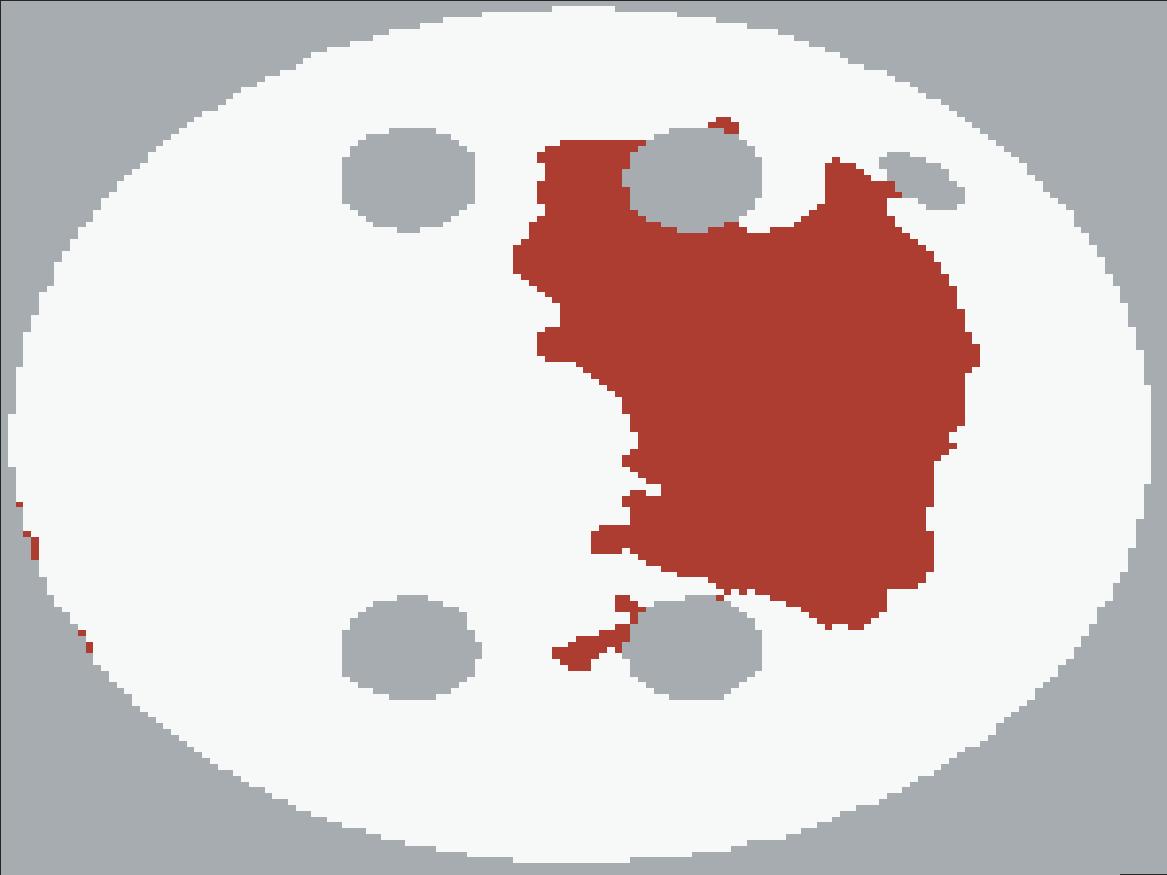

Supplement: Supplementary file 2 [file DataSheet1.ZIP › Dataset/synthetic_9ZXSR.jpg]

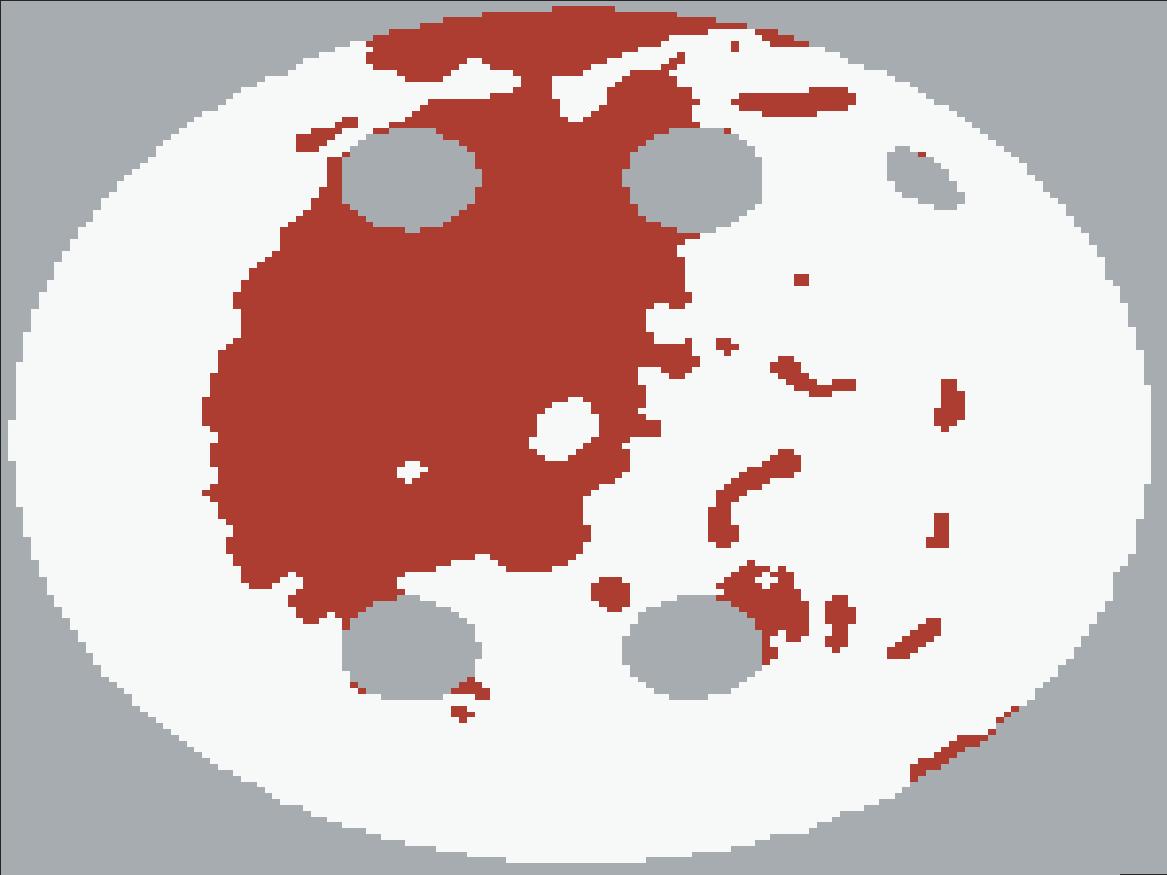

Supplement: Supplementary file 2 [file DataSheet1.ZIP › Dataset/synthetic_A8S82.jpg]

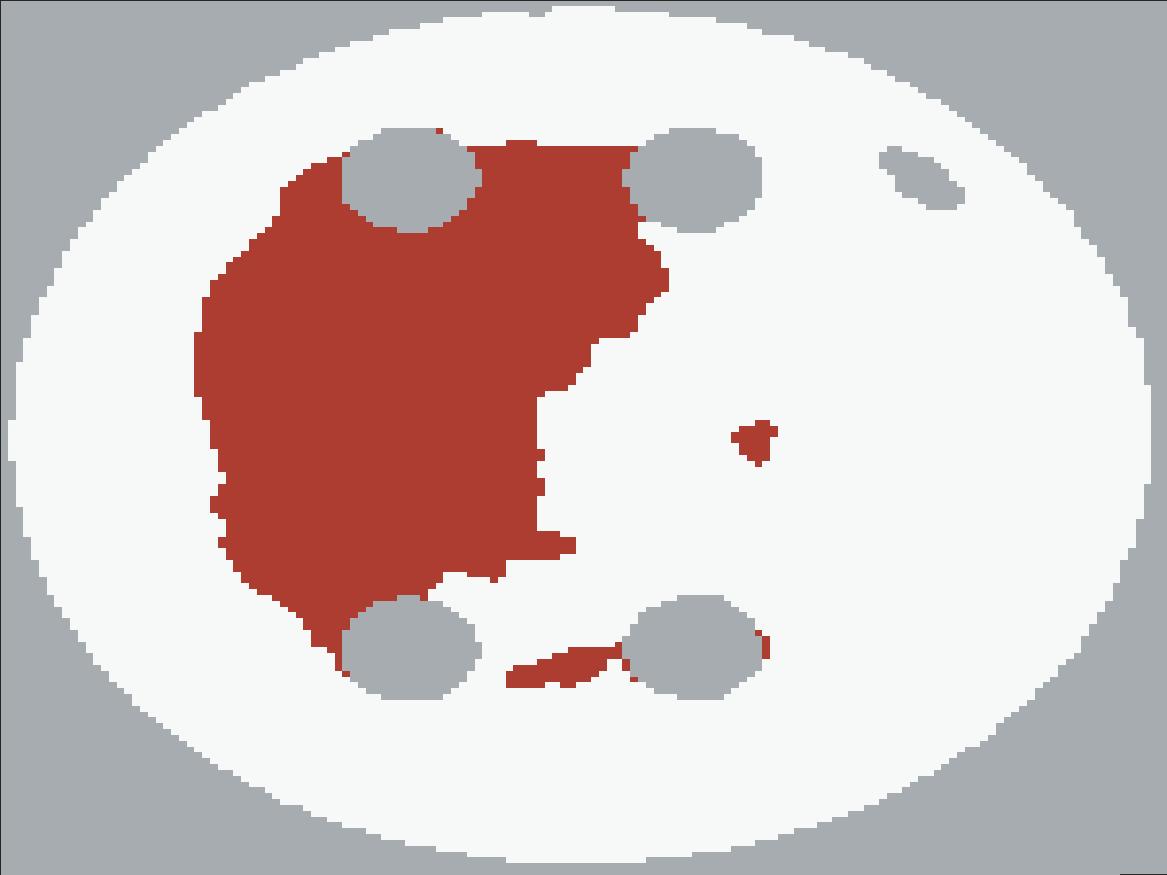

Supplement: Supplementary file 2 [file DataSheet1.ZIP › Dataset/synthetic_AC6K1.jpg]

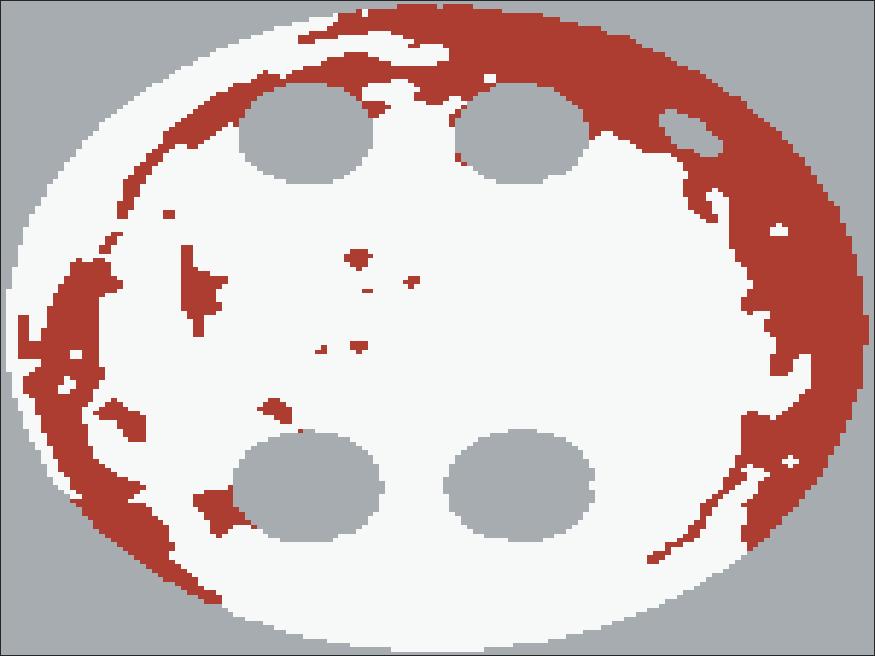

Supplement: Supplementary file 2 [file DataSheet1.ZIP › Dataset/synthetic_AD35W.jpg]

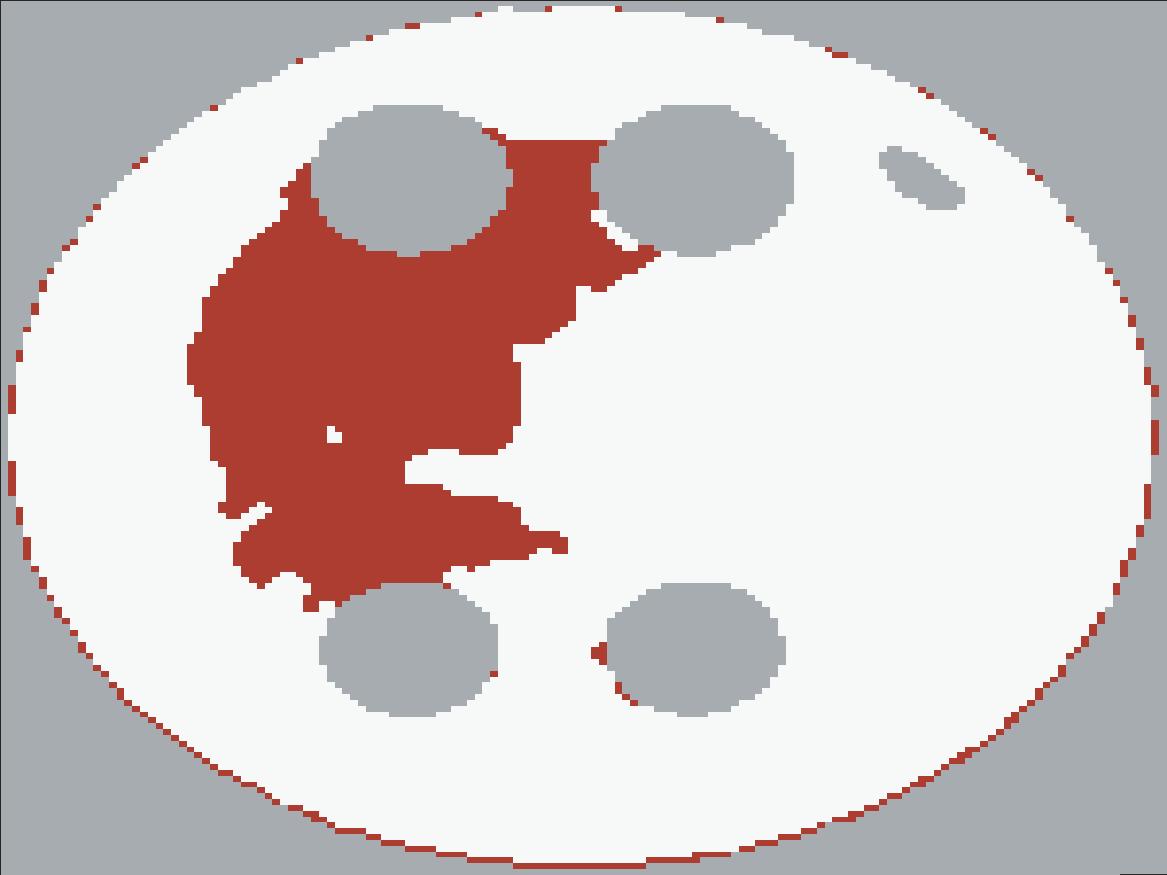

Supplement: Supplementary file 2 [file DataSheet1.ZIP › Dataset/synthetic_AD9JY.jpg]

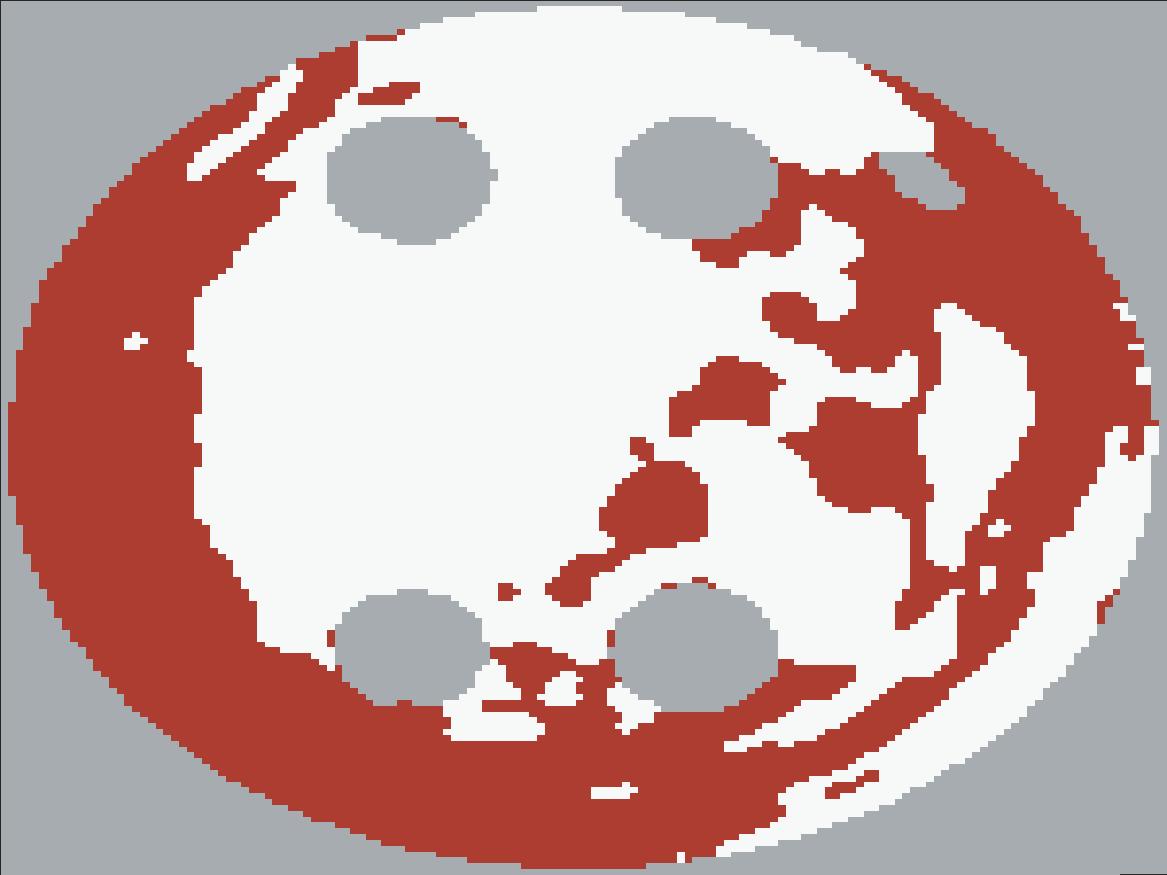

Supplement: Supplementary file 2 [file DataSheet1.ZIP › Dataset/synthetic_ADOAS.jpg]

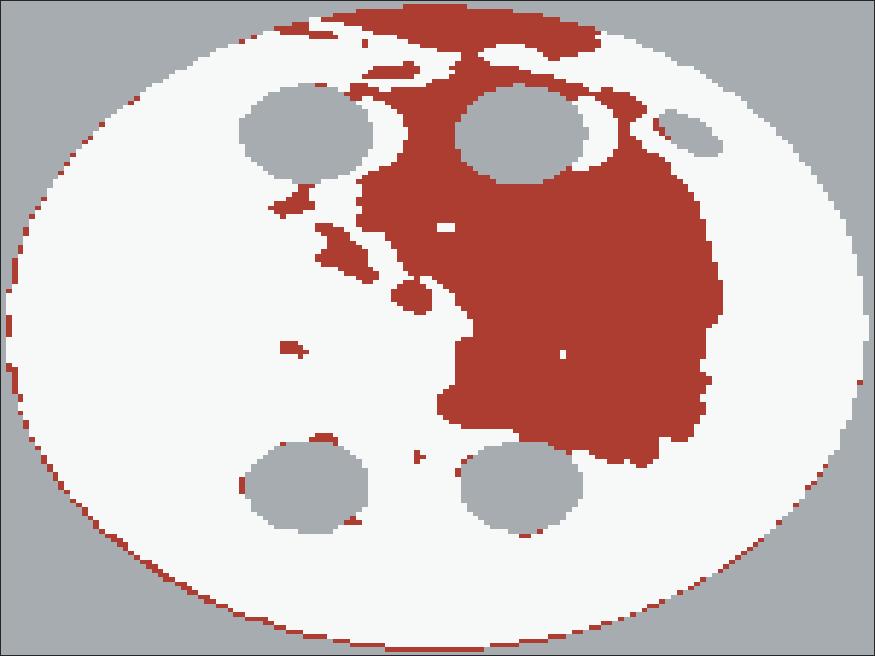

Supplement: Supplementary file 2 [file DataSheet1.ZIP › Dataset/synthetic_ATVYU.jpg]

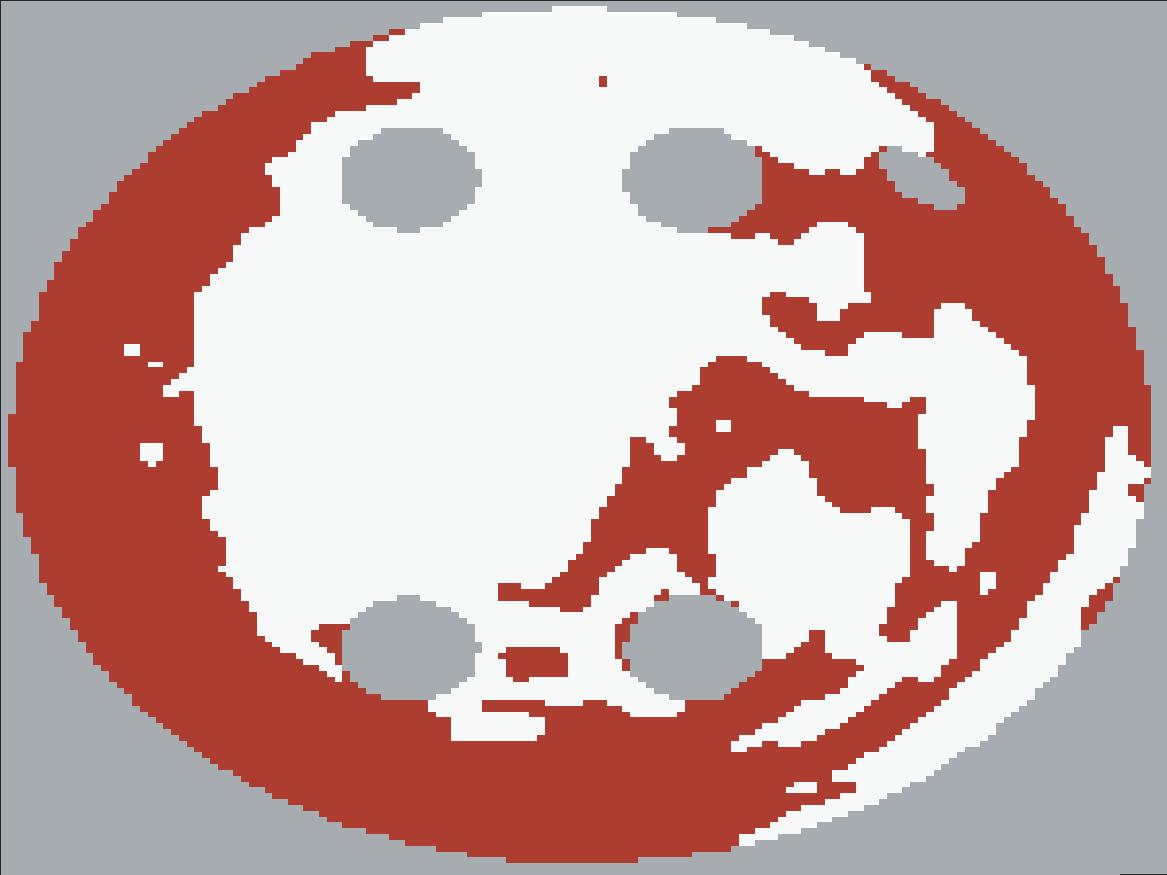

Supplement: Supplementary file 2 [file DataSheet1.ZIP › Dataset/synthetic_AVW8N.jpg]

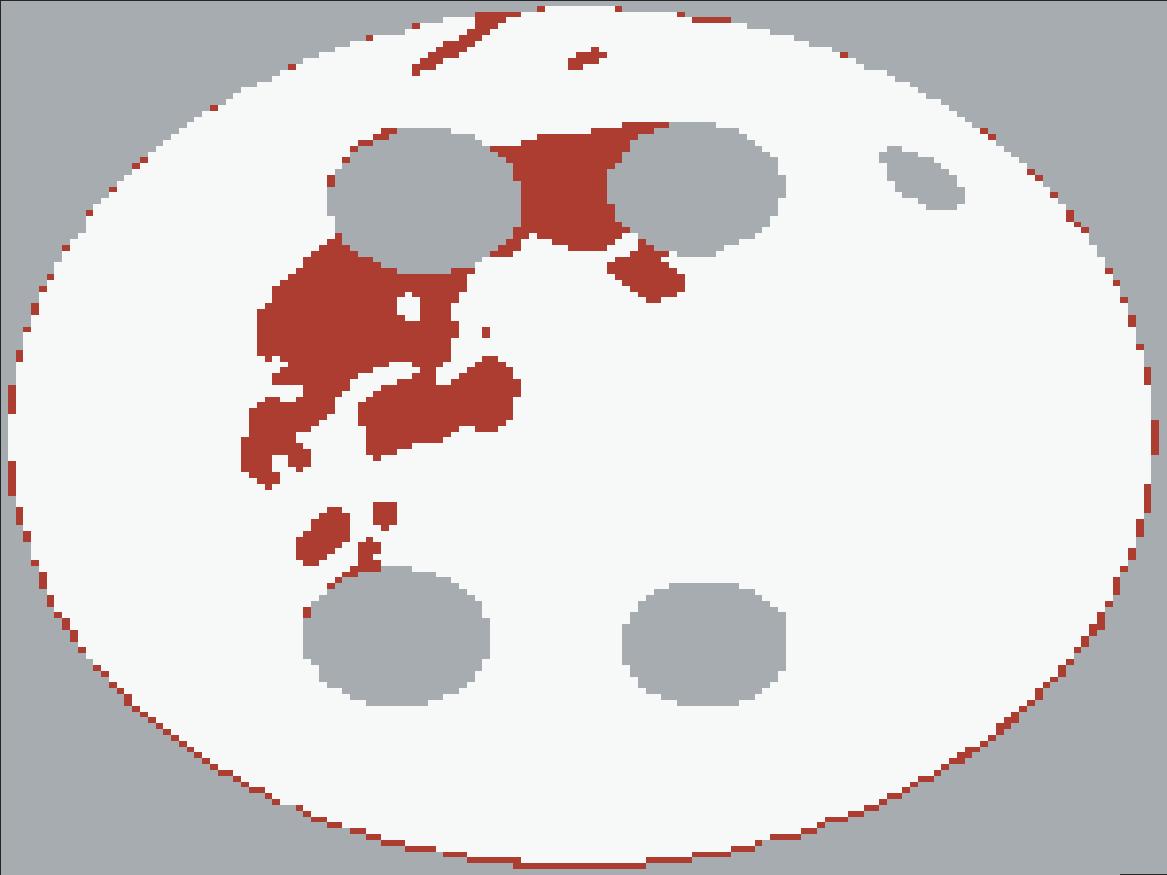

Supplement: Supplementary file 2 [file DataSheet1.ZIP › Dataset/synthetic_BEL0R.jpg]

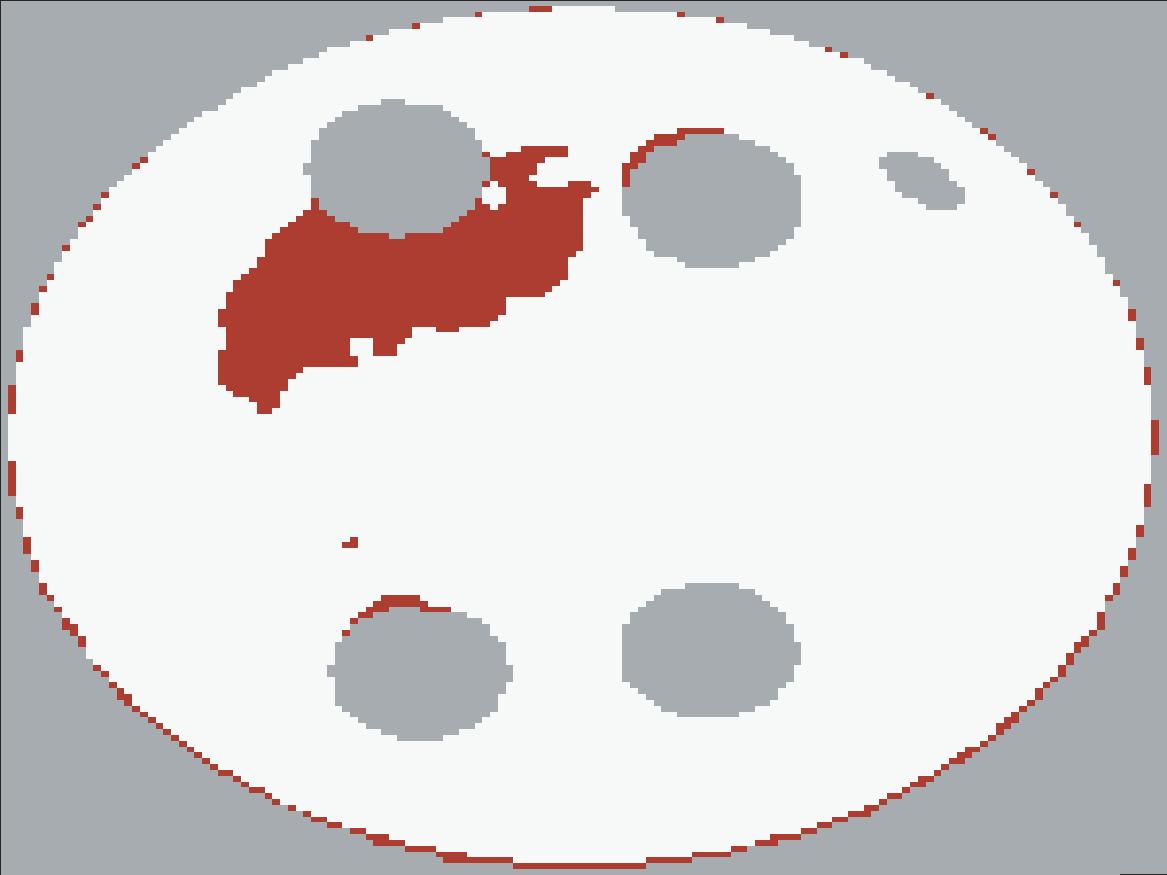

Supplement: Supplementary file 2 [file DataSheet1.ZIP › Dataset/synthetic_BGOUP.jpg]

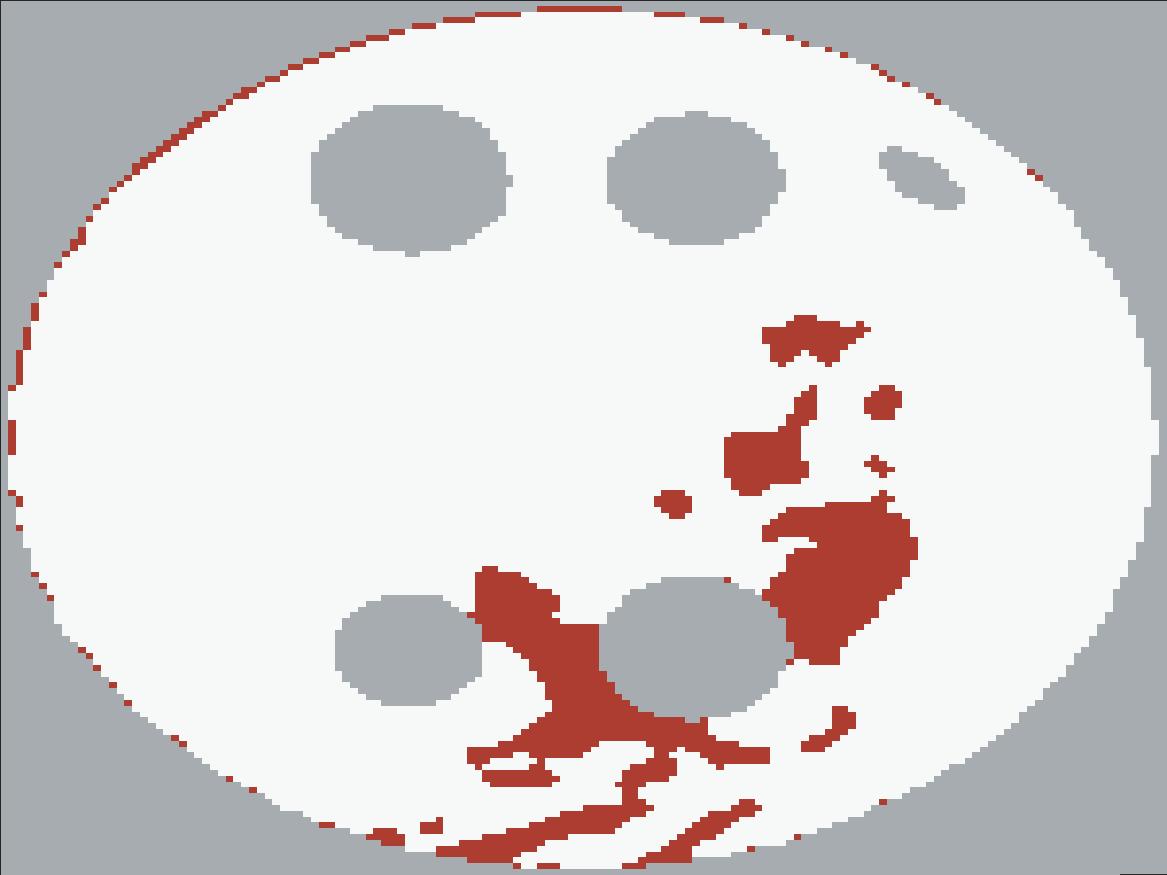

Supplement: Supplementary file 2 [file DataSheet1.ZIP › Dataset/synthetic_BNA6V.jpg]

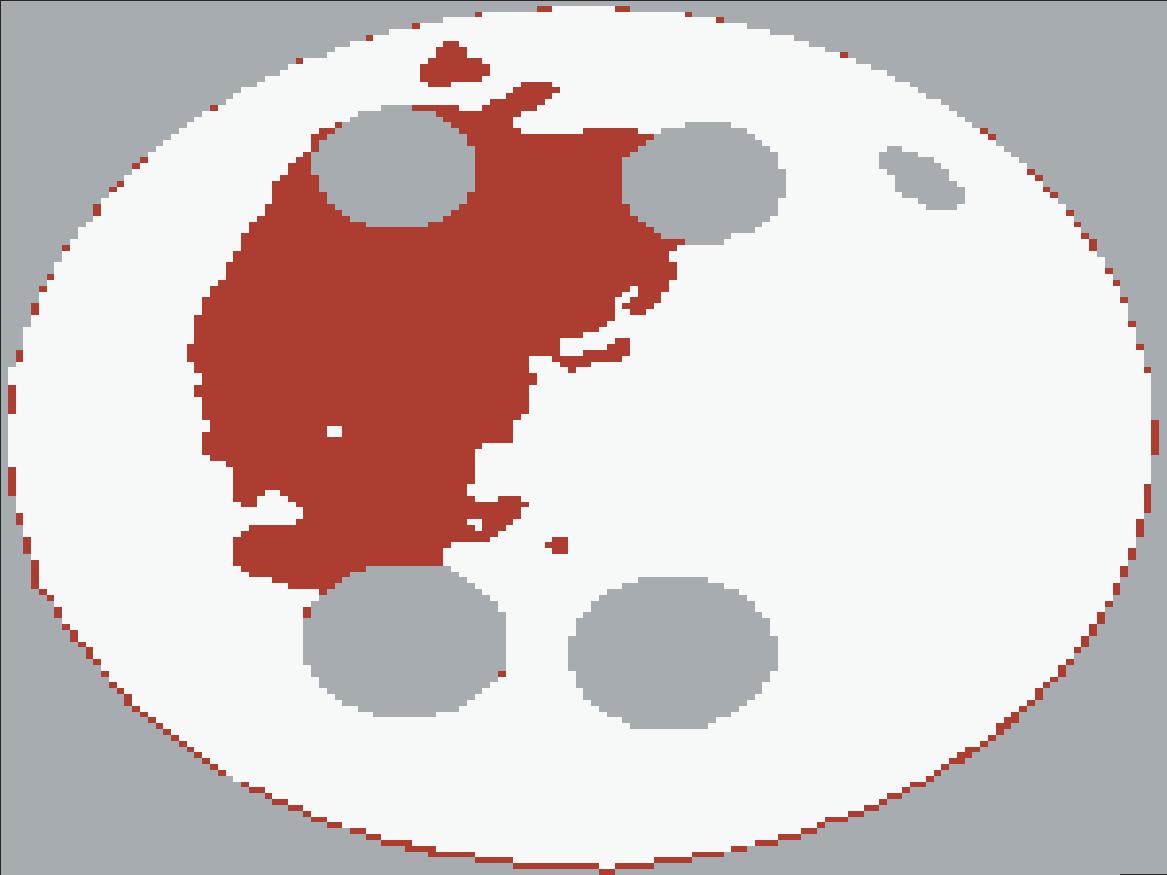

Supplement: Supplementary file 2 [file DataSheet1.ZIP › Dataset/synthetic_BVPPT.jpg]

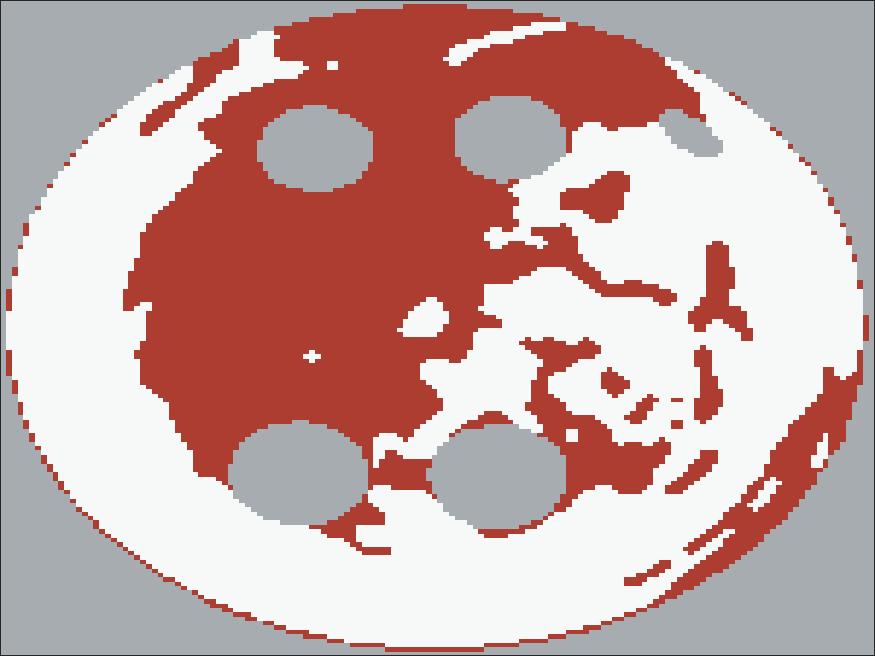

Supplement: Supplementary file 2 [file DataSheet1.ZIP › Dataset/synthetic_C19MF.jpg]

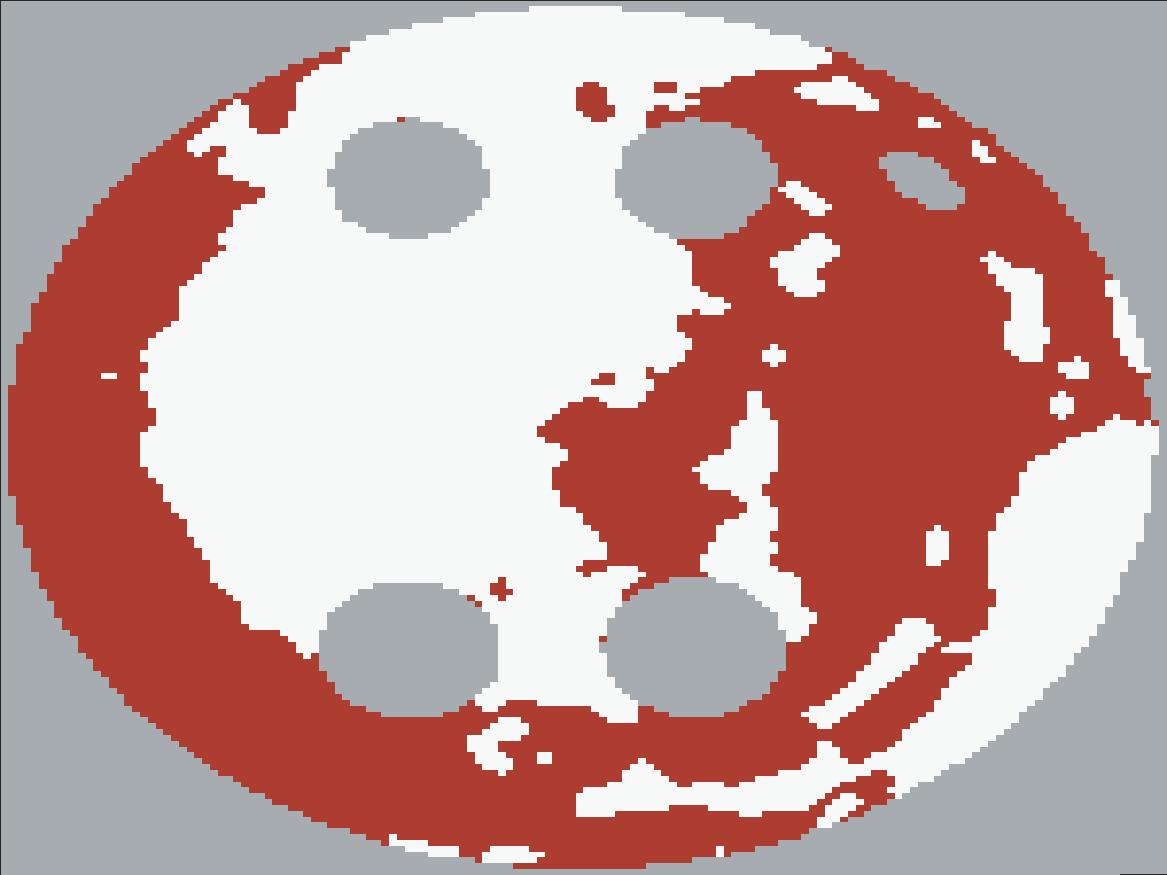

Supplement: Supplementary file 2 [file DataSheet1.ZIP › Dataset/synthetic_C1XI5.jpg]
