# Supplementary figures and images for: Exploring interpretability in deep learning prediction of successful ablation therapy for atrial fibrillation (part 1 of 2)
Source: Front Physiol. 2023 Mar 14;14:1054401. doi: 10.3389/fphys.2023.1054401 (PMC10043207; doi:10.3389/fphys.2023.1054401)

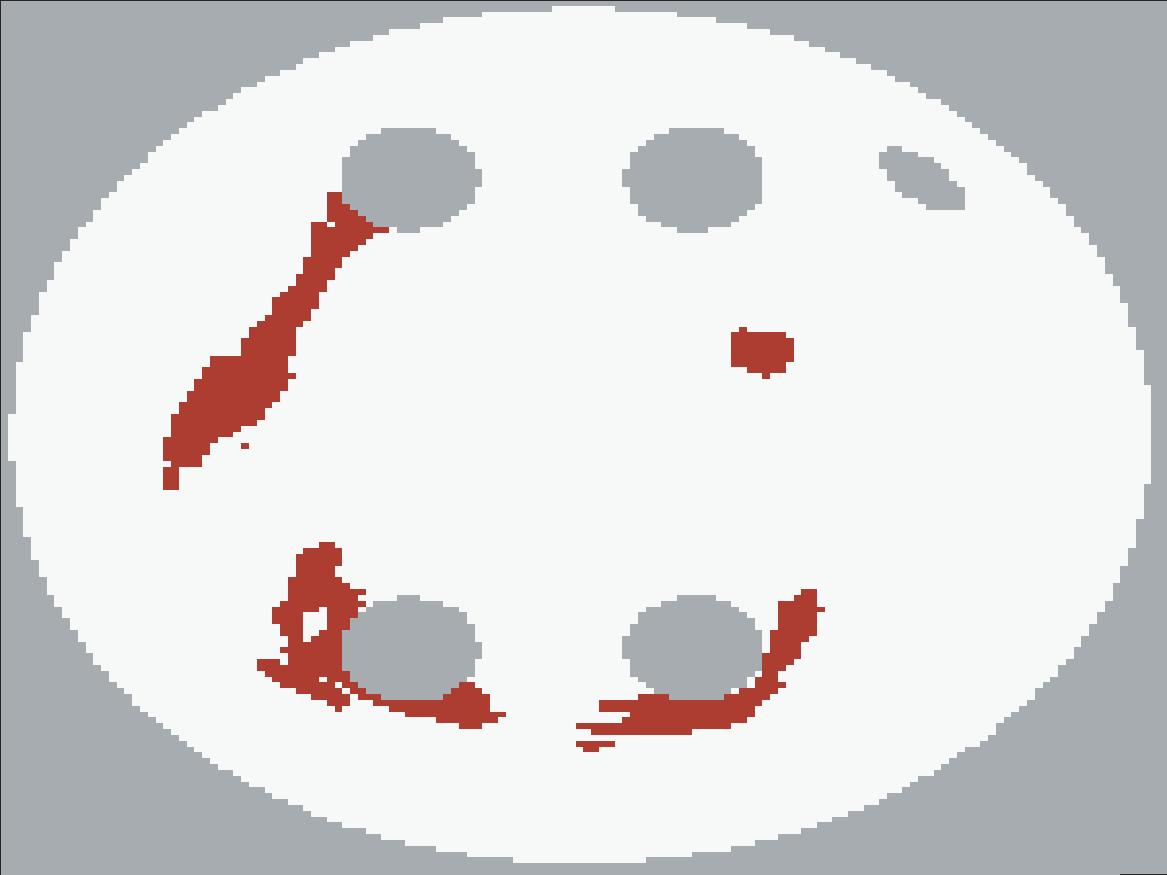

Supplement: Supplementary file 2 [file DataSheet1.ZIP › Dataset/real_12440.jpg]

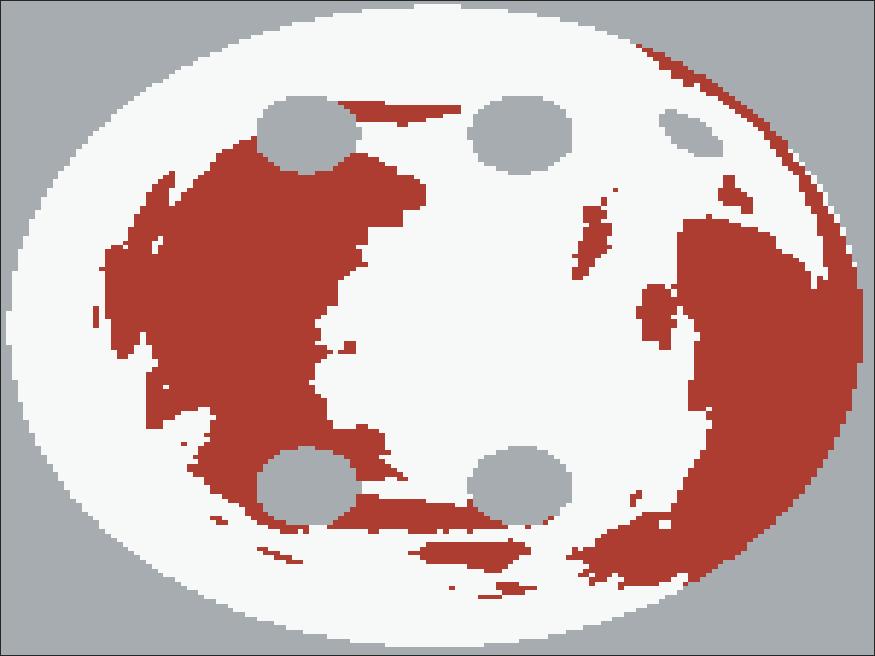

Supplement: Supplementary file 2 [file DataSheet1.ZIP › Dataset/real_15VB5.jpg]

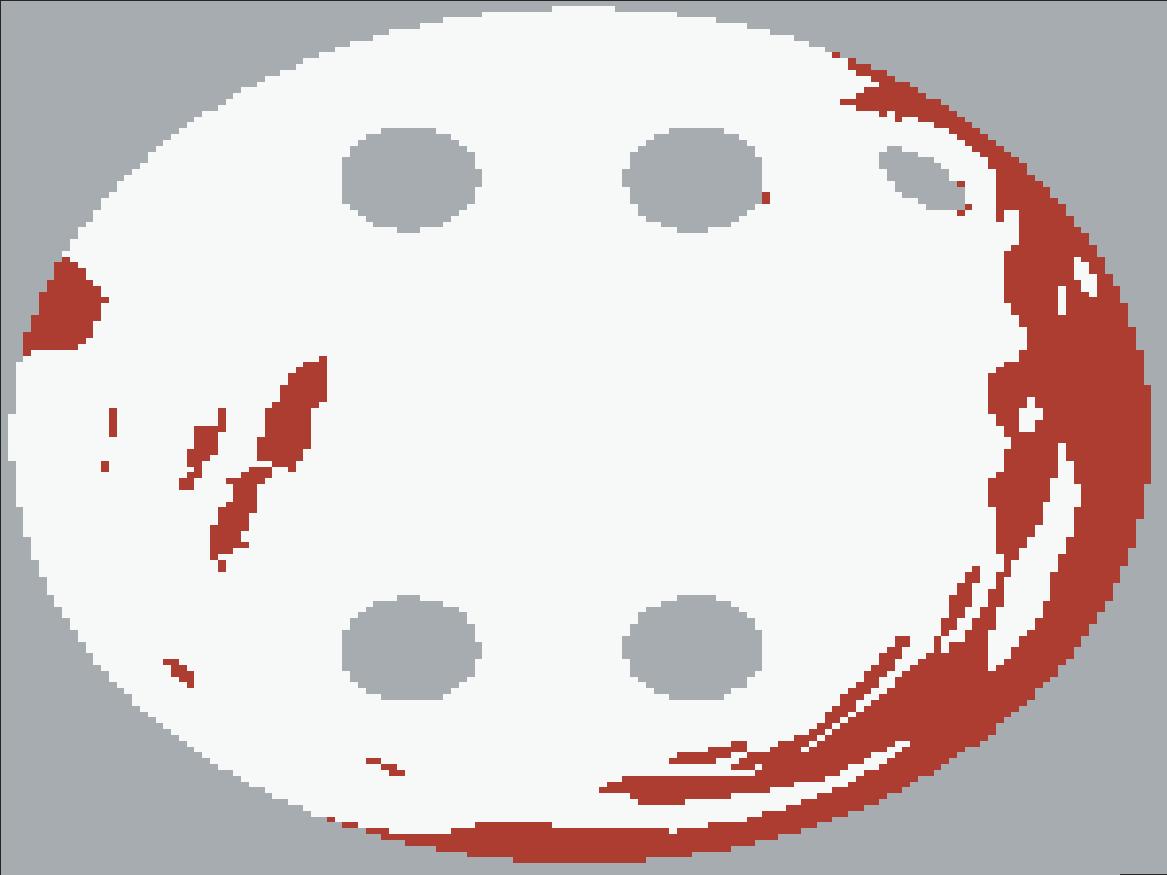

Supplement: Supplementary file 2 [file DataSheet1.ZIP › Dataset/real_1F9US.jpg]

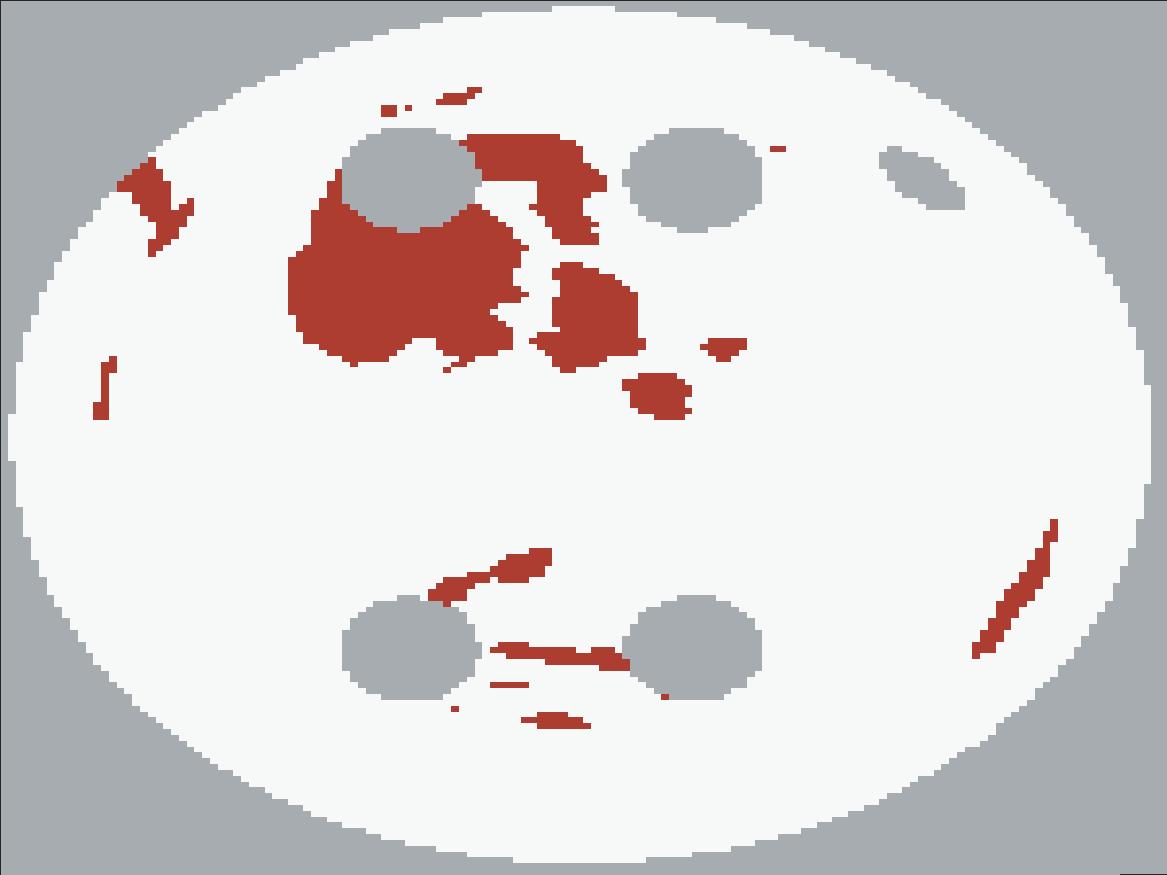

Supplement: Supplementary file 2 [file DataSheet1.ZIP › Dataset/real_1ORQD.jpg]

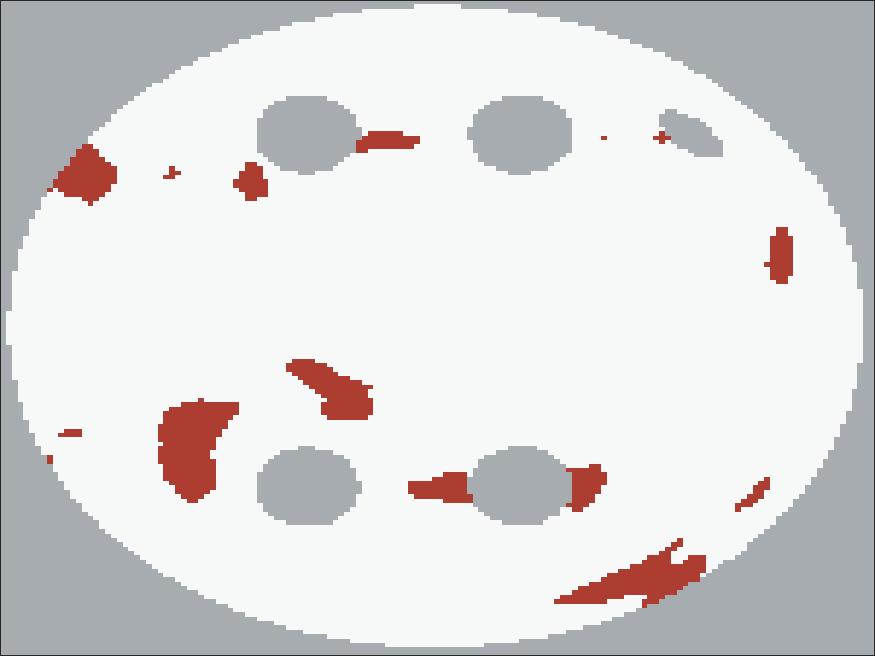

Supplement: Supplementary file 2 [file DataSheet1.ZIP › Dataset/real_1R71W.jpg]

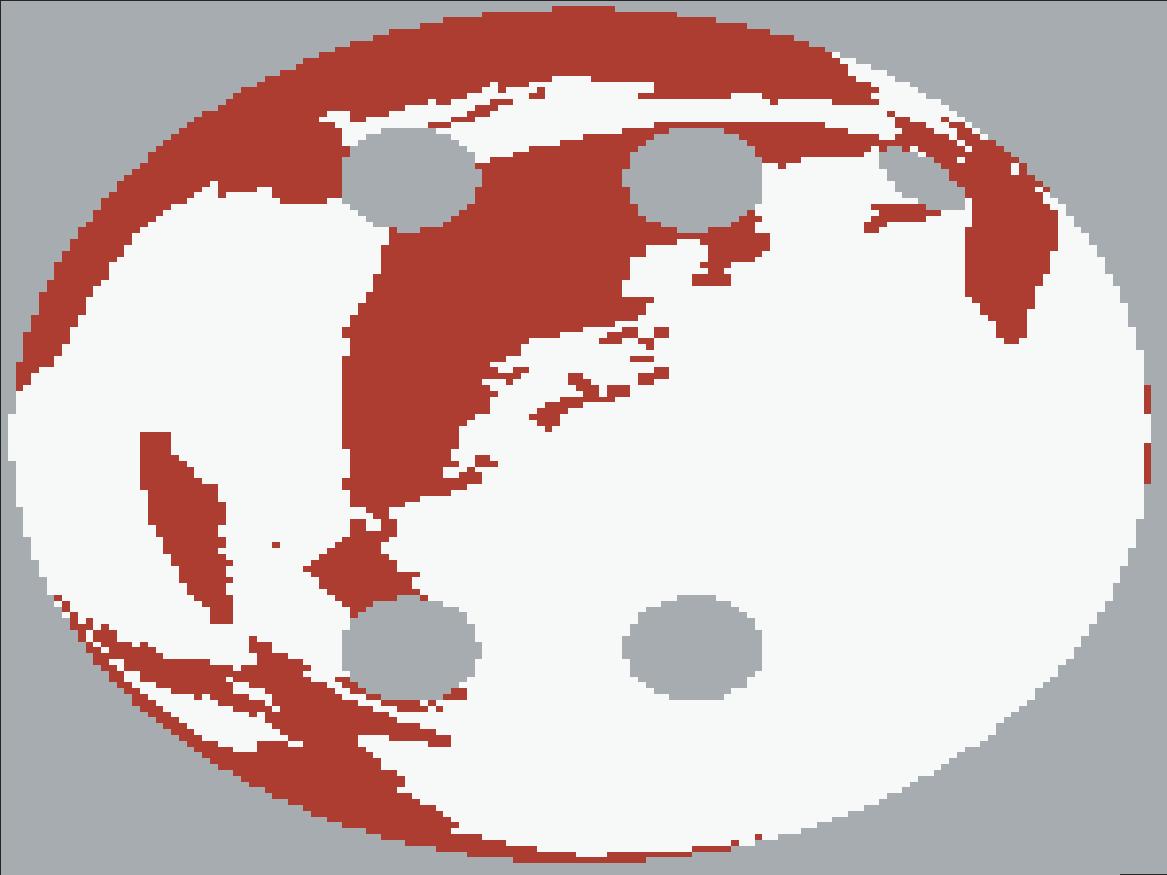

Supplement: Supplementary file 2 [file DataSheet1.ZIP › Dataset/real_1SXIH.jpg]

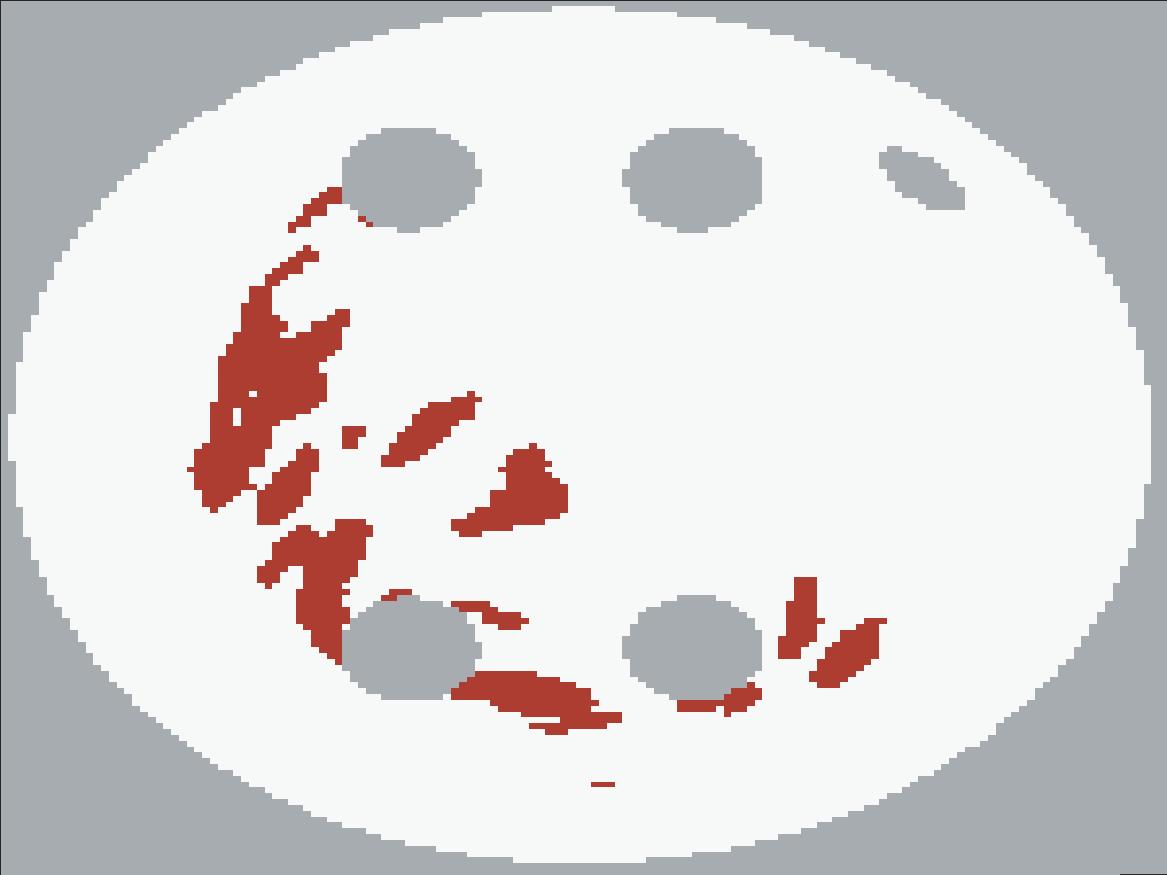

Supplement: Supplementary file 2 [file DataSheet1.ZIP › Dataset/real_1V7JO.jpg]

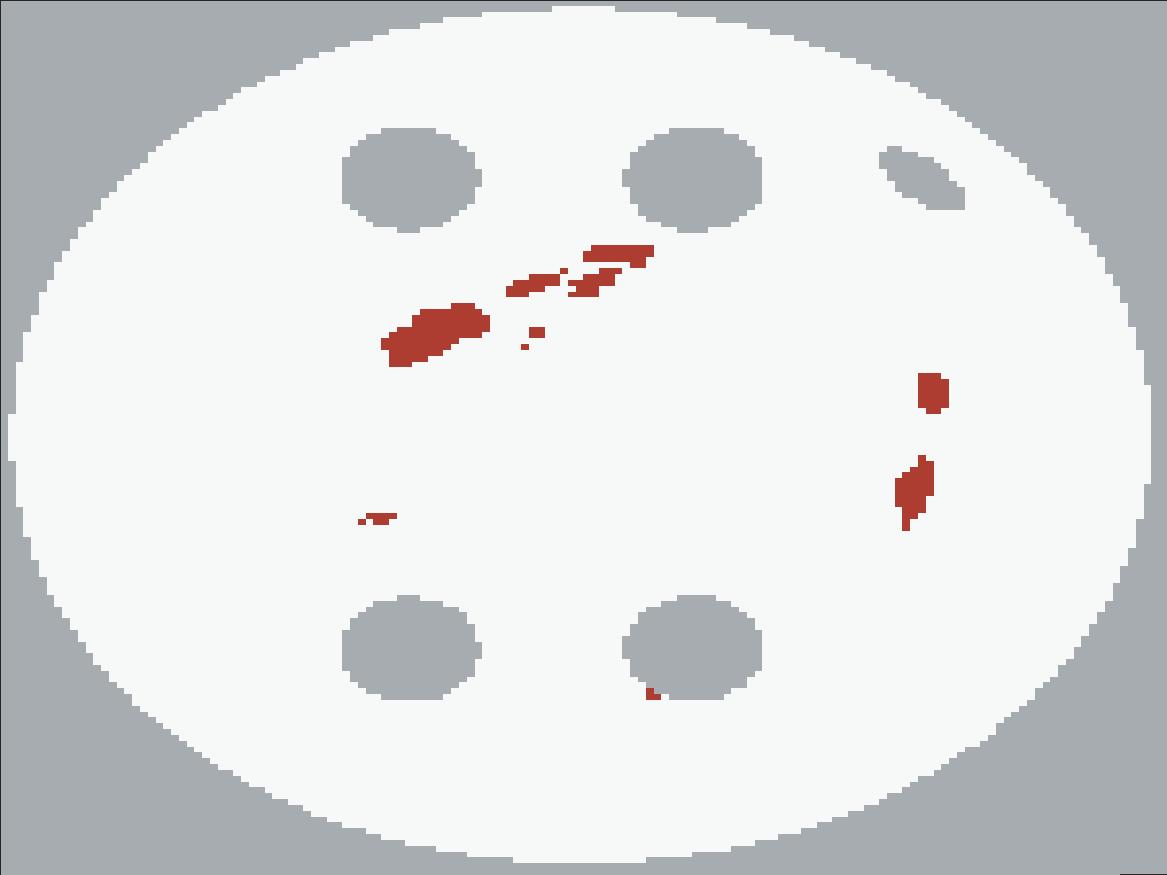

Supplement: Supplementary file 2 [file DataSheet1.ZIP › Dataset/real_1W2UL.jpg]

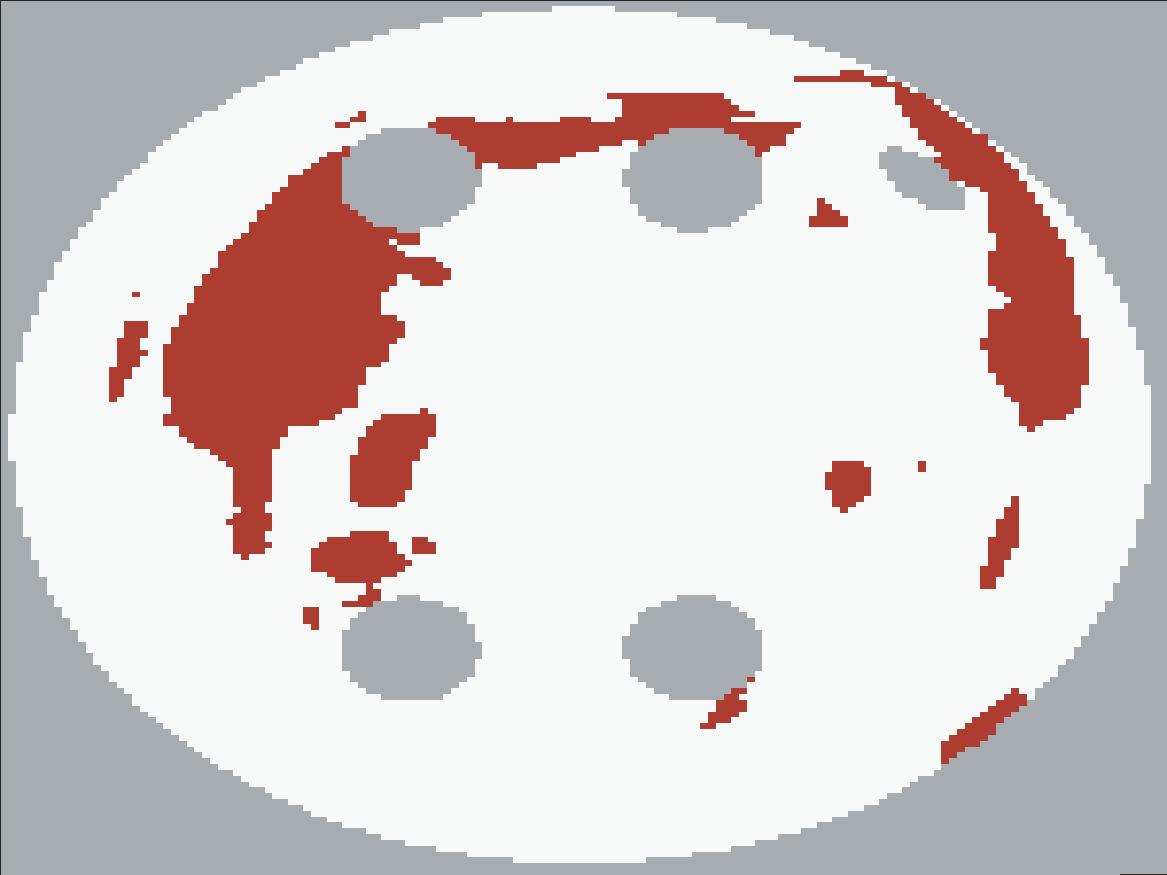

Supplement: Supplementary file 2 [file DataSheet1.ZIP › Dataset/real_2LW0J.jpg]

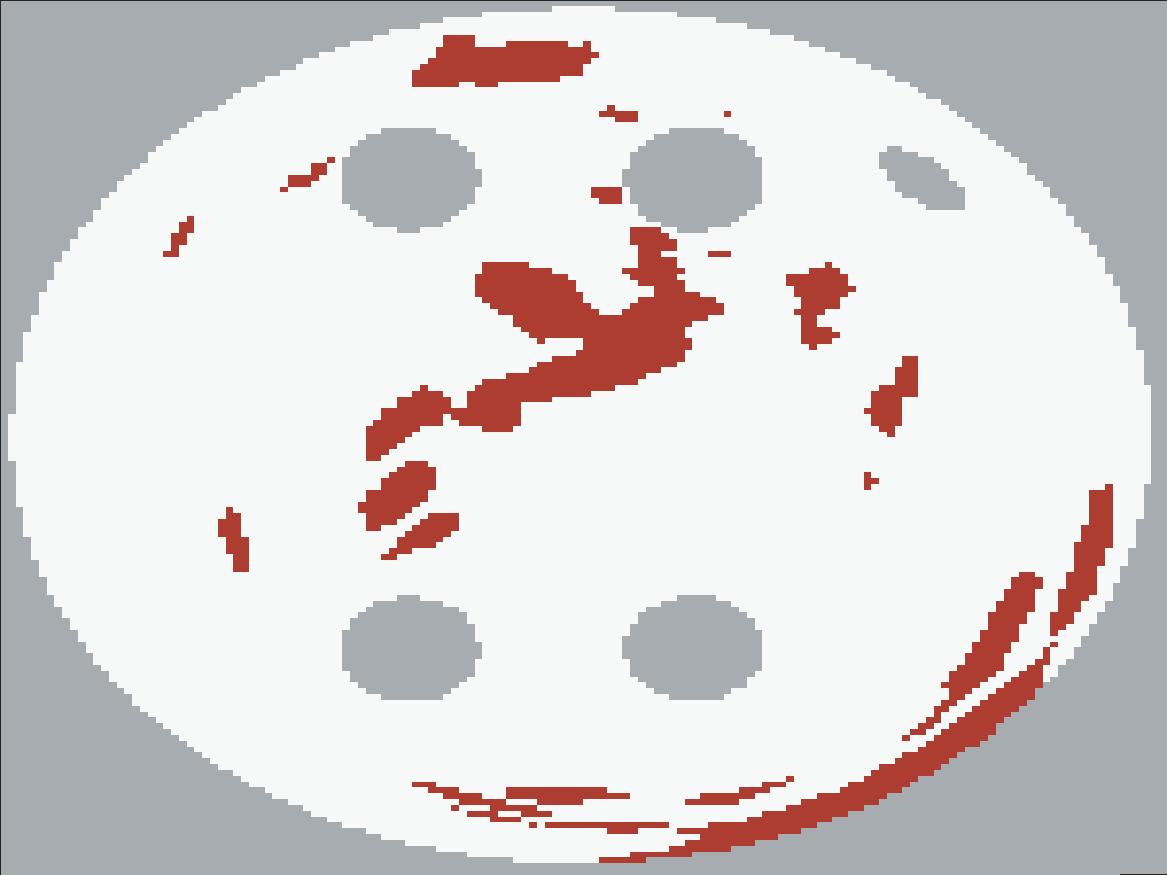

Supplement: Supplementary file 2 [file DataSheet1.ZIP › Dataset/real_2ONJP.jpg]

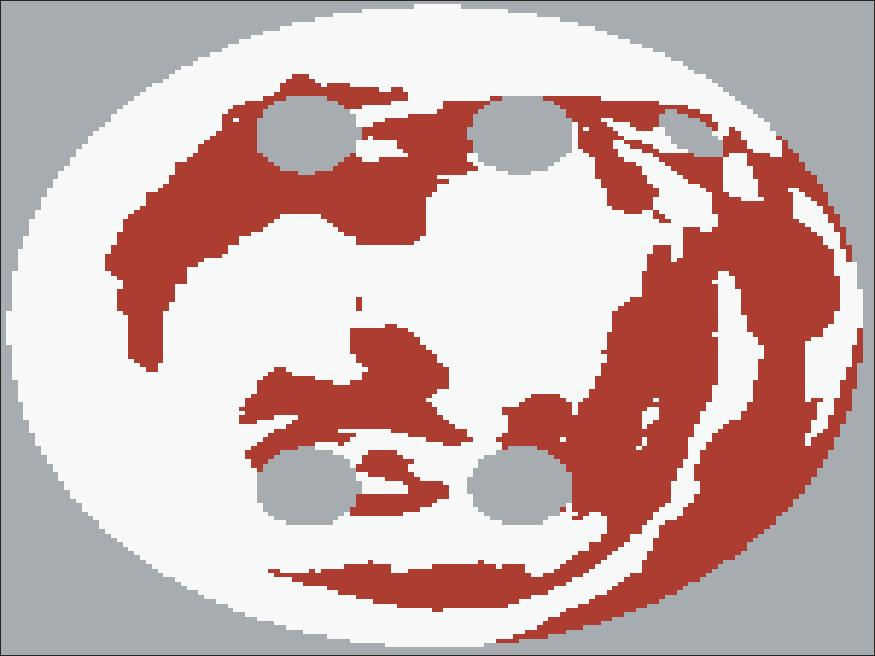

Supplement: Supplementary file 2 [file DataSheet1.ZIP › Dataset/real_3BBIZ.jpg]

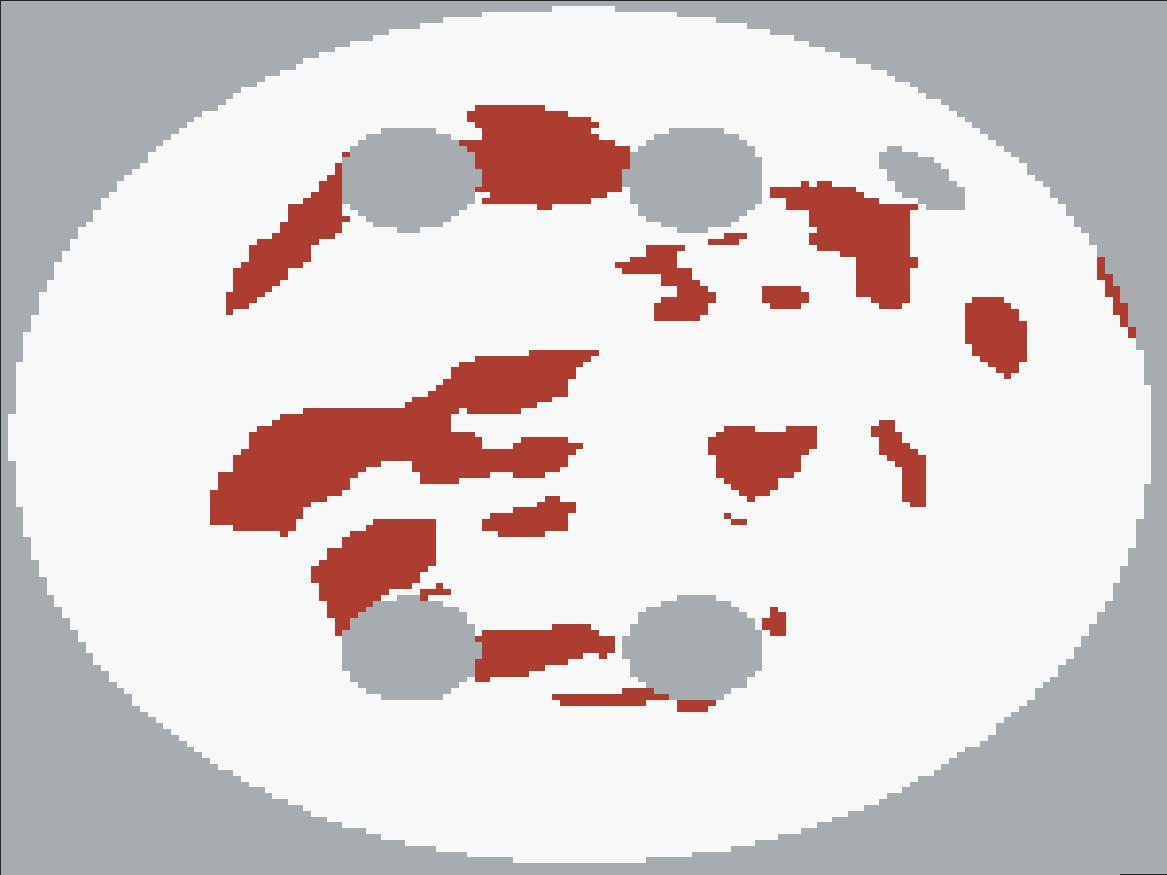

Supplement: Supplementary file 2 [file DataSheet1.ZIP › Dataset/real_3G6E4.jpg]

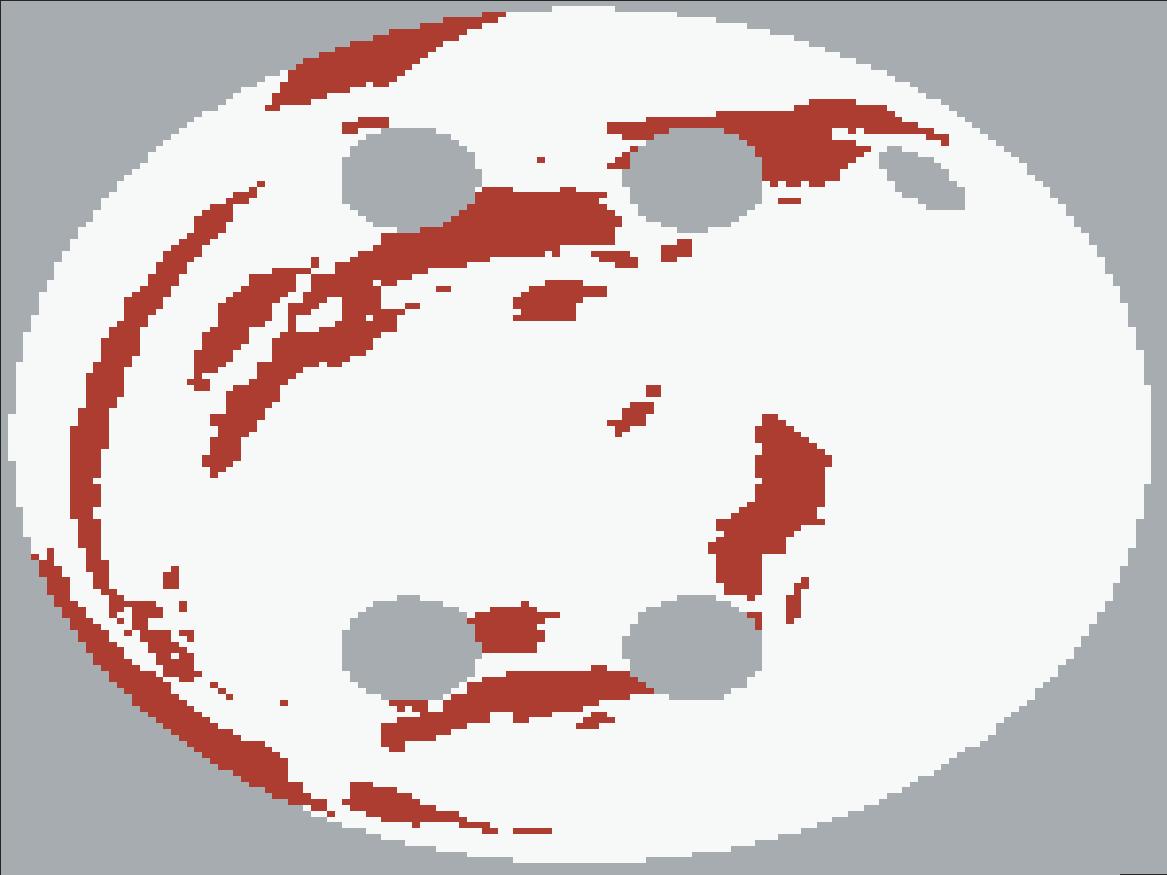

Supplement: Supplementary file 2 [file DataSheet1.ZIP › Dataset/real_3QJM7.jpg]

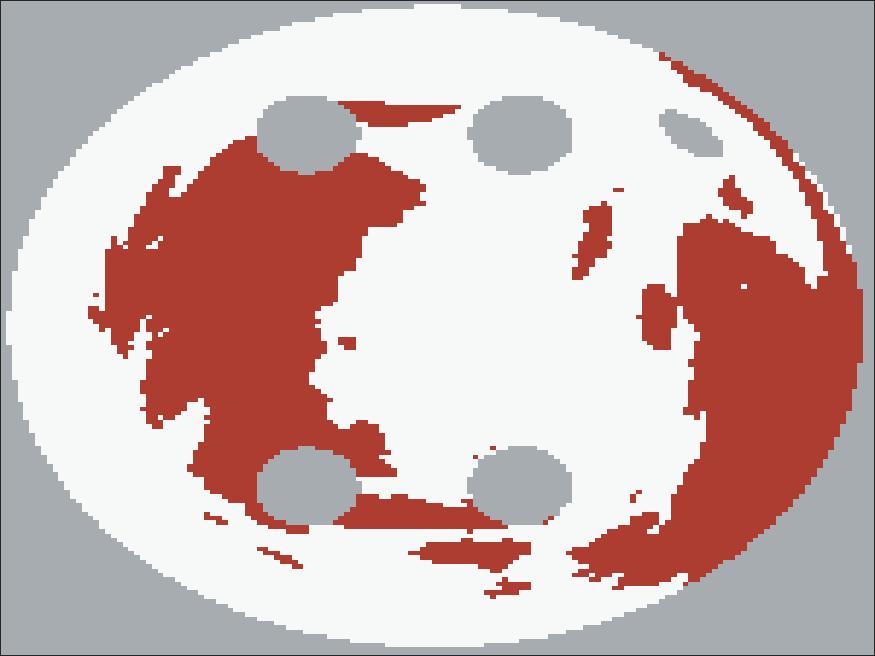

Supplement: Supplementary file 2 [file DataSheet1.ZIP › Dataset/real_3XA98.jpg]

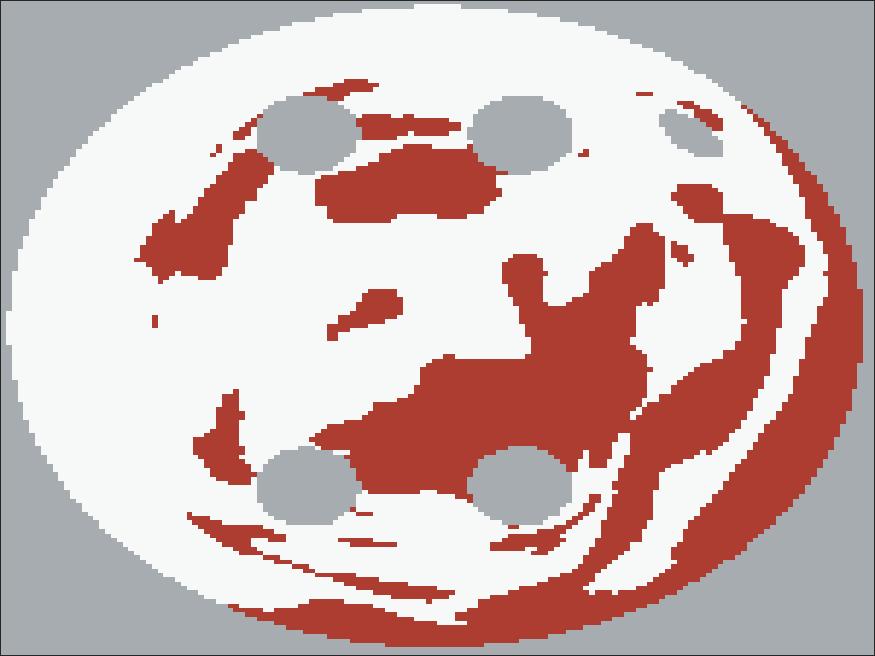

Supplement: Supplementary file 2 [file DataSheet1.ZIP › Dataset/real_453ZB.jpg]

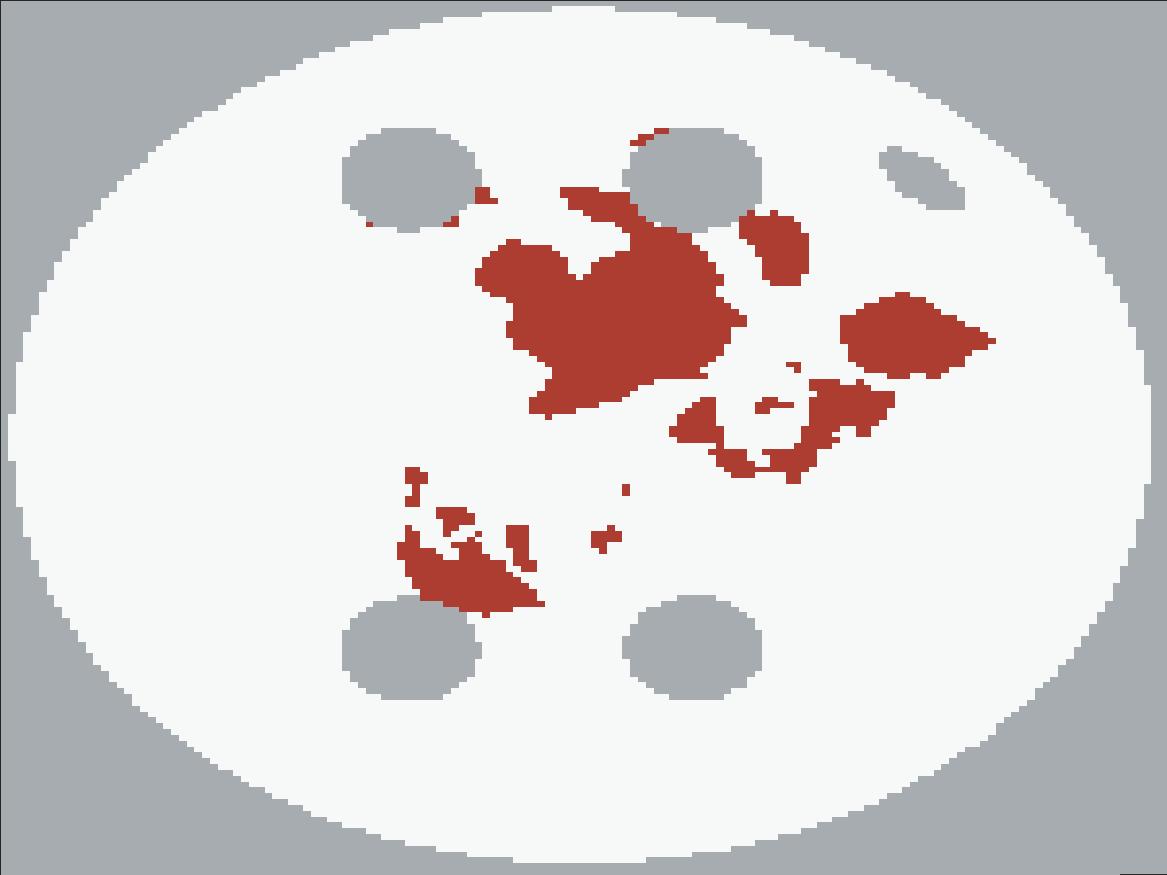

Supplement: Supplementary file 2 [file DataSheet1.ZIP › Dataset/real_46JK1.jpg]

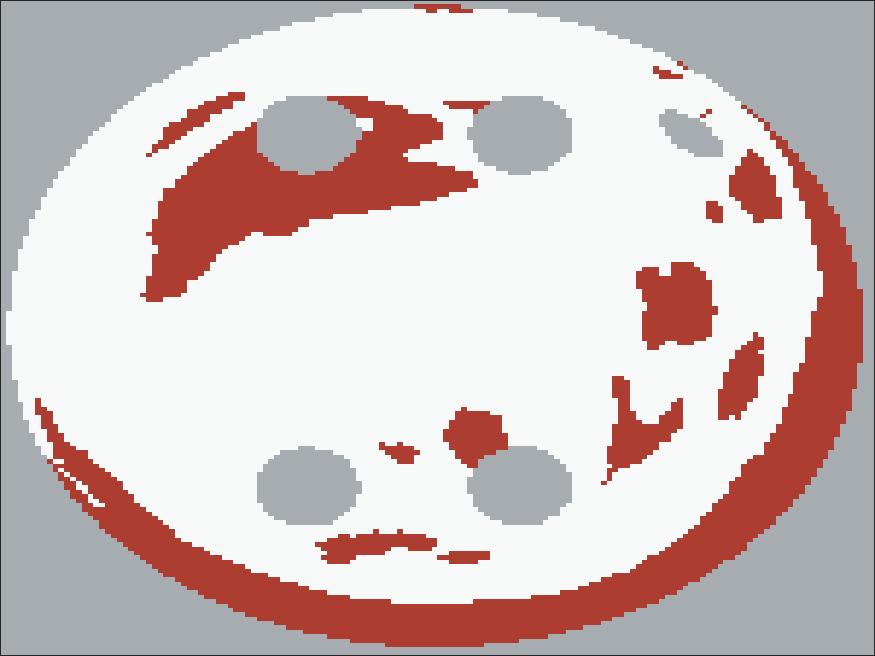

Supplement: Supplementary file 2 [file DataSheet1.ZIP › Dataset/real_4KDC0.jpg]

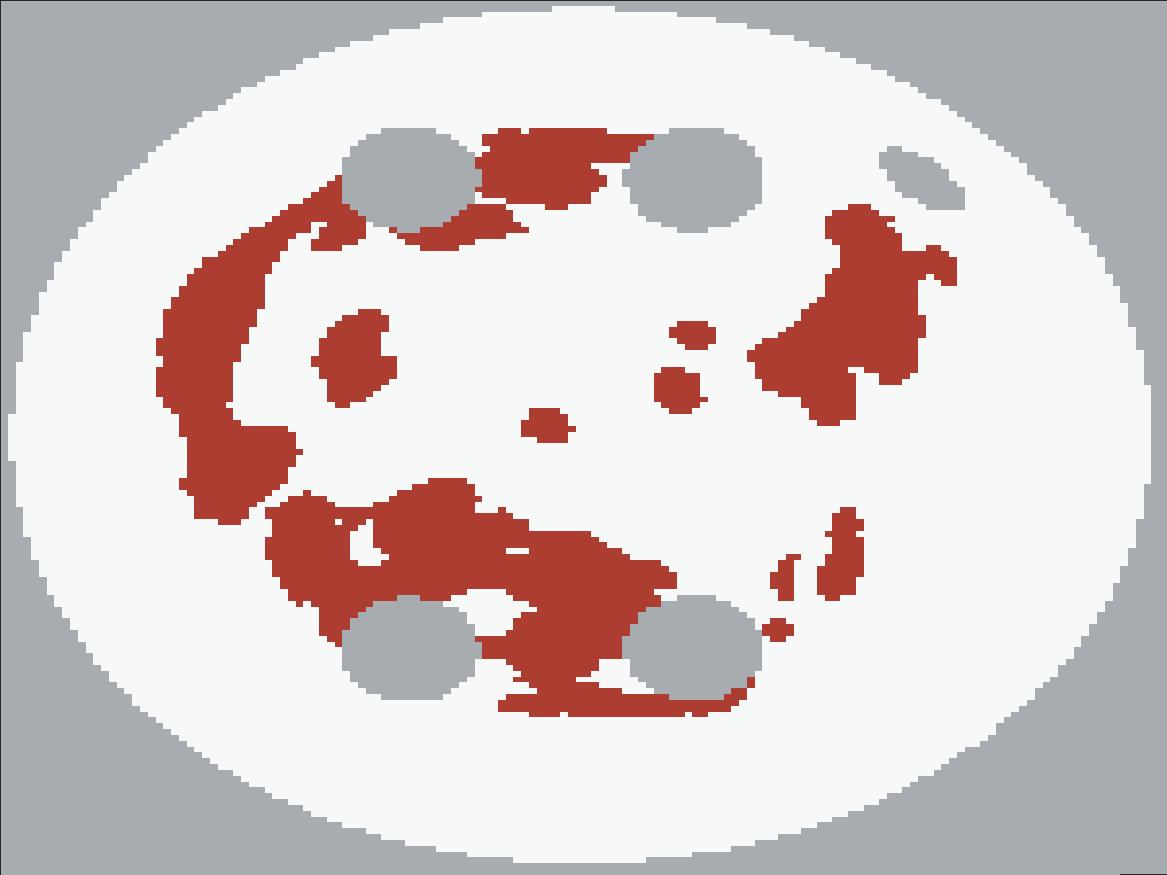

Supplement: Supplementary file 2 [file DataSheet1.ZIP › Dataset/real_4OWBV.jpg]

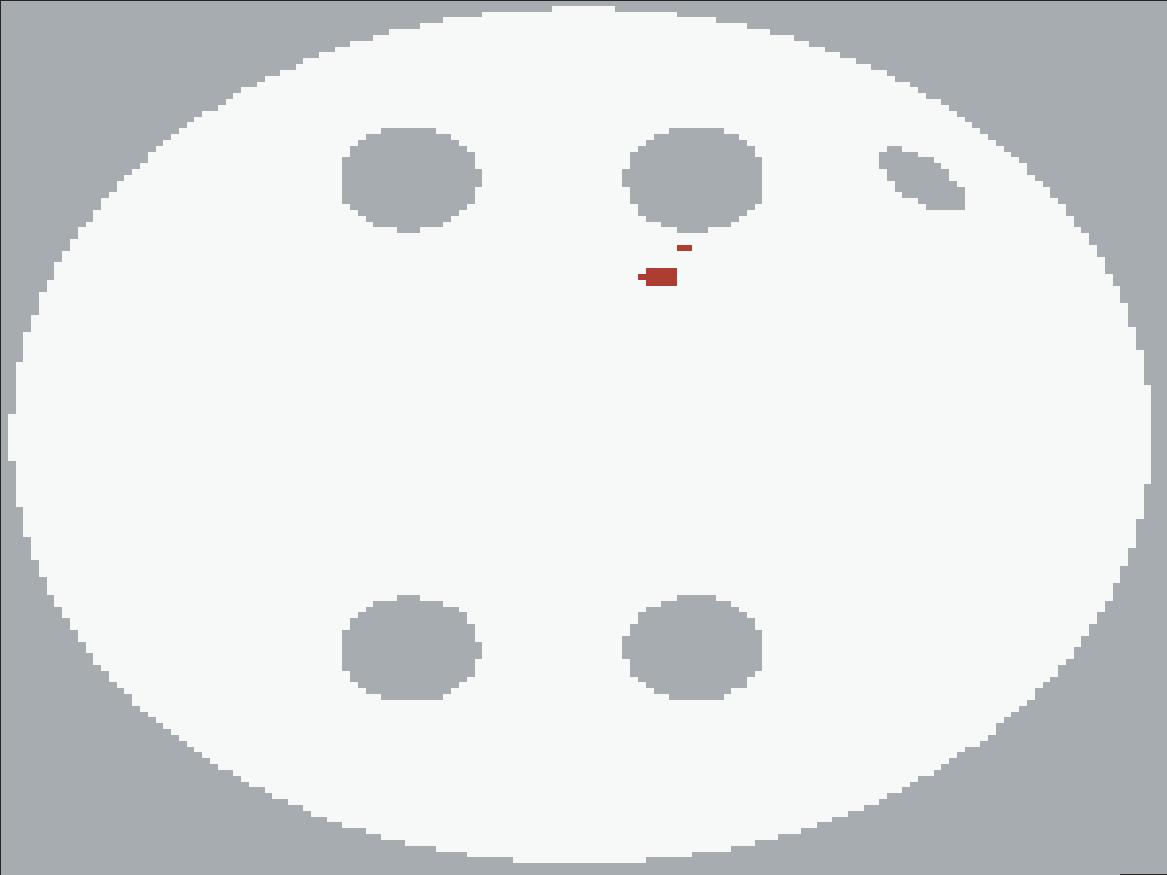

Supplement: Supplementary file 2 [file DataSheet1.ZIP › Dataset/real_4SKIE.jpg]

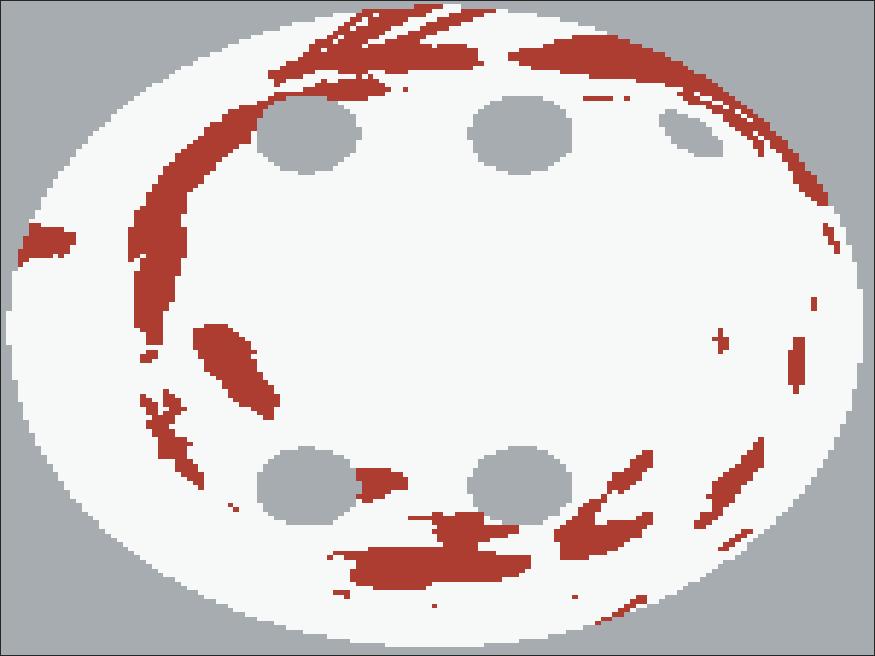

Supplement: Supplementary file 2 [file DataSheet1.ZIP › Dataset/real_4XD2S.jpg]

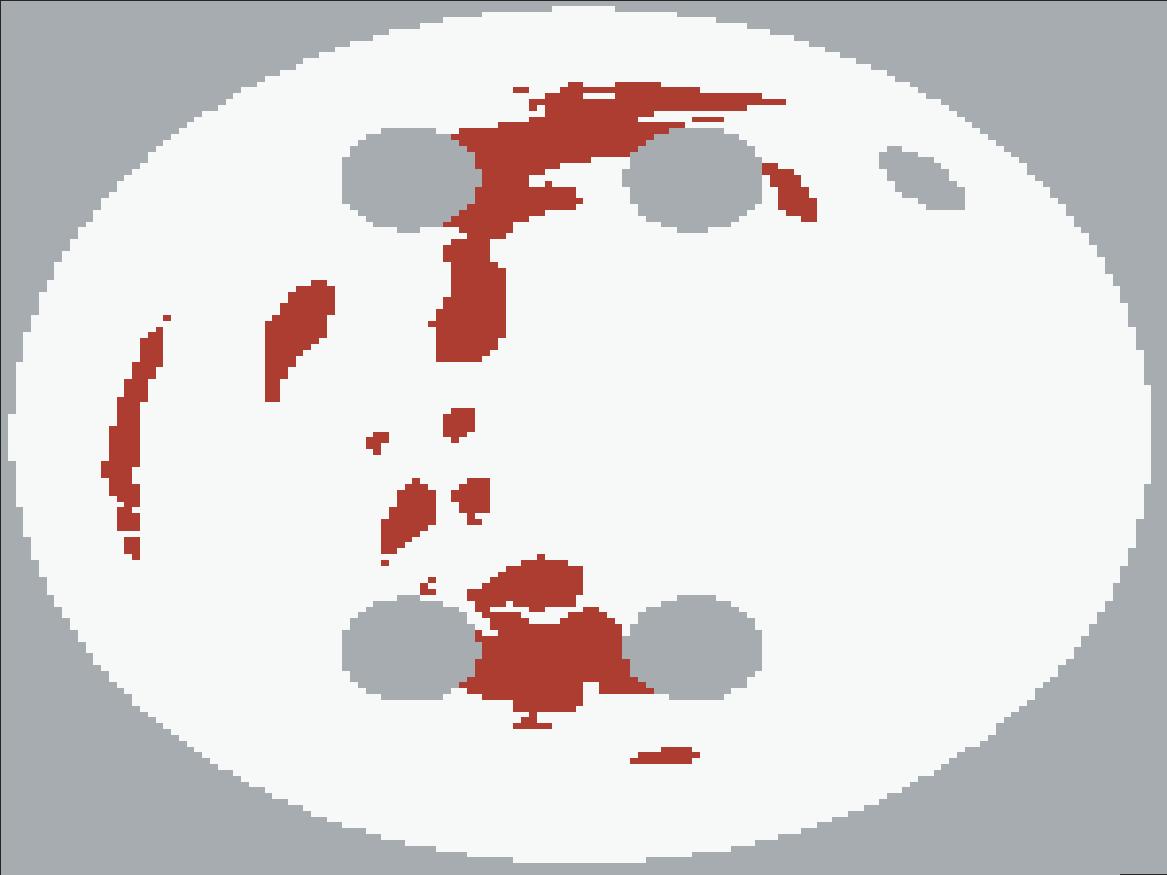

Supplement: Supplementary file 2 [file DataSheet1.ZIP › Dataset/real_56LNQ.jpg]

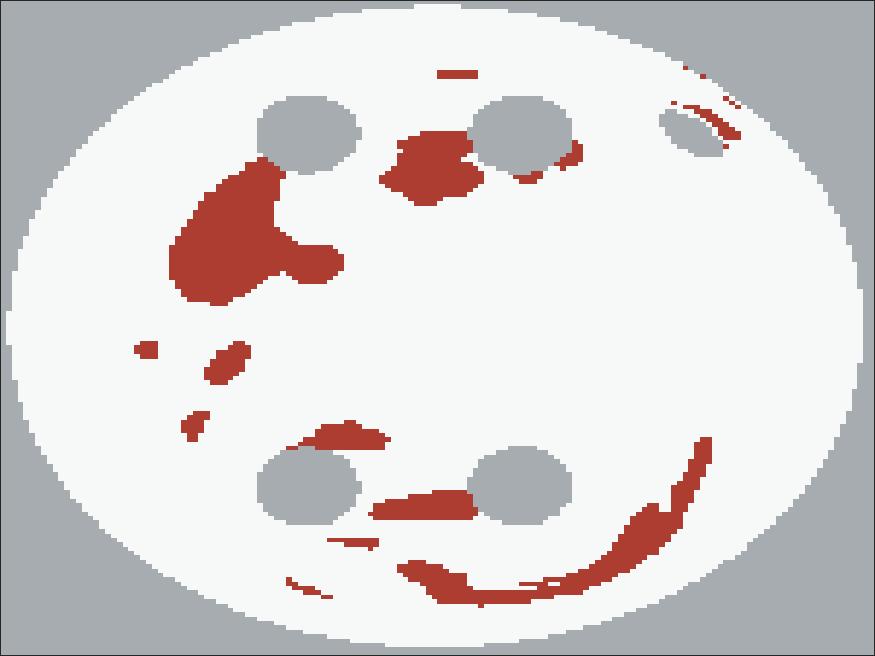

Supplement: Supplementary file 2 [file DataSheet1.ZIP › Dataset/real_5SVKU.jpg]

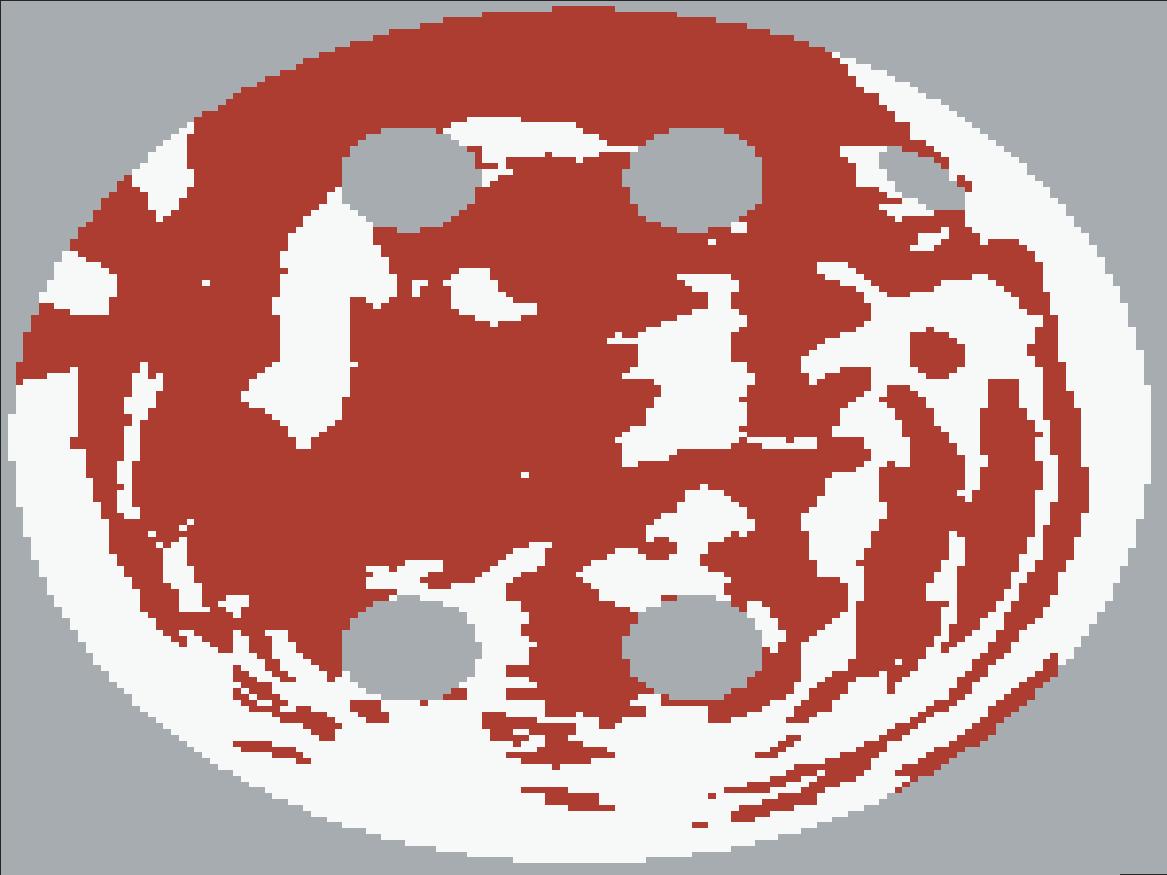

Supplement: Supplementary file 2 [file DataSheet1.ZIP › Dataset/real_6A0NU.jpg]

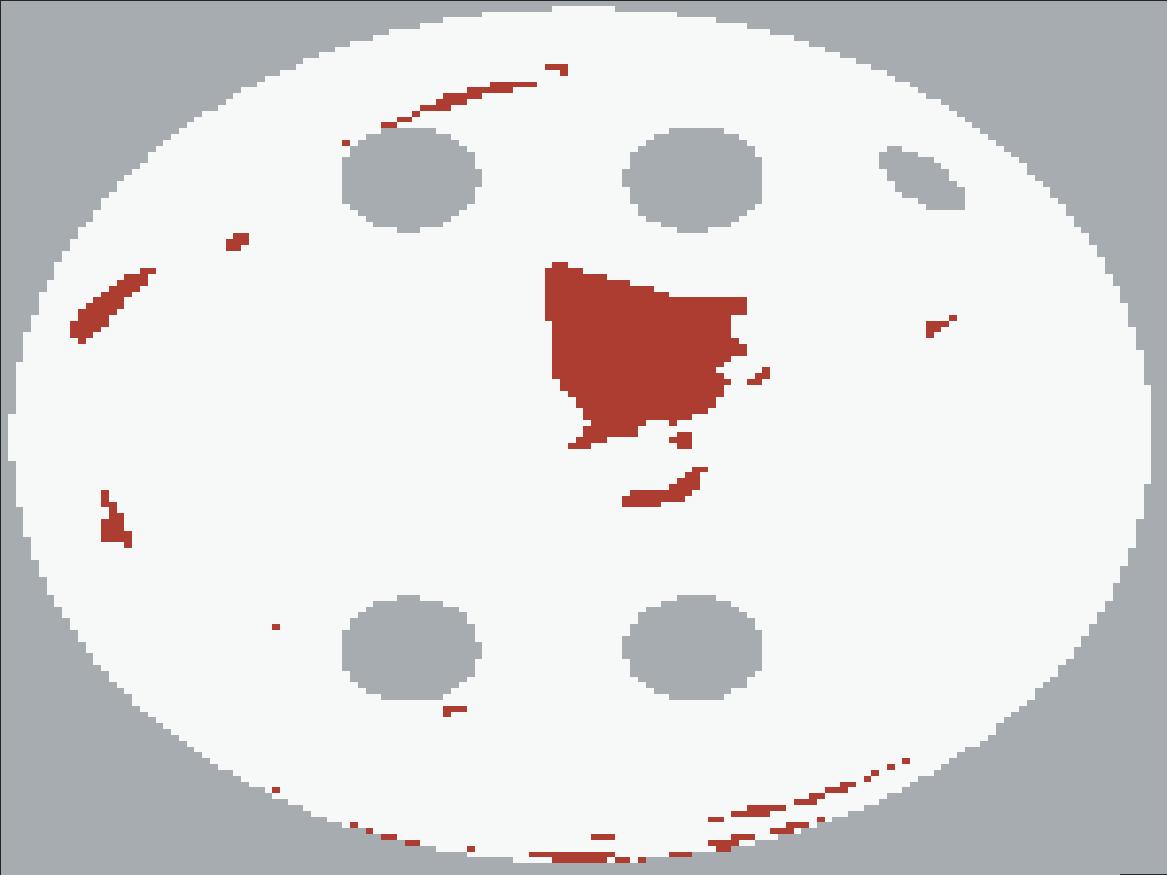

Supplement: Supplementary file 2 [file DataSheet1.ZIP › Dataset/real_6SSCP.jpg]

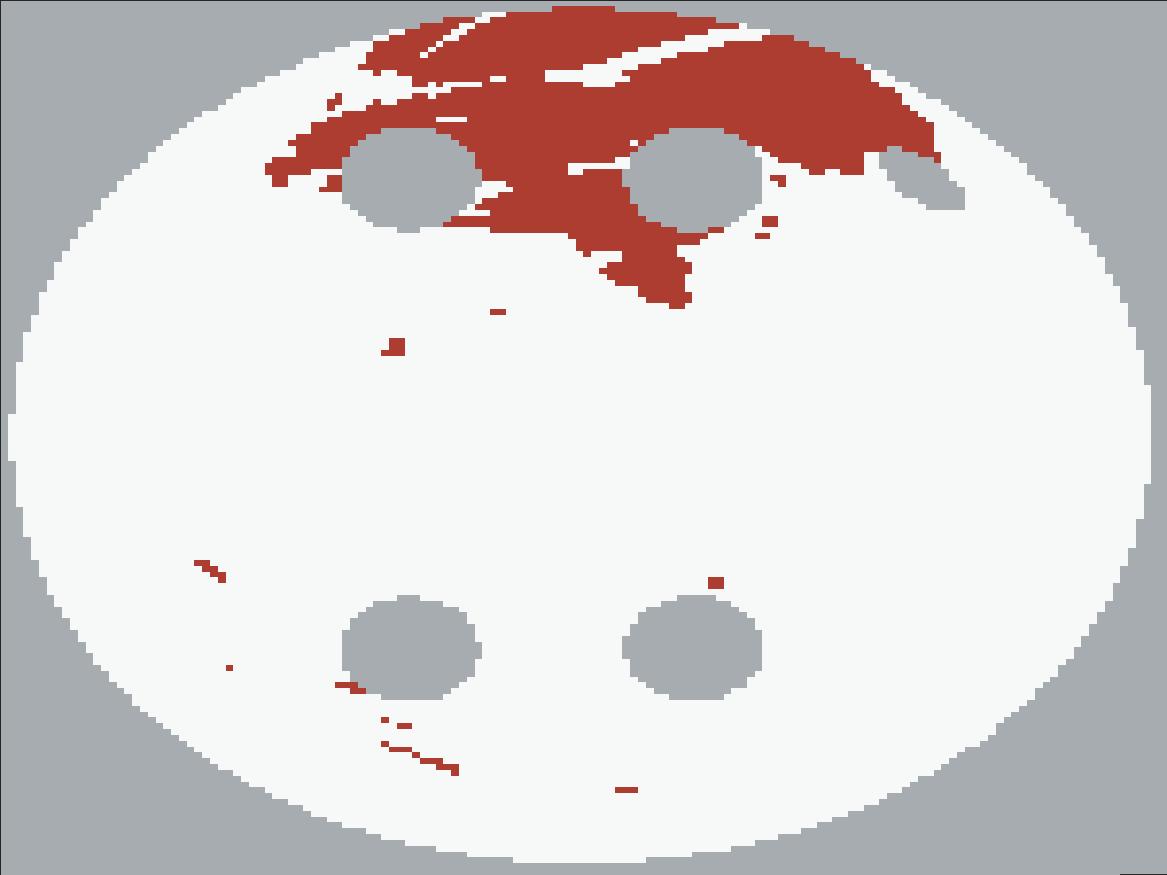

Supplement: Supplementary file 2 [file DataSheet1.ZIP › Dataset/real_6XESB.jpg]

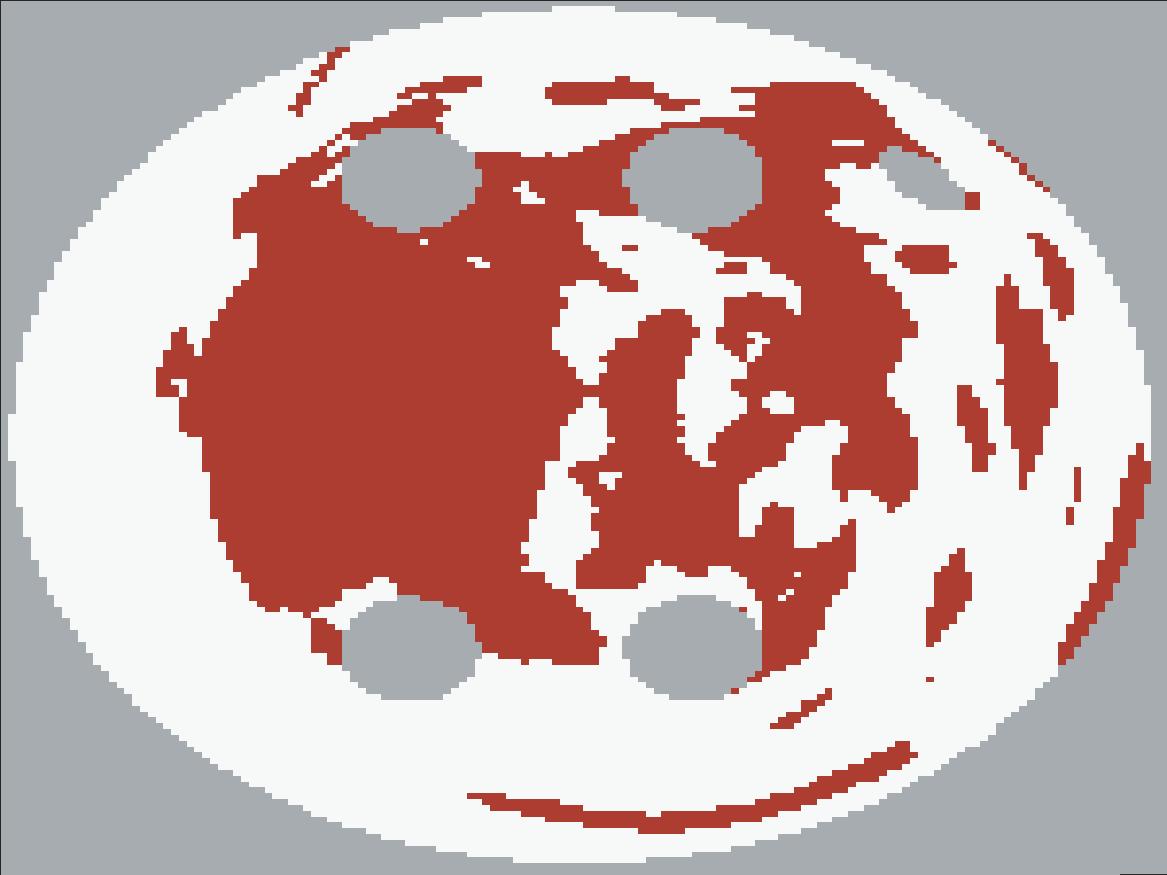

Supplement: Supplementary file 2 [file DataSheet1.ZIP › Dataset/real_75NPA.jpg]

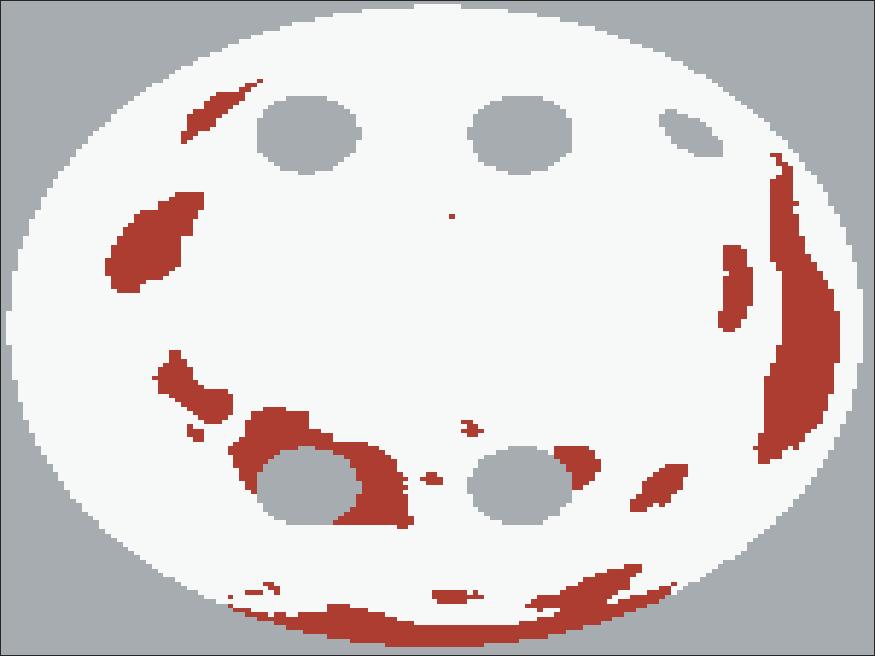

Supplement: Supplementary file 2 [file DataSheet1.ZIP › Dataset/real_7HJFD.jpg]

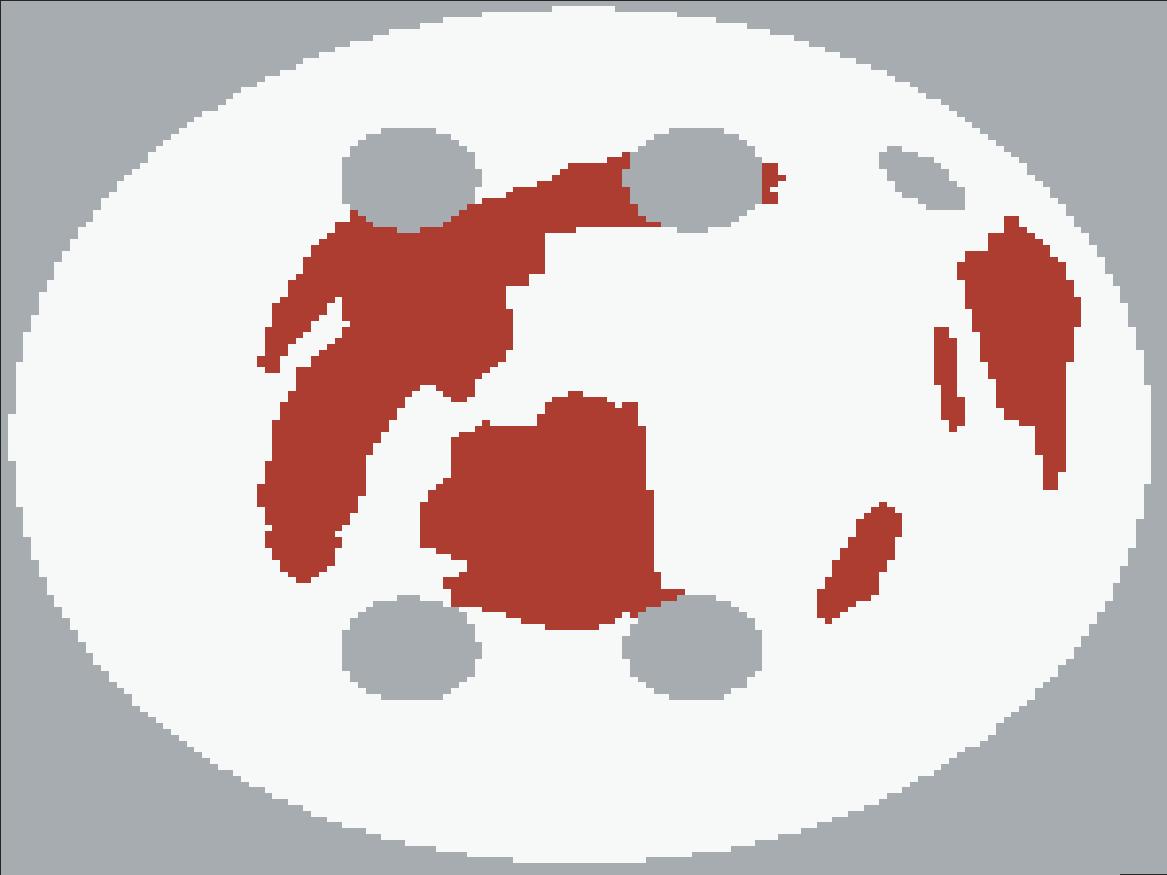

Supplement: Supplementary file 2 [file DataSheet1.ZIP › Dataset/real_7L6LG.jpg]

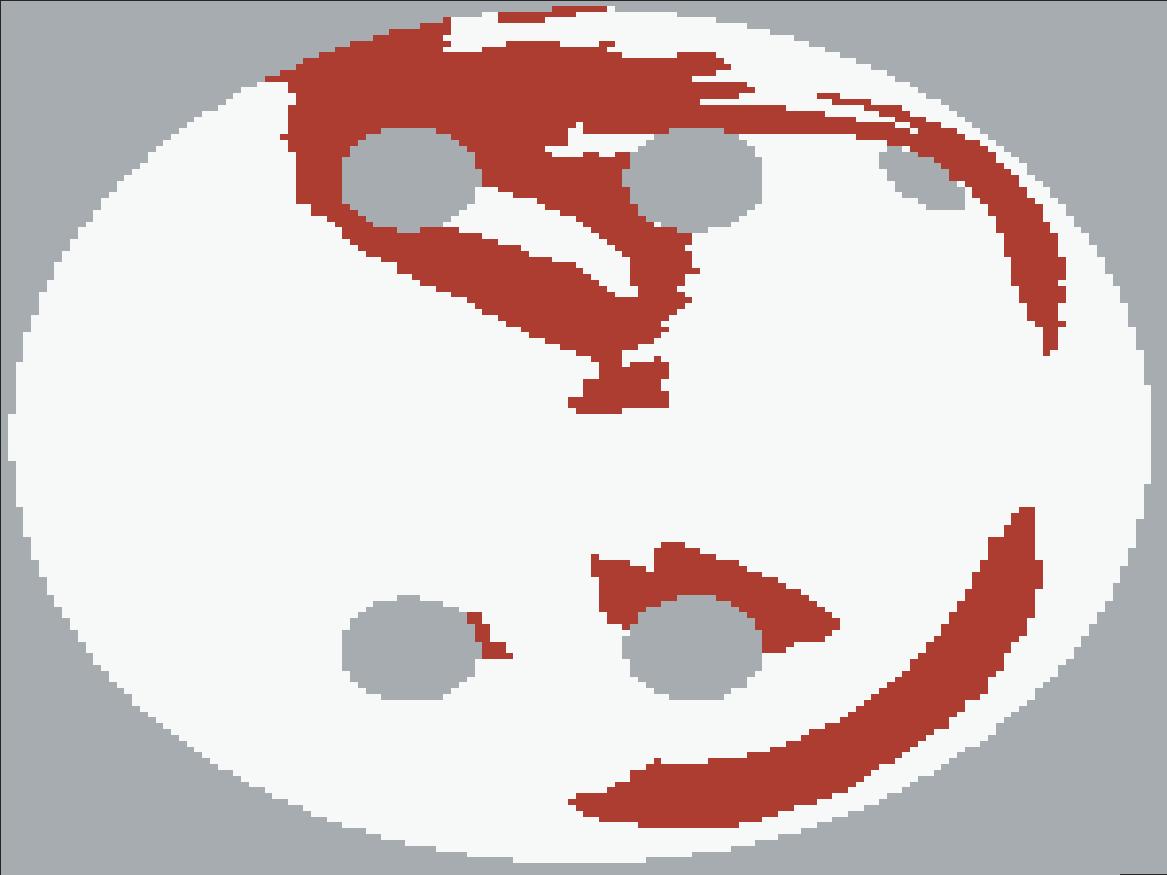

Supplement: Supplementary file 2 [file DataSheet1.ZIP › Dataset/real_7LBCC.jpg]

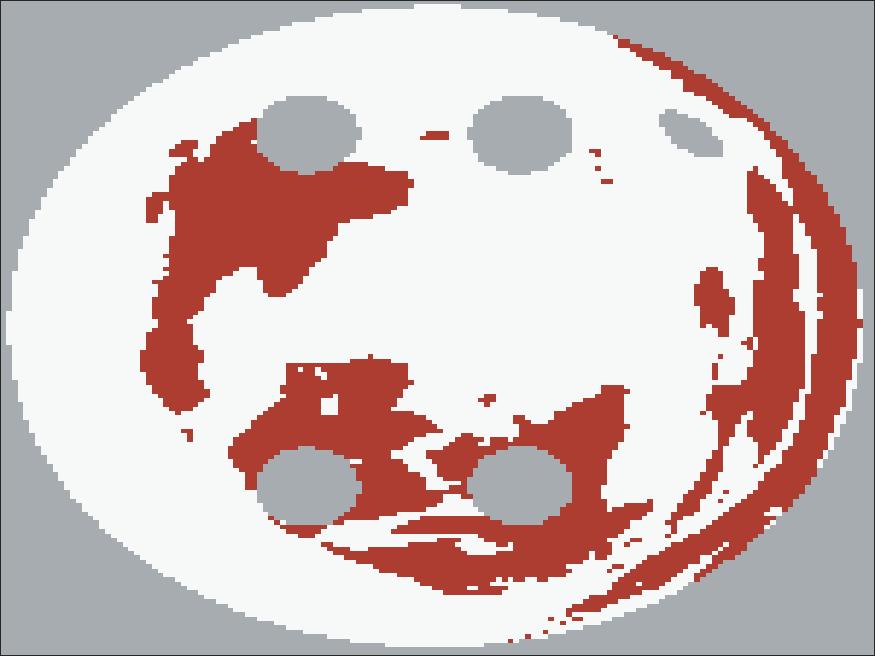

Supplement: Supplementary file 2 [file DataSheet1.ZIP › Dataset/real_7M7AE.jpg]

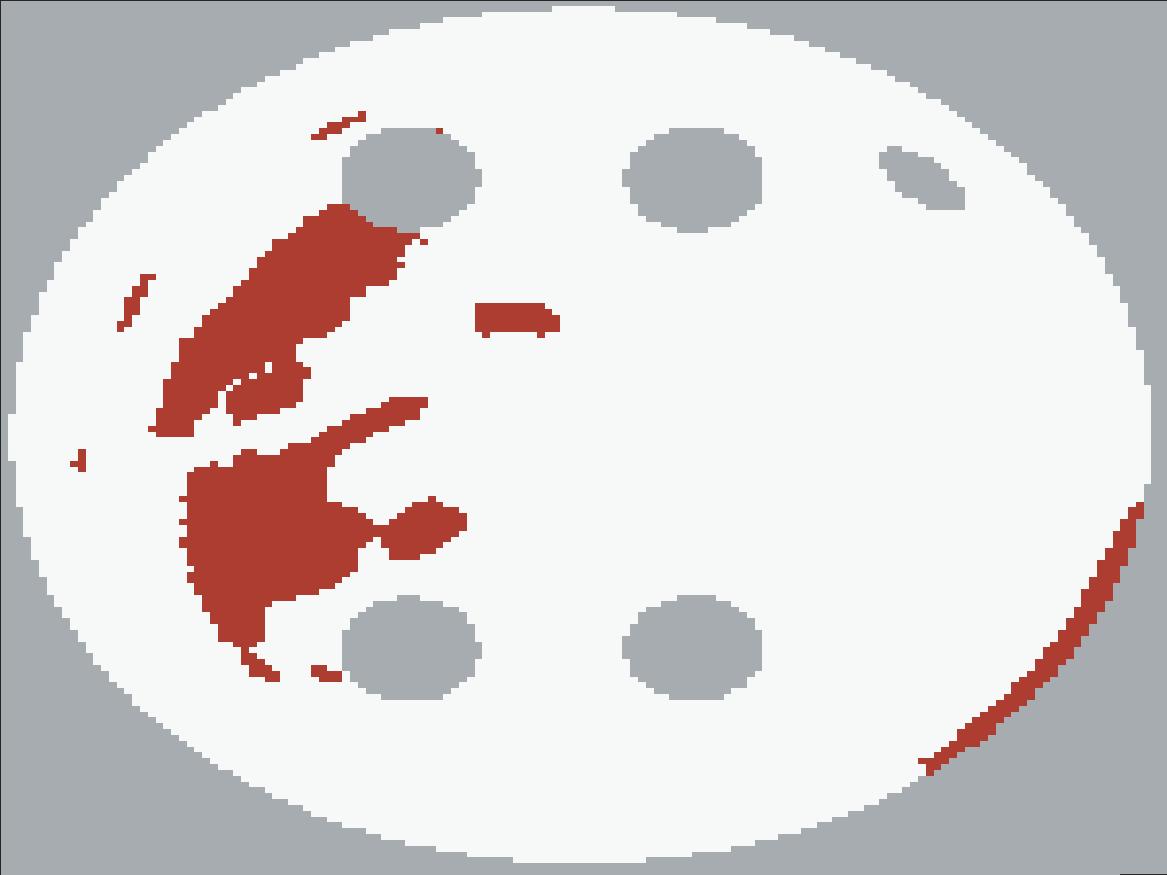

Supplement: Supplementary file 2 [file DataSheet1.ZIP › Dataset/real_7RGL0.jpg]

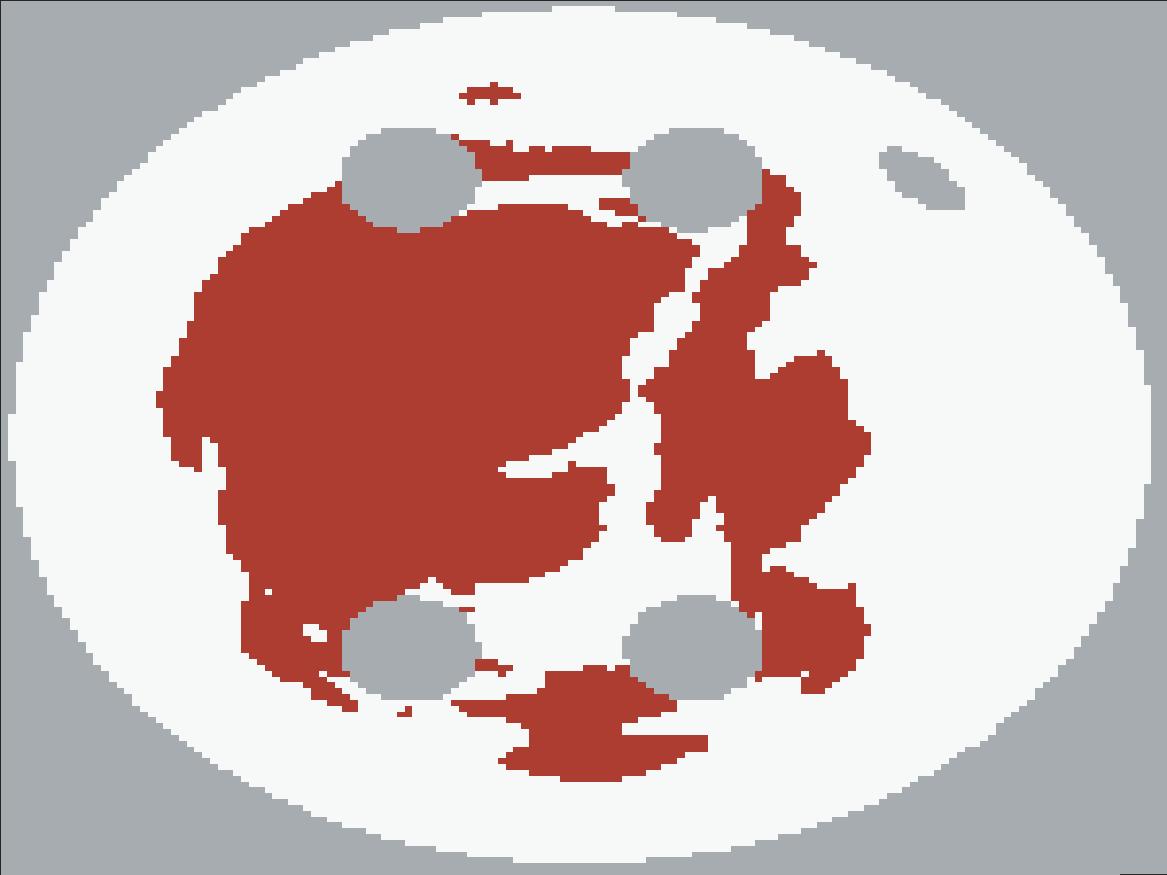

Supplement: Supplementary file 2 [file DataSheet1.ZIP › Dataset/real_87MHF.jpg]

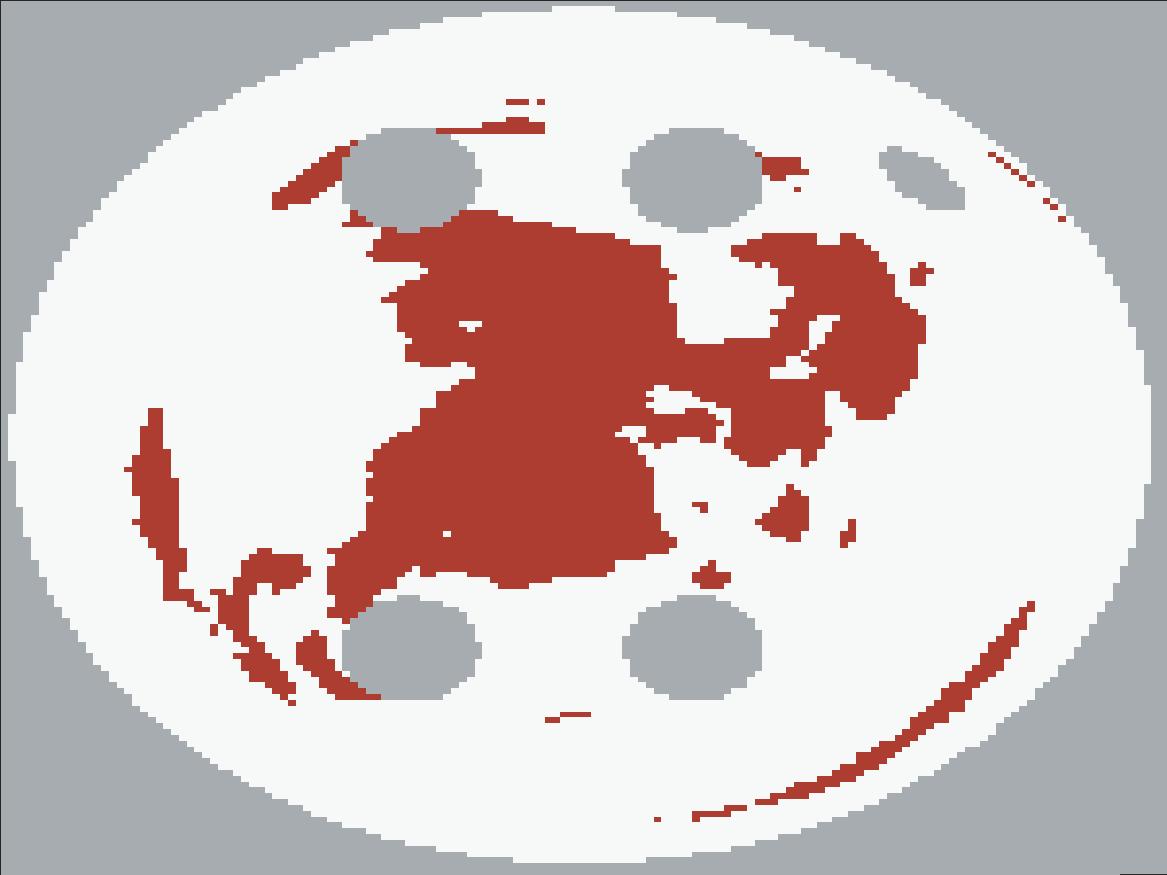

Supplement: Supplementary file 2 [file DataSheet1.ZIP › Dataset/real_89EFN.jpg]

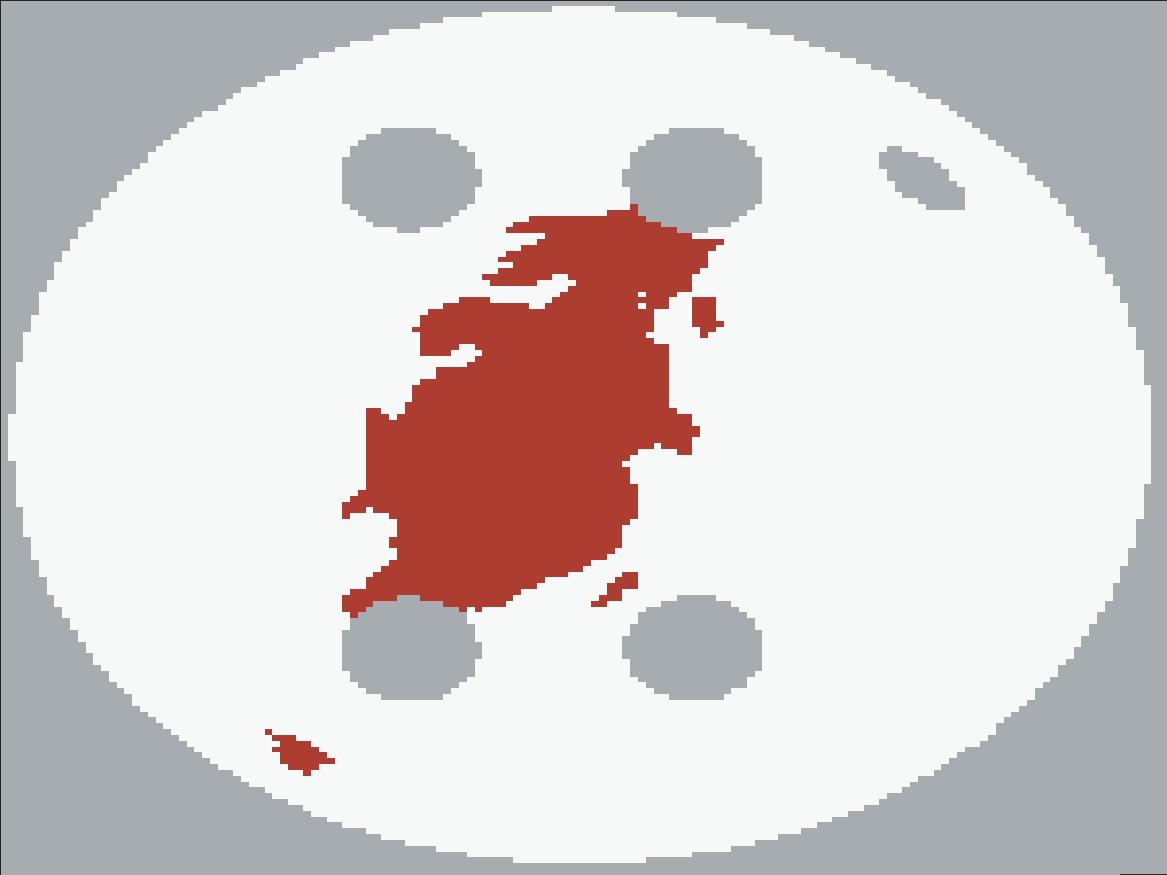

Supplement: Supplementary file 2 [file DataSheet1.ZIP › Dataset/real_8GINO.jpg]

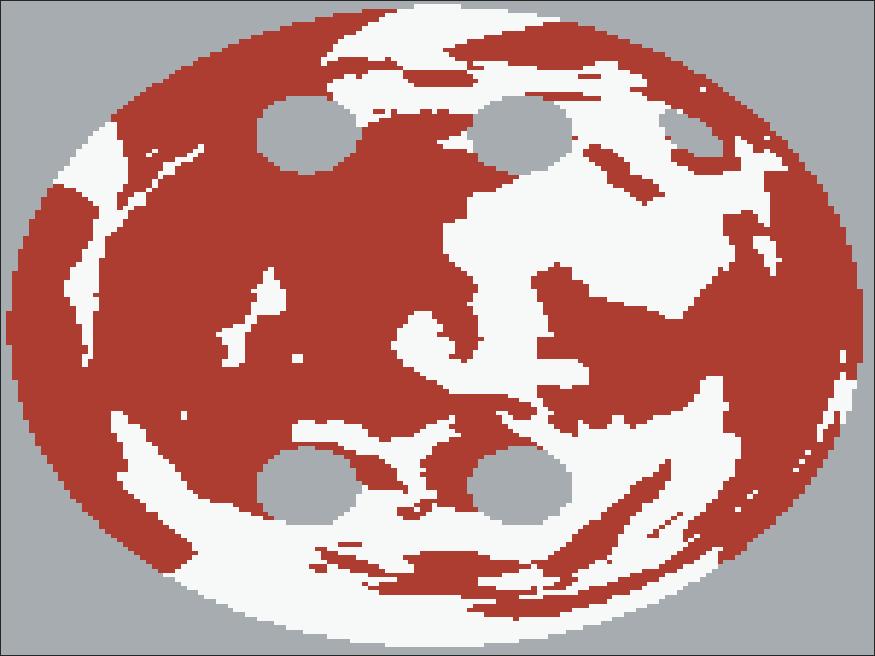

Supplement: Supplementary file 2 [file DataSheet1.ZIP › Dataset/real_8HFEE.jpg]

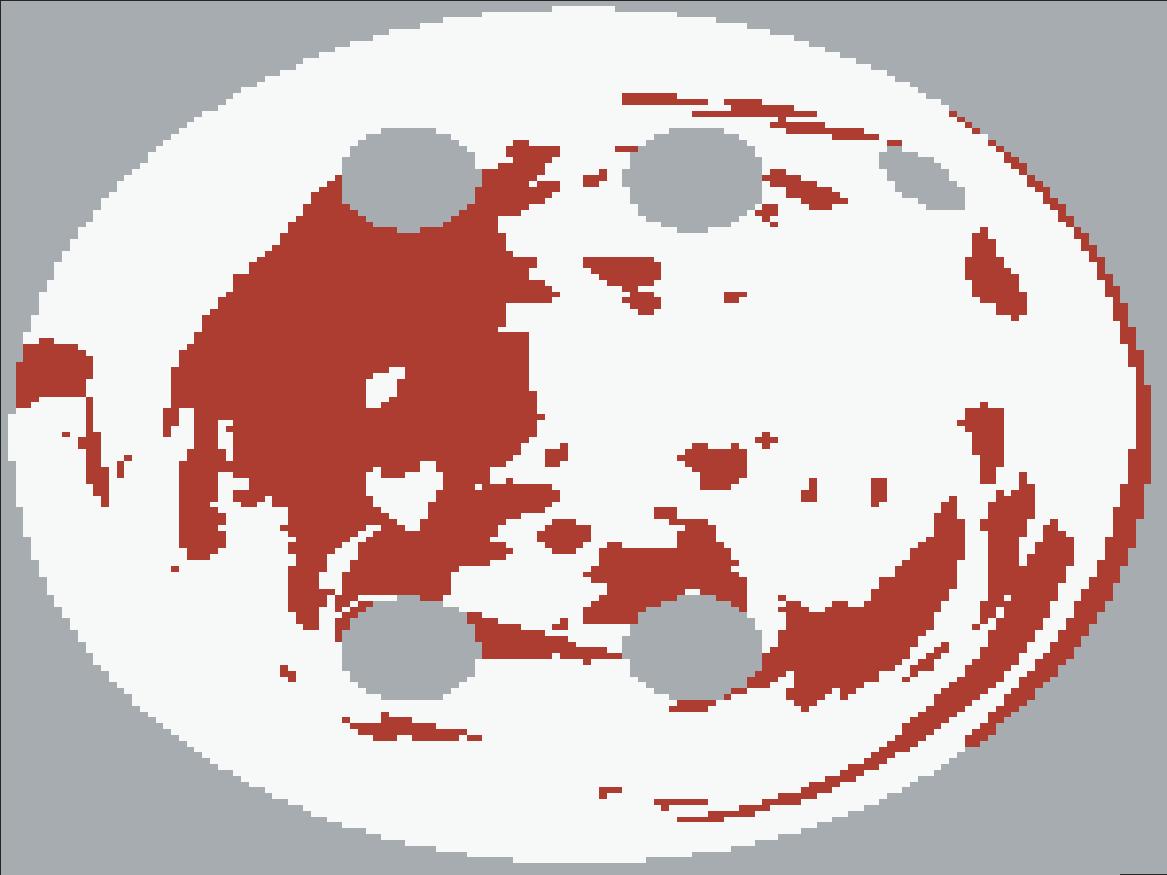

Supplement: Supplementary file 2 [file DataSheet1.ZIP › Dataset/real_8LDAW.jpg]

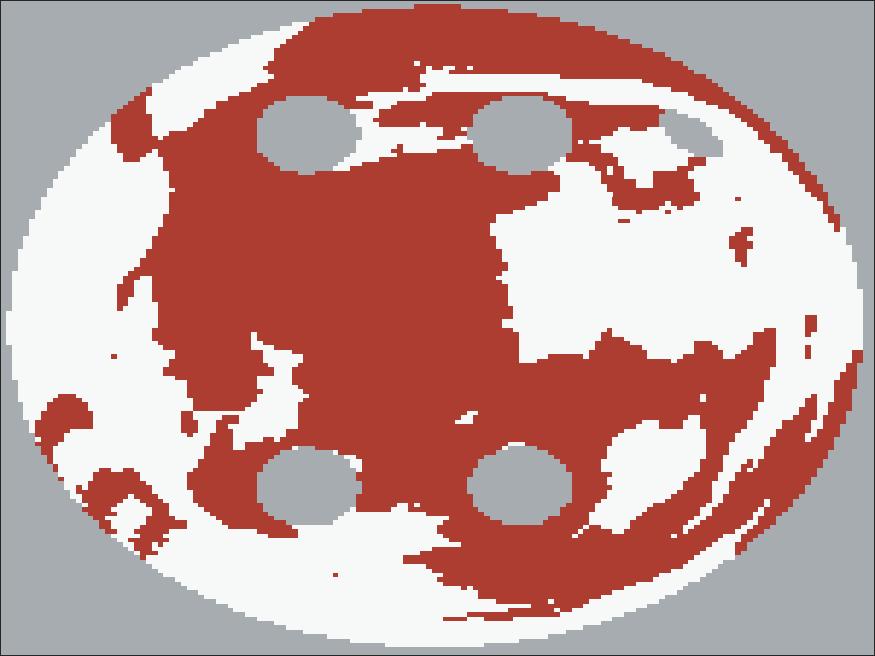

Supplement: Supplementary file 2 [file DataSheet1.ZIP › Dataset/real_8WM66.jpg]

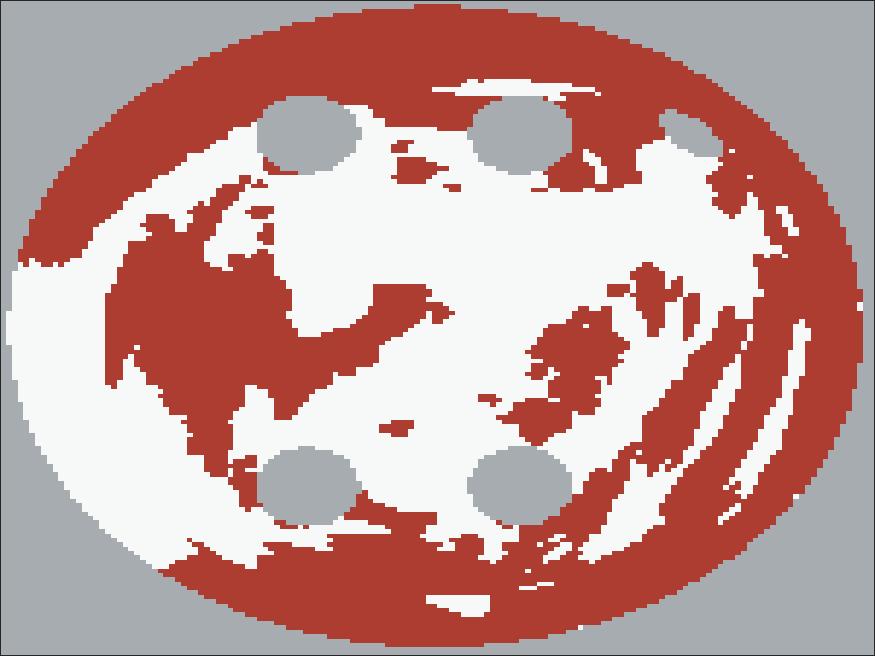

Supplement: Supplementary file 2 [file DataSheet1.ZIP › Dataset/real_97OYL.jpg]

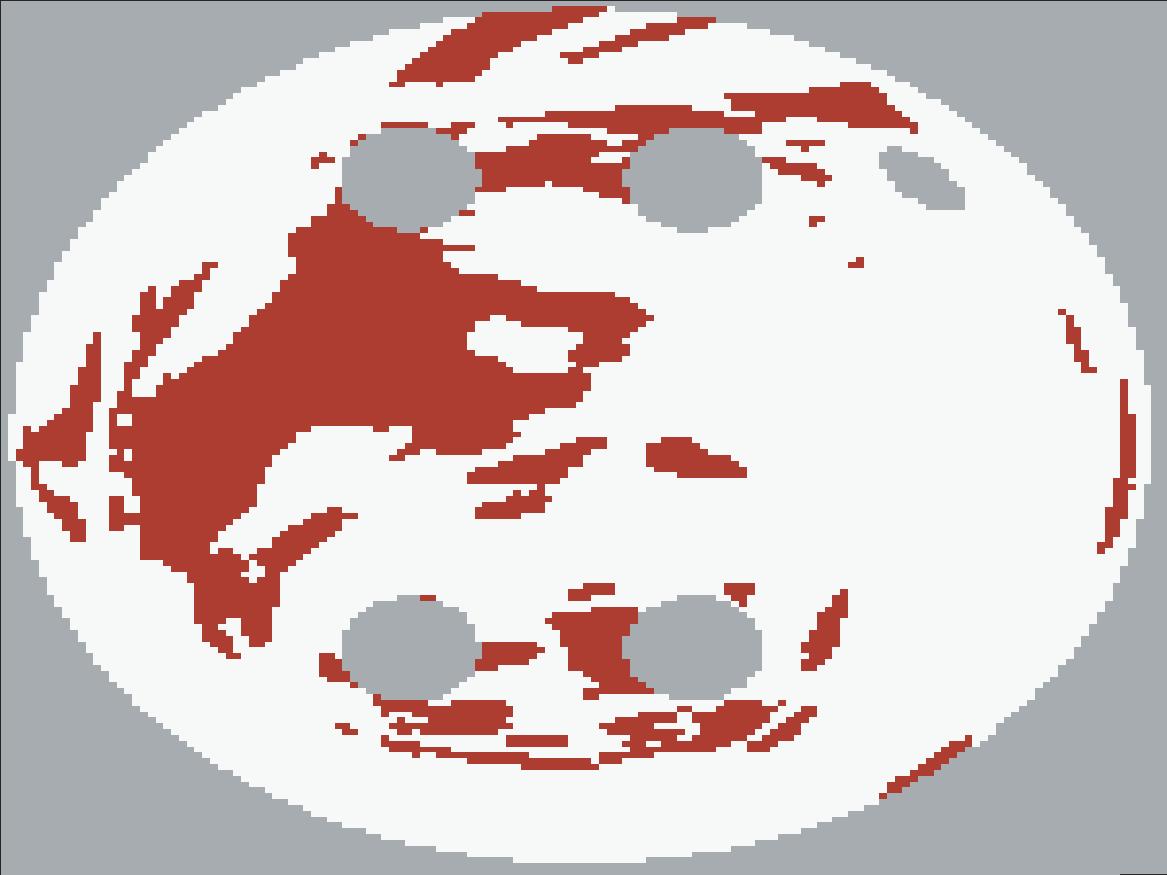

Supplement: Supplementary file 2 [file DataSheet1.ZIP › Dataset/real_9QOIO.jpg]

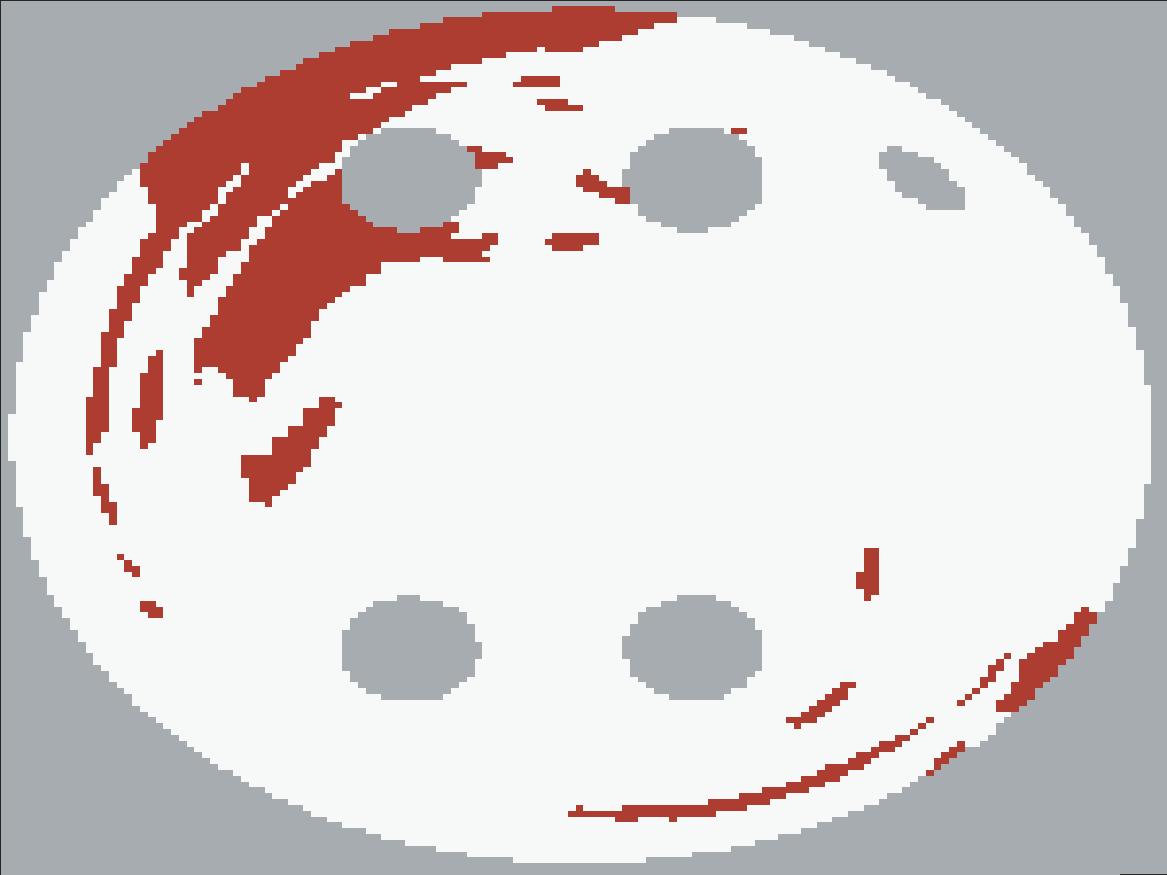

Supplement: Supplementary file 2 [file DataSheet1.ZIP › Dataset/real_9TGDE.jpg]

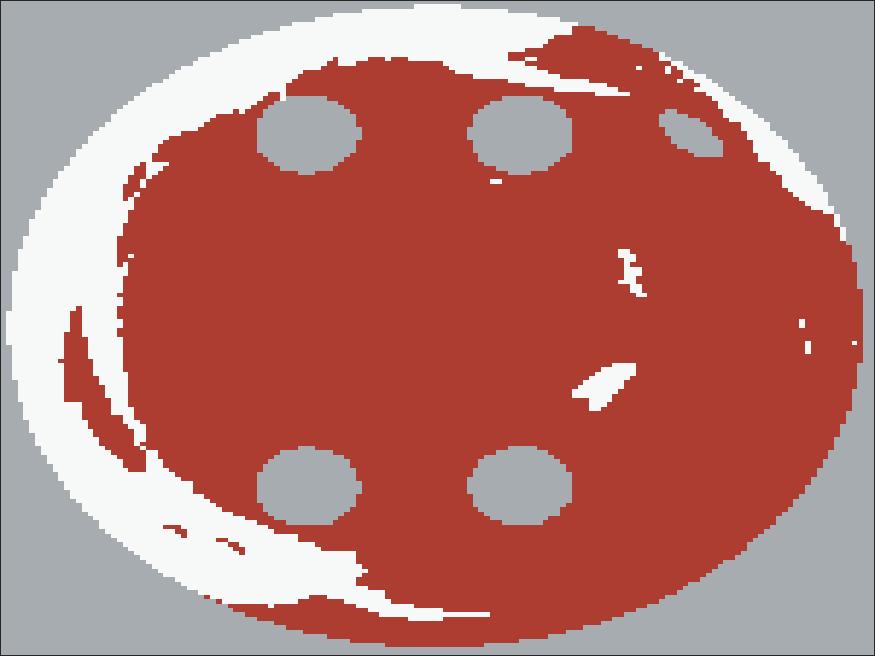

Supplement: Supplementary file 2 [file DataSheet1.ZIP › Dataset/real_9WNBH.jpg]

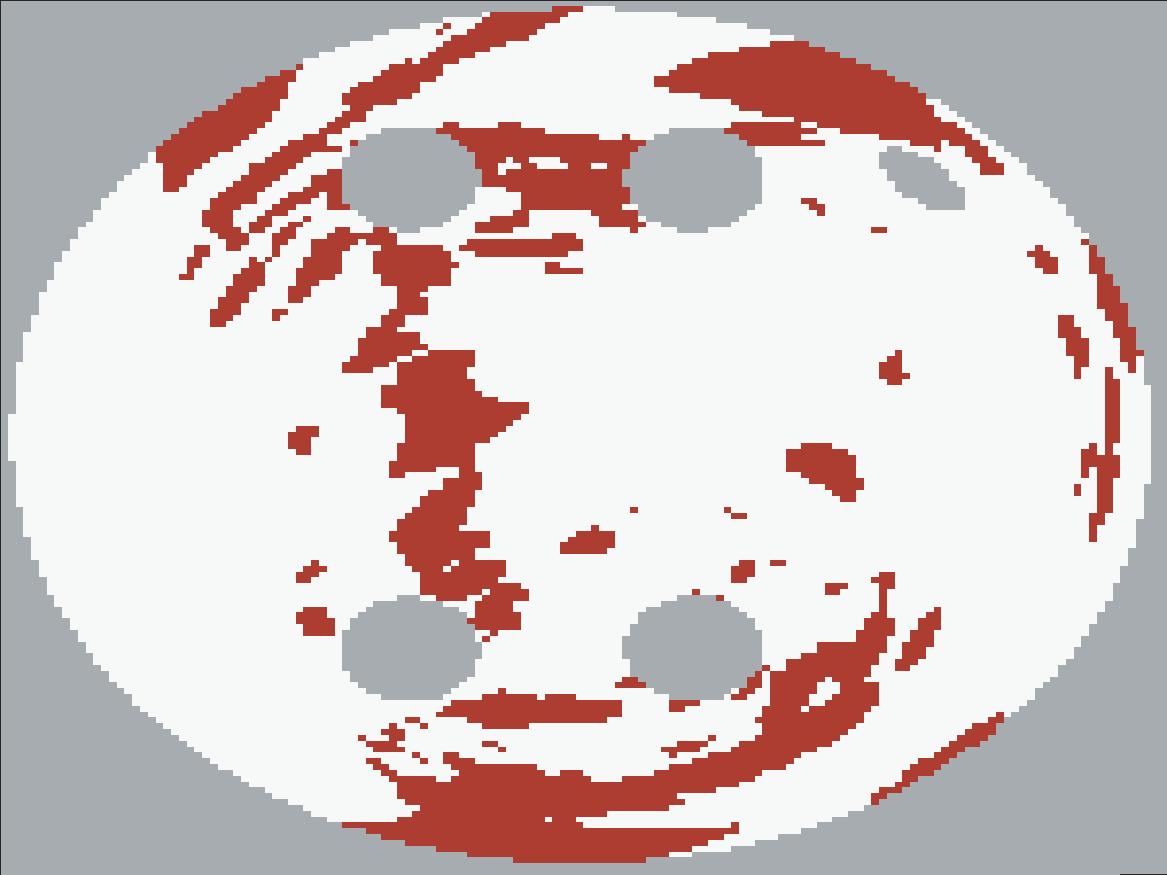

Supplement: Supplementary file 2 [file DataSheet1.ZIP › Dataset/real_9Y68E.jpg]

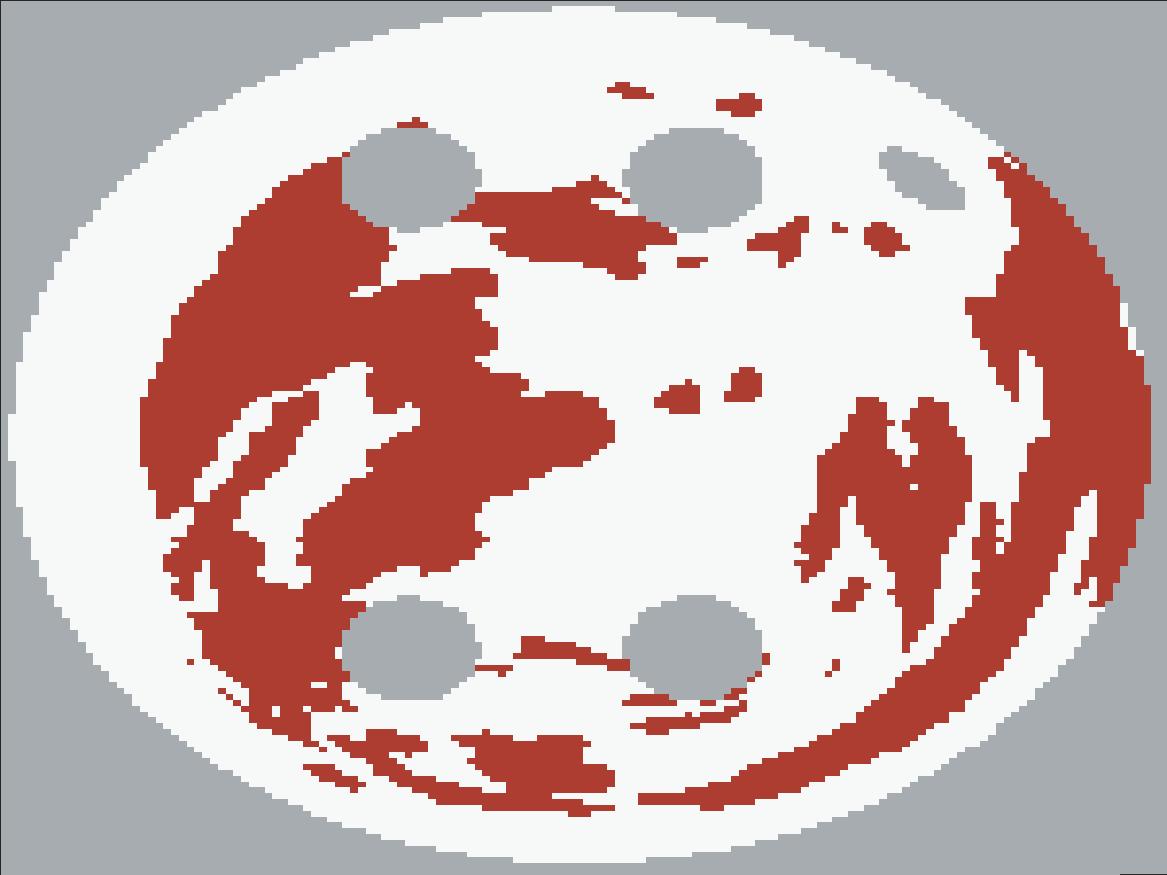

Supplement: Supplementary file 2 [file DataSheet1.ZIP › Dataset/real_AEV8W.jpg]

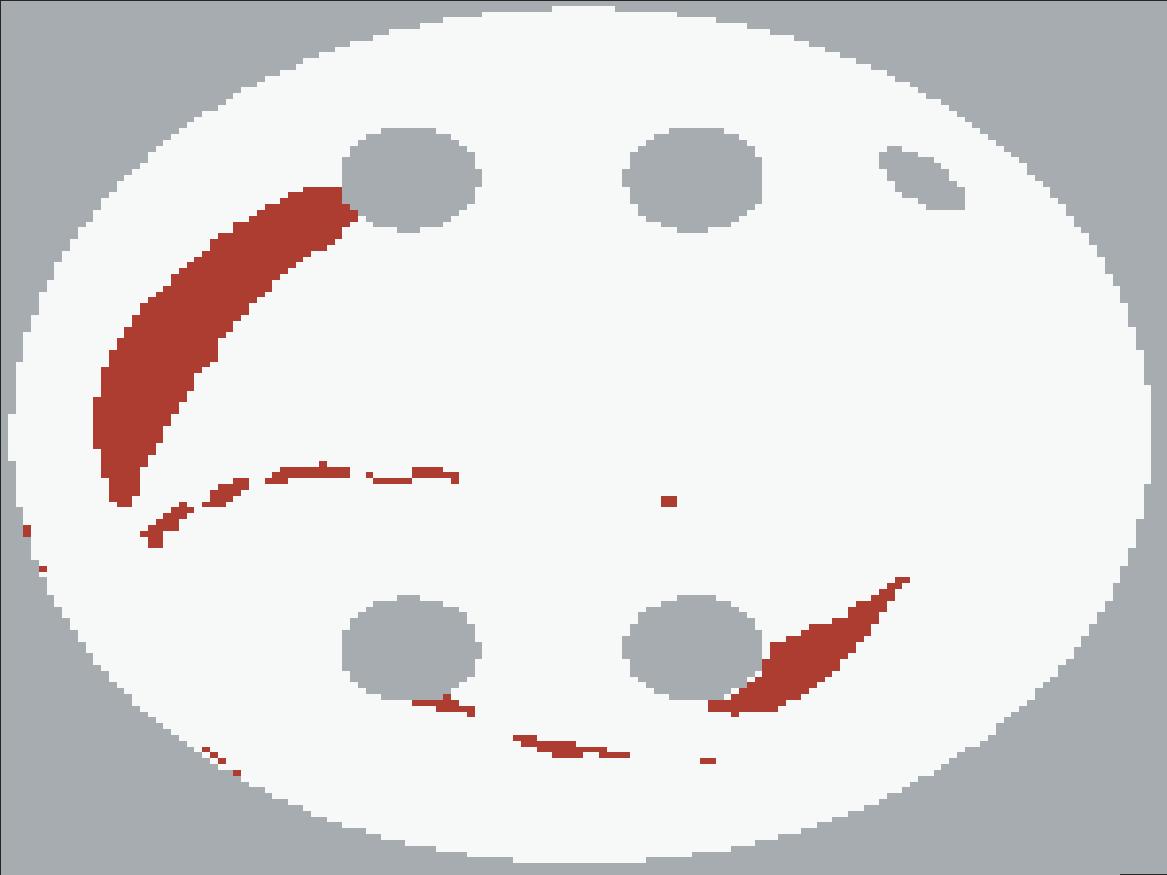

Supplement: Supplementary file 2 [file DataSheet1.ZIP › Dataset/real_C3DNO.jpg]

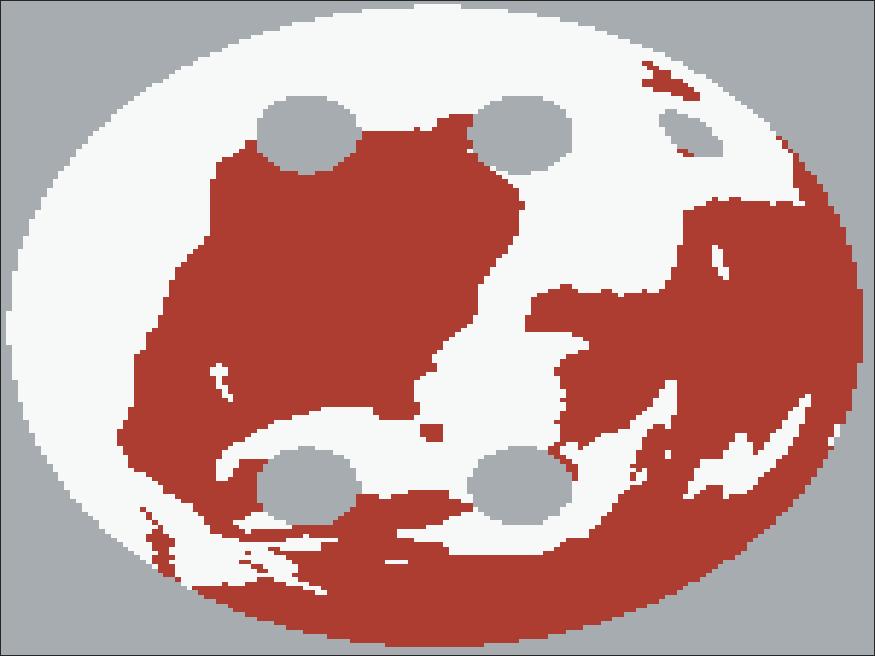

Supplement: Supplementary file 2 [file DataSheet1.ZIP › Dataset/real_CMSSG.jpg]

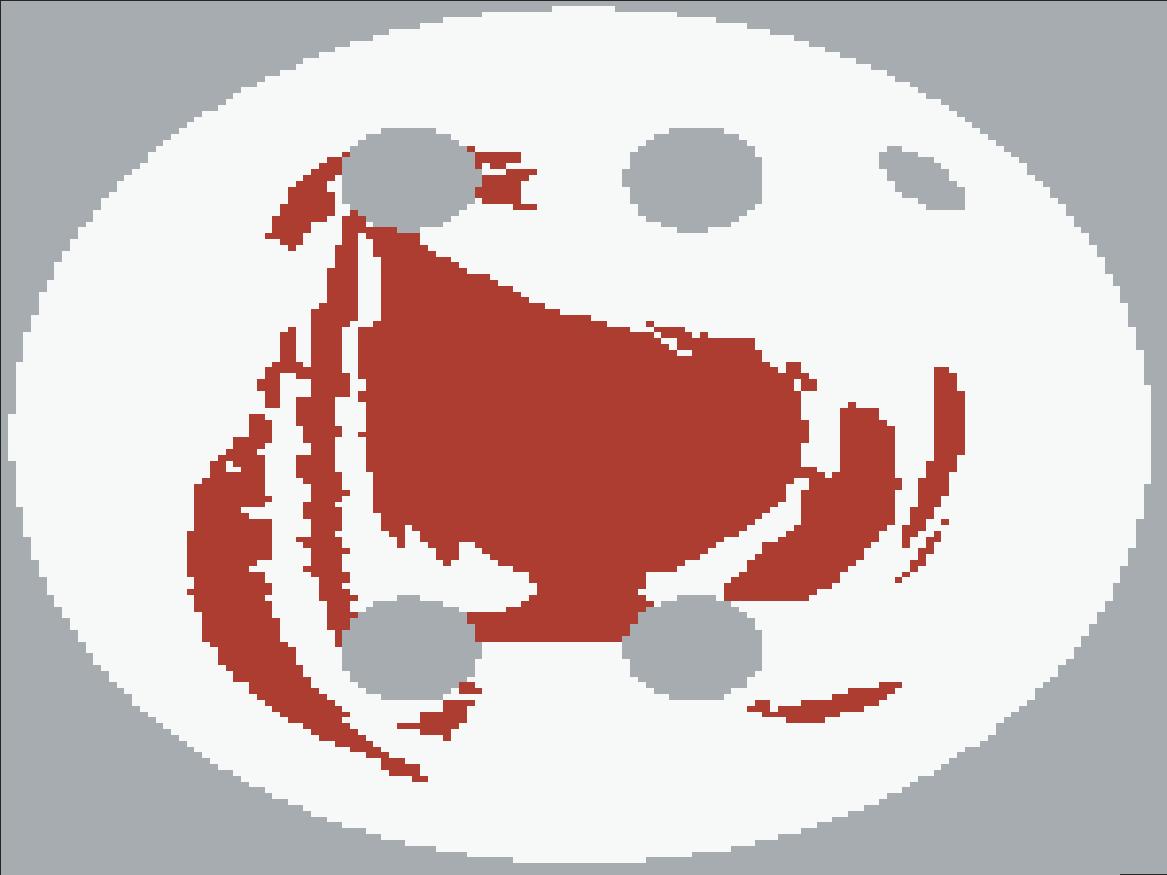

Supplement: Supplementary file 2 [file DataSheet1.ZIP › Dataset/real_CO4XE.jpg]

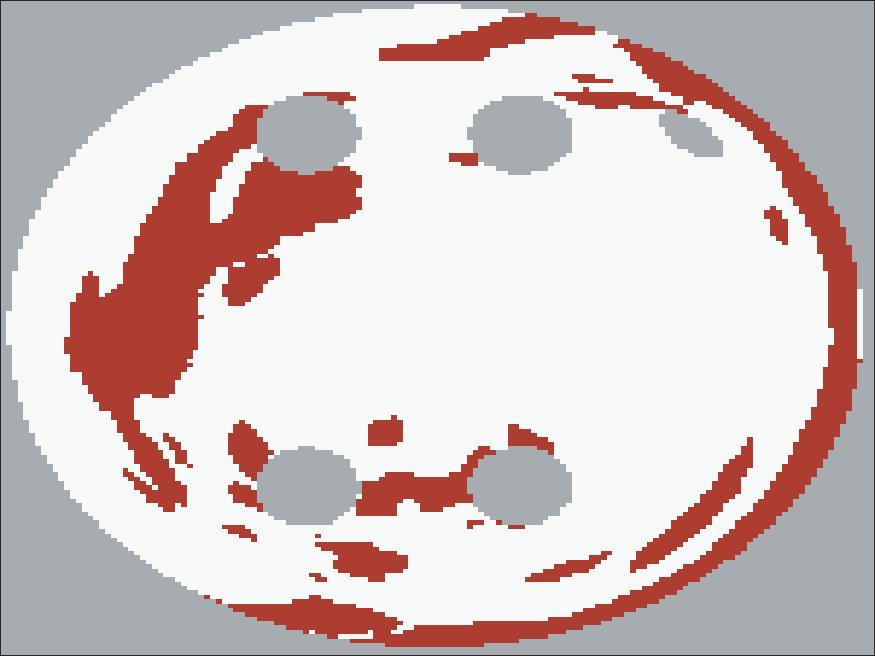

Supplement: Supplementary file 2 [file DataSheet1.ZIP › Dataset/real_D1VCK.jpg]

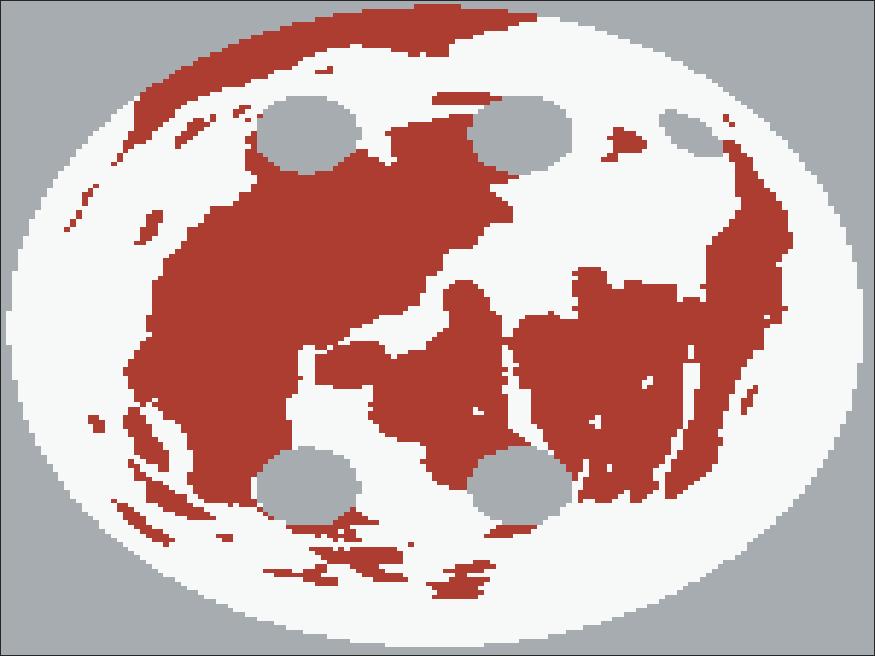

Supplement: Supplementary file 2 [file DataSheet1.ZIP › Dataset/real_D8N4Z.jpg]

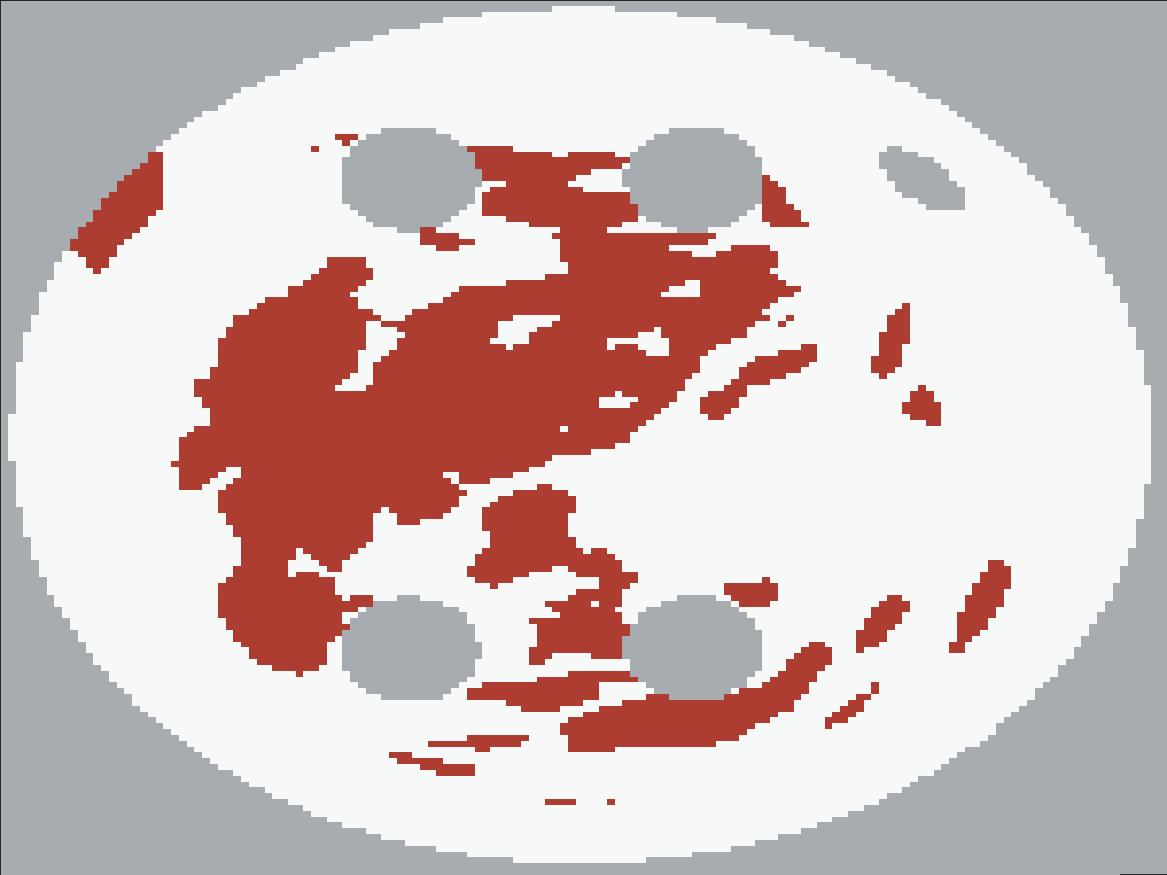

Supplement: Supplementary file 2 [file DataSheet1.ZIP › Dataset/real_DKC38.jpg]

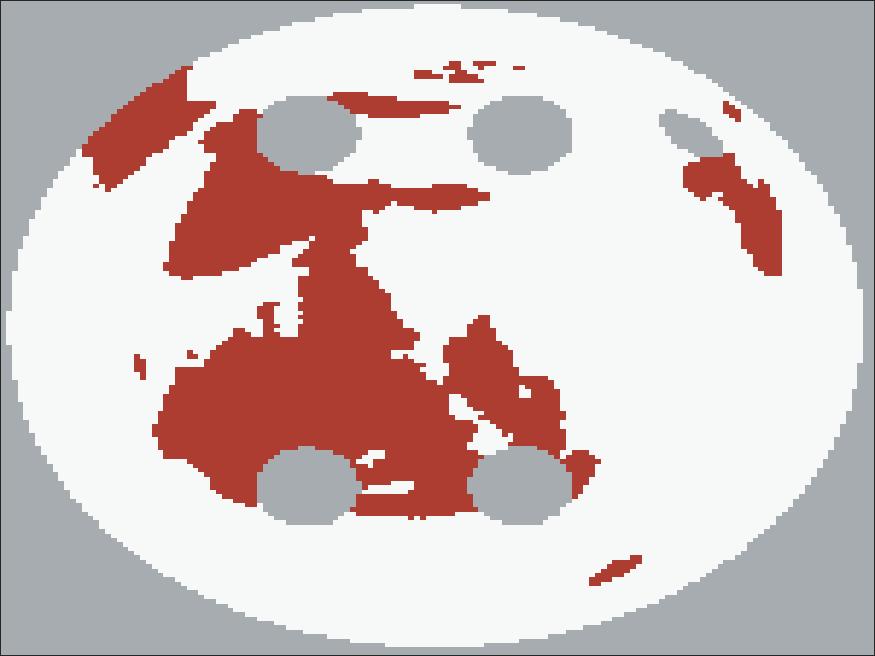

Supplement: Supplementary file 2 [file DataSheet1.ZIP › Dataset/real_DMS8G.jpg]

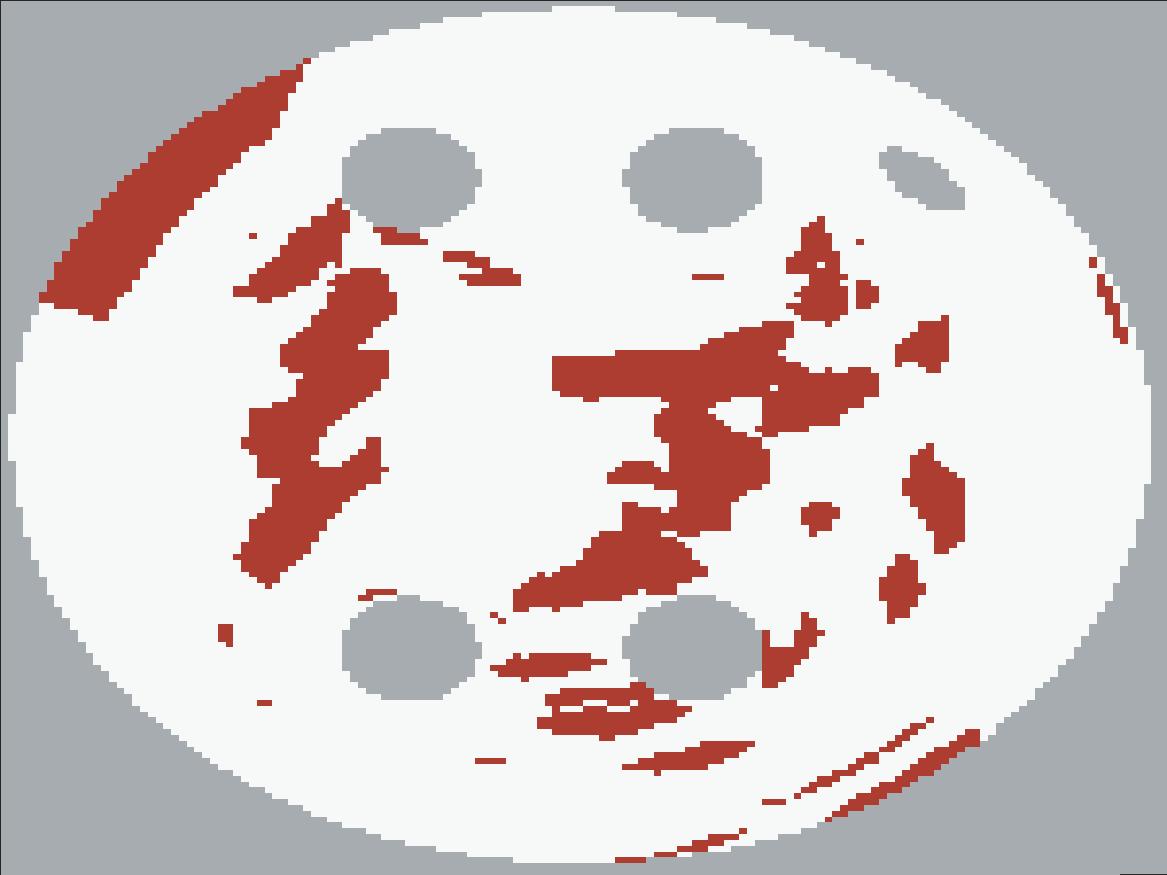

Supplement: Supplementary file 2 [file DataSheet1.ZIP › Dataset/real_E925G.jpg]

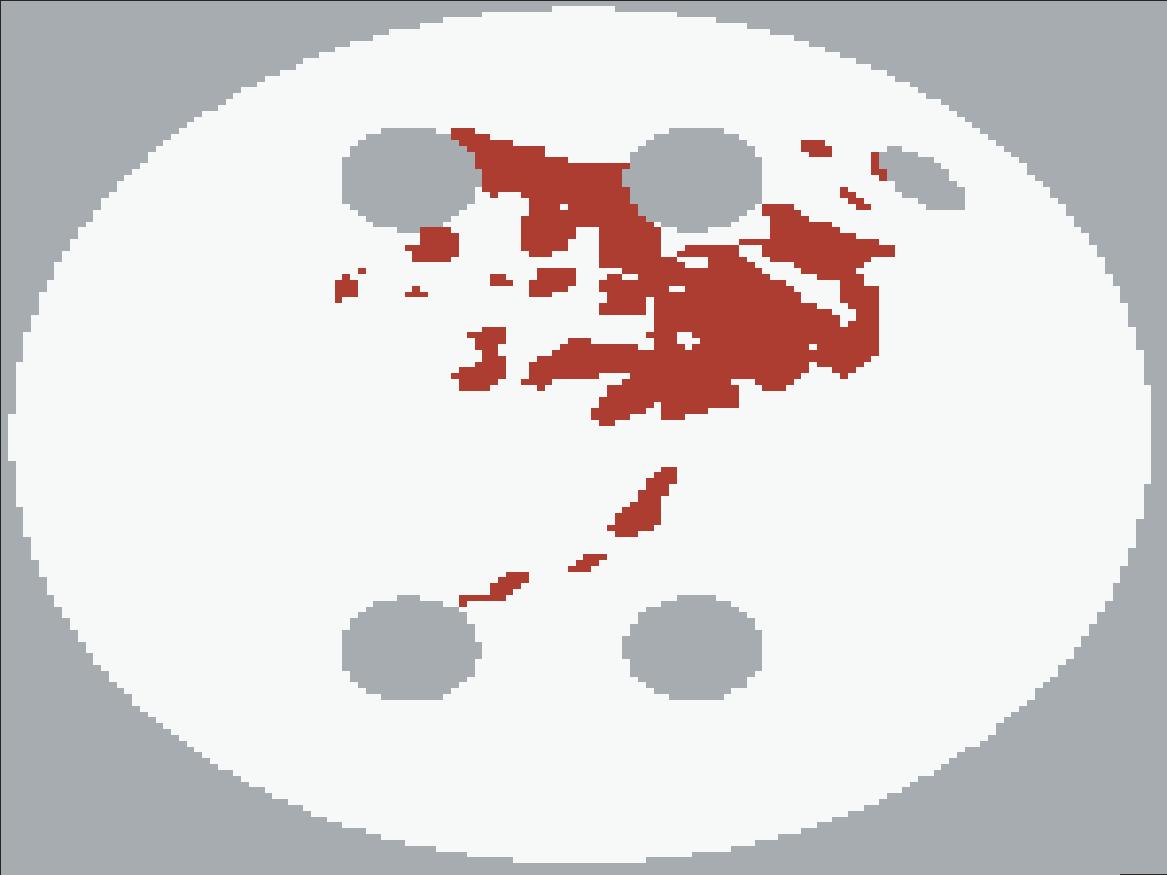

Supplement: Supplementary file 2 [file DataSheet1.ZIP › Dataset/real_EMZOT.jpg]

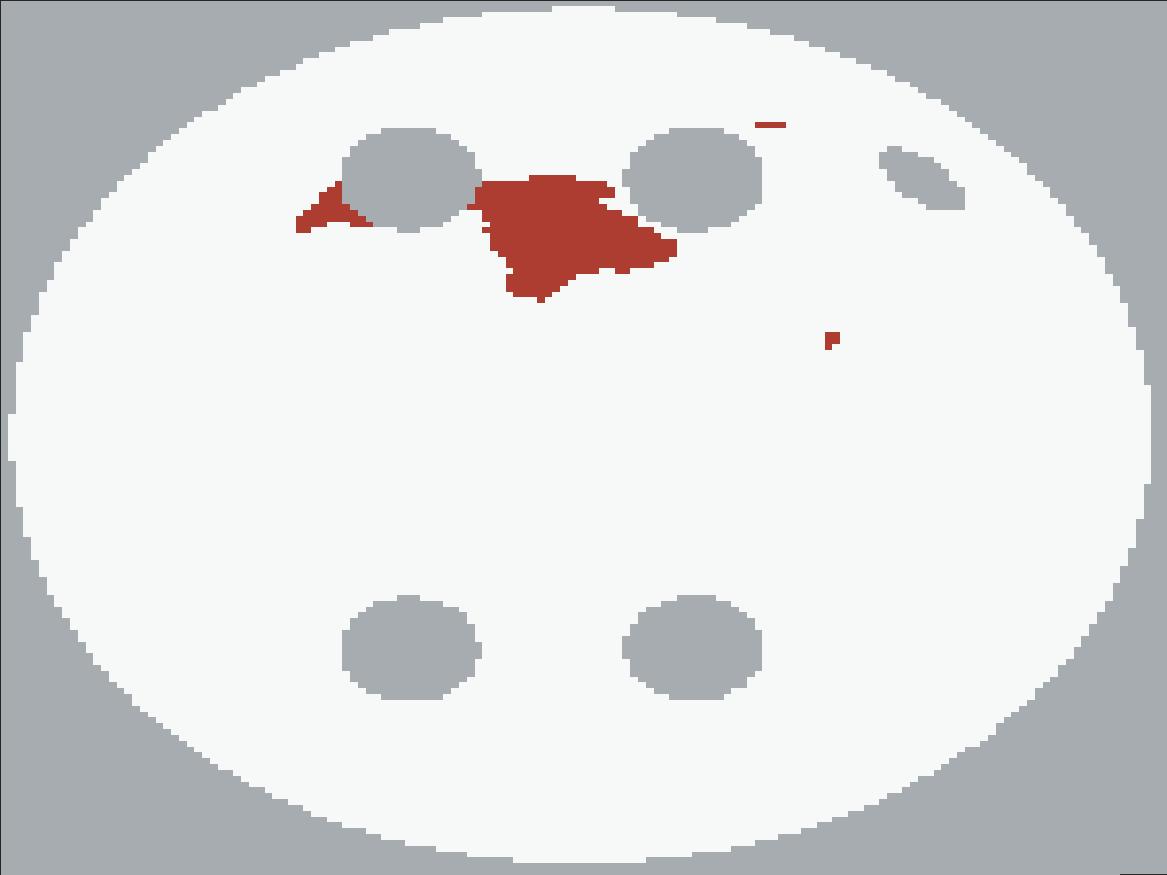

Supplement: Supplementary file 2 [file DataSheet1.ZIP › Dataset/real_EYOLI.jpg]

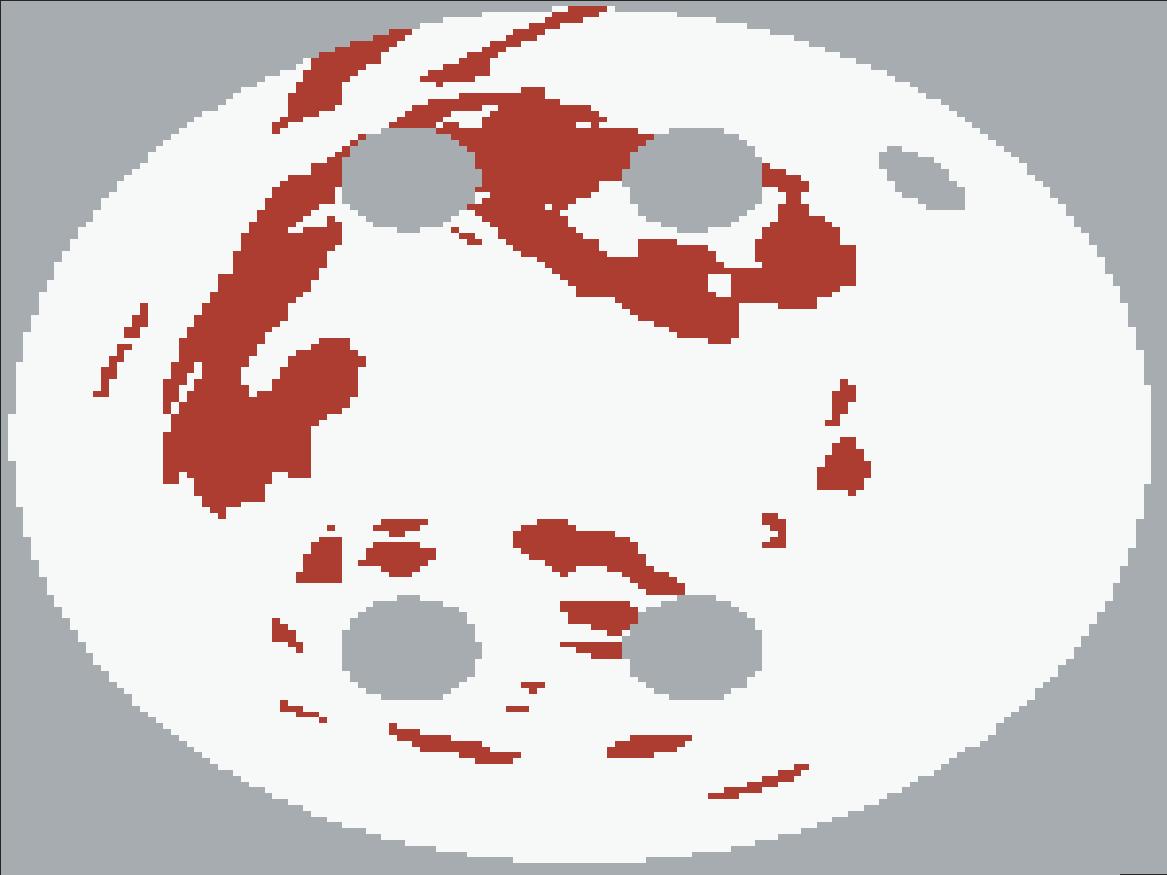

Supplement: Supplementary file 2 [file DataSheet1.ZIP › Dataset/real_F4AGS.jpg]

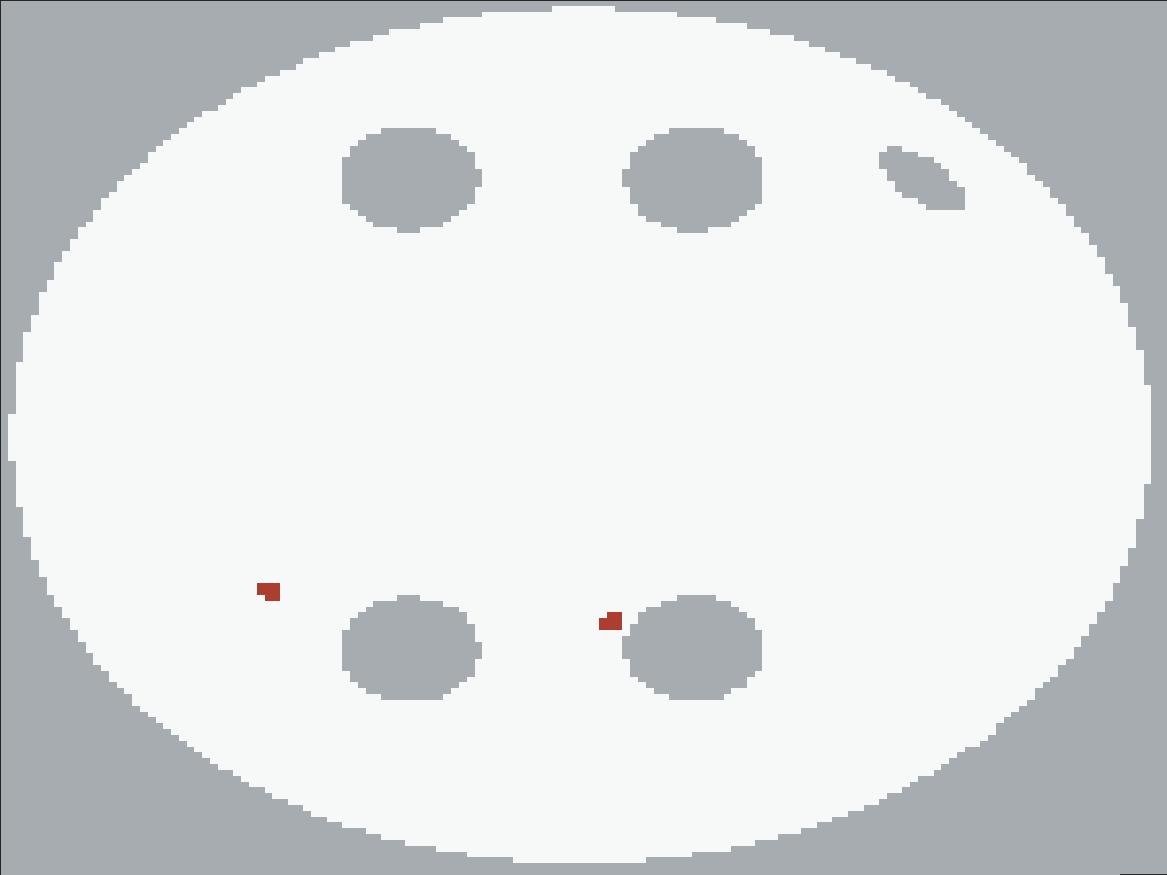

Supplement: Supplementary file 2 [file DataSheet1.ZIP › Dataset/real_F73F8.jpg]

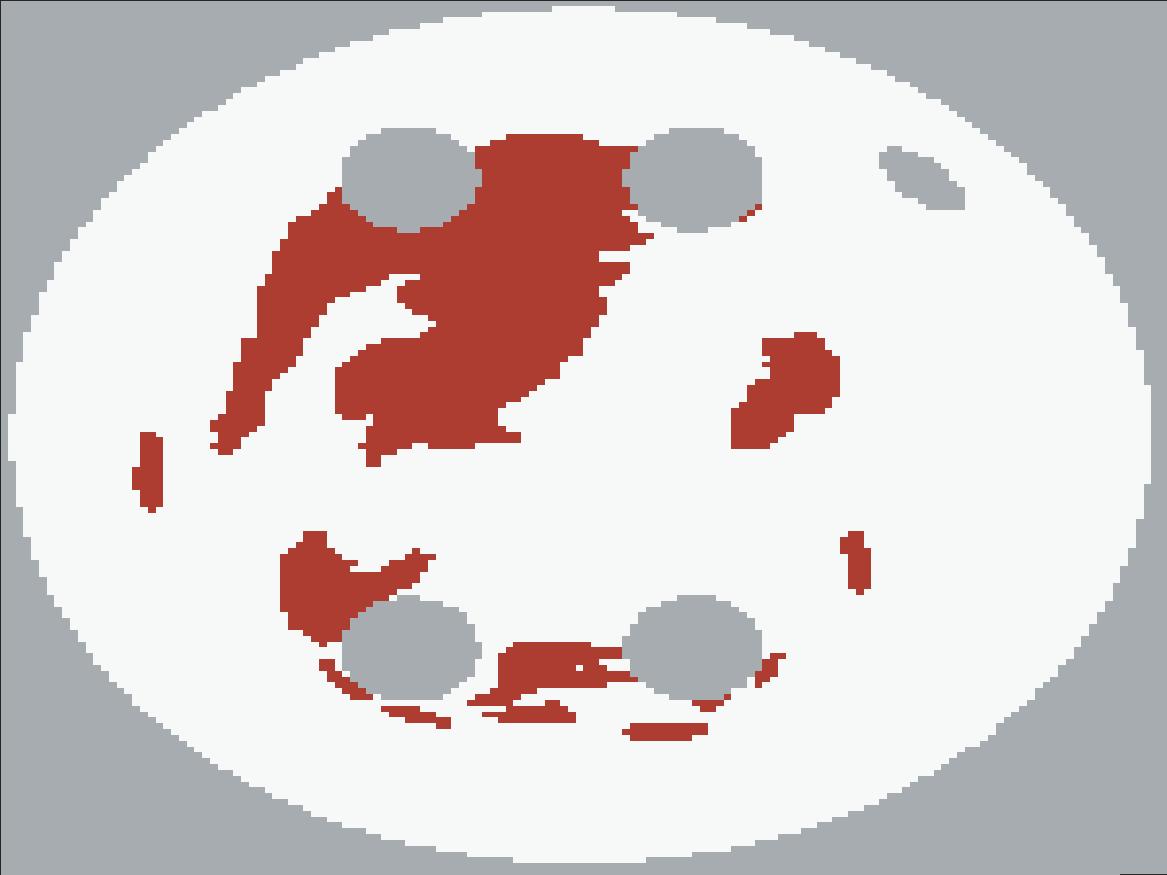

Supplement: Supplementary file 2 [file DataSheet1.ZIP › Dataset/real_F8OGT.jpg]

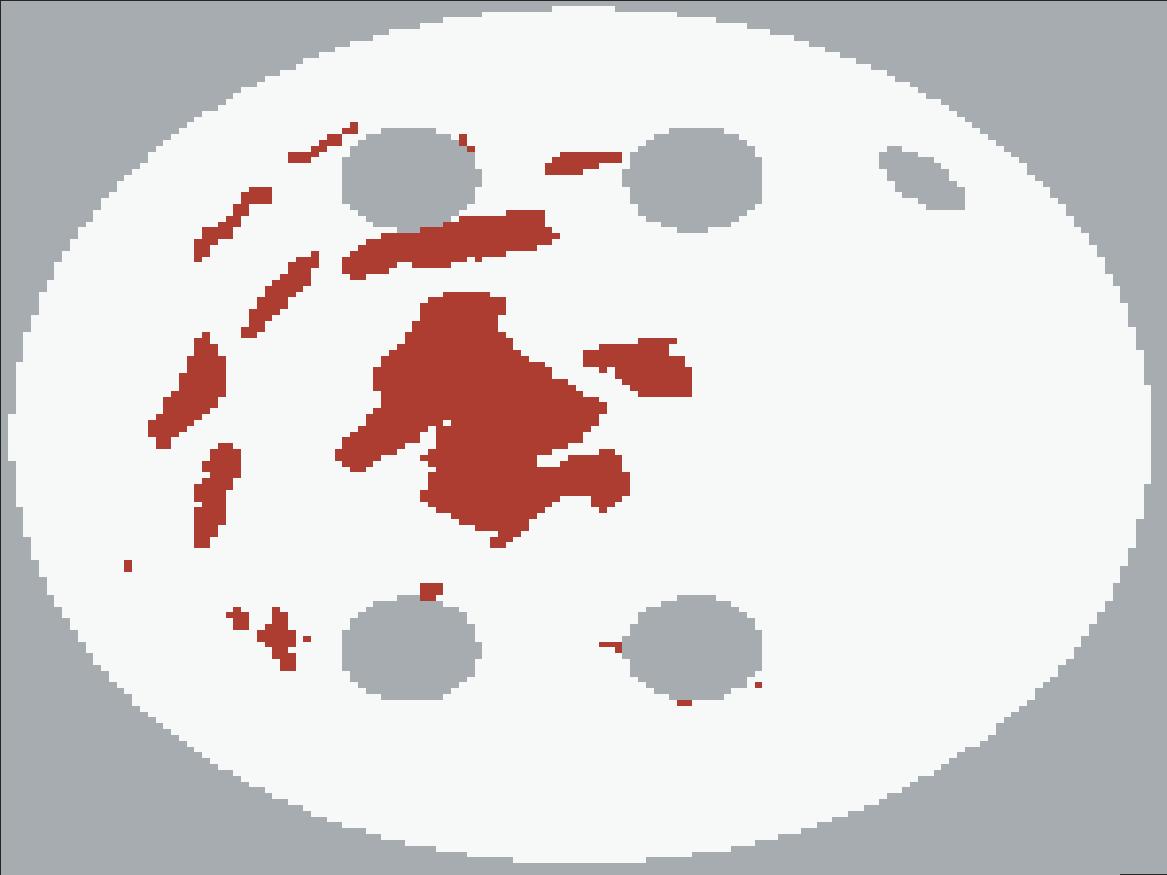

Supplement: Supplementary file 2 [file DataSheet1.ZIP › Dataset/real_FB46F.jpg]

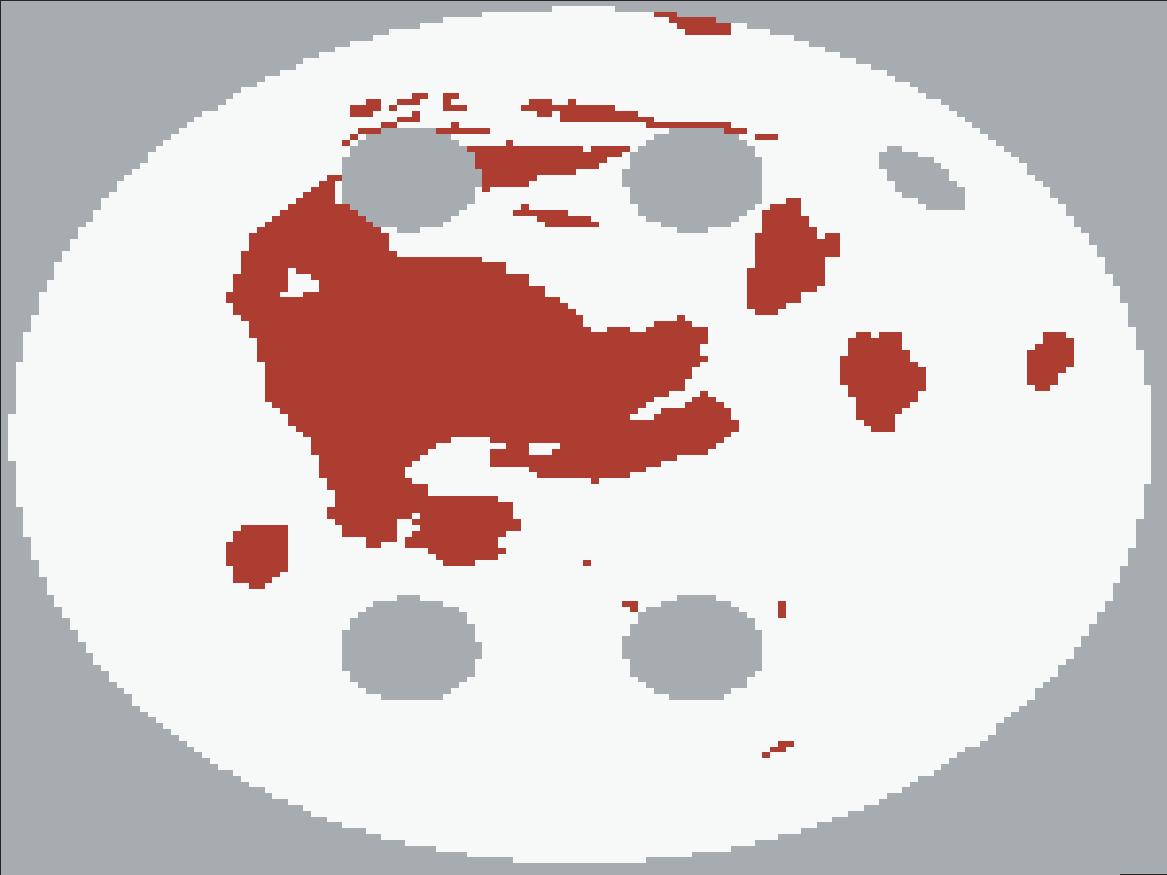

Supplement: Supplementary file 2 [file DataSheet1.ZIP › Dataset/real_FBLPH.jpg]

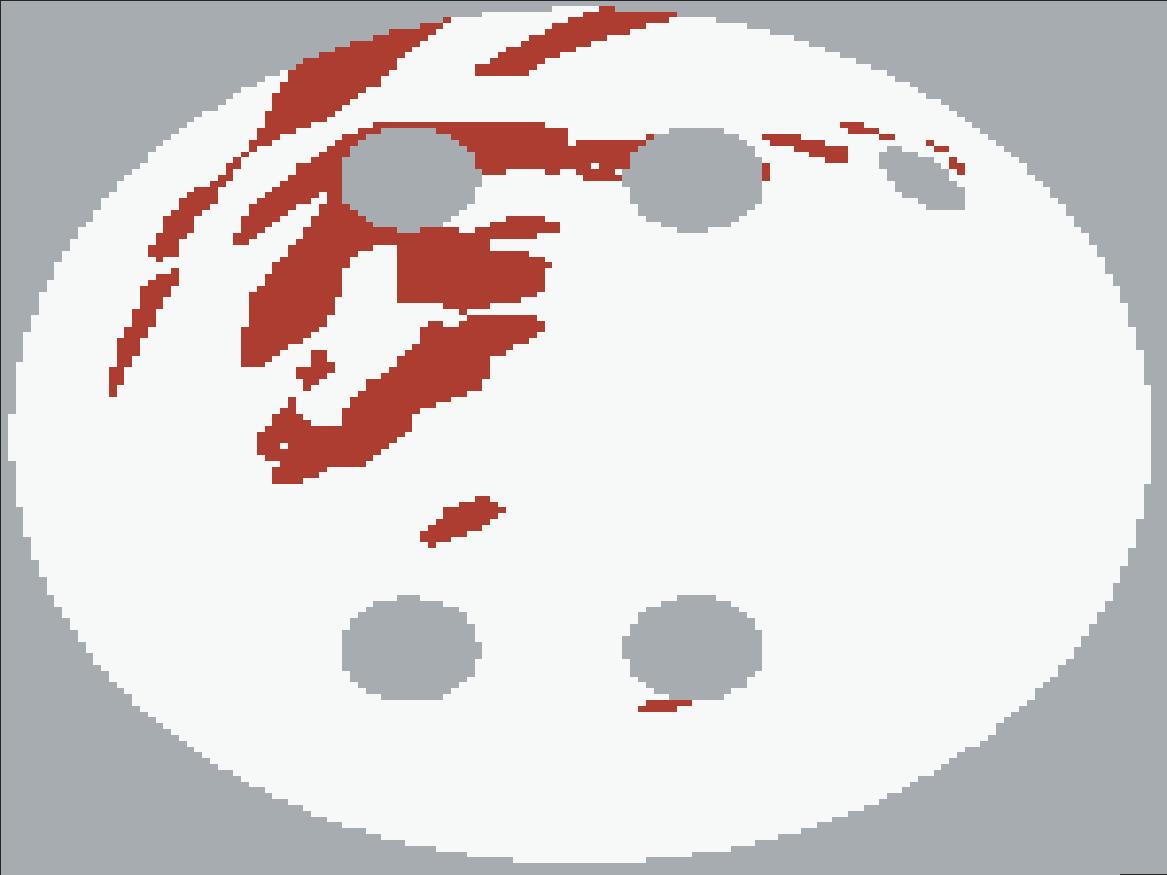

Supplement: Supplementary file 2 [file DataSheet1.ZIP › Dataset/real_FQ5FQ.jpg]

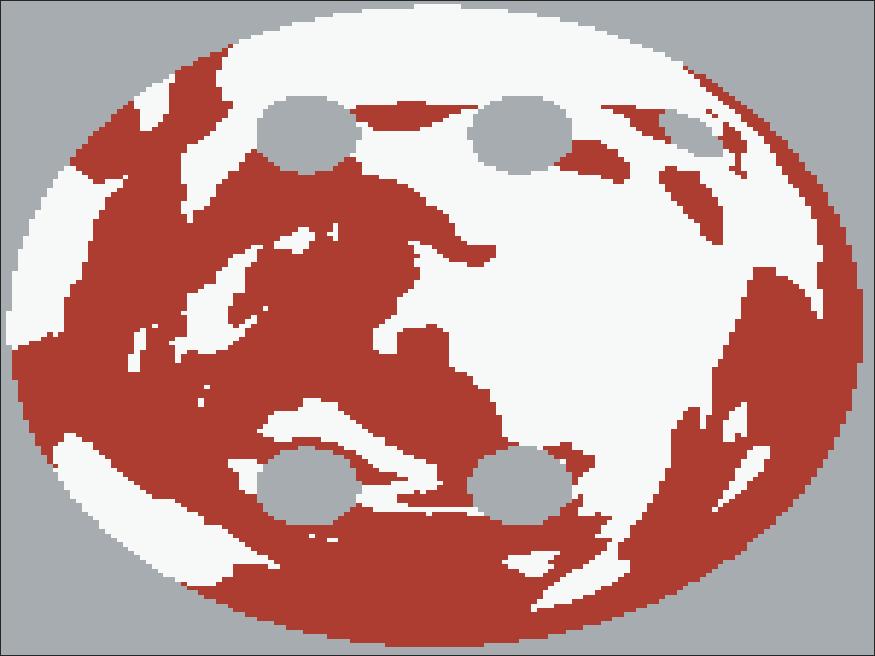

Supplement: Supplementary file 2 [file DataSheet1.ZIP › Dataset/real_FUE6V.jpg]

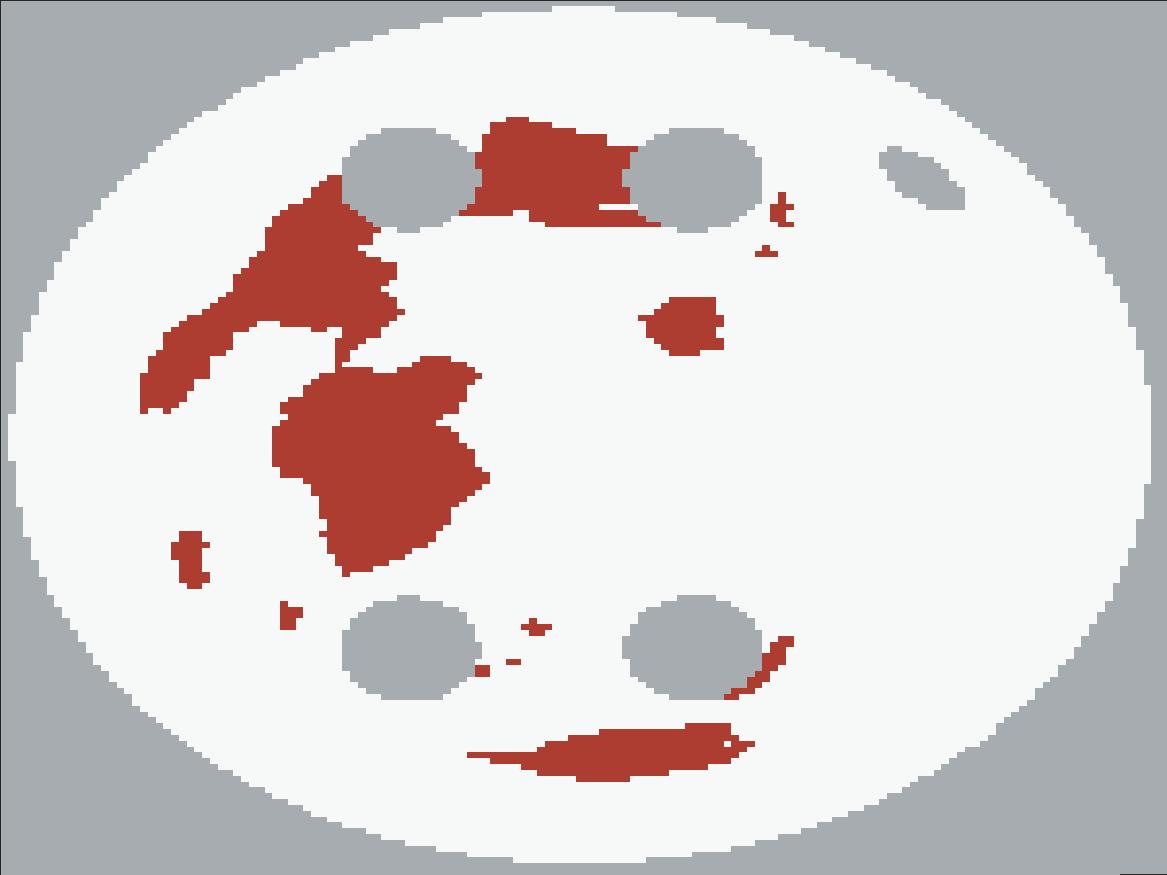

Supplement: Supplementary file 2 [file DataSheet1.ZIP › Dataset/real_GE3J7.jpg]

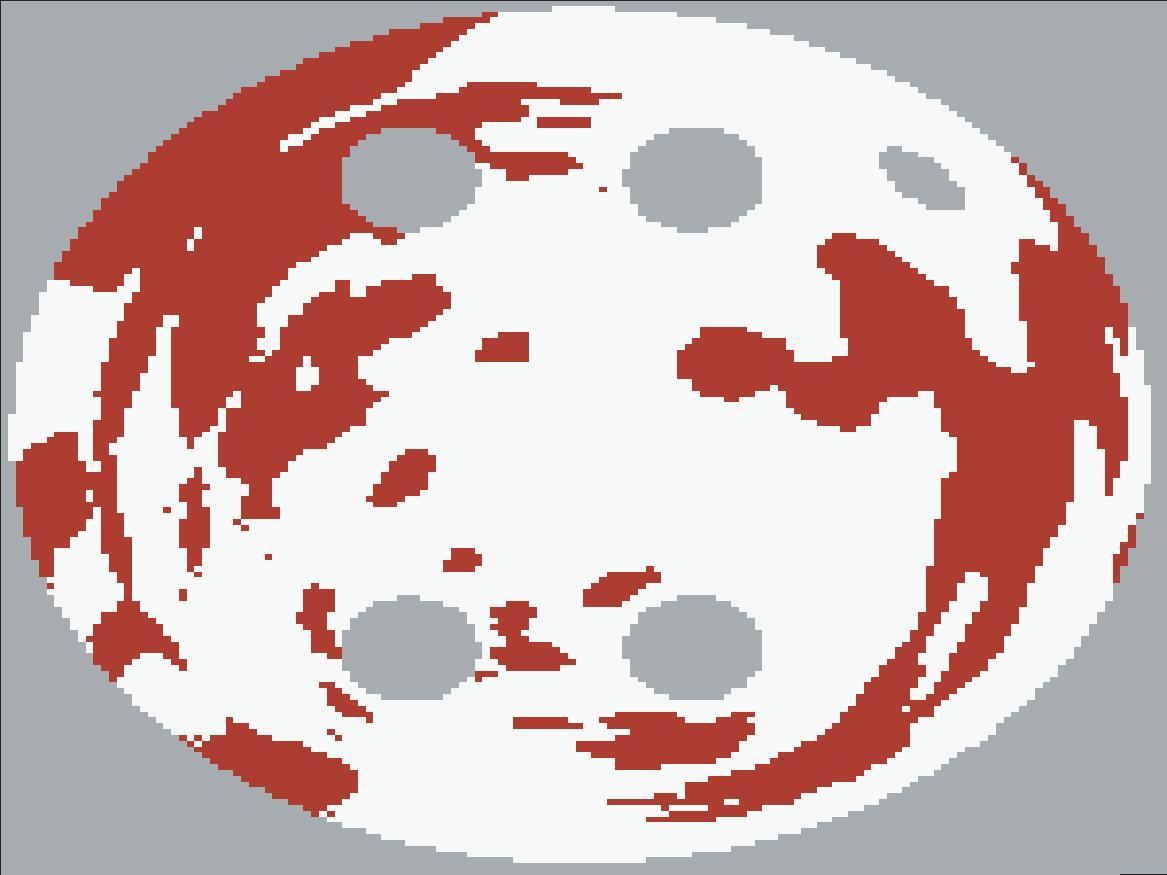

Supplement: Supplementary file 2 [file DataSheet1.ZIP › Dataset/real_GFDDR.jpg]

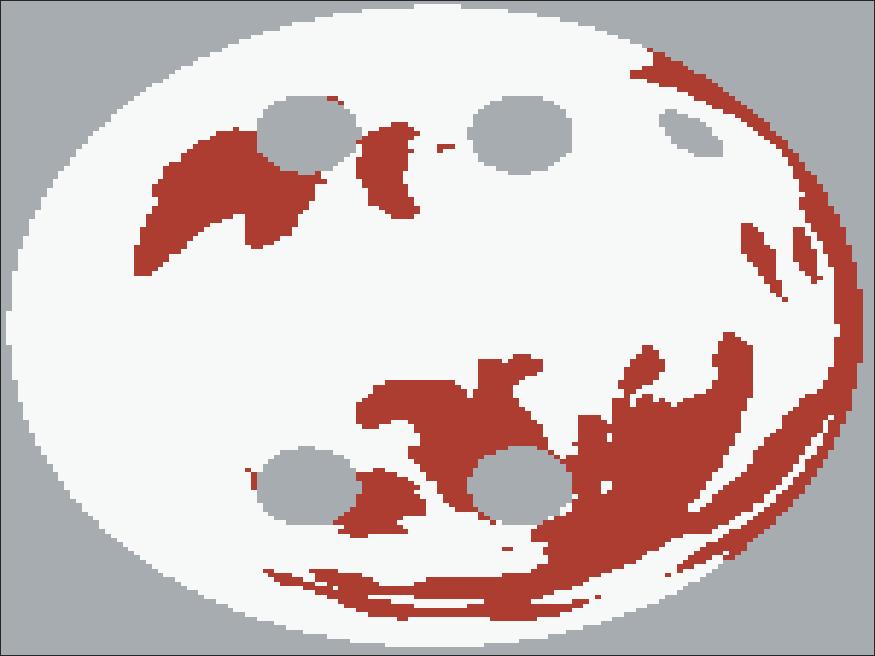

Supplement: Supplementary file 2 [file DataSheet1.ZIP › Dataset/real_GGRND.jpg]

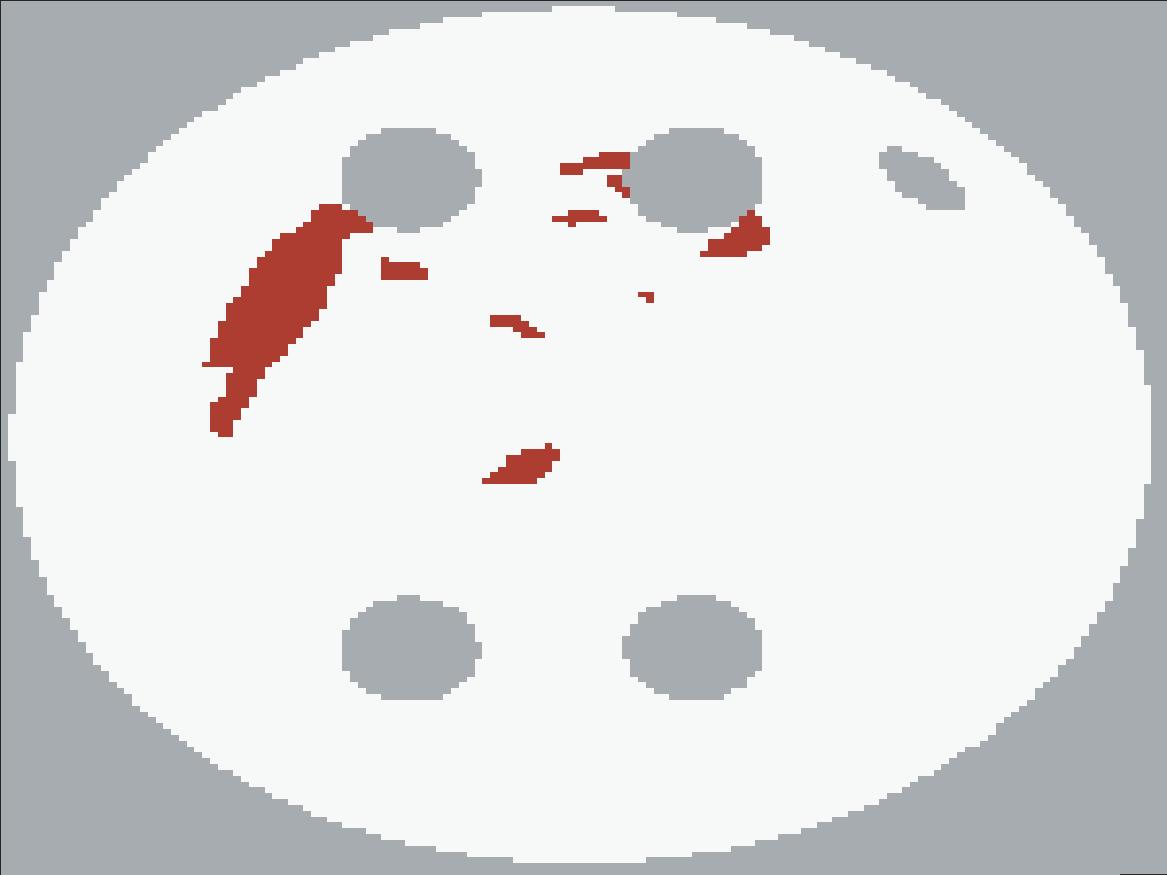

Supplement: Supplementary file 2 [file DataSheet1.ZIP › Dataset/real_GLKLE.jpg]

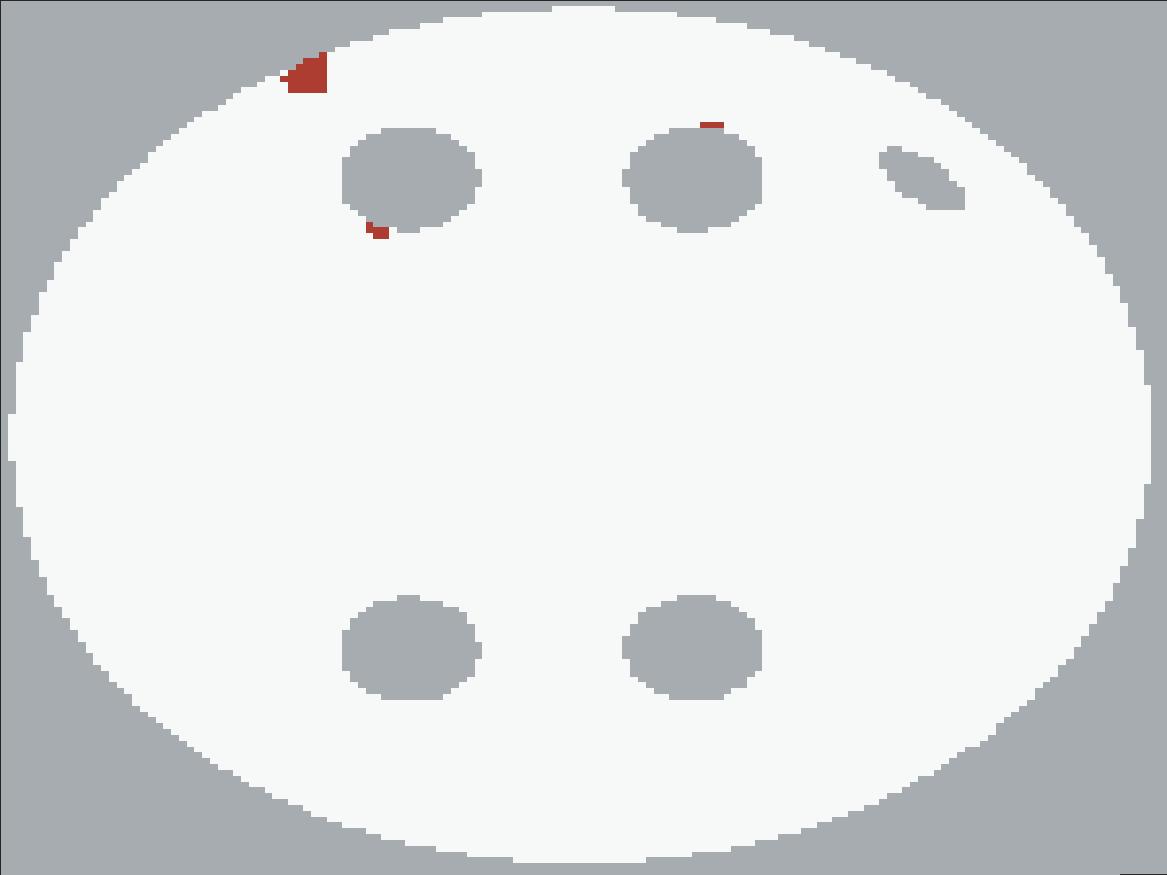

Supplement: Supplementary file 2 [file DataSheet1.ZIP › Dataset/real_GXY25.jpg]

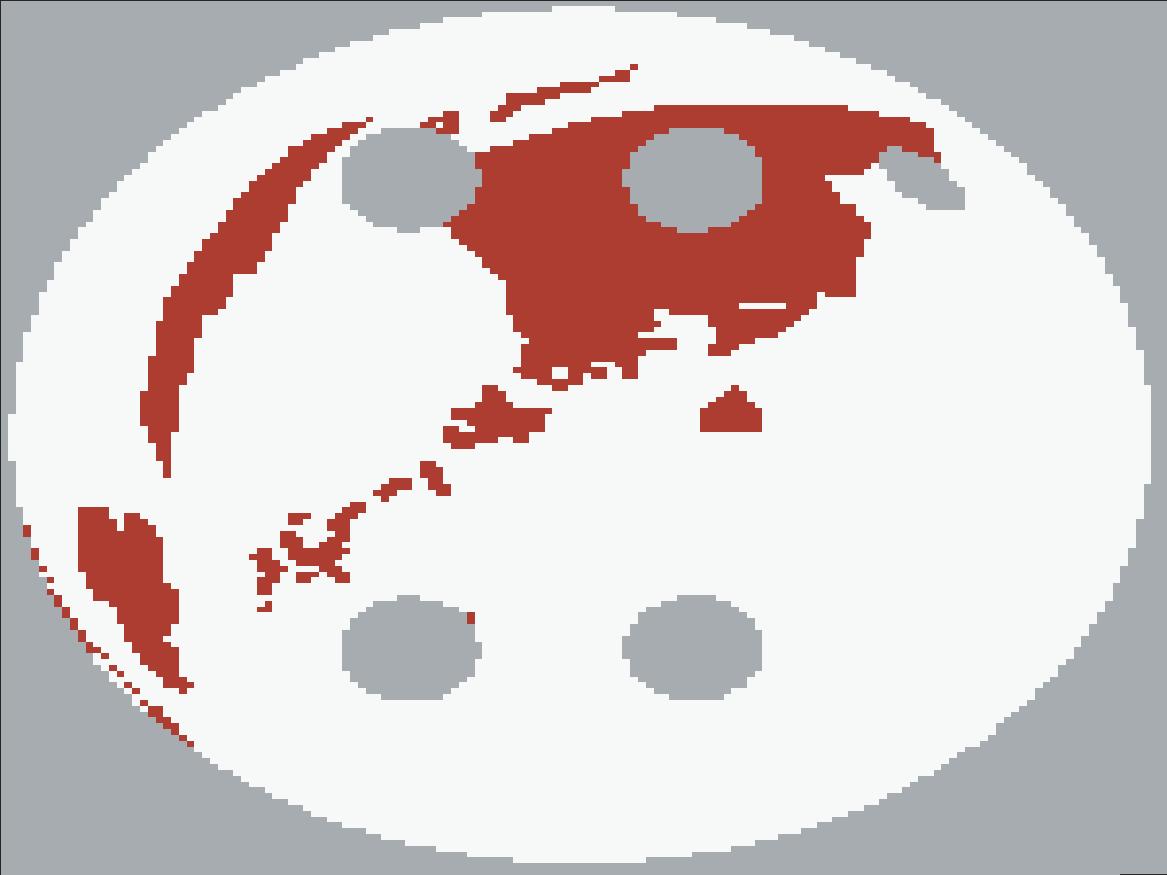

Supplement: Supplementary file 2 [file DataSheet1.ZIP › Dataset/real_HHKXO.jpg]

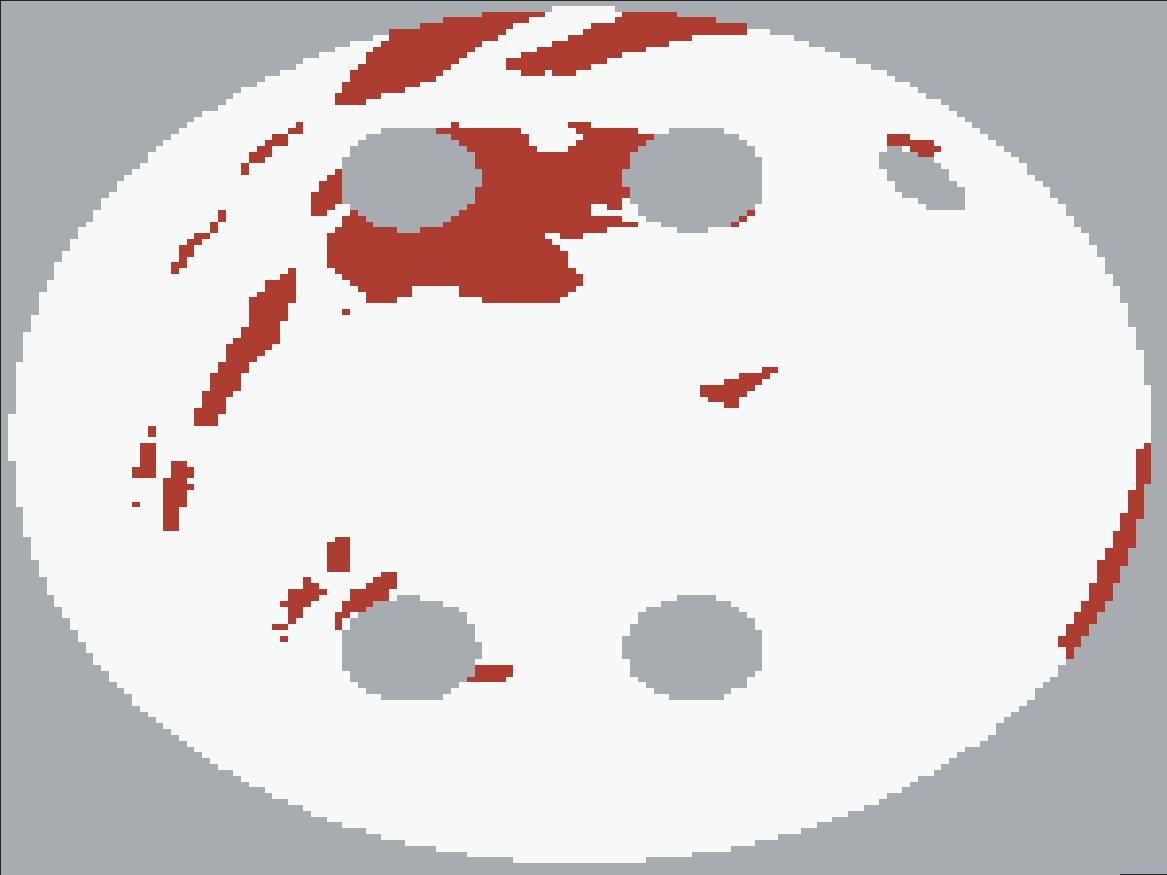

Supplement: Supplementary file 2 [file DataSheet1.ZIP › Dataset/real_HSQ9P.jpg]

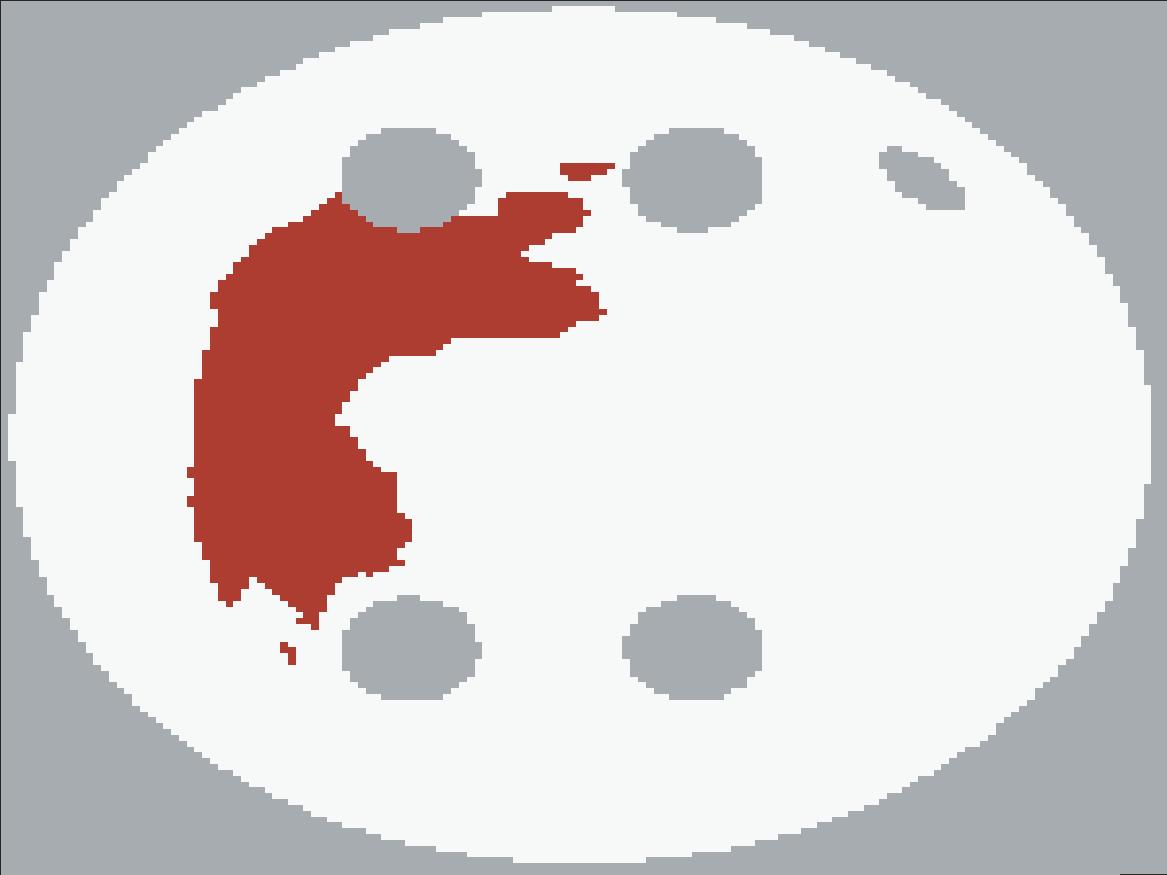

Supplement: Supplementary file 2 [file DataSheet1.ZIP › Dataset/real_HTLOU.jpg]

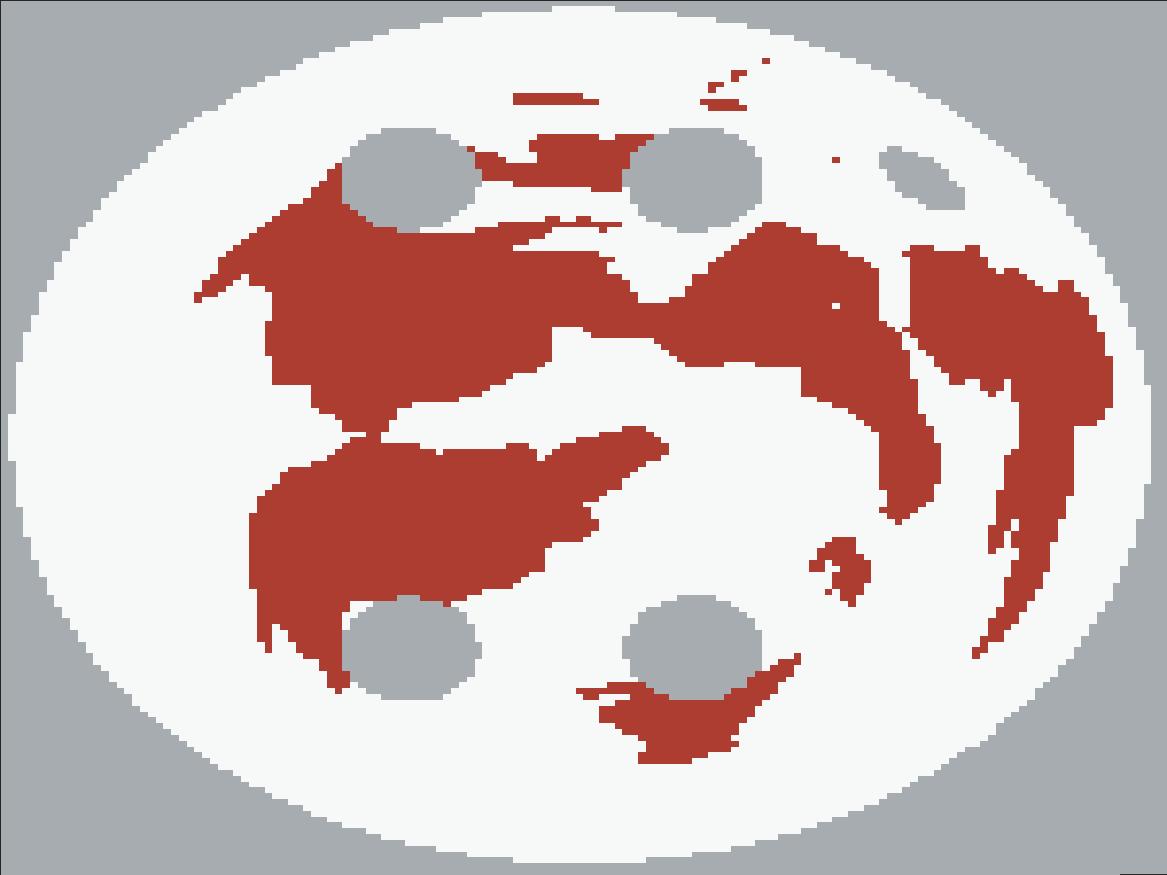

Supplement: Supplementary file 2 [file DataSheet1.ZIP › Dataset/real_HWOKF.jpg]

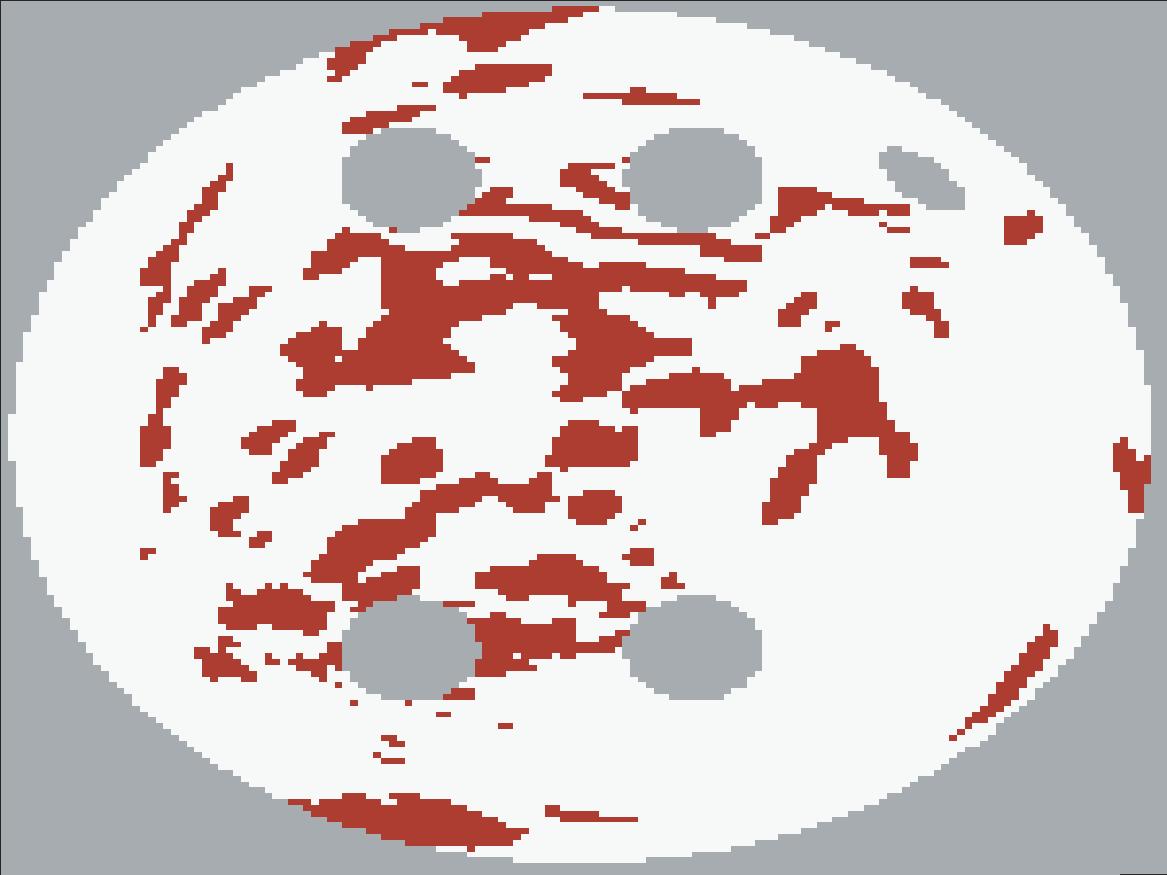

Supplement: Supplementary file 2 [file DataSheet1.ZIP › Dataset/real_HYH18.jpg]

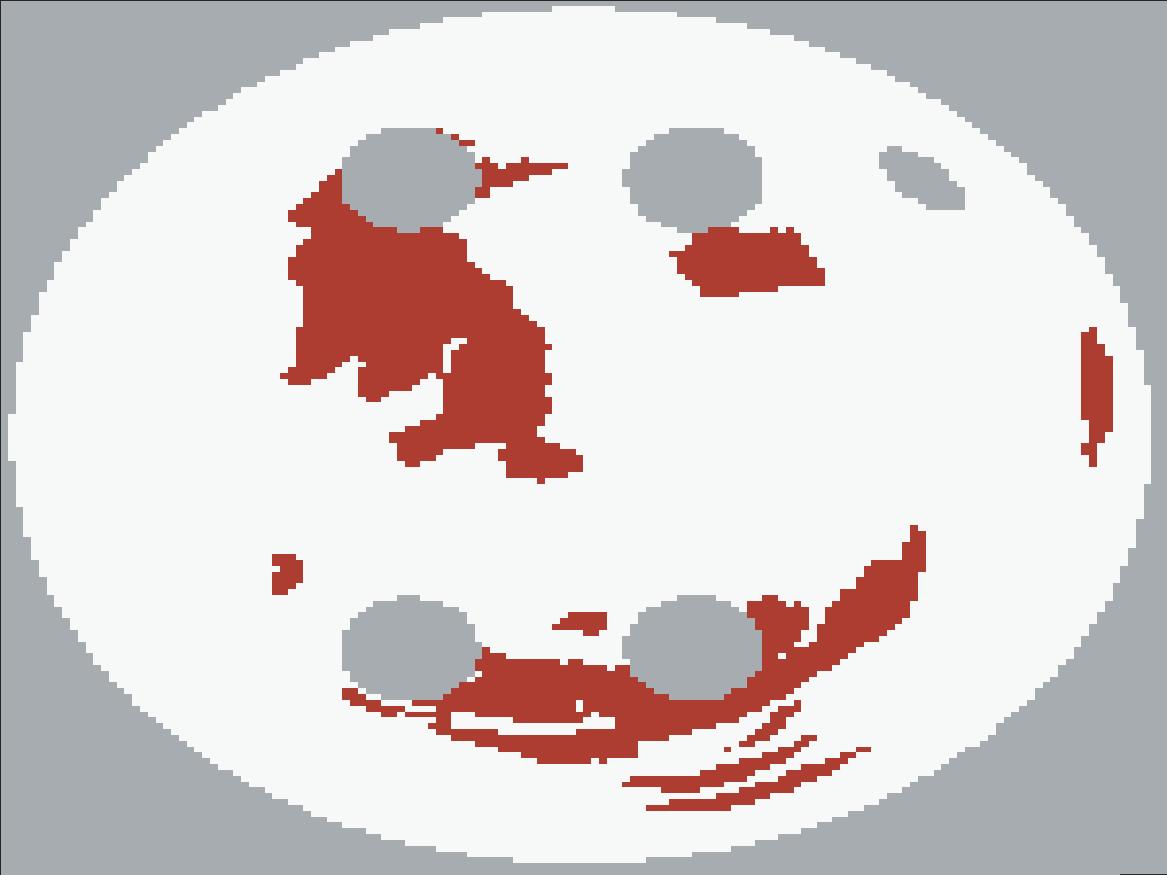

Supplement: Supplementary file 2 [file DataSheet1.ZIP › Dataset/real_I6GIA.jpg]

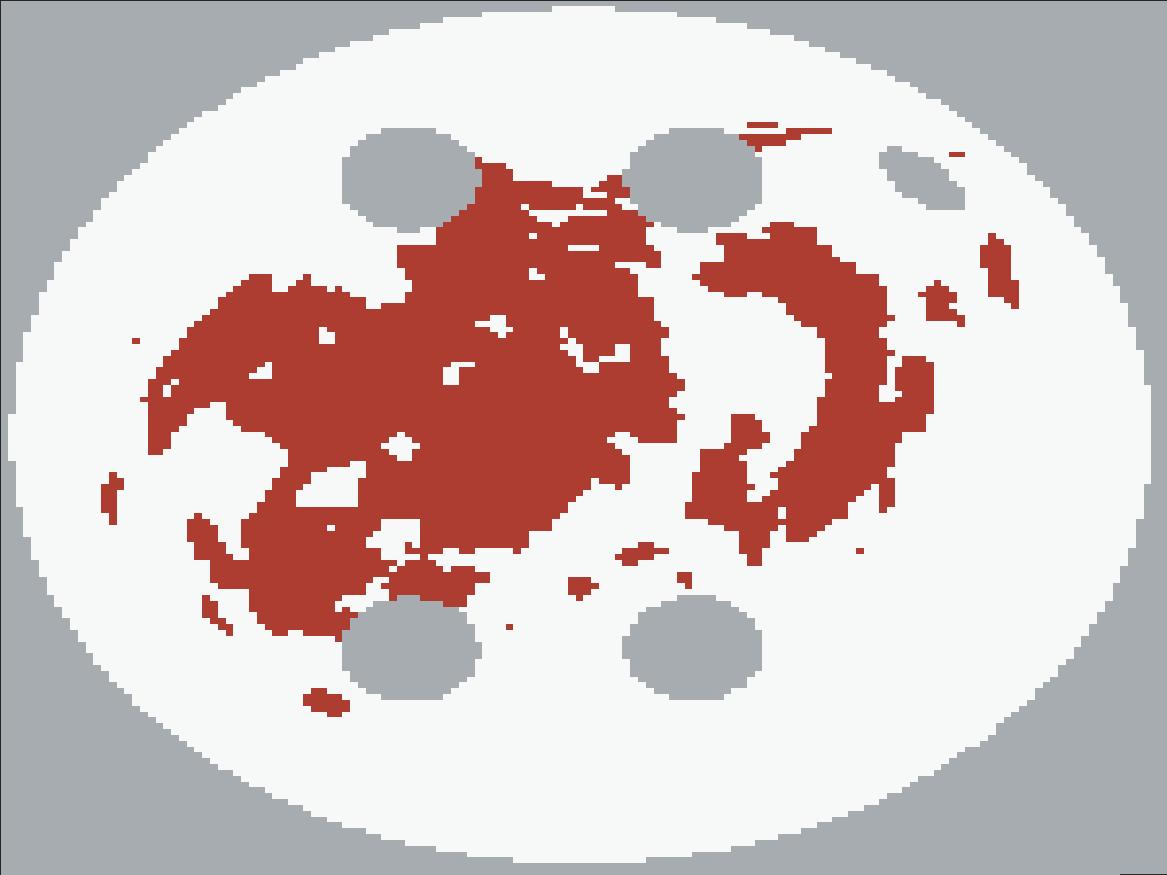

Supplement: Supplementary file 2 [file DataSheet1.ZIP › Dataset/real_I73AX.jpg]

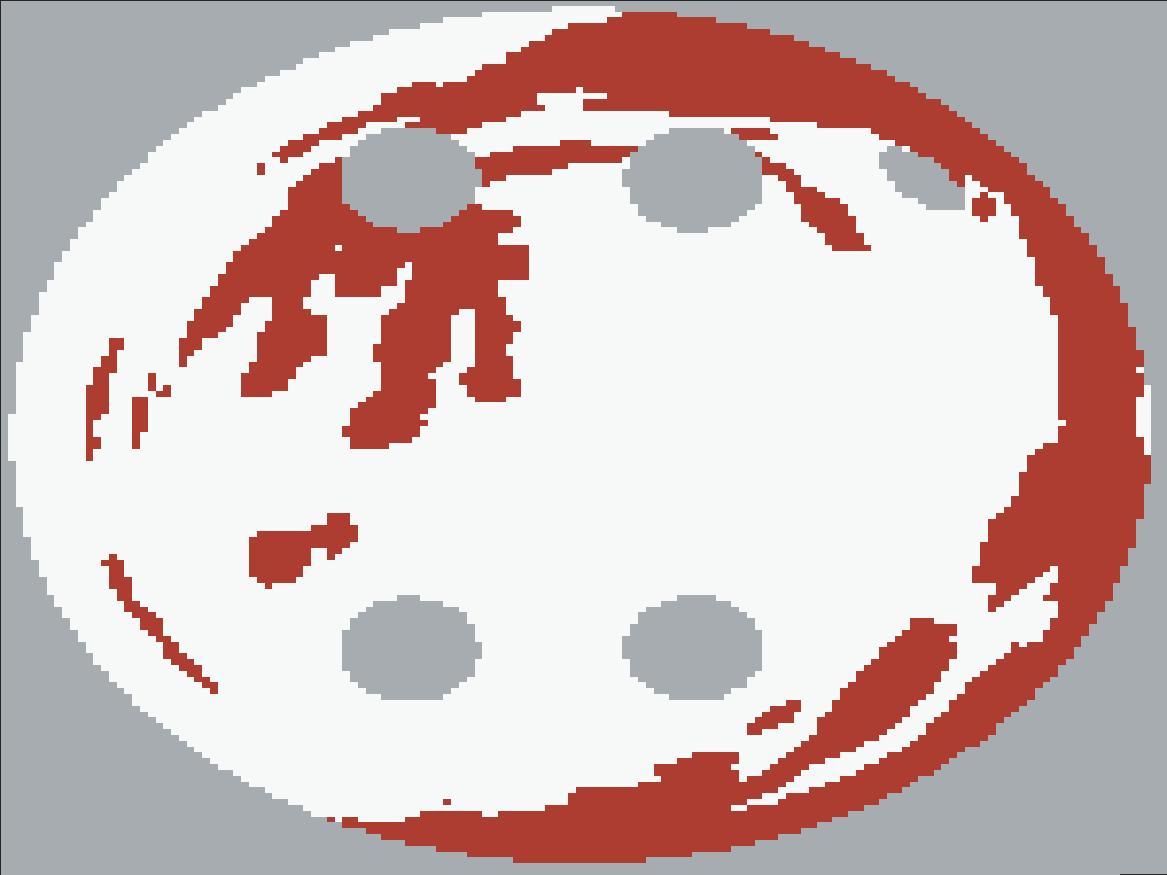

Supplement: Supplementary file 2 [file DataSheet1.ZIP › Dataset/real_IGAUD.jpg]

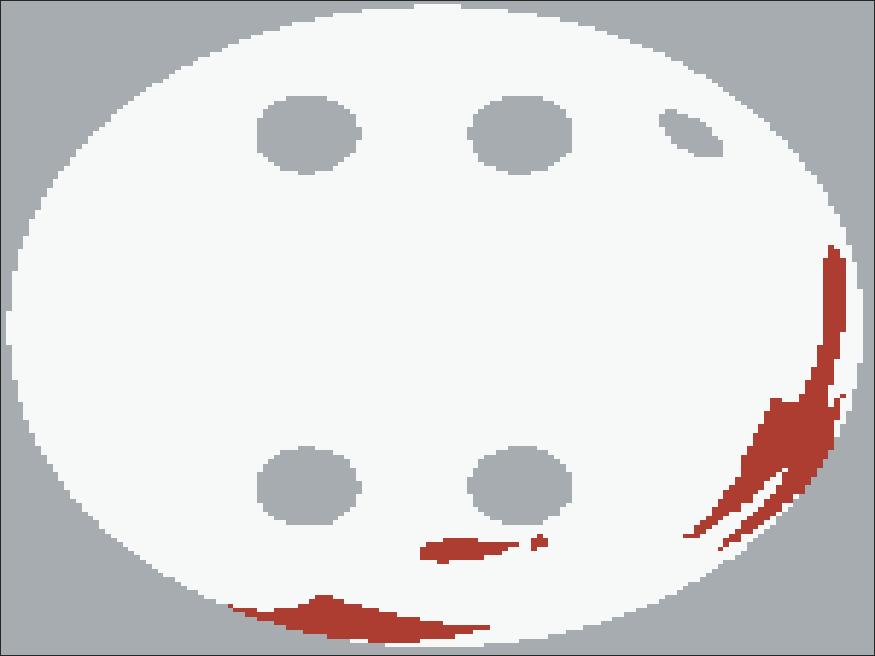

Supplement: Supplementary file 2 [file DataSheet1.ZIP › Dataset/real_IPAKF.jpg]

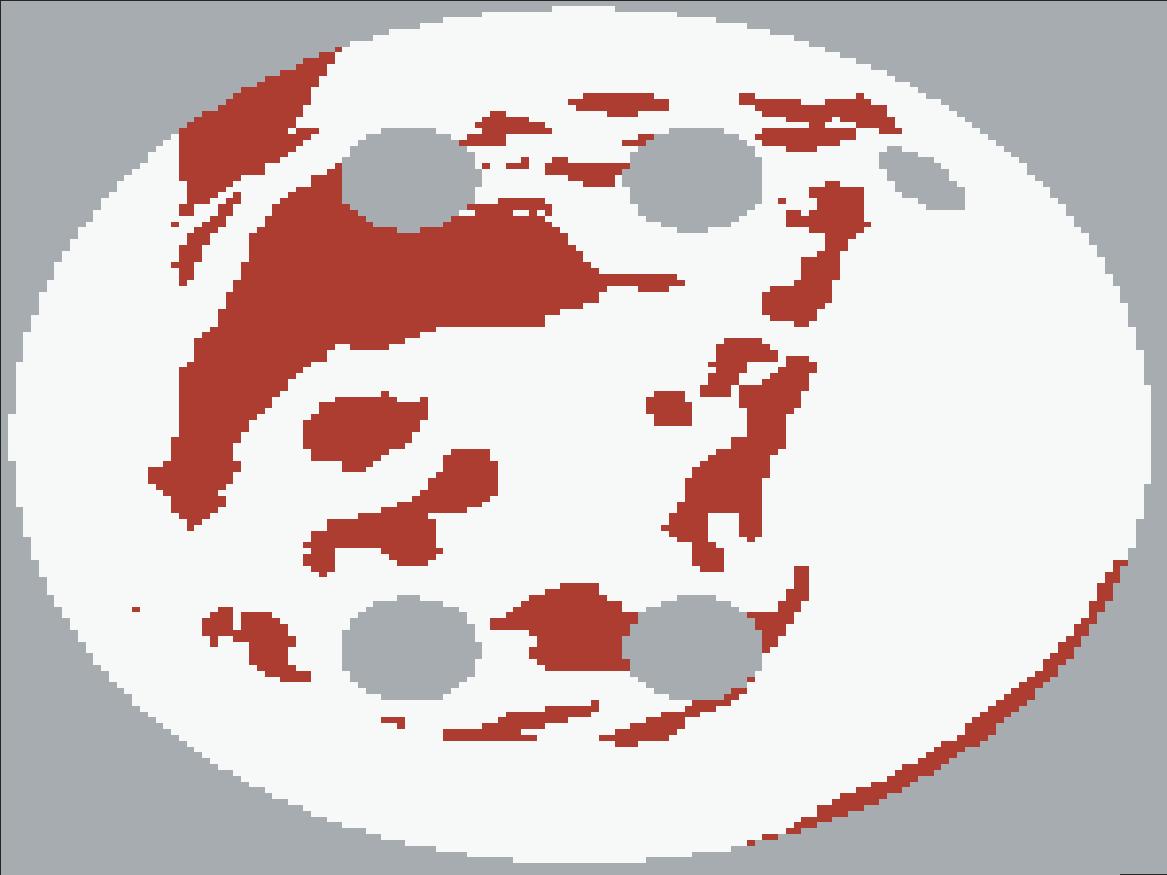

Supplement: Supplementary file 2 [file DataSheet1.ZIP › Dataset/real_IZXQ1.jpg]

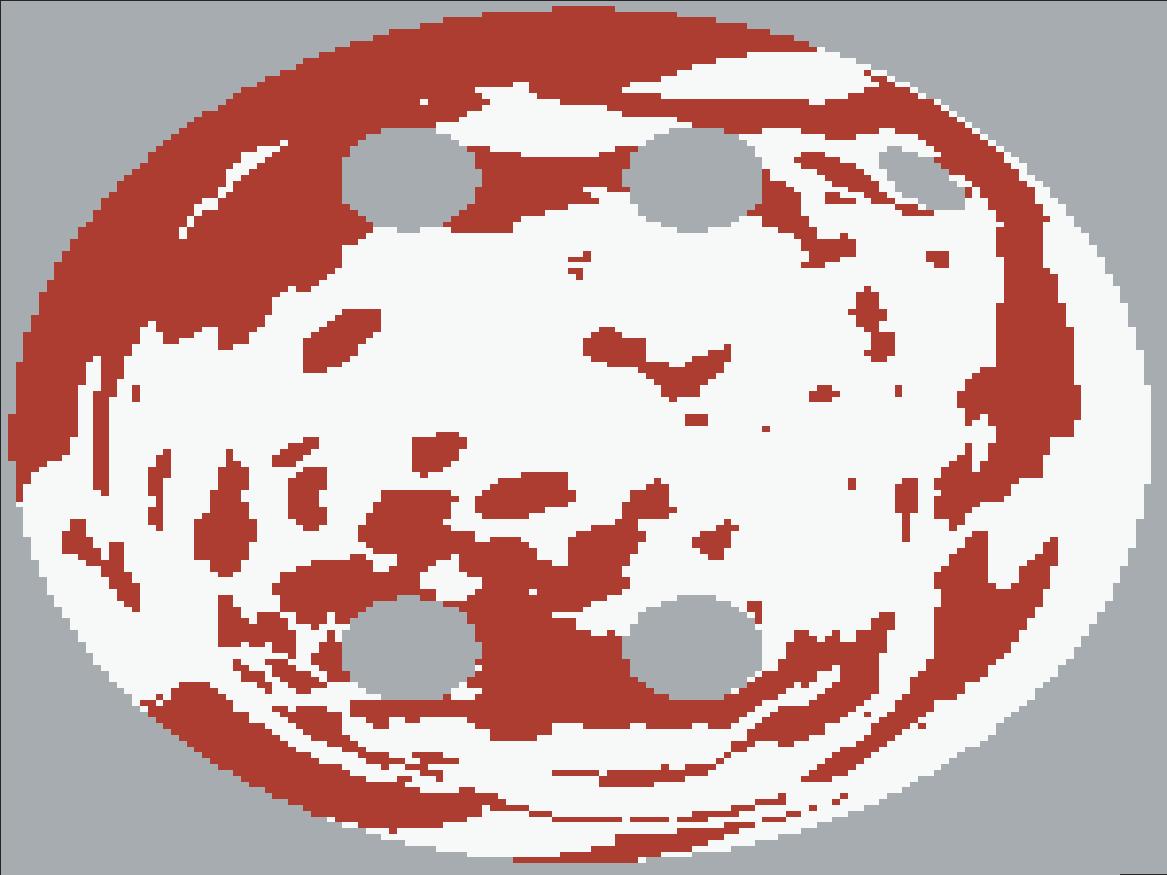

Supplement: Supplementary file 2 [file DataSheet1.ZIP › Dataset/real_J6NBL.jpg]

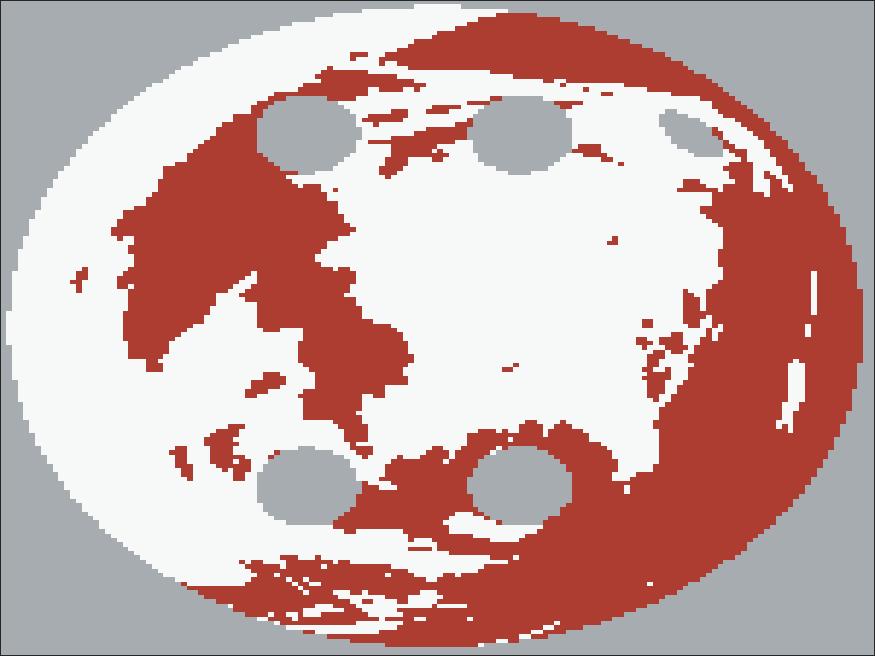

Supplement: Supplementary file 2 [file DataSheet1.ZIP › Dataset/real_K008Q.jpg]

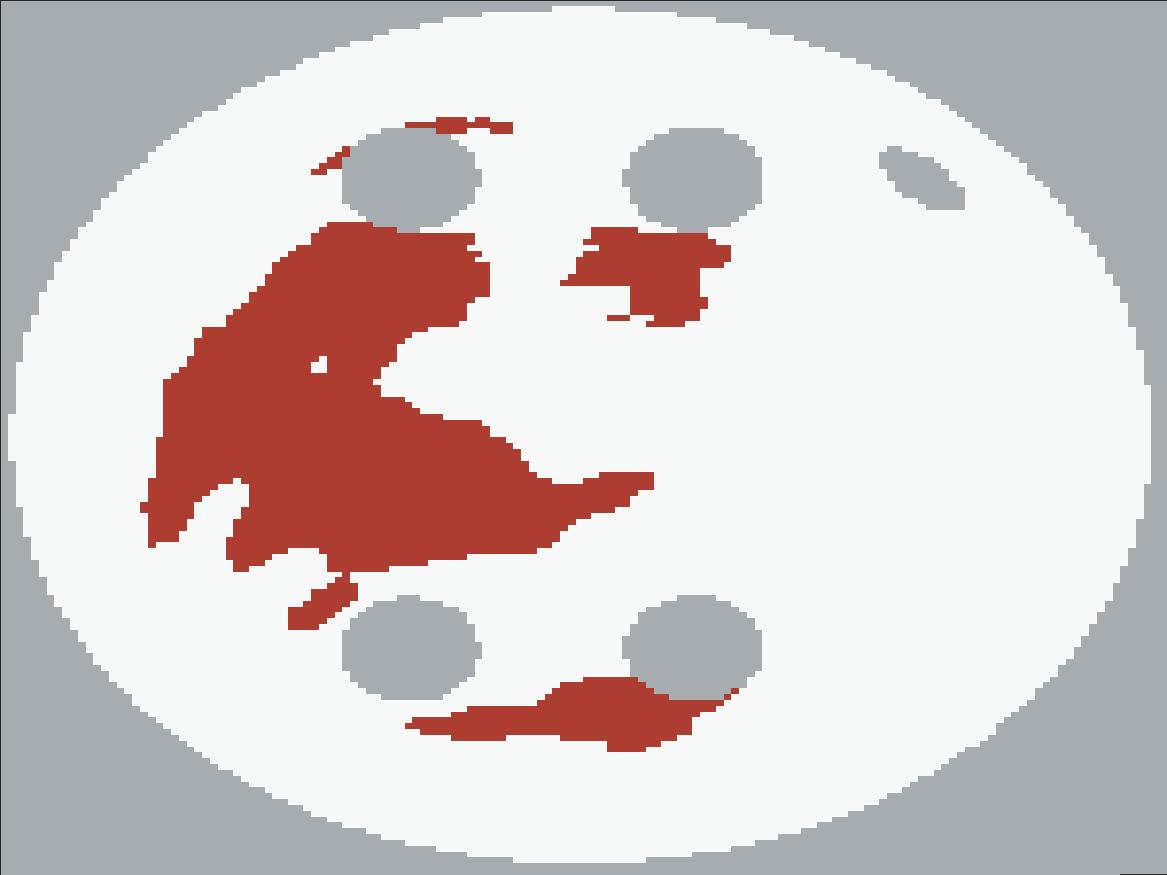

Supplement: Supplementary file 2 [file DataSheet1.ZIP › Dataset/real_KA0CO.jpg]

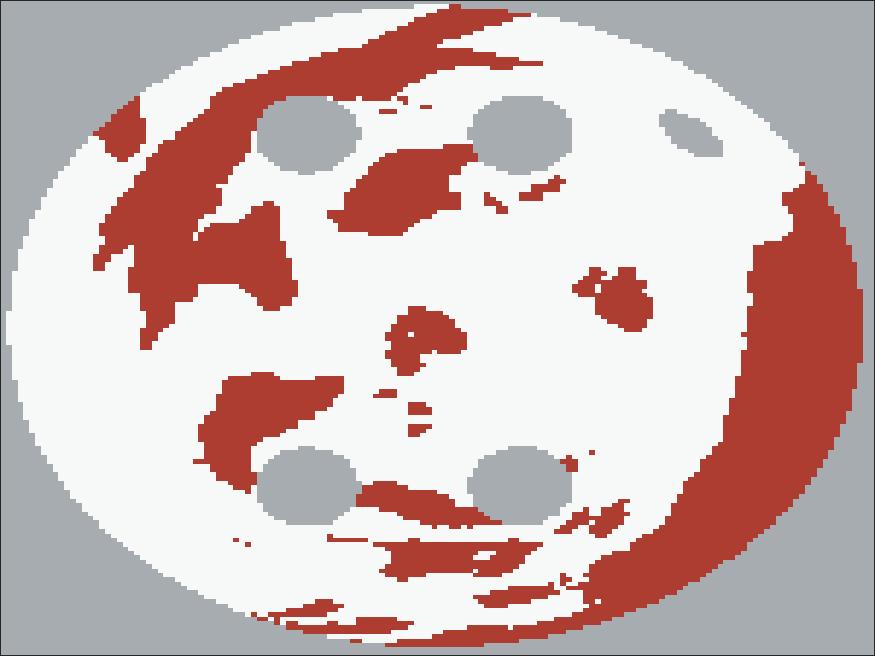

Supplement: Supplementary file 2 [file DataSheet1.ZIP › Dataset/real_KT5DI.jpg]

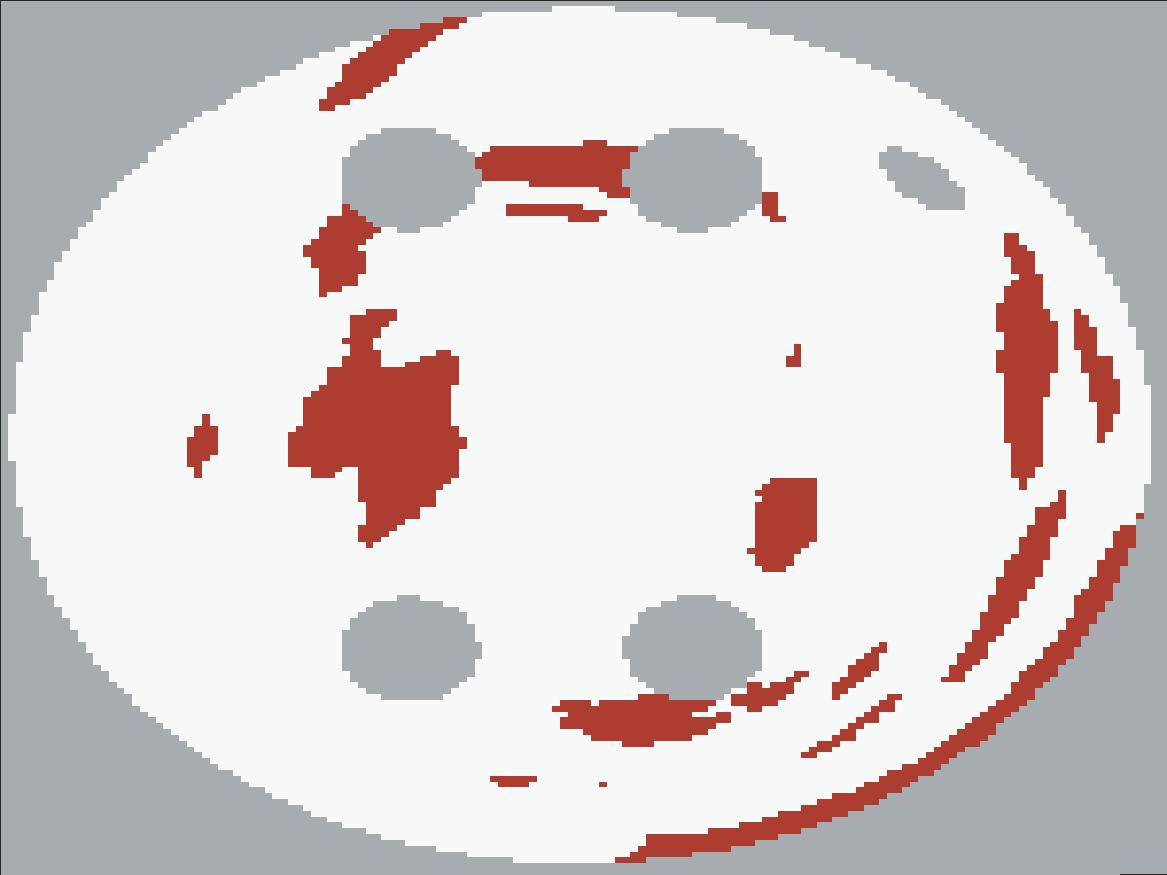

Supplement: Supplementary file 2 [file DataSheet1.ZIP › Dataset/real_L05SC.jpg]

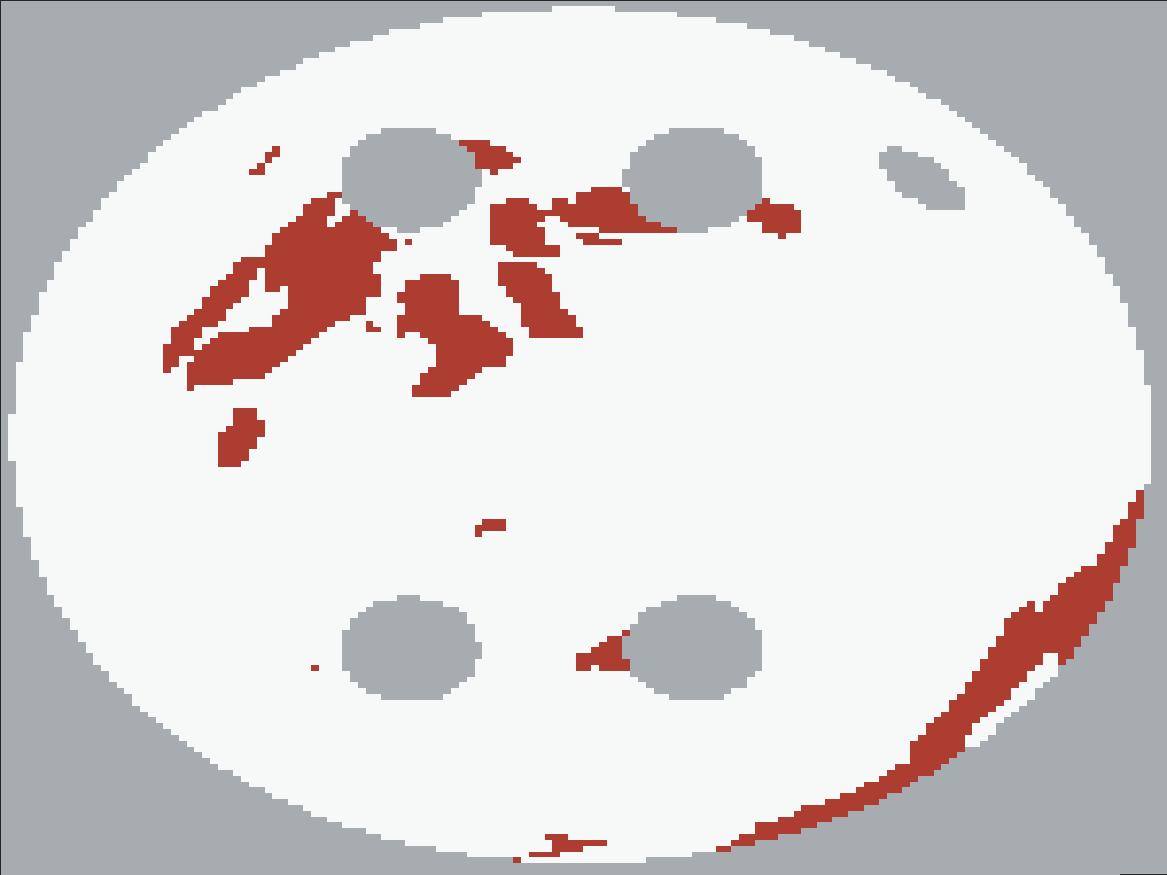

Supplement: Supplementary file 2 [file DataSheet1.ZIP › Dataset/real_LWVDE.jpg]

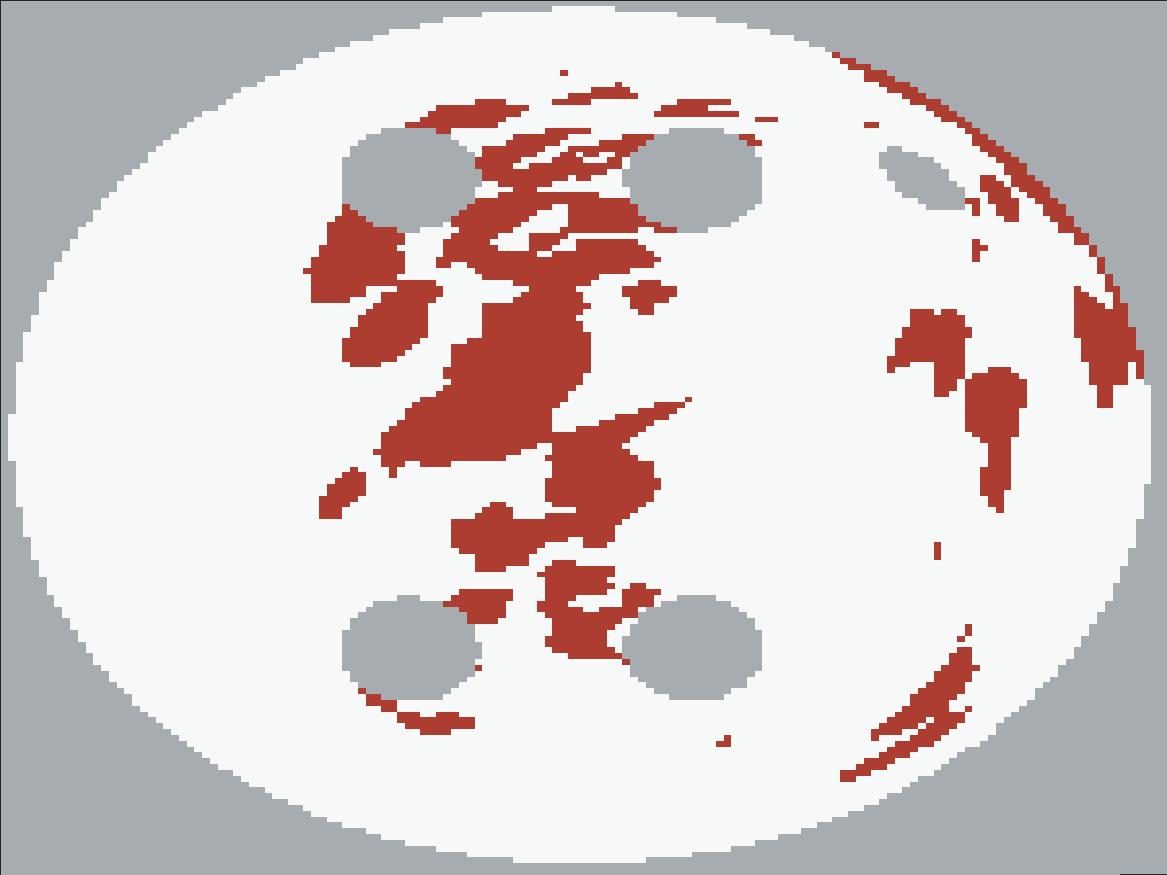

Supplement: Supplementary file 2 [file DataSheet1.ZIP › Dataset/real_MDF66.jpg]

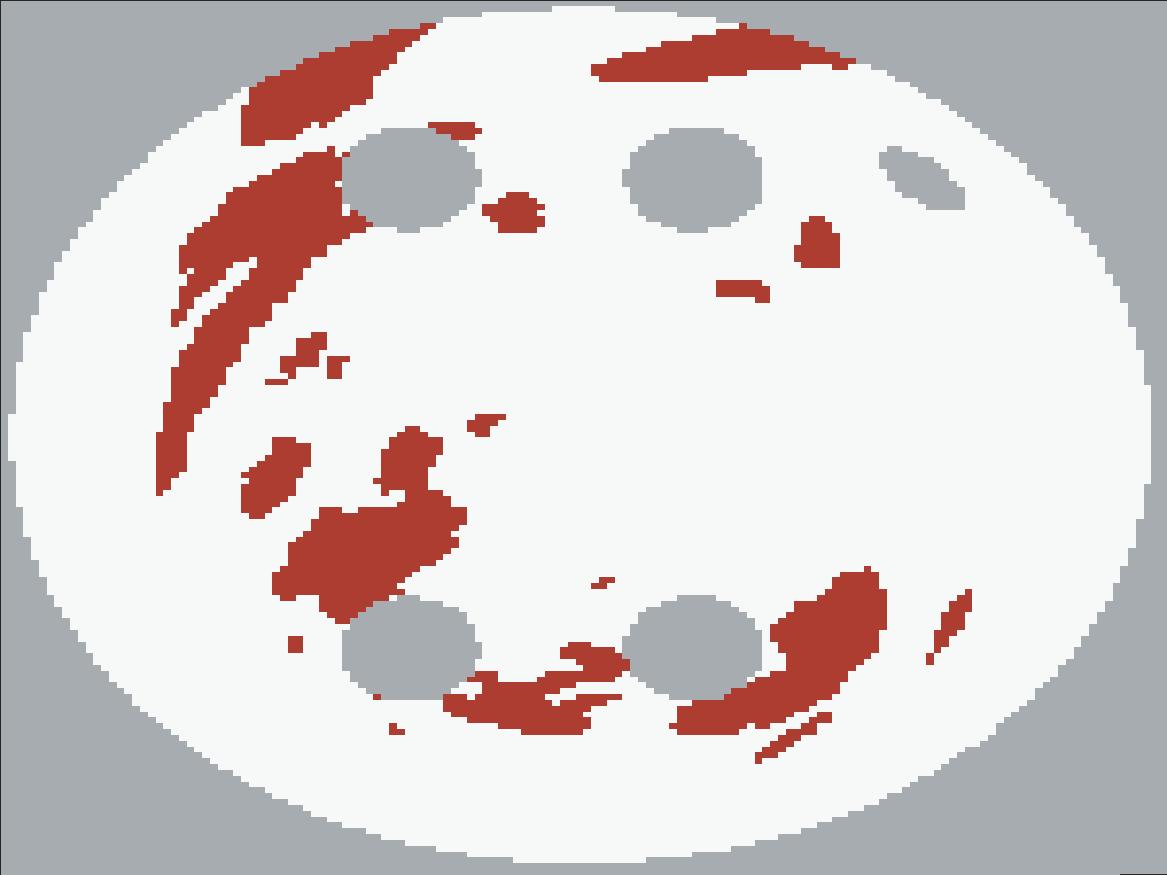

Supplement: Supplementary file 2 [file DataSheet1.ZIP › Dataset/real_MKS2S.jpg]

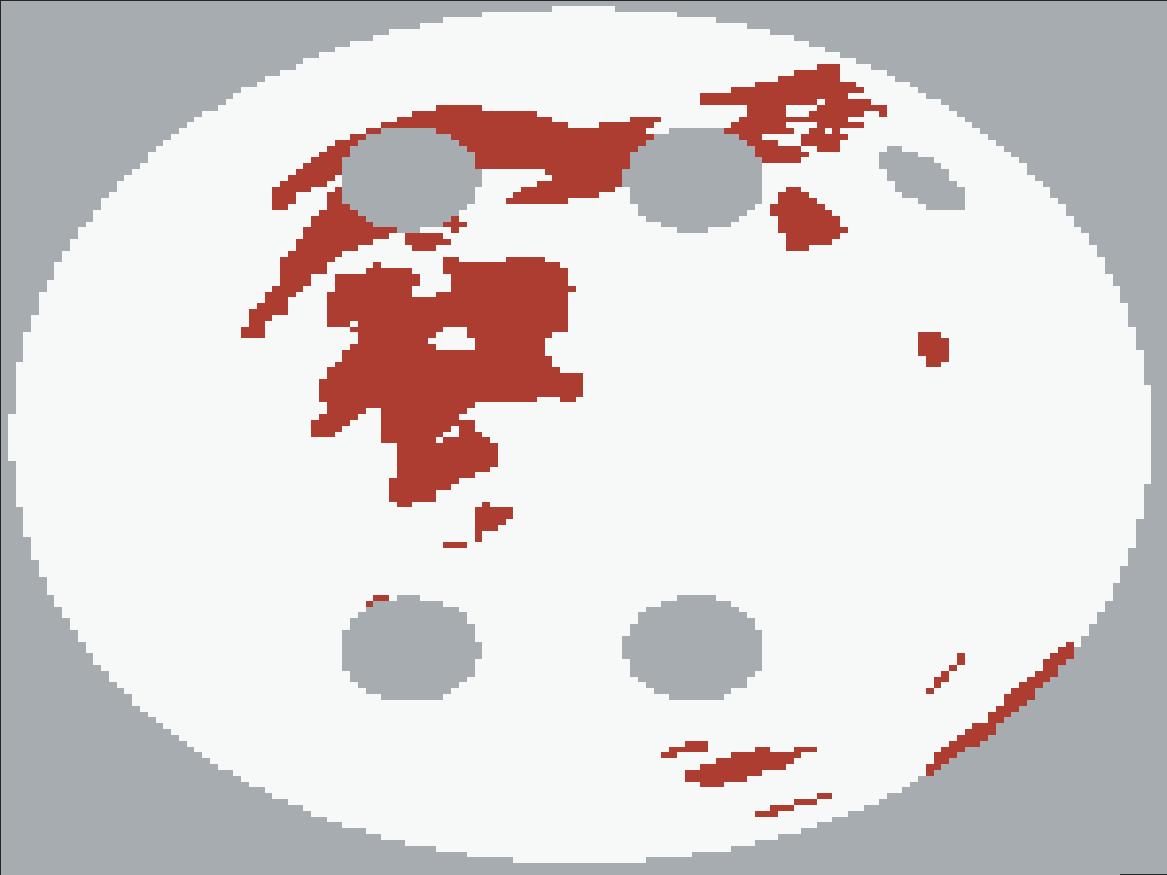

Supplement: Supplementary file 2 [file DataSheet1.ZIP › Dataset/real_MLC9B.jpg]

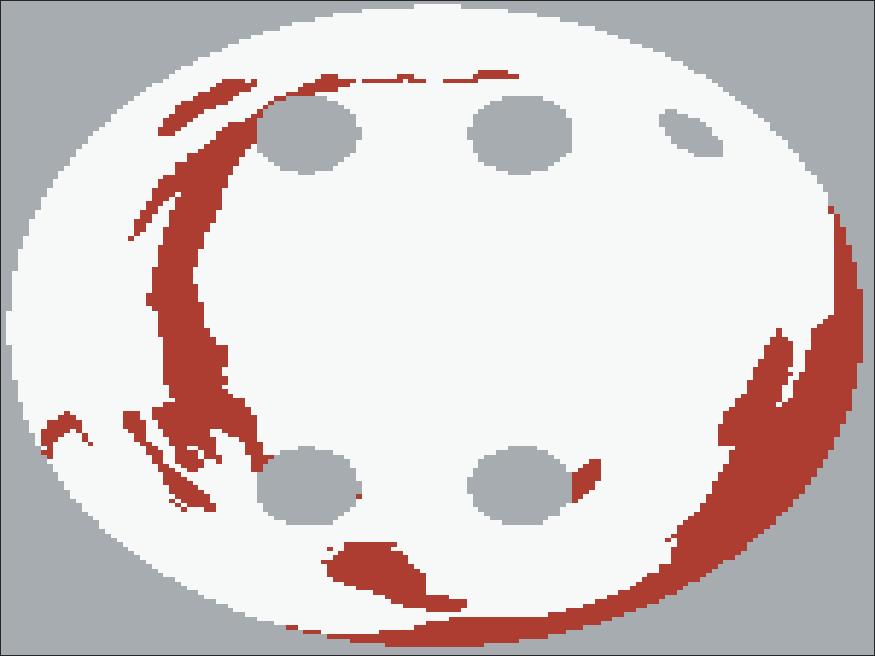

Supplement: Supplementary file 2 [file DataSheet1.ZIP › Dataset/real_MS8C9.jpg]

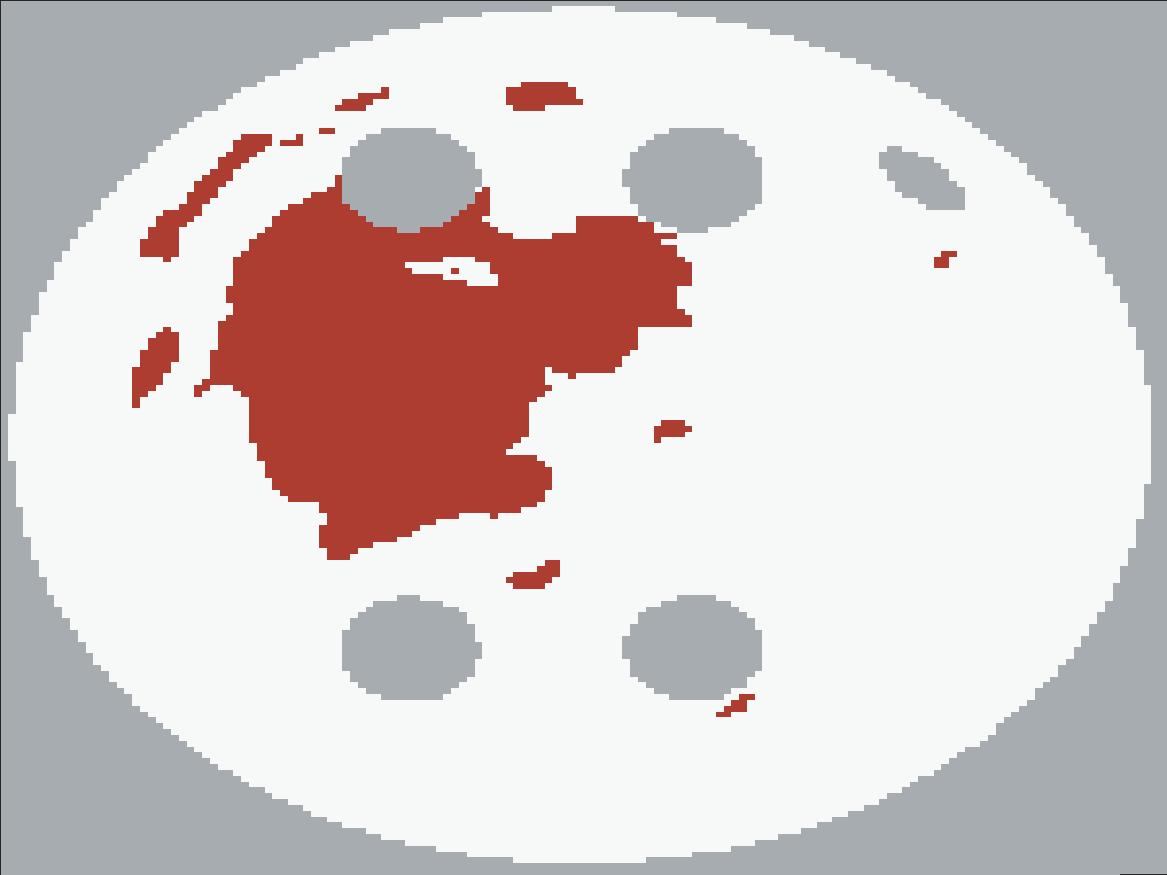

Supplement: Supplementary file 2 [file DataSheet1.ZIP › Dataset/real_N8QR0.jpg]

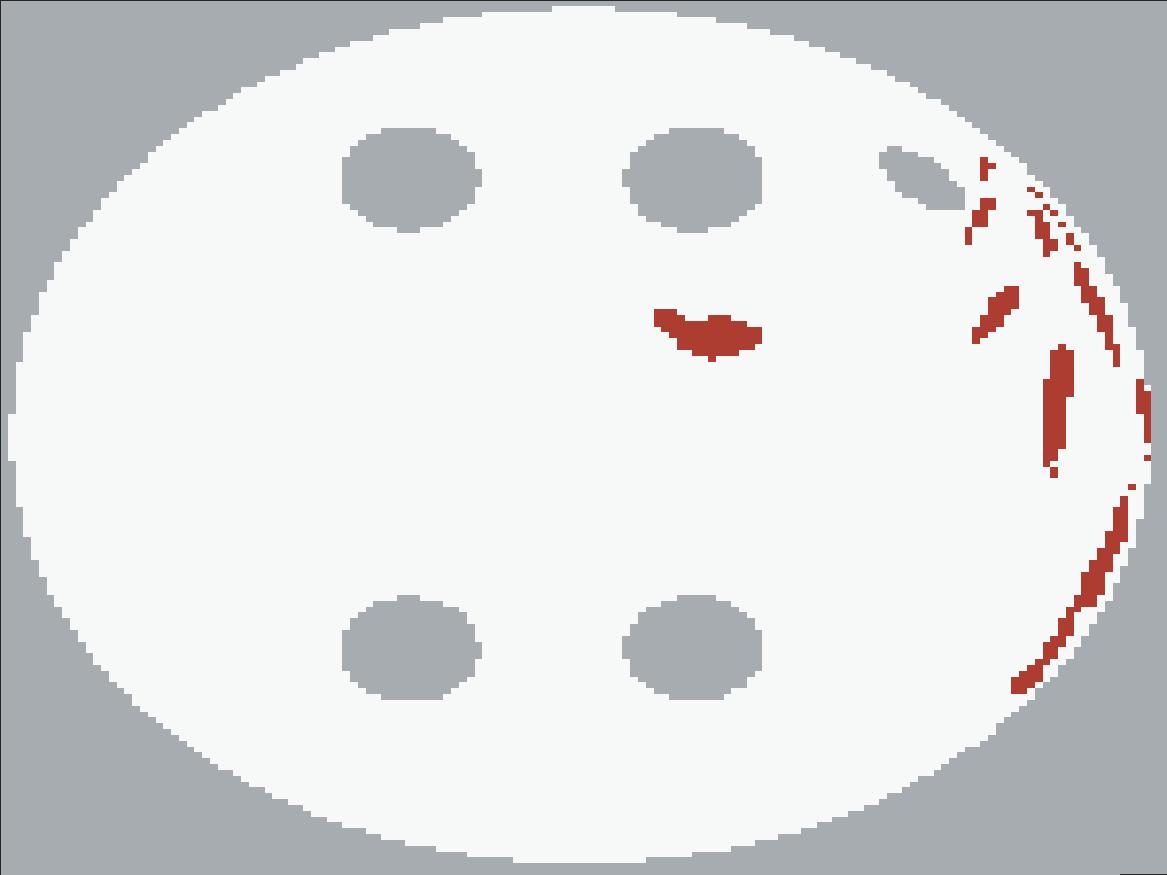

Supplement: Supplementary file 2 [file DataSheet1.ZIP › Dataset/real_NCYZI.jpg]

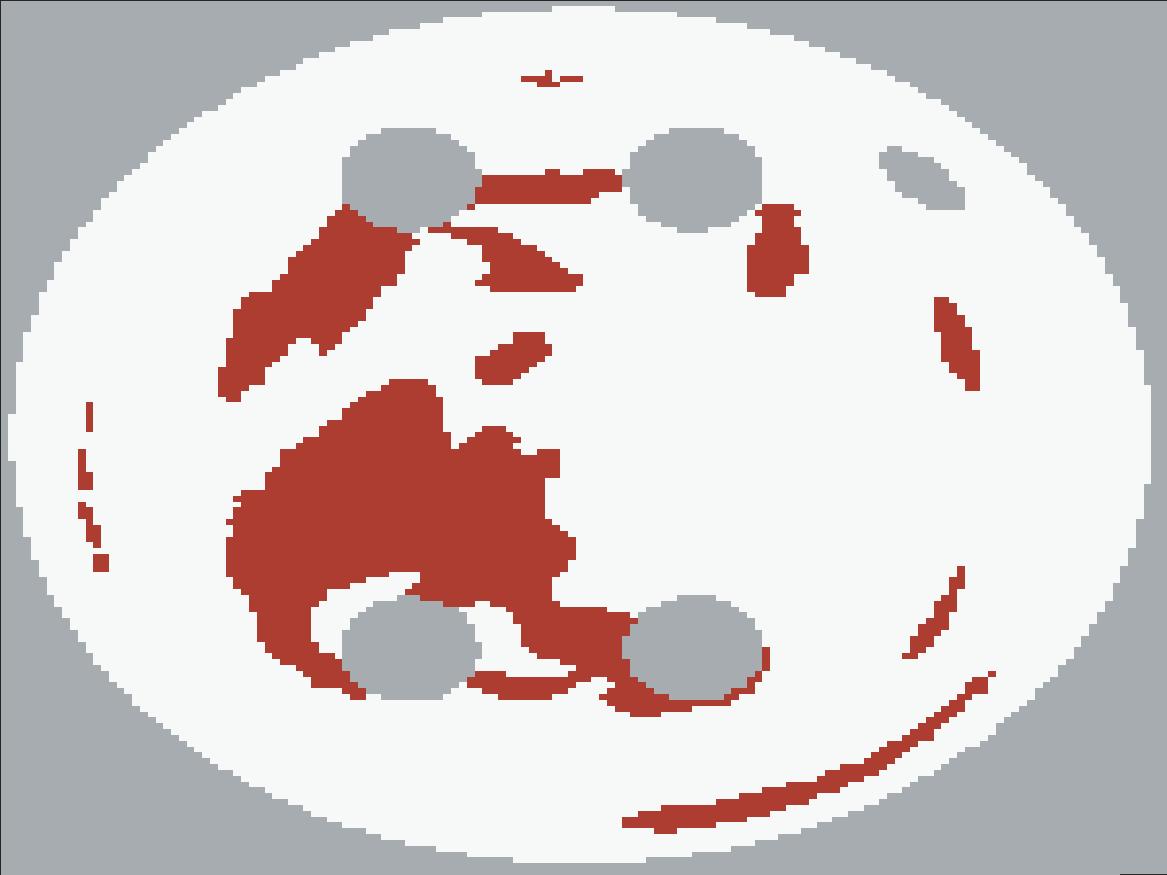

Supplement: Supplementary file 2 [file DataSheet1.ZIP › Dataset/real_OMIFH.jpg]

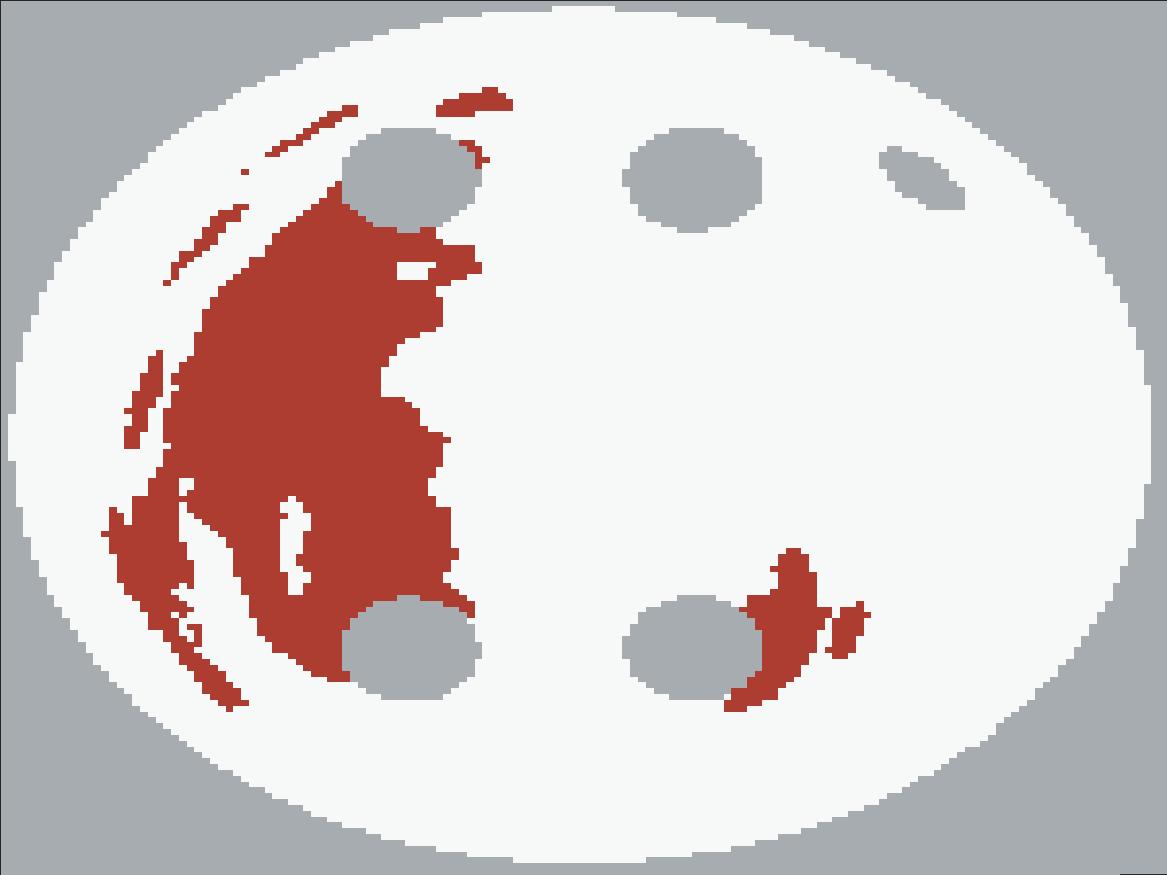

Supplement: Supplementary file 2 [file DataSheet1.ZIP › Dataset/real_P2WX9.jpg]

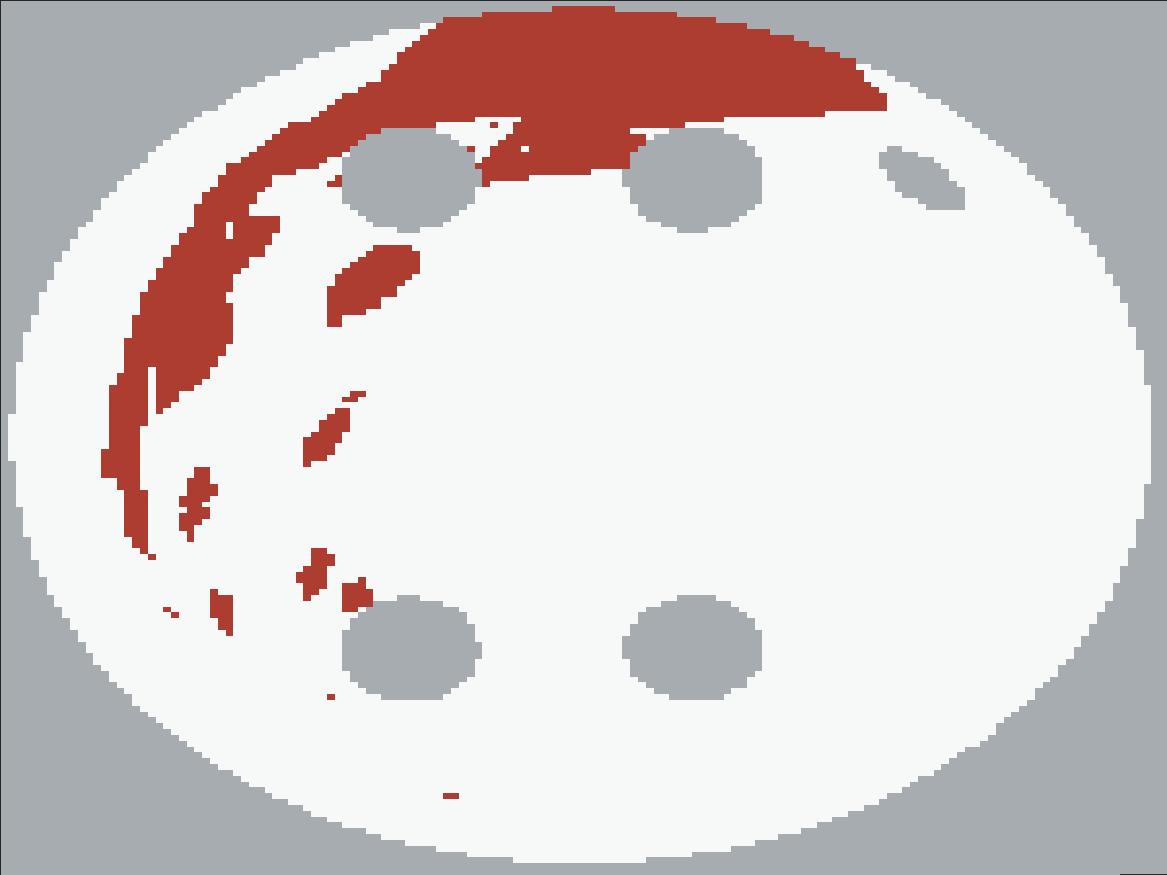

Supplement: Supplementary file 2 [file DataSheet1.ZIP › Dataset/real_PBCC4.jpg]

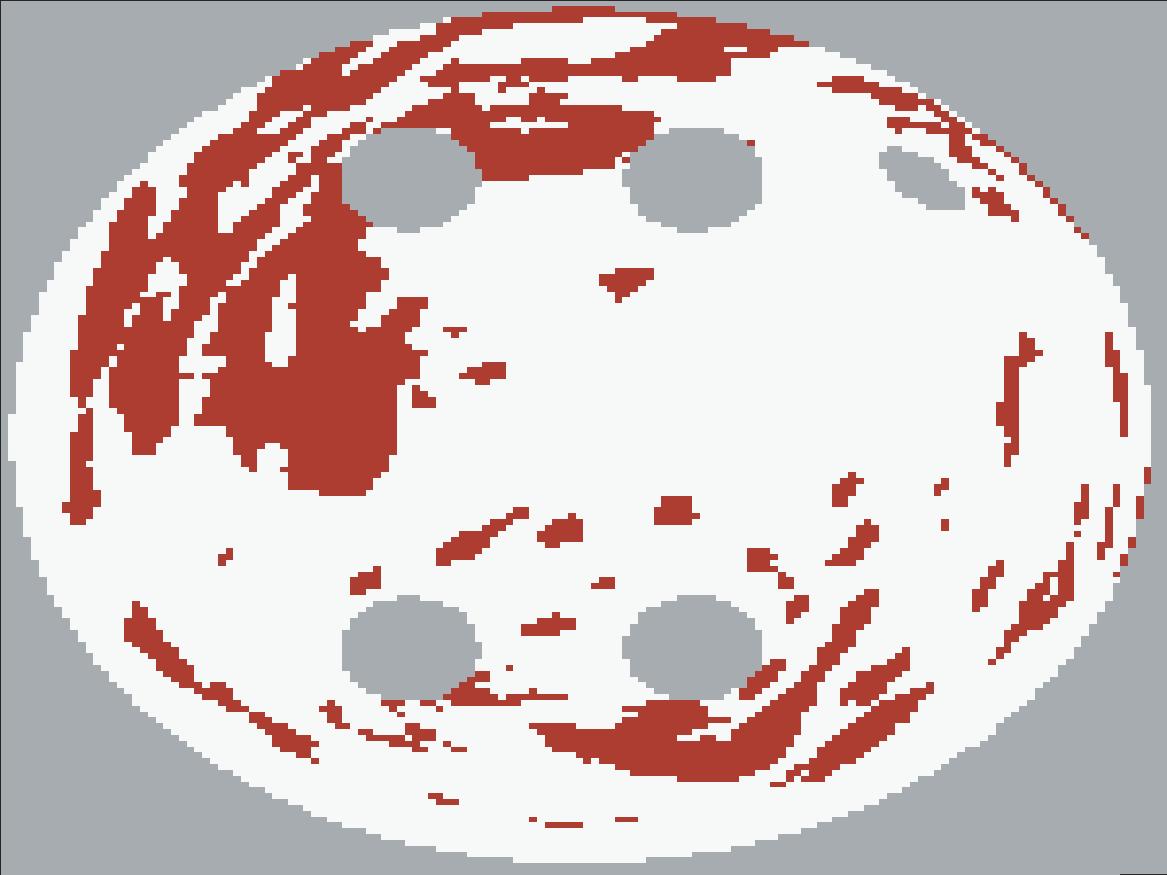

Supplement: Supplementary file 2 [file DataSheet1.ZIP › Dataset/real_PCQR1.jpg]

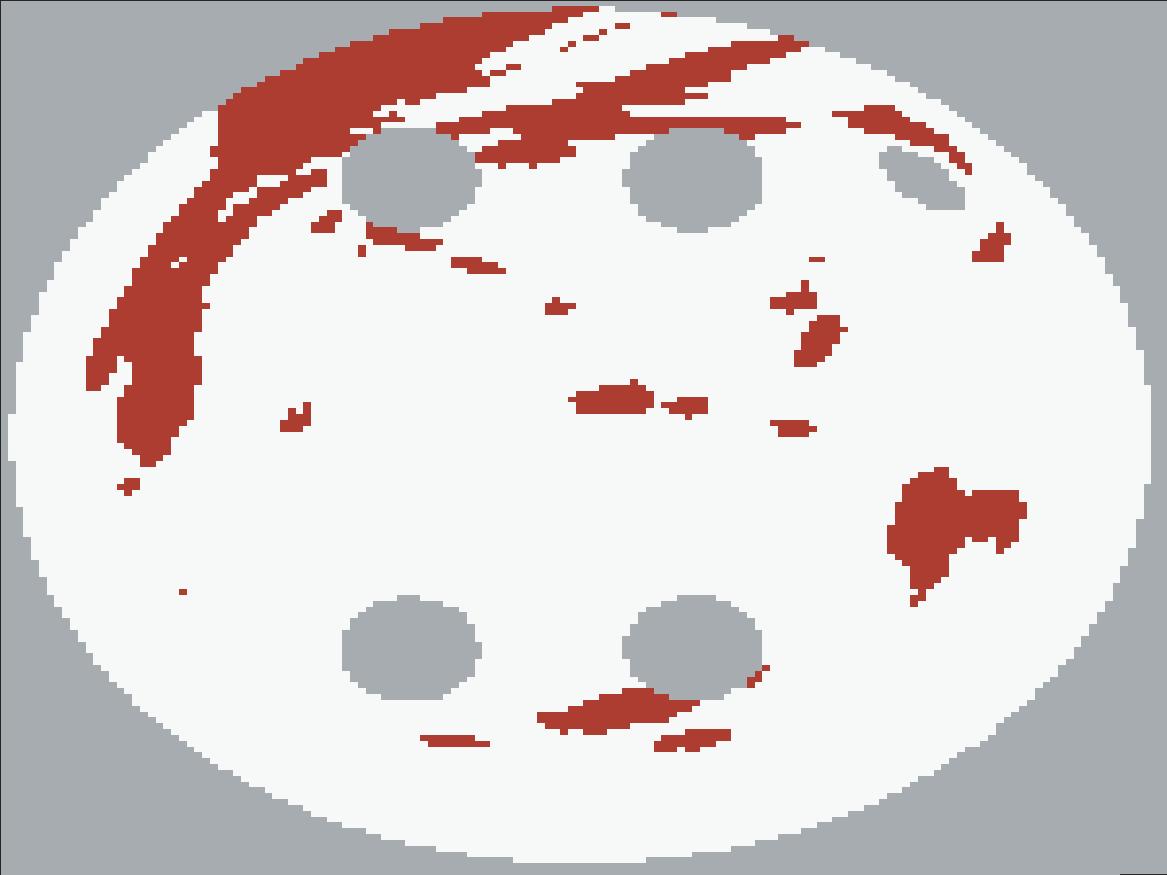

Supplement: Supplementary file 2 [file DataSheet1.ZIP › Dataset/real_PQ2M7.jpg]

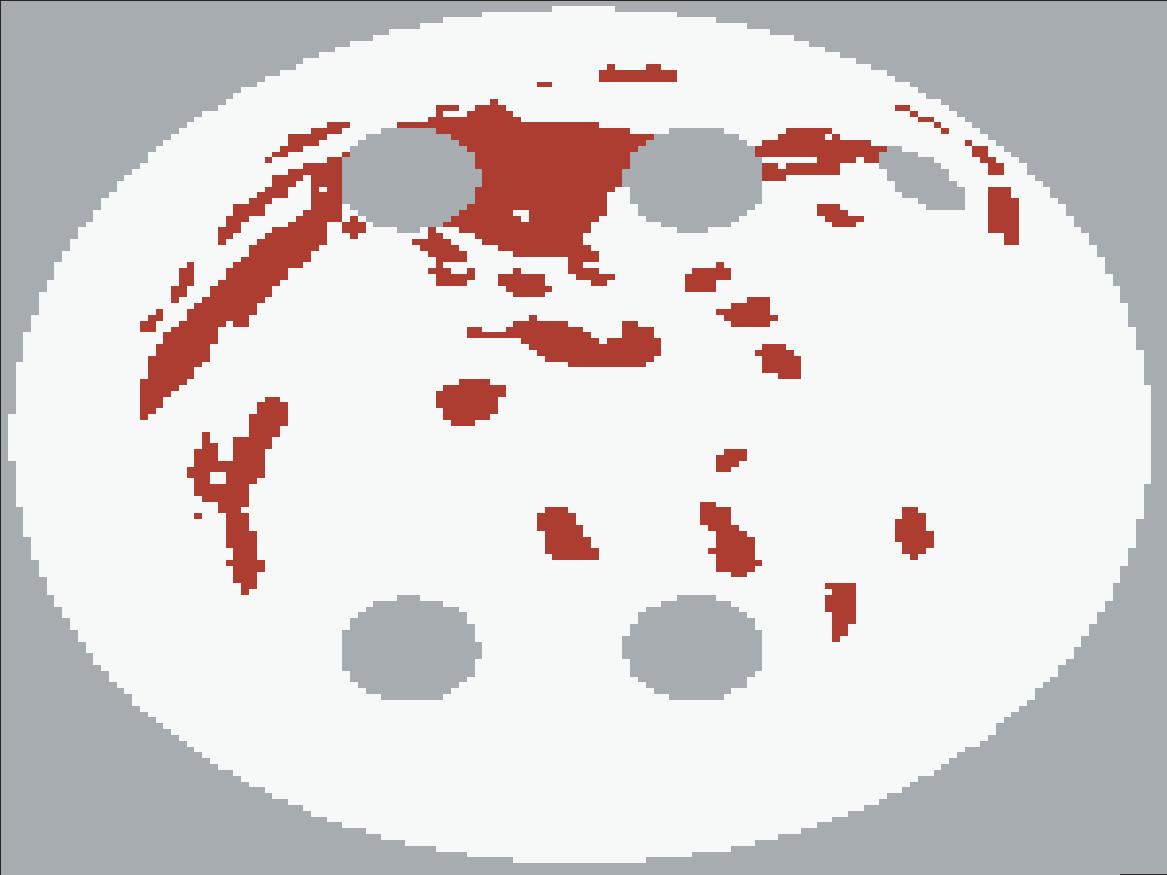

Supplement: Supplementary file 2 [file DataSheet1.ZIP › Dataset/real_Q38JP.jpg]

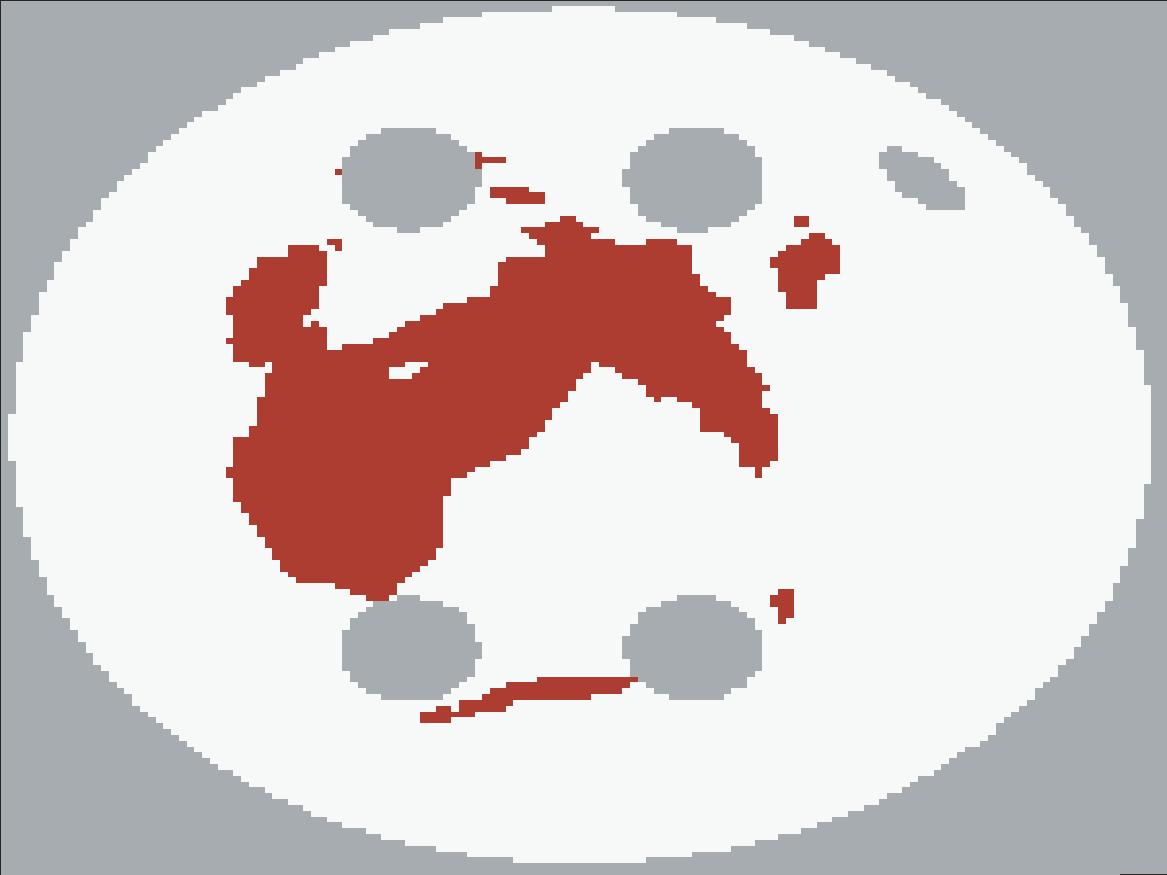

Supplement: Supplementary file 2 [file DataSheet1.ZIP › Dataset/real_Q60FT.jpg]

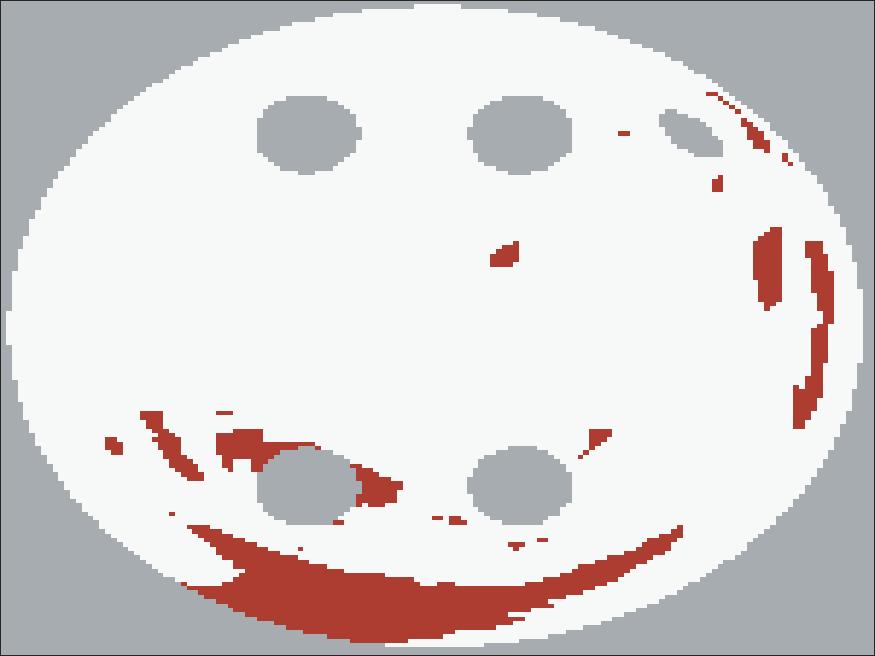

Supplement: Supplementary file 2 [file DataSheet1.ZIP › Dataset/real_QHOG2.jpg]

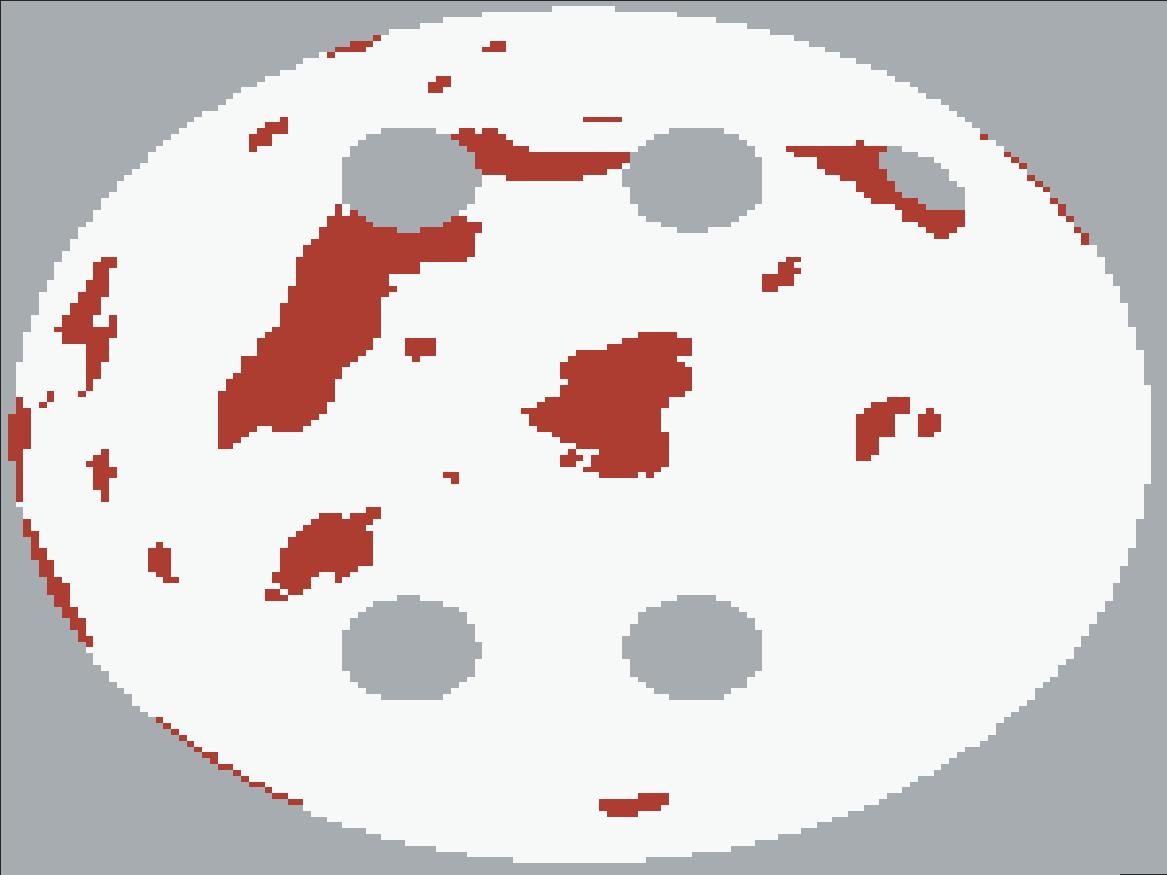

Supplement: Supplementary file 2 [file DataSheet1.ZIP › Dataset/real_QMIGN.jpg]

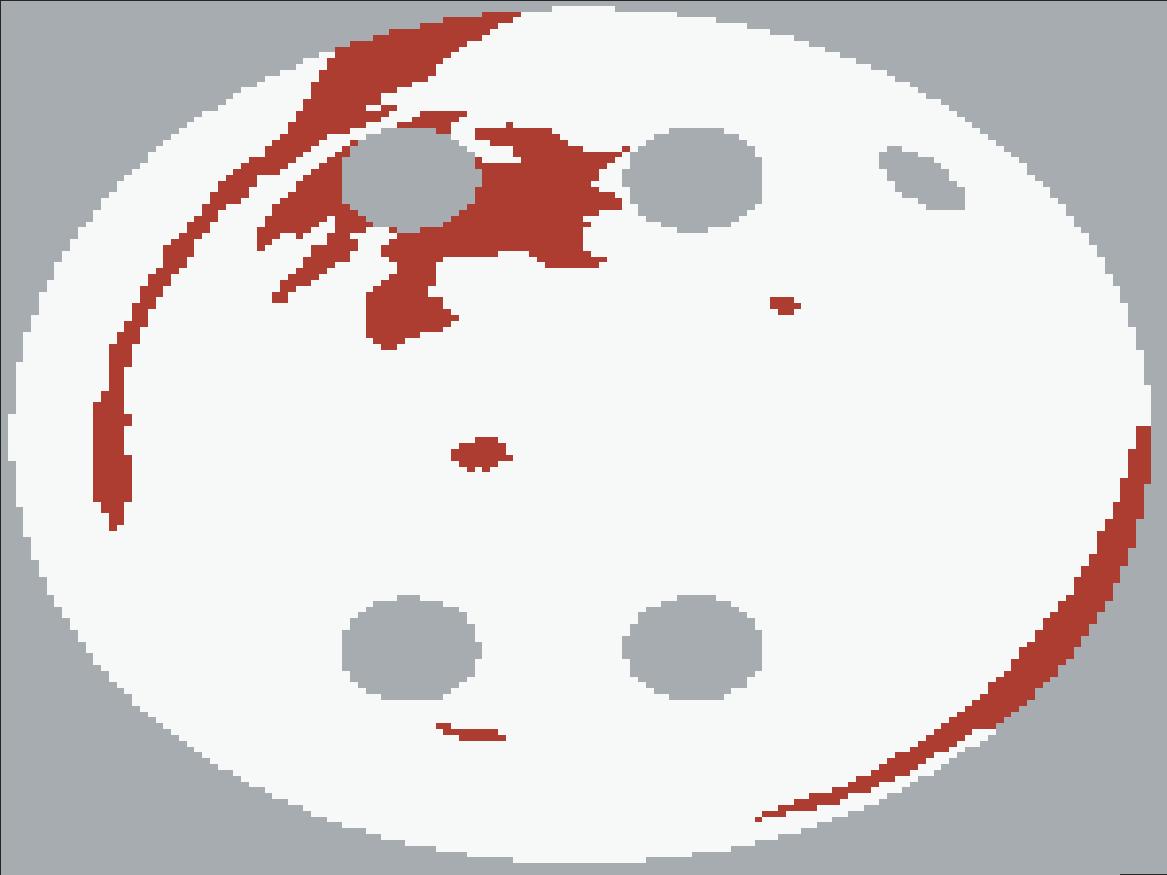

Supplement: Supplementary file 2 [file DataSheet1.ZIP › Dataset/real_QUYHG.jpg]

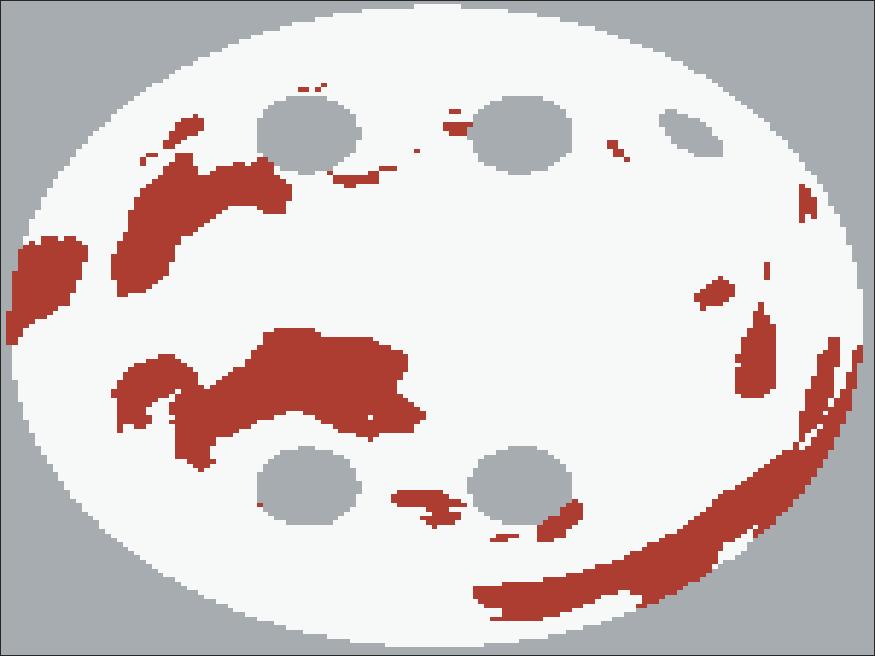

Supplement: Supplementary file 2 [file DataSheet1.ZIP › Dataset/real_RPH1P.jpg]

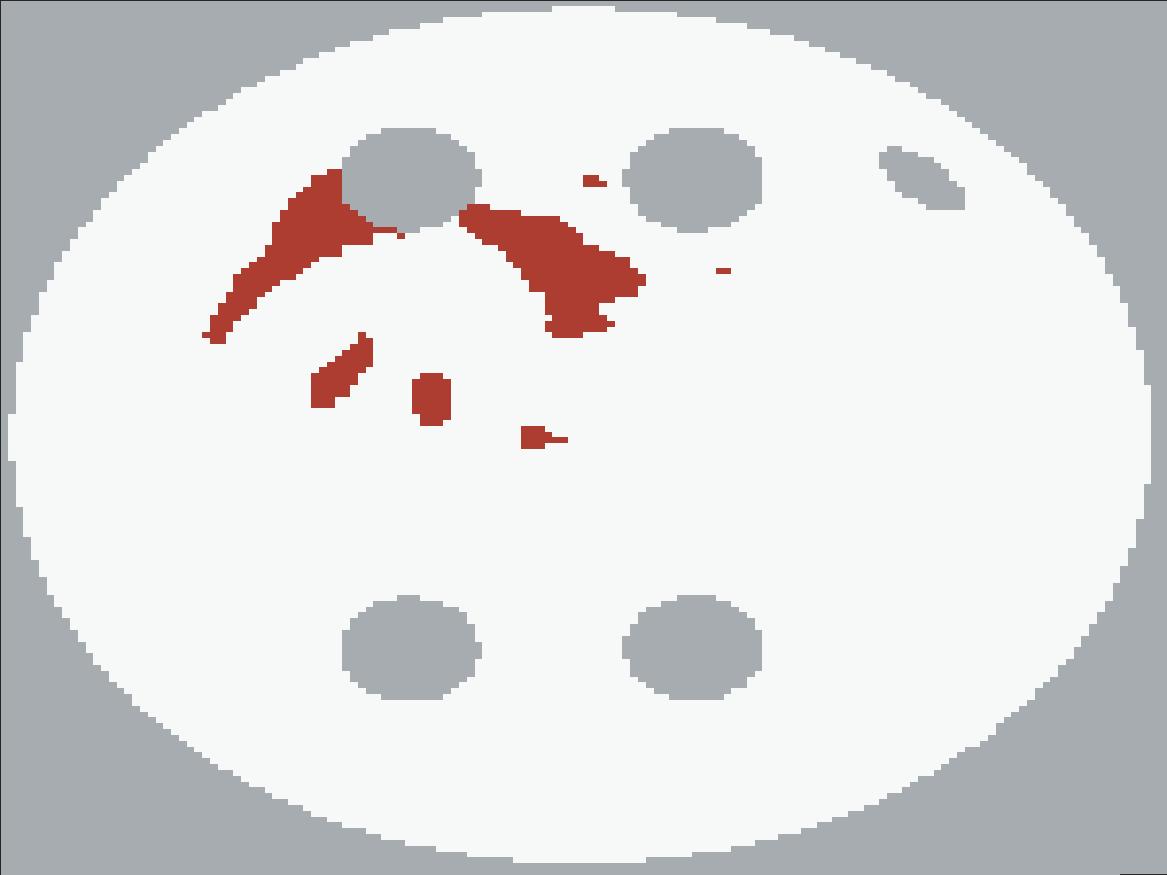

Supplement: Supplementary file 2 [file DataSheet1.ZIP › Dataset/real_RXAMM.jpg]

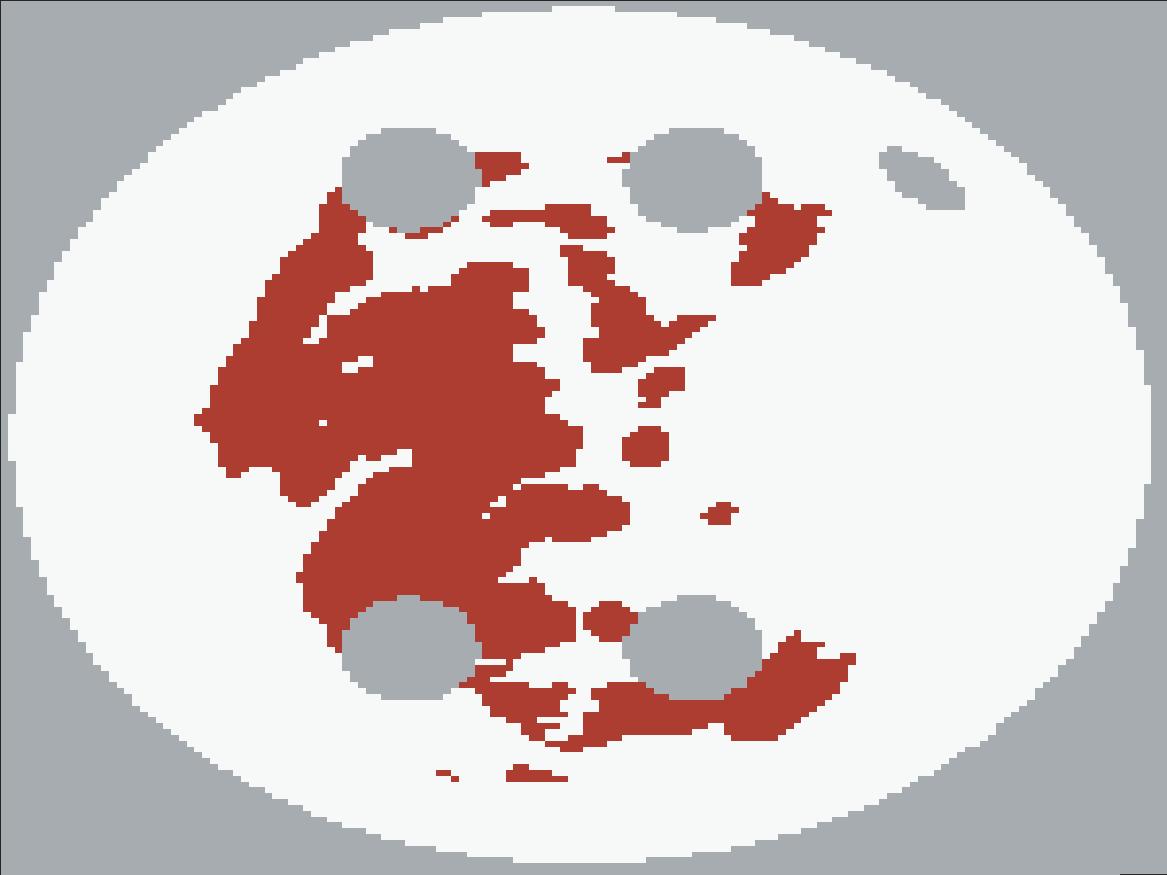

Supplement: Supplementary file 2 [file DataSheet1.ZIP › Dataset/real_SC4DX.jpg]
